# Diagnostic accuracy of ultrasound models for assessment of ovarian tumors: systematic review and meta‐analysis

**DOI:** 10.1002/uog.70135

**Published:** 2025-12-04

**Authors:** E. Lems, A. H. Koch, E. J. L. G. Delvaux, J. C. Leemans, M. Y. Bongers, C. A. R. Lok, B. L. Ramaekers, P. M. A. J. Geomini

**Affiliations:** ^1^ Department of Obstetrics and Gynaecology Máxima Medical Center Veldhoven The Netherlands; ^2^ Maastricht University Medical Center and Research School Grow Maastricht The Netherlands; ^3^ Department of Gynaecologic Oncology Center of Gynaecologic Oncology Amsterdam, location Antoni van Leeuwenhoek, Netherlands Cancer Institute Amsterdam The Netherlands; ^4^ Department of Research and Medical Innovation Máxima Medical Center Veldhoven The Netherlands; ^5^ Department of Clinical Epidemiology and Medical Technology Assessment Maastricht University Maastricht The Netherlands

**Keywords:** meta‐analysis, ovarian cancer, risk assessment, ultrasound

## Abstract

**Objective:**

Accurate preoperative classification of ovarian tumors is essential for guiding treatment. There is an increasing body of data evaluating ultrasound‐based models for this purpose in diverse clinical settings. The aim of this systematic review and meta‐analysis was to generate up‐to‐date evidence on the diagnostic accuracy of the most relevant ultrasound‐based models, including the Risk of Malignancy Index (RMI) versions 1, 2 and 3, Logistic Regression model 2 (LR2), Simple ultrasound‐based Rules (SR), the Assessment of Different NEoplasias in the adneXa (ADNEX) model and subjective assessment (SA), for the differentiation between benign and malignant ovarian tumors.

**Methods:**

Ovid/MEDLINE, EMBASE and the Cochrane Library were searched systematically from database inception until 19 June 2025. Eligible studies investigated the diagnostic accuracy of at least one of the preselected models, collected model parameters prospectively and provided sufficient data to construct 2 × 2 tables. The risk of bias of all included studies was assessed using the Quality Assessment of Diagnostic Accuracy Studies (QUADAS)‐2 and QUADAS‐C extension tools. Pooled summary estimates of sensitivity and specificity for all included models were calculated and bivariate models were fitted into hierarchical summary receiver‐operating‐characteristics curves. Bivariate random‐effects meta‐regression analysis was conducted to determine significant differences in sensitivity and specificity between models. Subgroup analyses were conducted according to menopausal status and prevalence of ovarian malignancy.

**Results:**

A total of 99 studies were included, describing 42 496 ovarian tumors, of which 31 371 (74%) were benign and 11 125 (26%) were malignant. SA had both high sensitivity (90.2% (95% CI, 87.8–92.2%)) and high specificity (91.4% (95% CI, 89.3–93.2%)). SR followed by SA of inconclusive cases (SR + SA) showed similar performance to SA (sensitivity, 88.6% (95% CI, 85.7–91.0%); *P* = 0.397 and specificity, 91.0% (95% CI, 89.0–92.7%); *P* = 0.811), as did the ADNEX model with a cut‐off of 20% (sensitivity, 86.7% (95% CI, 80.6–91.0%); *P* = 0.095; specificity, 87.9% (95% CI, 80.1–92.9%), *P* = 0.119). The ADNEX model with a cut‐off of 10% had a similar sensitivity to SA (92.7% (95% CI, 90.8–94.2%); *P* = 0.130), but lower specificity (78.4% (95% CI, 71.7–83.8%); *P* < 0.001). Higher cut‐offs of the ADNEX model led to a decrease in sensitivity, whereas lower cut‐offs resulted in reduced specificity. The LR2 model with a 10% cut‐off had a sensitivity of 89.5% (95% CI, 85.8–92.4%) and a specificity of 82.3% (95% CI, 75.0–87.8%). The RMI had the lowest diagnostic accuracy, with a sensitivity of 69.7% (95% CI, 67.0–72.2%) and a specificity of 90.5% (95% CI, 88.3–92.4%) for RMI version 1 with a cut‐off of 200. Subgroup analyses showed that both menopausal status and prevalence of malignancy significantly affected sensitivity (*P* < 0.01) and specificity (*P* < 0.01). Postmenopausal status and higher disease prevalence were associated with lower specificity, while sensitivity was lower in premenopausal women.

**Conclusions:**

All approaches, except for the RMI, performed well and could be used to differentiate between benign and malignant ovarian tumors. Although SA with or without SR had the highest diagnostic performance, it is dependent on operator expertise. If a strategy independent of operator expertise is preferred, the ADNEX model is recommended. Because of the high sensitivity of the ADNEX model, the likelihood of missing malignancies is low. In postmenopausal women, however, the reduced specificity may warrant a higher cut‐off, depending on how the impact of a false‐positive test result is evaluated. © 2025 The Author(s). *Ultrasound in Obstetrics & Gynecology* published by John Wiley & Sons Ltd on behalf of International Society of Ultrasound in Obstetrics and Gynecology.

## INTRODUCTION

Ovarian tumors are a common finding in women of all ages^1^, ^2^. Annually, approximately 1 in 1500 to 1 in 1700 women undergo surgery for an ovarian tumor. Only a minority of tumors (10–17%) are found to be malignant^3^, ^4^. Accurate preoperative characterization of ovarian tumors is critical as the optimal treatment pathway depends on the histology of the tumor. Women with an ovarian malignancy should be referred to an oncology center to undergo surgical staging by a gynecological oncologist^5^, ^6^. In contrast, women with a benign ovarian tumor can be treated in a general hospital, either with minimally invasive surgery or conservative treatment, thereby reducing complications and morbidity, and minimizing the effect on normal activity^6^, ^7^, ^8^.

To guide referral and treatment decisions, various models are available, mostly based on the ultrasound features of the ovarian tumor. Previous studies have demonstrated the high diagnostic accuracy of subjective assessment (SA) by an expert ultrasonographer in distinguishing between benign and malignant ovarian tumors^9^. SA is based on image recognition and thus requires significant exposure and training. However, if an expert is not available, ultrasound‐based models are useful alternatives^6^. The Risk of Malignancy Index (RMI) was the preferred strategy in The Netherlands until 2021 and is still recommended in several international guidelines^10^, ^11^. The International Ovarian Tumor Analysis (IOTA) group have developed several ultrasound models, including the Logistic Regression model 2 (LR2)^12^, Simple ultrasound‐based Rules (SR)^13^ and Assessment of Different NEoplasias in the adneXa (ADNEX) model^14^, all of which outperform the RMI in study settings^9^, ^15^. Previously, Meys *et al*.^9^, Westwood *et al*.^15^ and Davenport *et al*.^16^ performed systematic reviews and meta‐analyses that compared these models. However, none of them included all currently relevant ultrasound models. In addition, several new studies on the diagnostic value of the aforementioned models have been published in recent years, highlighting the need for an update of the existing reviews. Therefore, the aim of this study was to generate up‐to‐date evidence on the diagnostic accuracy of ultrasound‐based models for the differentiation between benign and malignant ovarian tumors.

## METHODS

This systematic review and meta‐analysis were conducted and reported in accordance with PRISMA guidelines^17^. The protocol of this study was registered in the international prospective register of systematic reviews (PROSPERO) in December 2022 (CRD42022282438)^18^.

### Eligibility criteria

Studies were eligible for inclusion if they investigated the diagnostic accuracy of at least one of RMI, LR2, SR, ADNEX and/or SA for the differentiation between benign and malignant ovarian tumors. We focused on the most widely used and clinically implemented models. While we recognize the relevance of other validated models, such as the Simple Rules Risk assessment (SRR), the Risk of Ovarian Malignancy Algorithm (ROMA) and scoring systems developed by Ferrazzi *et al*., Lerner *et al*. and Sassone *et al*., we opted not to include them in order to maintain a clear and focused scope^19^, ^20^, ^21^. Both prospective studies and retrospective studies were eligible for inclusion, provided that ultrasound model variables were collected prospectively and determined at the time of the ultrasound examination. Studies applying models retrospectively, such as to stored images or videos, were excluded because this does not reflect real‐world clinical practice wherein models are used prospectively to guide treatment decisions. No specific requirements were imposed on the execution of the ultrasound scan or the skill level or profession of the ultrasound examiner. Moreover, studies had to provide sufficient data to construct a 2 × 2 table of test performance or clinical outcomes.

Studies that investigated RMI versions 1, 2 and 3 at the most commonly used cut‐offs of 200 and/or 250 (RMI 1/2/3 − 200 and − 250, respectively) were included. For LR2, only studies investigating the widely used 10% cut‐off point (LR2 − 10%) were included. For SR, we considered studies that considered all inconclusive cases following application of SR as malignant (SR + Mal strategy) and those in which all inconclusive cases were reassessed by expert SA (SR + SA strategy). Articles were also eligible if the SR + Mal strategy was not reported in the publication but the available data allowed its calculation. Different cut‐off values for the ADNEX model are currently reported in the literature, in addition to the commonly used 10% cut‐off^22^. Therefore, studies with different cut‐off values were included in the review to provide a comprehensive overview of the available data. The meta‐analysis was restricted to the cut‐off values of 5%, 10%, 20%, 30% and 40% (ADNEX − 5%, − 10%, − 20%, − 30% and − 40%, respectively). Both versions of the ADNEX model, with and without cancer antigen 125 (CA‐125), were regarded as a single strategy because the addition of CA‐125 does not appear to affect the diagnostic accuracy of the model^23^. In case a study validated both versions of the ADNEX model, the version with CA‐125 was included. For SA, studies were eligible for inclusion if the assessment was based only on ultrasonographic examination of the ovarian tumor and clinical information. For both SR and SA, studies were excluded if they did not report clearly how inconclusive results were handled or if inconclusive results were explicitly excluded. If a study investigated multiple models but not all met the eligibility criteria, only those models that fulfilled our criteria were included. Studies from all healthcare settings were included, that is, those conducted in oncology centers, non‐oncology centers and mixed settings, as well as those in which the study setting was not specified.

We excluded case reports, reviews and model development studies. Additionally, we excluded studies that lacked full‐text availability, did not evaluate the diagnostic accuracy of one of the investigated models, were not available in Dutch or English language, had unclear eligibility status (e.g. unclear data collection method (prospective or retrospective) or unclear approach for classifying borderline tumors), used an incorrect definition of the RMI or menopausal status, included a subpopulation (e.g. only women with a specific risk profile based on other risk scores or specific tumor characteristics, only pregnant women, only children or only women with a history of ovarian cancer treatment), focused on only a specific histological subtype of ovarian cancer, lacked sufficient data to construct a 2 × 2 table, used a reference standard other than histology and/or follow‐up for at least 1 year or were not peer‐reviewed.

### Search strategy

Ovid/MEDLINE, EMBASE and the Cochrane Library were searched systematically from database inception until 19 June 2025 by a medical information specialist (E.J.L.G.D.). Keywords for the different risk‐assessment strategies and the target condition of ovarian cancer were used. The complete search strategy is provided in Table S1. In addition, references of systematic reviews, guidelines and relevant articles were searched manually for additional eligible records. Duplicate articles were excluded.

### Selection process

Two reviewers (E.L., A.H.K.) independently reviewed the eligibility of all identified records and selected full‐text articles for inclusion, using the review management tools Rayyan^24^ and Covidence (Veritas Health Innovation, Melbourne, Australia). Any disagreements were resolved by discussion or in consultation with a third reviewer (P.M.A.J.G.). If there was doubt about study eligibility due to missing information, the original authors were contacted for more information, after which a final decision was made. If the information was not provided and it remained unclear whether an article fulfilled the inclusion criteria, the article was excluded on the grounds of ‘unclear eligibility status’.

### Data extraction

Data extraction was performed independently by two researchers (E.L., A.H.K.) using a predesigned data‐extraction sheet. Data regarding study characteristics, study population, index and reference test characteristics and prevalence of ovarian malignancy were extracted. For studies investigating SA, the experience of the ultrasound examiner was recorded, preferably using the European Federation of Societies for Ultrasound in Medicine and Biology (EFSUMB) classification if reported^25^. The original study authors were consulted to clarify data queries. If the same population was reported in multiple publications, the publication with the largest population for each strategy was selected for data extraction. Data concerning pre‐ and postmenopausal women were extracted separately if possible to enable additional subgroup analyses. An overview of the data items extracted is provided in Table S2.

### Risk‐of‐bias assessment

The methodological quality of all included studies was assessed independently by two researchers (E.L., A.H.K.) using the Quality Assessment of Diagnostic Accuracy Studies (QUADAS)‐2 tool^26^. In addition, when multiple models were investigated in a single study, the QUADAS‐C extension was used. For each of the four domains, patient selection, index test, reference standard, and flow and timing, the risk of bias was classified as ‘low’, ‘high’ or ‘unclear’ (Appendix S1). Any disagreements were resolved through discussion and, if necessary, by consulting a third researcher (P.M.A.J.G.). None of the included studies were listed in the Retraction Watch Database^27^. Figures to present the risk of bias in the included studies were created using the Robvis package in R version 4.2.2 (R Foundation for Statistical Computing, Vienna, Austria)^28^.

### Statistical analysis

The primary outcomes were sensitivity and specificity for ovarian cancer of each strategy at different thresholds, as these are the most relevant parameters to guide clinical decision‐making. Statistical analysis was performed using the statistical program R (version 4.2.2), using the mada package for the meta‐analysis of diagnostic accuracy and the metafor package for assessing publication bias. For all strategies (RMI 1, RMI 2, RMI 3, LR2, SR + Mal, SR + SA, ADNEX, SA), the individual study results per cut‐off value were grouped and plotted graphically with 95% CI. Additionally, pooled positive and negative predictive values were calculated for all models and plotted graphically. Pairs of sensitivity and specificity were jointly analyzed using a bivariate model (Reitsma) to produce pooled summary estimates of sensitivity and specificity with 95% CI. These estimates were transformed into summary point estimates and, when appropriate, hierarchical summary receiver‐operating‐characteristics (HSROC) curves were generated from the fitted bivariate models. Heterogeneity was assessed primarily by visual inspection of the forest plots, prediction ellipses and 95% CI. Furthermore, a bivariate random‐effects meta‐regression, including all models with three or more studies as covariates, was performed to assess statistically significant differences in performance between the models. Funnel plots were used to assess publication bias and small‐study effects for sensitivity and specificity separately. The contour lines indicate conventional significance thresholds at the 10%, 5% and 1% levels (corresponding to CIs of 90%, 95% and 99%, respectively). Funnel plot asymmetry was evaluated using Egger's regression test^29^. Studies that classified borderline tumors as benign or excluded them were analyzed separately and excluded from the primary analysis. *P*‐values of < 0.05 were considered statistically significant.

The following subgroup analyses were performed to investigate potential sources of heterogeneity, including only models with three or more studies for each subgroup: menopausal status (pre‐ *vs* postmenopausal); prevalence of malignancy (low *vs* high, using the first quartile (Q1) of prevalence of ovarian cancer across the included studies as the cut‐off, as well as using prevalence as a continuous variable); reference standard (histology *vs* histology and follow‐up); overall risk of bias (low *vs* high, with studies rated unclear or high in any domain classified as ‘overall high’); and classification of borderline tumors (malignant *vs* benign). To investigate the impact of these covariates, each covariate was first examined individually. Subsequently, the covariate was included together with the model as an interaction term to explore potential effect modification. For covariates that significantly affected sensitivity or specificity (i.e. were considered effect modifiers), stratified analyses were subsequently performed.

## RESULTS

### Study selection and characteristics

**Figure 1:**
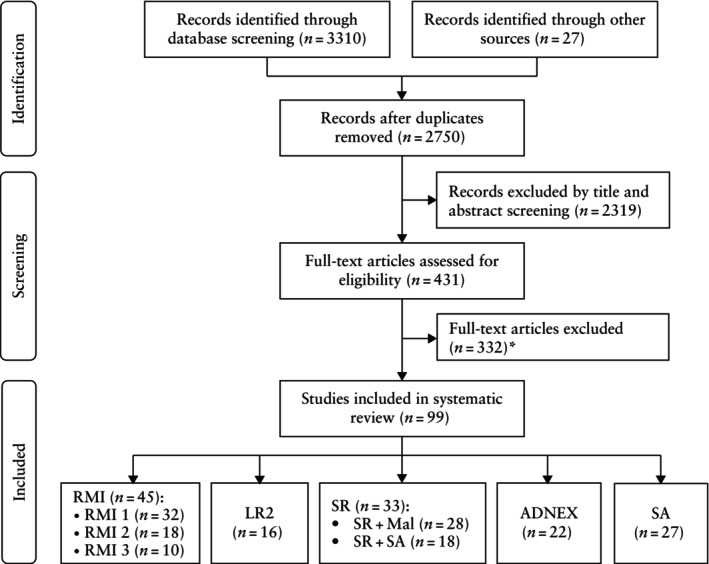
PRISMA flowchart summarizing inclusion of studies in the systematic review. *Reasons for exclusion are provided in Table S3. ADNEX, Assessment of Different NEoplasias in the adneXa; LR2, Logistic Regression model 2; RMI 1/2/3, Risk of Malignancy Index versions 1, 2 and 3; SA, subjective assessment; SR + Mal, inconclusive cases following application of Simple Rules (SR) considered as malignant (Mal); SR + SA, inconclusive cases following application of SR reassessed by SA.

Our search strategy identified 3310 potentially eligible articles from the Ovid/MEDLINE (*n* = 1451), EMBASE (*n* = 1629) and Cochrane (*n* = 230) databases, and a further 27 articles were identified through manual review of references (Figure 1). After removing duplicates and applying the inclusion and exclusion criteria, 99 articles were included. Excluded studies are detailed in Table S3. A total of 42 496 ovarian tumors were described, of which 31 371 (74%) were benign and 11 125 (26%) were malignant. Most of the included studies (76/99 (77%)) were prospective. In several studies (*n* = 34), multiple strategies were investigated. Studies investigating both variations of the SR (SR + Mal and SR + SA) were regarded as single‐strategy studies. The majority of studies (70/99 (71%)) were conducted in a single center, while the largest multicenter study involved 23 centers. Most studies (70/99 (71%)) were conducted in an oncology center, but 22/99 (22%) were carried out in a mixed setting, 4/99 (4%) in a non‐oncology center and in 3/99 (3%) studies the setting was unclear. The reported prevalence of malignancy ranged from 3.0% to 63.9%, with borderline tumors counted as malignant (Figure S1). A total of 32 studies were included for RMI 1^30^, ^31^, ^32^, ^33^, ^34^, ^35^, ^36^, ^37^, ^38^, ^39^, ^40^, ^41^, ^42^, ^43^, ^44^, ^45^, ^46^, ^47^, ^48^, ^49^, ^50^, ^51^, ^52^, ^53^, ^54^, ^55^, ^56^, ^57^, ^58^, ^59^, ^60^, ^61^, 18 studies for RMI 2^30^, ^32^, ^33^, ^35^, ^37^, ^44^, ^45^, ^59^, ^62^, ^63^, ^64^, ^65^, ^66^, ^67^, ^68^, ^69^, ^70^, ^71^, 10 studies for RMI 3^30^, ^35^, ^37^, ^44^, ^45^, ^70^, ^72^, ^73^, ^74^, ^75^, 16 studies for LR2^34^, ^44^, ^46^, ^49^, ^51^, ^55^, ^58^, ^74^, ^76^, ^77^, ^78^, ^79^, ^80^, ^81^, ^82^, ^83^, 32 studies for SR^34^, ^36^, ^44^, ^47^, ^48^, ^49^, ^51^, ^52^, ^55^, ^69^, ^80^, ^84^, ^85^, ^86^, ^87^, ^88^, ^89^, ^90^, ^91^, ^92^, ^93^, ^94^, ^95^, ^96^, ^97^, ^98^, ^99^, ^100^, ^101^, ^102^, ^103^, ^104^ (of which 28 reported on the SR + Mal strategy and 18 on the SR + SA strategy), 22 studies for the ADNEX model^34^, ^44^, ^52^, ^58^, ^60^, ^69^, ^74^, ^83^, ^105^, ^106^, ^107^, ^108^, ^109^, ^110^, ^111^, ^112^, ^113^, ^114^, ^115^, ^116^, ^117^, ^118^ and 27 studies for SA^34^, ^40^, ^44^, ^46^, ^49^, ^51^, ^55^, ^69^, ^74^, ^77^, ^78^, ^81^, ^86^, ^87^, ^90^, ^92^, ^112^, ^119^, ^120^, ^121^, ^122^, ^123^, ^124^, ^125^, ^126^, ^127^, ^128^. In 16/27 studies investigating SA, it was stated specifically that all ultrasound scans were performed by expert examiners or EFSUMB Level‐3 examiners^44^, ^55^, ^77^, ^78^, ^81^, ^86^, ^87^, ^90^, ^92^, ^119^, ^121^, ^122^, ^123^, ^124^, ^125^, ^128^. In one study, no information was provided regarding the level of experience of the examiner^120^. All other studies involved EFSUMB Level‐2 examiners, a combination of EFSUMB Level‐2 and ‐3 examiners, or ‘experienced examiners’ without further definition^34^, ^40^, ^46^, ^49^, ^51^, ^69^, ^74^, ^112^, ^126^, ^127^. In five studies, borderline tumors were classified as benign or were excluded (Table S4)^30^, ^52^, ^66^, ^118^, ^129^. However, in two of these studies, this applied only to additional data; the original reports classified borderline tumors as malignant, so the studies were included in the primary meta‐analysis^52^, ^118^. Characteristics of the studies included in the systematic review are summarized in Table S5.

### Risk of bias

**Figure 2:**
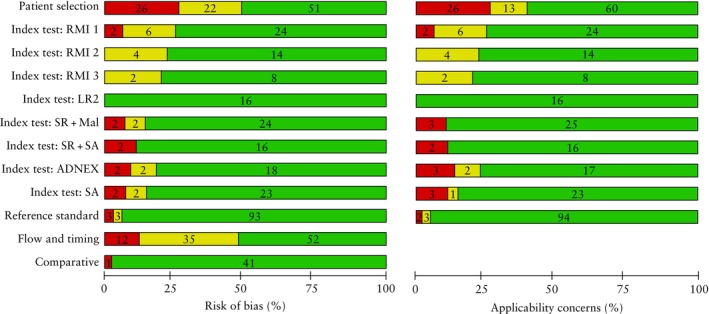
Summary of quality assessment of included studies using Quality Assessment of Diagnostic Accuracy Studies (QUADAS)‐2 and QUADAS‐C extension tools. Annotations indicate number of studies. 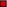, high; 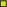, unclear; 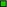, low. ADNEX, Assessment of Different NEoplasias in the adneXa; LR2, Logistic Regression model 2; Mal, malignant; RMI 1/2/3, Risk of Malignancy Index versions 1, 2 and 3; SA, subjective assessment; SR + Mal, inconclusive cases following application of Simple Rules (SR) considered as malignant (Mal); SR + SA, inconclusive cases following application of SR reassessed by SA.

The results of the risk‐of‐bias assessment using the QUADAS‐2 and QUADAS‐C extension tools are summarized in Figure 2. Detailed information regarding the risk‐of‐bias assessment in individual studies can be found in Table S6. For the patient selection, index test, reference standard, and flow and timing domains, 51 (52%), 81 (82%), 93 (94%) and 52 (53%) studies, respectively, were judged to have a low risk of bias. Only 41% of studies explicitly stated that patients were collected consecutively, and in 45% of studies there was a risk of inappropriate patient exclusions. In 56% of studies, the time interval between the index test(s) and the reference standard was considered appropriate, i.e. < 120 days. All included studies that investigated multiple risk‐assessment strategies in the same population were scored as low risk using the QUADAS‐C tool because all studies used a fully paired design and applied the index tests at the same time.

Visual inspection of the funnel plots suggested some evidence of publication bias because smaller studies showed higher sensitivity and specificity estimates (Figure S2). Egger's regression test was statistically significant (*P* < 0.001) for both outcomes, confirming funnel plot asymmetry. Visual inspection of the funnel plots stratified by strategy showed a similar pattern, suggesting that the presence of publication bias was consistent across strategies and not caused by a single strategy biasing the overall result.

### Primary analysis

**Figure 3:**
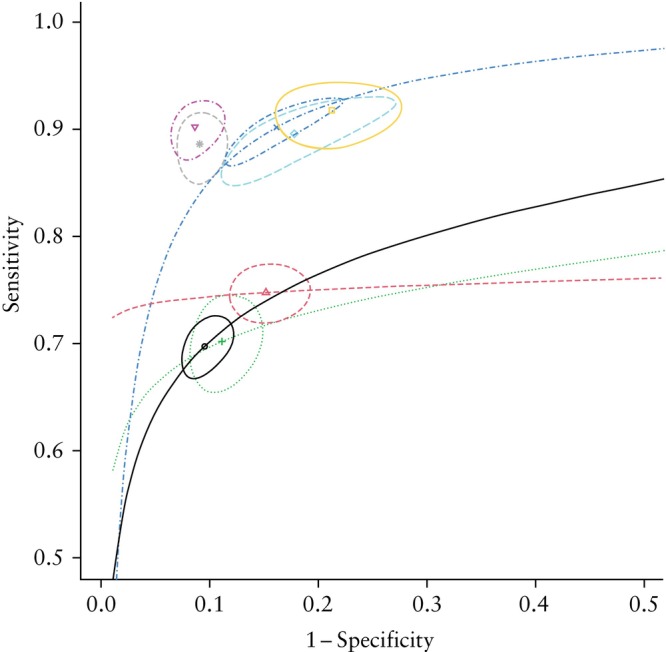
Summary point estimates of sensitivity and specificity with 95% CI for all models. For models with multiple thresholds, hierarchical summary receiver‐operating‐characteristics curves were fitted additionally. 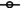, Risk of Malignancy Index (RMI) 1; 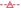, RMI 2; 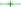, RMI 3; 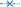, Assessment of Different NEoplasias in the adneXa (ADNEX); 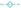, Logistic Regression model 2 (LR2) with 10% cut‐off; 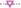, subject assessment (SA); 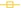, Simple Rules (SR) + malignant; 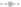, SR + SA.

A total of 96 studies in which borderline tumors were classified as malignant were included in the primary analysis. The pooled summary estimates of sensitivity and specificity for each model are shown in Table 1 and Figure 3. The individual study results and estimated pooled predictive values, grouped by model and cut‐off, are presented in Figures S3 and S4. In the overall population, SA was the only model for which both sensitivity and specificity exceeded 90% (90.2% (95% CI, 87.8–92.2%) and 91.4% (95% CI, 89.3–93.2%), respectively). Additionally, the strategies SR + Mal, ADNEX – 5% and ADNEX – 10% had sensitivity > 90%, while specificity > 90% was observed for RMI 1 – 200, RMI 1 – 250, SR + SA, ADNEX – 30% and ADNEX – 40%. SA demonstrated the best diagnostic performance, followed closely by SR + SA; neither sensitivity nor specificity differed significantly between the two models based on meta‐regression analysis (*P* = 0.397 for sensitivity, *P* = 0.811 for specificity) (Table 2). Likewise, the sensitivity and specificity of ADNEX – 20% were not significantly different from those of SA (*P* = 0.095 for sensitivity, *P* = 0.119 for specificity) or SR + SA (*P* = 0.377 for sensitivity, *P* = 0.201 for specificity). SR + Mal demonstrated similar sensitivity, but significantly lower specificity compared with SA and SR + SA (*P* < 0.001 for both comparisons). In addition to SA and SR + SA, the ADNEX and LR2 models showed high overall diagnostic accuracy. Higher cut‐offs of the ADNEX model led to a decrease in sensitivity, whereas lower cut‐offs resulted in reduced specificity. All models outperformed the various versions of the RMI, with consistently higher sensitivities (Figure 3). Visual inspection of the forest plots and prediction ellipses suggested moderate heterogeneity (Figures S3 and S4).

**Table 1:** Pooled summary point estimates of sensitivity and specificity of all ultrasound‐based models evaluated in meta‐analysis for diagnosis of ovarian cancer

Model*	Studies (*n*)	Tumors (*n*)	Sensitivity (%) (95% CI)	Specificity (%) (95% CI)
RMI 1 – 200	29	16 203	69.7 (67.0–72.2)	90.5 (88.3–92.4)
RMI 1 – 250	7	7504	70.3 (64.6–75.5)	91.0 (83.4–95.3)
RMI 2 – 200	16	5672	74.8 (72.5–77.0)	84.8 (81.2–87.7)
RMI 2 – 250	2	216	73.3 (61.4–82.6)	85.3 (77.0–90.9)
RMI 3 – 200	8	2321	70.5 (66.5–74.2)	88.9 (85.6–91.5)
RMI 3 – 250	1	158	66.7 (52.8–78.2)	88.8 (81.3–93.5)
LR2 – 10%	15	12 647	89.5 (85.8–92.4)	82.3 (75.0–87.8)
SR + Mal†	28	12 968	91.7 (89.0–93.9)	78.8 (73.7–83.1)
SR + SA‡	18	9137	88.6 (85.7–91.0)	91.0 (89.0–92.7)
ADNEX – 5%	12	11 179	95.9 (94.2–97.2)	68.2 (56.0–78.4)
ADNEX – 10%	21	13 971	92.7 (90.8–94.2)	78.4 (71.7–83.8)
ADNEX – 20%	10	10 409	86.7 (80.6–91.0)	87.9 (80.1–92.9)
ADNEX – 30%	6	9069	74.1 (62.5–83.0)	94.4 (89.8–97.0)
ADNEX – 40%	4	7888	62.0 (44.9–76.6)	97.5 (95.2–98.8)
SA	26	11 557	90.2 (87.8–92.2)	91.4 (89.3–93.2)

Only studies in which borderline tumors were classified as malignant are included.

* For RMI, LR2 and ADNEX models, cut‐offs are specified.

† Inconclusive cases following application of Simple Rules (SR) considered as malignant (Mal).

‡ Inconclusive cases reassessed by subjective assessment (SA). ADNEX, Assessment of Different NEoplasias in the adneXa; LR2, Logistic Regression model 2; RMI 1/2/3, Risk of Malignancy Index versions 1, 2 and 3.

**Table 2:** Meta‐regression analysis of sensitivity and specificity of ultrasound‐based models for diagnosis of ovarian cancer

Model*	RMI 1 – 250	RMI 2 – 200	RMI 3 – 200	LR2 – 10%	SR + Mal†	SR + SA‡	ADNEX – 5%	ADNEX – 10%	ADNEX – 20%	ADNEX – 30%	ADNEX – 40%	SA
RMI 1 – 200												
Sens	0.649	0.129	0.736	< 0.001	< 0.001	< 0.001	< 0.001	< 0.001	< 0.001	0.400	0.234	< 0.001
Spec	0.960	0.015	0.483	0.002	< 0.001	0.778	< 0.001	< 0.001	0.255	0.108	0.001	0.562
RMI 1 – 250												
Sens		0.511	0.927	< 0.001	< 0.001	0.001	< 0.001	< 0.001	0.001	0.747	0.186	< 0.001
Spec		0.103	0.615	0.032	0.002	0.812	< 0.001	0.002	0.428	0.186	0.005	0.673
RMI 2 – 200												
Sens			0.447	< 0.001	< 0.001	< 0.001	< 0.001	< 0.001	0.001	0.789	0.043	< 0.001
Spec			0.271	0.474	0.083	0.014	0.001	0.072	0.369	0.002	< 0.001	0.004
RMI 3 – 200												
Sens				< 0.001	< 0.001	< 0.001	< 0.001	< 0.001	0.001	0.681	0.216	< 0.001
Spec				0.101	0.011	0.389	< 0.001	0.011	0.793	0.064	0.001	0.279
LR2 – 10%												
Sens					0.399	0.630	< 0.001	0.106	0.200	< 0.001	< 0.001	0.770
Spec					0.340	0.002	0.006	0.305	0.131	< 0.001	< 0.001	< 0.001
SR + Mal†												
Sens						0.155	0.002	0.359	0.030	< 0.001	< 0.001	0.527
Spec						< 0.001	0.027	0.888	0.011	< 0.001	< 0.001	< 0.001
SR + SA‡												
Sens							< 0.001	0.028	0.377	< 0.001	< 0.001	0.397
Spec							< 0.001	<0.001	0.201	0.180	0.002	0.811
ADNEX – 5%												
Sens								0.026	< 0.001	< 0.001	< 0.001	< 0.001
Spec								0.046	< 0.001	< 0.001	< 0.001	< 0.001
ADNEX – 10%												
Sens									0.005	< 0.001	< 0.001	0.130
Spec									0.011	< 0.001	< 0.001	< 0.001
ADNEX – 20%												
Sens										0.004	< 0.001	0.095
Spec										0.025	< 0.001	0.119
ADNEX – 30%												
Sens											0.113	< 0.001
Spec											0.083	0.220
ADNEX – 40%												
Sens												< 0.001
Spec												0.002

Presented are *P*‐values for sensitivity (Sens) and specificity (Spec). Statistically significant differences are displayed in color: green indicates higher sensitivity or specificity of model in column compared with model in row; red color lower sensitivity or specificity of model in column compared with model in row. Only models for which three or more studies were included in meta‐analysis are shown.

* For RMI, LR2 and ADNEX models, cut‐offs are specified.

† Inconclusive cases following application of Simple Rules (SR) considered as malignant (Mal).

‡ Inconclusive cases reassessed by subjective assessment (SA). ADNEX, Assessment of Different NEoplasias in the adneXa; LR2, Logistic Regression model 2; RMI 1/2/3, Risk of Malignancy Index versions 1, 2 and 3.

### Subgroup analyses

#### Menopausal status

In total, 24 studies reported data on premenopausal women and 25 studies reported data on postmenopausal women. For all strategies except for RMI 2 – 250, RMI 3 – 250, ADNEX – 30% and ADNEX – 40%, separate data for pre‐ and postmenopausal women were available. Menopausal status impacted both sensitivity (*P* < 0.001) and specificity (*P* < 0.001). Forest plots with individual study results for pre‐ and postmenopausal women are displayed in Figures S5 and S6, respectively. For all strategies, specificity was higher in premenopausal women compared with postmenopausal women, especially for LR2 – 10% (88.9% (95% CI, 81.3–93.7%) *vs* 68.0% (95% CI, 64.5–71.3%)) and the ADNEX model (83.5% (95% CI, 73.9–90.1%) *vs* 61.4% (95% CI, 47.7–73.5%) for ADNEX – 10%) (Table 3, Figure S7). Only RMI 1, SR + SA and SA had a specificity > 80% in postmenopausal women. Sensitivity was lower in premenopausal women compared with postmenopausal women, particularly for RMI (53.9% (95% CI, 47.7–60.0%) *vs* 77.1% (95% CI, 72.5–81.1%) for RMI 1 – 200). In both groups, SA and SR + SA yielded the highest overall performance (Table S7). Among premenopausal women, SR + Mal and ADNEX also performed well. In postmenopausal women, these models also demonstrated the highest performance following SA and SR + SA, although they incurred a substantial decrease in specificity, which, for the ADNEX model, varied depending on the selected cut‐off value.

**Table 3:** Pooled summary point estimates of sensitivity and specificity of ultrasound‐based models for ovarian cancer in pre‐ *vs* postmenopausal women

Model*	Studies (*n*)	Tumors (*n*)	Sensitivity (%) (95% CI)	Specificity (%) (95% CI)
RMI 1 – 200				
Premenopausal	12	3493	53.9 (47.7–60.0)	93.5 (91.3–95.2)
Postmenopausal	13	3989	77.1 (72.5–81.1)	85.2 (79.7–89.5)
RMI 1 – 250				
Premenopausal	1	356	54.8 (37.4–71.1)	88.3 (84.3–91.4)
Postmenopausal	2	1286	75.3 (65.1–83.2)	84.4 (75.8–90.3)
RMI 2 – 200				
Premenopausal	5	1092	55.7 (47.0–64.0)	91.3 (87.9–93.9)
Postmenopausal	5	1006	81.8 (76.1–86.4)	74.1 (62.6–83.1)
RMI 3 – 200				
Premenopausal	2	419	49.3 (27.8–71.1)	93.2 (88.4–96.1)
Postmenopausal	2	315	83.7 (76.2–89.2)	79.4 (55.7–92.2)
LR2 – 10%				
Premenopausal	6	2443	82.2 (78.2–85.6)	88.9 (81.3–93.7)
Postmenopausal	6	1921	86.8 (68.3–95.2)	68.0 (64.5–71.3)
SR + Mal†				
Premenopausal	5	2222	92.3 (87.7–95.2)	84.2 (77.8–89.0)
Postmenopausal	6	2919	93.4 (88.3–96.3)	73.7 (66.1–80.1)
SR + SA‡				
Premenopausal	6	2280	87.8 (82.9–91.5)	95.2 (92.7–96.9)
Postmenopausal	6	1972	89.4 (85.3–92.4)	86.0 (83.3–89.8)
ADNEX – 5%				
Premenopausal	1	320	93.7 (85.7–97.3)	52.3 (46.0–58.5)
Postmenopausal	1	253	98.1 (92.6–99.5)	22.1 (16.2–29.5)
ADNEX – 10%				
Premenopausal	6	1419	88.1 (81.2–92.7)	83.5 (73.9–90.1)
Postmenopausal	7	2236	94.4 (90.5–96.8)	61.4 (47.7–73.5)
ADNEX – 20%				
Premenopausal	1	320	87.3 (78.0–93.1)	80.1 (74.6–84.7)
Postmenopausal	1	253	97.1 (91.4–99.1)	63.8 (55.7–71.1)
SA				
Premenopausal	9	2032	85.3 (80.7–89.0)	93.8 (91.0–95.8)
Postmenopausal	9	1734	90.8 (87.6–93.2)	83.4 (78.1–87.6)

Only models for which three or more studies were included in meta‐analysis for each subgroup are shown.

* For RMI, LR2 and ADNEX models, cut‐offs are specified.

† Inconclusive cases following application of Simple Rules (SR) considered as malignant (Mal).

‡ Inconclusive cases reassessed by subjective assessment (SA). ADNEX, Assessment of Different NEoplasias in the adneXa; LR2, Logistic Regression model 2; RMI 1/2/3, Risk of Malignancy Index versions 1, 2 and 3.

#### Prevalence of ovarian cancer

The prevalence of ovarian cancer affected both sensitivity (*P* = 0.001) and specificity (*P* < 0.001). The relationships between sensitivity and prevalence, and between specificity and prevalence are shown in Figure S8. Subsequently, a subgroup analysis was performed in which studies were divided into low *vs* high prevalence, based on the Q1 of prevalence across studies, which was 21.1%. Of the 96 studies included in the meta‐analysis, 24 (25%) were classified as low and 72 (75%) as high prevalence. Generally, specificity was lower in studies in which the population had a high prevalence of ovarian cancer (Table 4). In both low‐ and high‐prevalence studies, SA and SR + SA demonstrated the highest diagnostic performance, followed by the ADNEX model, SR + Mal and LR2 (Table S8, Figures S9–S11).

**Table 4:** Pooled summary point estimates of sensitivity and specificity of ultrasound‐based models for ovarian cancer in studies with low (< 21.1%) *vs* high (≥ 21.1%) disease prevalence

Model*	Studies (*n*)	Tumors (*n*)	Sensitivity (%) (95% CI)	Specificity (%) (95% CI)
RMI 1 – 200				
Low prevalence	5	1240	65.2 (54.5–74.6)	94.8 (89.5–97.5)
High prevalence	24	14 963	70.6 (67.8–73.2)	89.9 (87.6–91.8)
RMI 1 – 250				
Low prevalence	1	540	73.3 (64.1–80.9)	86.9 (83.4–89.8)
High prevalence	6	6964	69.9 (63.2–75.9)	91.6 (82.7–96.1)
RMI 2 – 200				
Low prevalence	5	1658	76.9 (70.9–81.9)	88.4 (86.4–90.2)
High prevalence	11	4014	74.5 (71.9–77.0)	83.0 (78.0–87.1)
RMI 2 – 250				
Low prevalence	0	—	—	—
High prevalence	2	216	73.3 (61.4–82.6)	85.3 (77.0–90.9)
RMI 3 – 200				
Low prevalence	3	568	70.1 (56.2–81.1)	89.9 (86.6–92.4)
High prevalence	5	1753	70.5 (65.9–74.7)	88.4 (83.0–92.2)
RMI 3 – 250				
Low prevalence	0	—	—	—
High prevalence	1	158	66.7 (52.8–78.2)	88.8 (81.3–93.5)
LR2 – 10%				
Low prevalence	3	501	92.6 (83.8–96.8)	90.8 (72.5–97.4)
High prevalence	12	12 146	90.4 (85.6–93.7)	80.2 (72.1–86.4)
SR + Mal†				
Low prevalence	6	1921	93.4 (88.6–96.3)	89.4 (76.9–95.6)
High prevalence	22	11 047	91.7 (88.4–94.2)	75.4 (70.8–79.6)
SR + SA‡				
Low prevalence	4	1334	91.2 (83.7–95.4)	94.7 (89.8–97.3)
High prevalence	14	7803	88.6 (85.3–91.3)	90.3 (88.2–92.1)
ADNEX – 5%				
Low prevalence	4	7883	93.8 (88.1–96.8)	81.4 (65.1–91.1)
High prevalence	8	3296	96.5 (94.7–97.7)	60.0 (46.9–71.8)
ADNEX – 10%				
Low prevalence	5	3167	85.1 (79.2–89.5)	87.7 (76.7–93.9)
High prevalence	16	10 804	93.5 (92.1–94.7)	74.5 (67.4–80.4)
ADNEX – 20%				
Low prevalence	3	2977	83.1 (51.4–95.8)	95.6 (92.1–97.6)
High prevalence	7	7432	89.3 (84.7–92.6)	82.2 (73.8–88.3)
ADNEX – 30%				
Low prevalence	2	2620	57.0 (49.1–64.5)	97.7 (96.8–98.4)
High prevalence	4	6449	80.6 (74.2–85.7)	91.4 (86.7–94.6)
ADNEX – 40%				
Low prevalence	2	2620	48.6 (39.9–57.4)	98.6 (97.1–99.3)
High prevalence	2	5268	73.2 (61.1–82.7)	95.2 (94.5–95.9)
SA				
Low prevalence	2	254	87.5 (71.1–95.2)	96.6 (92.9–98.3)
High prevalence	24	11 303	90.4 (88.0–92.4)	91.0 (88.7–92.9)

Only models for which three or more studies were included in meta‐analysis for each subgroup are shown.

* For RMI, LR2 and ADNEX models, cut‐offs are specified.

† Inconclusive cases following application of Simple Rules (SR) considered as malignant (Mal).

‡ Inconclusive cases reassessed by subjective assessment (SA). ADNEX, Assessment of Different NEoplasias in the adneXa; LR2, Logistic Regression model 2; RMI 1/2/3, Risk of Malignancy Index versions 1, 2 and 3.

#### Reference standard

No statistically significant differences were found in sensitivity or specificity between studies using histology *vs* those using histology and follow‐up as the reference standard (*P* > 0.05 for all comparisons).

#### Risk of bias

No statistically significant differences were found in sensitivity or specificity between studies with ‘low’ overall risk of bias *vs* those with ‘high’ overall risk of bias (*P* > 0.05 for all comparisons).

#### Classification of borderline tumors

Because of the limited number of studies that classified borderline tumors as benign (Table S4), no subgroup analysis could be performed for the classification of borderline tumors as benign *vs* malignant.

## DISCUSSION

### Main findings

In this systematic review and meta‐analysis, we evaluated and summarized the evidence on the diagnostic accuracy of ultrasound‐based strategies for the differentiation between benign and malignant ovarian tumors. Our results indicate that LR2, SR, ADNEX and SA have good diagnostic performance and outperform the RMI. SA demonstrated both high sensitivity and high specificity. The SR + SA strategy yielded comparable performance to SA, followed by the ADNEX and LR2 models. The selected cut‐off value of all models compromised either sensitivity or specificity. In high‐prevalence settings and in postmenopausal women, the specificity of the models was lower, whereas sensitivity was lower in premenopausal women, especially for RMI.

Our results are largely consistent with previous literature, with sensitivities and specificities comparable to those reported by Meys *et al*.^9^, Westwood *et al*.^15^, Barreñada *et al*.^23^ and Davenport *et al*.^16^. The similarity between our pooled sensitivity and specificity estimates and those reported in previous reviews underscores the reliability of our data and suggests that the inclusion of more recent studies has not altered significantly the pooled estimates of diagnostic performance^9^, ^15^, ^16^, ^23^.

### Strengths and limitations

A strength of our extensive systematic review is the large number of included studies (*n* = 99) and ovarian tumors (*n* = 42 496). Its added value lies in the up‐to‐date analysis and direct comparison of all key prediction models, including SA and ADNEX with various cut‐offs, thereby offering a more comprehensive overview compared with previous literature. Because of the strict selection criteria, the majority of included studies had a low risk of bias. The data from this review are applicable to routine clinical practice because we applied no restrictions on the experience of the ultrasound operator. In addition, only studies with prospective data collection were included, and we considered studies using either histology or histology and follow‐up as the reference standard, further ensuring alignment with routine clinical practice.

Limitations include the restriction to studies in English and Dutch language because of insufficient proficiency in other languages, as well as the poor author response rate to requests for additional data, which led to the exclusion of studies due to unclear eligibility. We did not include all models described in the literature and focused on the most clinically relevant ultrasound‐based models. This resulted in the exclusion of two‐step strategies, with the exception of SR + SA, as well as studies investigating artificial intelligence because these models have not yet been validated externally^130^, ^131^. We considered ADNEX with and without CA‐125 as a single strategy, which precluded evaluation of the added value of CA‐125. However, the study of Barreñada *et al*.^23^ showed that including CA‐125 does not substantially improve diagnostic accuracy and only a few included studies allowed for such a comparison. Another limitation is that we focused exclusively on threshold‐specific sensitivity and specificity, without reporting areas under the receiver‐operating‐characteristics curve as summary measures of overall model performance. However, to support clinical decision‐making, we prioritized the metrics that are directly interpretable in a clinical context. In addition, we did not assess calibration performance, which reflects how well predicted risks agree with observed outcomes. Furthermore, we identified publication bias, suggesting that smaller studies with less favorable results may be under‐represented. This bias may have led to an overestimation of diagnostic performance. Lastly, limitations of the evidence included in our review are the small number of studies classifying borderline tumors as benign, the small number of studies utilizing the ADNEX model with higher cut‐off values, the predominance of studies conducted in oncology centers and the limited reporting of data stratified by menopausal status, precluding robust stratified analyses relevant to clinical practice.

### Implications for clinical practice and research

Findings from this meta‐analysis demonstrate that use of the RMI should be avoided, especially for premenopausal women, as more accurate models are available. Given the evidence, SA or SR + SA are the recommended strategies. In contrast to pure SA, which requires expert review of all cases, SR + SA applies the SR first and refers only the inconclusive cases (in this systematic review, 19%) for subjective evaluation by an experienced examiner. This substantially reduces the workload for experienced examiners while preserving high diagnostic accuracy. However, previous studies demonstrated that experience affects the diagnostic accuracy of SA^126^, ^132^, ^133^. If it is preferred that SA is avoided because of the impact of examiner uncertainty on performance^134^, the ADNEX model is the most suitable alternative. Unlike SR, it yields no inconclusive results and provides risk estimates as percentages. Compared with LR2, ADNEX offers the additional advantage of multiclass risk prediction and allows the selection of a specific cut‐off to balance sensitivity and specificity tailored to the healthcare setting, whereas LR2 has only been validated extensively at a 10% threshold. Because of the high sensitivity of the ANDEX model, the likelihood of missing malignancies is low. In postmenopausal women, however, the reduced specificity may warrant a higher cut‐off, depending on how the impact of a false‐positive test result is evaluated. Use of the RMI is discouraged due to its lower sensitivity and the associated risk of missing malignancies. Besides referral decisions, risk models can also support the choice between surgery and conservative management. However, most of the studies included in our review lacked a conservative (non‐surgical) arm. Outcome data for women with low predicted risk who were managed expectantly are largely absent from the included literature. As a result, our meta‐analysis cannot provide evidence‐based conclusions about the safety or appropriateness of very low cut‐off values (e.g. 1%) for conservative management.

To enhance applicability, future studies should assess the performance of the ADNEX model at various cut‐offs, in pre‐ and postmenopausal women, and investigate accuracy when borderline tumors are classified as benign because optimal thresholds may vary by population. Assessment of diagnostic performance alone is not comprehensive; potential harm of false‐positive and false‐negative referrals should also be considered. Additionally, a cost‐effectiveness analysis is essential and results may vary depending on the logistics of a healthcare system. In The Netherlands, this is currently being investigated in the ACCEPT study (trial number: NL9572)^135^.

### Conclusions

LR2, SR, ADNEX and SA all outperform the RMI for the preoperative differentiation between benign and malignant ovarian tumors. Of these strategies, SA and SR + SA have the best diagnostic performance in all subgroups. If a strategy independent of operator expertise is preferred, the ADNEX model is recommended. Because of the high sensitivity of the ANDEX model, the likelihood of missing malignancies is low. In postmenopausal women, however, the reduced specificity may warrant a higher cut‐off, depending on how the impact of a false‐positive test result is evaluated.

## Supporting information


**Table S1** Search strategy.
**Table S2** Information extracted from each study.
**Table S3** Excluded studies and reason for exclusion.
**Table S4** Individual study results not included in analyses.
**Table S5** Characteristics of included studies.
**Table S6** Quality assessment of included studies.
**Table S7** Meta‐regression analysis for pre‐ *vs* postmenopausal women.
**Table S8** Meta‐regression analysis for studies with low (< 21.1%) *vs* high (≥ 21.1%) prevalence of ovarian cancer.
**Appendix S1** QUADAS‐2 and QUADAS‐C tools.
**Figure S1** Prevalence and size of included studies.
**Figure S2** Funnel plots for sensitivity and specificity.
**Figure S3** Forest plots of all included models and cut‐off values.
**Figure S4** Estimated pooled predictive values of included models.
**Figure S5** Forest plots with individual study results for subgroup of premenopausal women.
**Figure S6** Forest plots with individual study results for subgroup of postmenopausal women.
**Figure S7** Summary point estimates of sensitivity and specificity, and hierarchical summary receiver‐operating‐characteristics curves for subgroups of pre‐ and postmenopausal women.
**Figure S8** Sensitivity, specificity and prevalence of ovarian cancer in included studies.
**Figure S9** Forest plots with individual study results for studies with low prevalence (< 21.1%) of ovarian cancer.
**Figure S10** Forest plots with individual study results for studies with high prevalence (≥ 21.1%) of ovarian cancer.
**Figure S11** Summary point estimates of sensitivity and specificity, and hierarchical summary receiver‐operating‐characteristics curves for studies with low (< 21.1%) *vs* high (≥ 21.1%) prevalence of ovarian cancer.

## Data Availability

The data that support the findings of this study are available from the corresponding author upon reasonable request.
