# Supplementary material for: Diagnostic accuracy of ultrasound models for assessment of ovarian tumors: systematic review and meta‐analysis
Source: Ultrasound Obstet Gynecol. 2025 Dec 4;67(5):590–603. doi: 10.1002/uog.70135 (PMC13136063; doi:10.1002/uog.70135)
Supplement: Supplementary file 1 — Table S1 Search strategy. Table S2 Information extracted from each study. Table S3 Excluded studies and reason for exclusion. Table S4 Individual study results not included in analyses. Table S5 Characteristics of included studies. Table S6 Quality assessment of included studies. Table S7 Meta‐regression analysis for pre‐ vs postmenopausal women. Table S8 Meta‐regression analysis for studies with low (< 21.1%) vs high (≥ 21.1%) prevalence of ovarian cancer. Appendix S1 QUADAS‐2 and QUADAS‐C tools. Figure S1 Prevalence and size of included studies. Figure S2 Funnel plots for sensitivity and specificity. Figure S3 Forest plots of all included models and cut‐off values. Figure S4 Estimated pooled predictive values of included models. Figure S5 Forest plots with individual study results for subgroup of premenopausal women. Figure S6 Forest plots with individual study results for subgroup of postmenopausal women. Figure S7 Summary point estimates of sensitivity and specificity, and hierarchical summary receiver‐operating‐characteristics curves for subgroups of pre‐ and postmenopausal women. Figure S8 Sensitivity, specificity and prevalence of ovarian cancer in included studies. Figure S9 Forest plots with individual study results for studies with low prevalence (< 21.1%) of ovarian cancer. Figure S10 Forest plots with individual study results for studies with high prevalence (≥ 21.1%) of ovarian cancer. Figure S11 Summary point estimates of sensitivity and specificity, and hierarchical summary receiver‐operating‐characteristics curves for studies with low (< 21.1%) vs high (≥ 21.1%) prevalence of ovarian cancer. [file UOG-67-590-s001.docx]

**Table S1** Search strategy

**Search Medline/OVID 19-06-2025**

| **#** | **Searches** | **Results** |
| --- | --- | --- |
| 12 | 4 AND 10 AND 11 | 1.451 |
| 11 | exp Ultrasonography/ OR (ultraso* OR phonopheresis OR sonication OR sonification OR ultra sound OR ultrashell OR sonograph* OR doptone* OR echograph* OR echogram* OR echosound*).ti,ab,kf. | 820.804 |
| 10 | 5 OR 6 OR 7 OR 8 OR 9 | 1.454.380 |
| 9 | exp risk assessment/ OR exp risk factors/ OR ((risk ADJ4 malignan* ADJ4 factor*) OR (risk ADJ4 malignan* ADJ4 assessment) OR (risk ADJ4 malignan ADJ4 score*) OR RMI).ti,ab,kf. | 1.271.728 |
| 8 | (adnex* ADJ8 (model* OR score* OR assess*)).ti,ab,kf. | 714 |
| 7 | ((simple ADJ3 rules) OR (simple ADJ3 descriptors) OR SRrisk* OR b-rules OR m-rules).ti,ab,kf. | 2.856 |
| 6 | 'subjective assessment*'.ti,ab,kf. | 6.988 |
| 5 | exp logistic models/ OR (IOTA OR 'international ovarian tumor analysis' OR 'international ovarian tumour analysis' OR LR2 OR 'logistic model*' OR 'logistic regression model*').ti,ab,kf. | 257.779 |
| 4 | 1 OR 2 OR 3 | 641.302 |
| 3 | exp Peritoneal Neoplasms/ OR (peritoneum OR borderline OR epithelial OR primary peritoneal OR ((peritoneal OR peritoneal OR peritoneum) ADJ5 (cancer* OR adenocarcin* OR adeno-carcin* OR tumor* OR tumour* OR neoplas* OR metasta* OR meta sta* OR carcino* OR oncogenesis OR choriocarcinom* OR teratom* OR cystadenocarcin* OR rhabdomyosarcom* OR rhabdo-myosarcom* OR rhabdosarcom* OR leiomyosarcoma* OR leio-myosarcom* OR androblastom* OR arrhenoblastom* OR ledion OR neoplasia OR neoplastic OR sarcoma* OR malignan* OR adenoma* OR oncolo*))).ti,ab,kf | 524.196 |
| 2 | ((ovar* OR high-grade serous OR low-grade serous OR sertoli-leydig cell OR fallopian OR oviduct OR tubal) ADJ5 (cancer* OR adenocarcin* OR adeno-carcin* OR tumor* OR tumour* OR neoplas* OR metasta* OR meta-sta* OR carcino* OR oncogenesis OR choriocarcinom* OR teratom* OR cystadenocarcin* OR rhabdomyosarcom* OR rhabdo-myosarcom* OR rhabdosarcom* OR leiomyosarcoma* OR leio-myosarcom* OR androblastom* OR arrhenoblastom* OR ledion OR neoplasia OR neoplastic OR sarcoma* OR malignan* OR adenoma* OR oncolo*)).ti,ab,kf. | 130.588 |
| 1 | exp Ovarian Neoplasms/ OR Fallopian Tube Neoplasms/ | 103.337 |

**Search Embase 19-06-2025**

| **No.** | **Query** | **Results** |
| --- | --- | --- |
| #12 | #4 AND #10 AND #11 | 1.629 |
| #11 | 'echography'/exp OR ((ultraso*:ti,ab,kw OR phonopheresis:ti,ab,kw OR sonication:ti,ab,kw OR sonification:ti,ab,kw OR ultra:ti,ab,kw) AND sound:ti,ab,kw) OR ultrashell:ti,ab,kw OR sonograph*:ti,ab,kw OR doptone*:ti,ab,kw OR echograph*:ti,ab,kw OR echogram*:ti,ab,kw OR echosound*:ti,ab,kw | 1.225.346 |
| #10 | #5 OR #6 OR #7 OR #8 OR #9 | 2.973.577 |
| #9 | 'risk assessment'/exp OR 'risk factors'/exp OR ((risk NEAR/4 malignan* NEAR/4 factor*):ti,ab,kw) OR ((risk NEAR/4 malignan* NEAR/4 assessment):ti,ab,kw) OR ((risk NEAR/4 malignan NEAR/4 score*):ti,ab,kw) OR ((risk NEAR/4 malignan NEAR/4 index):ti,ab,kw) OR rmi:ti,ab,kw | 2.238.487 |
| #8 | (adnex* NEAR/8 (model* OR score* OR assess*)):ti,ab,kw | 1.131 |
| #7 | ((simple NEAR/3 rules):ti,ab,kw) OR ((simple NEAR/3 descriptors):ti,ab,kw) OR srrisk*:ti,ab,kw OR 'b rules':ti,ab,kw OR 'm rules':ti,ab,kw | 3.133 |
| #6 | 'subjective assessment*':ti,ab,kw | 10.043 |
| #5 | 'statistical model'/exp OR iota:ti,ab,kw OR 'international ovarian tumor analysis':ti,ab,kw OR 'international ovarian tumour analysis':ti,ab,kw OR lr2:ti,ab,kw OR 'logistic model*':ti,ab,kw OR 'logistic regression model*':ti,ab,kw | 909.643 |
| #4 | #1 OR #2 OR #3 | 282.732 |
| #3 | 'peritoneum tumor'/exp OR (((peritoneal OR peritoneal OR peritoneum) NEAR/5 (cancer* OR adenocarcin* OR 'adeno-carcin*' OR tumor* OR tumour* OR sarcoma* OR neoplas* OR metasta* OR 'meta sta*' OR carcino* OR oncogenesis OR choriocarcinom* OR teratom* OR cystadenocarcin* OR rhabdomyosarcom* OR 'rhabdo-myosarcom*' OR rhabdosarcom* OR leiomyosarcoma* OR 'leio-myosarcom*' OR androblastom* OR arrhenoblastom* OR ledion OR neoplasia OR neoplastic OR sarcoma* OR malignan* OR adenoma* OR oncolo*)):ti,ab,kw) | 58.065 |
| #2 | ((ovar* OR 'high-grade serous' OR 'low-grade serous' OR 'sertoli-leydig cell' OR fallopian OR oviduct OR tubal) NEAR/5 (cancer* OR adenocarcin* OR 'adeno-carcin*' OR tumor* OR tumour* OR neoplas* OR metasta* OR 'meta sta*' OR carcino* OR oncogenesis OR choriocarcinom* OR teratom* OR cystadenocarcin* OR rhabdomyosarcom* OR 'rhabdo myosarcom*' OR rhabdosarcom* OR leiomyosarcoma* OR 'leio-myosarcom*' OR androblastom* OR arrhenoblastom* OR ledion OR neoplasia OR neoplastic OR sarcoma* OR malignan* OR adenoma* OR oncolo*)):ti,ab,kw | 194.631 |
| #1 | 'ovary cancer'/exp OR 'uterine tube tumor'/exp OR 'uterine tube carcinoma'/exp | 177.348 |

**Search Cochrane 19-06-2025**

| **ID** | **Search** | **Hits** |
| --- | --- | --- |
| #23 | #10 AND #19 AND #22 | 230 |
| #22 | #20 OR #21 | 74914 |
| #21 | (ultraso* or phonopheresis or sonication or sonification or ultra sound or ultrashell or sonograph* or doptone* or echograph* or echogram* or echosound*):ti,ab,kw | 69514 |
| #20 | MeSH descriptor: [Ultrasonography] explode all trees | 19422 |
| #19 | #11 OR #12 OR #13 OR #14 OR #15 OR #16 OR #17 OR #18 | 236593 |
| #18 | ('risk malignant factor*' or 'risk malignancy factor*' OR 'risk assessment*' OR 'risk factor*' OR 'risk score* or RMI OR 'risk malignancy index'):ti,ab,kw | 203872 |
| #17 | MeSH descriptor: [Risk Factors] explode all trees | 37946 |
| #16 | MeSH descriptor: [Risk Assessment] explode all trees | 13580 |
| #15 | ('adnex* model*' OR ' adnex score*' OR ' adnex assess*):ti,ab,kw | 37 |
| #14 | ('simple rules' OR simple descriptors' OR srrisk* OR 'b rules' OR 'm rules'):ti,ab,kw | 915 |
| #13 | (subjective assessment):ti,ab,kw | 11725 |
| #12 | (IOTA or 'international ovarian tumor analysis' or 'international ovarian tumour analysis' or LR2 or 'logistic model*' or 'logistic regression model*'):ti,ab,kw | 19540 |
| #11 | MeSH descriptor: [Models, Statistical] explode all trees | 22698 |
| #10 | #5 OR #6 OR #9 | 28013 |
| #9 | #7 OR #8 | 19763 |
| #8 | (peritoneum or borderline or epithelial or primary peritoneal):ti,ab,kw | 19612 |
| #7 | MeSH descriptor: [Peritoneal Neoplasms] explode all trees | 581 |
| #6 | #3 AND #4 | 11521 |
| #5 | #1 OR #2 | 3683 |
| #4 | (cancer* OR adenocarcin* OR 'adeno-carcin*' OR tumor* OR tumour* OR neoplas* OR metasta* OR 'meta sta*' OR carcino* OR oncogenesis OR choriocarcinom* OR teratom* OR cystadenocarcin* OR rhabdomyosarcom* OR 'rhabdo myosarcom*' OR rhabdosarcom* OR leiomyosarcoma* OR 'leio-myosarcom*' OR androblastom* OR arrhenoblastom* OR ledion OR neoplasia OR neoplastic OR sarcoma* OR malignan* OR adenoma* OR oncolo*):ti,ab,kw | 313404 |
| #3 | (ovar* OR 'high-grade serous' OR 'low-grade serous' OR 'sertoli-leydig cell' OR fallopian OR oviduct OR tubal):ti,ab,kw | 27746 |
| #2 | MeSH descriptor: [Fallopian Tubes] explode all trees | 217 |
| #1 | MeSH descriptor: [Ovarian Neoplasms] explode all trees | 3484 |

**Table S2** Information extracted from each study

| **Category** | **Items** |
| --- | --- |
| **Study details** | Author |
|  | Year |
|  | Funding |
|  | Country |
| **Study design** | Study design |
|  | Randomization |
|  | Blinding |
|  | Time from test to surgery |
| **Recruitment details** | Recruitment start date |
|  | Recruitment end date |
|  | Single- or multicenter study |
|  | Number of centers |
|  | Study setting (2^nd^ or 3^rd^ line hospital, mixed) |
|  | Inclusion criteria |
|  | Exclusion criteria |
| **Ultrasound details** | Experience of examiners |
|  | Ultrasound technique used |
| **Sample size** | Number of included patients |
|  | Allocation of borderline tumors (as benign or malignant) |
| **Patient characteristics** | Menopausal status |
|  | Definition of menopausal status |
|  | Age |
|  | CA-125 |
| **Reference standard** | Reference standard (histology, histology and follow-up) |
|  | Reference standard details |
|  | Number and percentage of patients with histology |
| **Index test(s)** | Index test(s) used |
|  | Threshold(s) used |
|  | Subgroup data for menopausal status |
| **Test results** | Sensitivity |
|  | Specificity |
|  | Number of false-positives |
|  | Number of true-positives |
|  | Number of true-negatives |
|  | Number of false-negatives |
| **Prevalence** | Number and percentage of benign tumors |
|  | Number and percentage of borderline tumors |
|  | Number and percentage of malignant tumors |
|  | Origin of malignant tumors (epithelial, non-epithelial, metastatic non-primary ovarian) |
|  | FIGO stage |
| **QUADAS-2 + QUADAS-C** | Items of the Quality Assessment of Diagnostic Accuracy Studies (QUADAS)-2 and QUADAS-C checklist (S3) |

**Table S3** Excluded studies and reason for exclusion

| **Exclusion reason: only abstract/poster available (n = 49)** |
| --- |
| 1. Anthoulakis C, Bisxiniotis S, Zouzoulas D, Tsolakidis D, Chatzistamatiou K, Theodoulidis V, et al. External validation of the ADNEX model to triage adnexal masses in Greece: a tertiary center study conducted by non-expert sonographers. International Journal of Gynecological Cancer*.* 2022;32:A74. |
| 1. Anthoulakis C, Chatzoula M, Tsolakidis D, Theodoulidis V, Zouzoulas D, Chatzistamatiou K, et al. External validation of the ADNEX model to triage adnexal masses in Greece: updated data from a gynecologic oncology center. International Journal of Gynecological Cancer. 2024;34:A293-A4. |
| 1. Baker C, Pasipanodya T, Dwivedi R. The management of suspected ovarian masses in premenopausal women in a DGHsetting. BJOG: An International Journal of Obstetrics and Gynaecology. 2013;120:372. |
| 1. Barcelo IM, Cabedo L, Fusté P, Ros C, Sebastia MC, Munmany M. Inconclusive ovarian tumours by IOTA simple rules and application of O-RADS MRI scores in a tertiary referral centre. International Journal of Gynecological Cancer*. 2022*;32:A86-A7. |
| 1. Ben-Meir LC, Mashiach R, Eisenberg VH. External validation of the IOTA classification in women with ovarianmasses suspected to be endometrioma. Journal of Clinical Medicine. 2021;10(13). |
| 1. Borges A, Rodrigues S, Aguino J, Bernardo M, Mahomed F, Djokovic D. Performance of the IOTA ADNEX model in preoperative discrimination ofadnexal formations: A Portuguese prospective multicenter pilot study. Australasian Journal of Ultrasound in Medicine. 2019;22(2):145. |
| 1. Borges A, Pinto P, Ambrósio P, Condeço R, Martins A, Passarinho R, et al. Performance of the IOTA ADNEX model in differentiating between benign and malignant adnexal lesions in a Portuguese population. International Journal of Gynecological Cancer. 2022;32:A71-A2. |
| 1. Braicu E, Torsten U, Richter R, Zimmermann M, Chekerov R, Kronenberger C, et al. Value of biomarkers and sonography in predicting malignancy in pelvic masspatients. preliminary results from prospective, multicentric, ongoingstudy. International Journal of Gynecological Cancer. 2014;24(9):366-7. |
| 1. Bouguerra S, Souayeh N, Mbarki C, Bouguerra B. Comparison Of The Performance Of RMI, ORADS US, ORADS MRI, And IOTA Scores In Predicting Malignancy Of Adnexal Masses In Postmenopausal Women. International Journal of Gynecological Cancer. 2025;35(2). |
| 1. Bouguerra SS, Souayeh NESRINE, Mbarki C, Bouguerra B. Performance Of The RMI-4 Score In Predicting Malignancy Of Adnexal Masses In Postmenopausal Women. International Journal of Gynecological Cancer. 2025;35(2). |
| 1. Braicu EI, Torsten U, Mecke H, Richter R, Ames K, Hellmeyer L, et al. Role of HE4, CA125, and ultrasound in risk assessment in pelvic masspatients: Results from a prospective, multicentric study. Journal of Clinical Oncology. 2015;33(15). |
| 1. Carballo EV, Li Z, Sadowski E, Barroilhet LM. Surgical outcomes of adnexal masses classified by IOTA simple rules:Identifying opportunities to reduce surgical morbidity. Gynecologic Oncology. 2020;159:124. |
| 1. Dochez V, Randet M, Renaudeau C, Dimet J, Le Thuaut A, Caillon H, et al. Diagnostic performances of HE4, CA125, RMI and ROMA for the detection ofovarian cancer in presumed benign ovarian tumours. BJOG Int J Obstet Gynaecol. 2017;124:77. |
| 1. Filiz AA, Atalay CR. Comparison of O-RADS and IOTA ADNEX model criteria with pathology results in adnexial masses. International Journal of Gynecological Cancer. 2022;32:A291 |
| 1. Froyman W, Landolfo C, Bourne T, Cock BD, Testa A, Valentin L, et al. Performance of the RMI and IOTA ADNEX and Simple Rules risk model in theevaluation of adnexal masses not classifiable using the Easy Descriptorsas first step. BJOG: An International Journal of Obstetrics and Gynaecology. 2016;123:83-4. |
| 1. Gueriero S, Pascual MA, Piras A, Musa E, Ajossa S, Rodriguez I, et al. Cost-effective evaluation of magnetic resonance after use of simple rulesin ovarian cancer. Australasian Journal of Ultrasound in Medicine. 2019;22(2):145. |
| 1. Jan Ž, Mörtl MG. Sonography of adnexal lesion, “ADNEX”, and ovarian cancer: clinicalutility of a predictive model. Geburtshilfe und Frauenheilkunde. 2022;82(4):e6. |
| 1. Kaijser J, Van Gorp T, Van Holsbeke C, Sayasneh A, Vergote I, Bourne T, et al. Diagnostic test performance of CA125, HE4, ROMA and IOTA's LR2 in adnexaltumours of different size. BJOG: An International Journal of Obstetrics and Gynaecology. 2013;120:360-361 |
| 1. Kaijser J, Van Gorp T, Van Holsbeke C, Sayasneh A, Vergote I, Bourne T, et al. IOTA simple descriptors (SD) or simple rules (SR) as a triage test inpatients with ovarian tumours: Subsequent value of CA125, HE4 or ROMA inclinical reality? BJOG: An International Journal of Obstetrics and Gynaecology. 2013;120:371. |
| 1. Kaijser J, Van Gorp T, Van Hoorde K, Van Holsbeke C, Bourne T, Vergote I, et al. Serum CA-125 and HE-4 versus an ultrasound based predictive model toassess risk of malignancy in women with adnexal masses. International Journal of Gynecological Cancer. 2012;22:E149-E50. |
| 1. Knez J, Gruškovnjak G, Pakiž M. Ultrasound diagnosis of ovarian tumours by pattern recognition. International Journal of Gynecological Cancer. 2019;29:A290. |
| 1. Kung RWK, Dorman G, Morgan D, Johnston K. Unexpected malignancy in laparoscopic adnexal surgery. Gynecological Surgery. 2012;9(1):S92. |
| 1. Lefringhouse J, Ueland FR, Ore RM, Headley BL, Lynch E, Robbins R, et al. Comparing 2 sonographic scoring systems for distinguishing benign from malignant ovarian tumors. Gynecologic Oncology. 2016;141:57. |
| 1. Leone Roberti Maggiore U, Chiappa V, Bogani G, Perotto S, Signorelli M, Martinelli F, et al. Subjective ultrasound assessment and the adnex model to differentiatebetween benign and malignant ovarian tumors. International Journal of Gynecological Cancer. 2017;27:972. |
| 1. Madár I, Szabó G. Evaluation of IOTA simple rules and IOTA ADNEX model in the hands ofexpert examiners at the diagnosis of the adnexal tumors. Australasian Journal of Ultrasound in Medicine. 2019;22(2):147. |
| 1. Manegold-Brauer G, Schoetzau A, Hacker N, Lapaire O, Heinzelmann- Schwarz V. Proposal of a new two-step use of the risk of malignancy index in ageneral gynecological outpatient setting as compared to a gynecologicalcancer center. International Journal of Gynecological Cancer. 2015;25(9):223. |
| 1. Martra F, Tripodi E, Modaffari P, Zanfagnin V, Fuso L, De Sanso G, et al. Ultrasound score versus experienced ultrasound examiner interpretation:Are both necessary to improve the management of ovarian masses? International Journal of Gynecological Cancer. 2011;21(12):S385. |
| 1. Mathikatti AV, Venkatesh S, Saxena R. Clinical study of adnexal mass in gynaecology patients. BJOG: An International Journal of Obstetrics and Gynaecology. 2015;122:143-4. |
| 1. Minocha S. Predictability of RMI and RI for malignancy in ovarian masses-Value in lowcost and secondary care setting. BJOG: An International Journal of Obstetrics and Gynaecology. 2018;125:178. |
| 1. Modi SS, Goyal P, Desai D, Verma R. 57P International ovarian tumor analysis (IOTA) simple ultrasound rules and risk of malignancy index in differentiating benign from malignant ovarian masses. ESMO Open. 2023;8(1). |
| 1. Moro F, Ciancia M, Di Berardino S, Baldassari G, Tran HE, Boldrini L, et al. Developing And Validating Ultrasound-Based Machine Learning Models Incorporating Radiomics Features To Predict Risk Of Malignancy And Lymph Node Dissemination In Patients With Adnexal Masses. International Journal of Gynecological Cancer. 2025;35(2). |
| 1. Moszynski R, Szpurek D, Szubert S, Michalak S, Krygowska J, Sajdak S. Prognostic value of adnexal masses subjective ultrasonography assessmentin qualification for laparoscopy. Gynecological Surgery. 2011;8:S141-S2. |
| 1. Nikolova T, Zivadinovic R, Nikolova N, Klisarovska V, Evtimovska N, Stanojevic M. Comparison of diagnostic performances of HE4, risk of malignancy algorithmand morphology index in discrimination of ovarian endometriosis fromepithelial ovarian cancer in premenopausal women. International Journal of Gynecological Cancer. 2017;27:515. |
| 1. Rodrigues A, Castro MG, Negrão L, Matias S, Águas F. International ovarian tumor analysis simple rules: The essential startingpoint for optimal management. Australasian Journal of Ultrasound in Medicine. 2019;22(2):149. |
| 1. Rodríguez N, Pérez S, Rodríguez JN, Cantor N, Ardila F, Esquivel AL, et al. Ovarian-Adnexal Reporting & Data System (O-RADS) versus modified benign descriptors and IOTA ADNEX (two-step strategy) in classification of ovarian masses. Journal of Clinical Oncology. 2024;42(16). |
| 1. Sayasneh A, Preisler J, Stlader C, Husicka R, Naji O, Kaijser J, et al. A randomised controlled trial to compare the clinical impact of RMI versusLR2 to characterise adnexal masses: interim analysis of phase 4 IOTA study. BJOG. 2013;120:357‐8. |
| 1. Sebajuri JMV, Small M, Magriples U, Rukundo JD, Ntasumbumuyange D, Rulisa S, et al. Obgyn resident use of IOTA ultrasound guidelines for benign vs malignant ovarian tumors in Rwanda. International Journal of Gynecology and Obstetrics. 2018;143:316. |
| 1. Shimada K, Mimura T, Ishikawa T, Ichizuka K, Sekizawa A. The usefulness of ultrasonographic evaluation of malignant ovarian tumorsby iota (international ovarian tumor analysis) study. Gynecological Surgery. 2015;12(1):S363 |
| 1. Sole-Sedeno J, Agramunt S, Mancebo G, Rueda C, Sastre M, Alameda F, et al. Risk malignancy index in the evaluation of the adnexal masses. International Journal of Gynecological Cancer. 2012;22:E967-E8. |
| 1. Strolyte D, Celkiene I, Paskauskas S, Vaitkiene D, Gaurilcikas A. Evaluation of a transvaginal ultrasound report quality in cases ofborderline ovarian tumors: A retrospective study. International Journal of Gynecological Cancer. 2015;25(9):1351. |
| 1. Sundar S, Agarwal R, Scandrett K, Davenport C, Mallett S, Sengupta P, et al. PO008/#619  A multicentre, prospective cohort study investigating diagnostic accuracy in women with symptoms of suspected ovarian cancer (The Rockets Study): results for pre-menopausal women. International Journal of Gynecological Cancer. 2024;34:A20. |
| 1. Sundar S, Agarwal R, Deeks J, Mallett S, Scandrett K, Davenport C, et al. Refining Ovarian Cancer Test accuracy Scores: A multicentre, prospective cohort study investigating diagnostic accuracy among women with symptoms of suspected ovarian cancer (the ROCkeTS study): Results for post-menopausal women. International Journal of Gynecological Cancer. 2024;34:A37-A8. |
| 1. Thompson R, Dempsey A, Abdel-Aty M. Which risk of malignancy index (RMI) calculation is a better predictor ofmalignancy, and at what level should we refer to the cancer centre? Aretrospective observational study conducted at East Lancashire HospitalsNHS Trust. BJOG: An International Journal of Obstetrics and Gynaecology. 2014;121:9. |
| 1. Toledo KL, Audifred JR, Topete RE, Niebla DC, Hernandez SE, Morales L. Comparison between histopathological results and malignancy index risk inadnexal complex cysts treated by laparoscopic surgery. Journal of Minimally Invasive Gynecology. 2016;23(7):S217-S8. |
| 1. Vaes E, Manchanda R, Nir R, Nir D, Bleiberg H, Robert A, et al. A sequential use of the Risk of Malignancy Index and Ovarian HistoScanningfor the differential diagnosis of adnexal masses. European Journal of Cancer, Supplement. 2009;7(2):147. |
| 1. Velayo C, Reforma K, Sicam RV, Diwa M, Sy AD. Determining a reliable strategy for the prediction of ovarian cancer:serial versus parallel testing with a multivariate index assay. Gynecologic oncology. 2021;162:S127‐S8. |
| 1. Velayo C, Reforma K, Sicam R, Hernandez-Diwa M, Sy A. Prediction of ovarian cancer using a multivariate assay: A randomizedcontrolled trial to improve diagnostic strategies in Filipino women (preliminary results of the overa study). International Journal of Gynecological Cancer. 2020;30:A70-A1. |
| 1. Weinberger V, Minar L. Diagnostics of malign ovarian tumors by ultrosound and CA 125-ourexperience. International Journal of Gynecological Cancer. 2013;23(8):498. |
| 1. Yadav SK, D'Angelo A, Abdurazaq B, Amso NN. Morphological changes in conservatively managed benign ovarian cystcharacterised by simple ultrasonography rules in asymptomaticpostmenopausal women: A retrospective cohort study based on the UnitedKingdom Collaborative Trial of Ovarian Cancer Screening. BJOG: An International Journal of Obstetrics and Gynaecology. 2014;121:5. |
| **Exclusion reason: Not a diagnostic accuracy study (n = 14)** |
| 1. Faschingbauer F, Benz M, Häberle L, Goecke TW, Beckmann MW, Renner S, et al. Subjective assessment of ovarian masses using pattern recognition: The impact of experience on diagnostic performance and interobservervariability. Archives of Gynecology and Obstetrics. 2012;285(6):1663-9. |
| 1. Ahmed ASAE, Alrahman HAMA, Esmaiyl AAE, Basha MAA. The Ovarian-Adnexal Reporting and Data System (O-RADS) and Adnexal Masses. Journal of Pharmaceutical Negative Results. 2023;14:597-609. |
| 1. Levine D. O-RADS US: A Retrospective Assessment of Prediction of Malignancy in a High-Risk Setting. Radiology. 2022;304(1):121-2. |
| 1. Lu C, Suykens JAK, Timmerman D, Vergote I, Huffel Sv. Linear and nonlinear preoperative classification of ovarian tumors Ichimura T, Yoshida K, editors. Knowledge based intelligent systems for health care International Series on Advanced Intelligence 7; Advanced Knowledge International;. 2004:343-82. |
| 1. Meys E, Rutten I, Kruitwagen R, Slangen B, Lambrechts S, Mertens H, et al. Simple Rules, Not So Simple: The Use of International Ovarian TumorAnalysis (IOTA) Terminology and Simple Rules in Inexperienced Hands in aProspective Multicenter Cohort Study. Simple Rules" - nicht so einfach: Anwendung der International OvarianTumor Analysis" (IOTA)- Terminologie und der Simple Rules" in unerfahrenenHanden in einer prospektiven multizentrischen Kohortenstudie. 2017;38(6):633-41. |
| 1. Miller RW, Van Nagell JR. Preoperative evaluation of adnexal masses. Women's Health. 2011;7(1):37-9. |
| 1. Moisidis-Tesch CM, Vedder N, Ginsberg NA. Rapid rate of growth in adnexal masses, despite benign appearance onultrasound, was associated with malignancy. A retrospective analysis of 48consecutive cases from a single institution. Clinical and Experimental Obstetrics and Gynecology. 2021;48(5):1081-8. |
| 1. Nunes N, Ambler G, Foo X, Naftalin J, Derdelis G, Widschwendter M, et al. Comparison of two protocols for the management of asymptomaticpostmenopausal women with adnexal tumours - a randomised controlled trialof RMI/RCOG vs Simple Rules. British journal of cancer. 2017;116(5):584-91. |
| 1. Rizzuto I, Rezai S, Lane A, Prior M, Robledo KP, Obermair A. Subjective Assessment of Adnexal Masses Using Various Ultrasonographic Diagnostic Models: An Analysis of Interobserver Variability. Australian and New Zealand Journal of Obstetrics and Gynaecology. 2025 |
| 1. Sebajuri JMV, Magriples U, Small M, Ntasumbumuyange D, Rulisa S, Bazzett-Matabele L. Obstetrics and Gynecology Residents Can Accurately Classify Benign OvarianTumors Using the International Ovarian Tumor Analysis Rules. Journal of ultrasound in medicine : official journal of the American Institute of Ultrasound in Medicine. 2020;39(7):1389-93. |
| 1. Terzic M, Dotlic J, Likic I, Ladjevic N, Brndusic N, Mihailovic T, et al. Predictive factors of malignancy in patients with adnexal masses. European journal of gynaecological oncology. 2013;34(1):65-9. |
| 1. Van Calster B, Timmerman D, Valentin L, McIndoe A, Ghaem-Maghami S, Testa AC, et al. Triaging women with ovarian masses for surgery: Observational diagnosticstudy to compare RCOG guidelines with an international ovarian tumourAnalysis (IOTA) group protocol. BJOG: An International Journal of Obstetrics and Gynaecology. 2012;119(6):662-71. |
| 1. Van Holsbeke C, Daemen A, Yazbek J, Holland TK, Bourne T, Mesens T, et al. Ultrasound experience substantially impacts on diagnostic performance and confidence when adnexal masses are classified using pattern recognition. Gynecol Obstet Invest. 2010;69(3):160-8. |
| 1. Wynants L, Timmerman D, Verbakel JY, Testa A, Savelli L, Fischerova D, et al. Clinical Utility of Risk Models to Refer Patients with Adnexal Masses toSpecialized Oncology Care: Multicenter External Validation Using DecisionCurve Analysis. Clinical cancer research : an official journal of the American Associationfor Cancer Research. 2017;23(17):5082-90. |
| **Exclusion reason: Retrospective data collection (n = 65)**  *Studies in which ultrasound model variables were not collected prospectively at the time of ultrasound examination and with retrospective application of the models, such as to stored images or videos* |
| 1. Abdalla N, Bachanek M, Trojanowski S, Cendrowski K, Sawicki W. Diagnostic value of ultrasound indicators of neoplastic risk inpreoperative differentiation of adnexal masses. Journal of ultrasonography. 2013;13(53):145-54. |
| 1. Akdeniz N, Kuyumcuoǧlu U, Kale A, Erdemoǧlu M, Caca F. Risk of malignancy index for adnexal masses. European Journal of Gynaecological Oncology. 2009;30(2):178-80. |
| 1. Al-Musalhi K, Al-Kindi M, Ramadhan F, Al-Rawahi T, Al-Hatali K, Mula-Abed WA. Validity of cancer antigen-125 (CA-125) and risk of malignancy index (RMI)in the diagnosis of ovarian cancer. Oman Medical Journal. 2015;30(6):428-34. |
| 1. Aziz AB, Najmi N. Is Risk Malignancy Index a Useful Tool for Predicting Malignant OvarianMasses in Developing Countries? Obstetrics and gynecology international. 2015;2015:951256. |
| 1. Bailey J, Tailor A, Naik R, Lopes A, Godfrey K, Hatem HM, et al. Risk of malignancy index for referral of ovarian cancer cases to atertiary center: Does it identify the correct cases? International Journal of Gynecological Cancer. 2006;16:30-4. |
| 1. Basha MAA, Metwally MI, Gamil SA, Khater HM, Aly SA, El Sammak AA, et al. Comparison of O-RADS, GI-RADS, and IOTA simple rules regarding malignancyrate, validity, and reliability for diagnosis of adnexal masses. European radiology. 2021;31(2):674-84. |
| 1. Boll D. The pre-operative assessment of the adnexal mass: the accuracy of clinical estimates versus clinical prediction rules. BJOG: An International Journal of Obstetrics and Gynaecology. 2003;110(5):519-23. |
| 1. Campos A, Villermain-Lecolier C, Sadowski EA, Bazot M, Touboul C, Razakamanantsoa L, et al. O-RADS scoring system for adnexal lesions: Diagnostic performance on TVUS performed by an expert sonographer and MRI. European journal of radiology. 2023;169:111172. |
| 1. Chen G-Y, Hsu T-F, Chan IS, Liu C-H, Chao W-T, Shih Y-C, et al. Comparison of the O-RADS and ADNEX models regarding malignancy rate andvalidity in evaluating adnexal lesions. European radiology. 2022;32(11):7854-64. |
| 1. Chopra S, Vaishya R, Kaur J. An Evaluation of the Applicability of the Risk of Malignancy Index forAdnexal Masses to Patients Seen at a Tertiary Hospital in Chandigarh,India. Journal of obstetrics and gynaecology of India. 2015;65(6):405-10. |
| 1. Clarke SE, Grimshaw R, Rittenberg P, Kieser K, Bentley J. Risk of malignancy index in the evaluation of patients with adnexal masses. J Obstet Gynaecol Can. 2009;31(5):440-5. |
| 1. Dai W-L, Wu Y-N, Ling Y-T, Zhao J, Zhang S, Gu Z-W, et al. Development and validation of a deep learning pipeline to diagnose ovarian masses using ultrasound screening: a retrospective multicenter study. eClinicalMedicine. 2024;78. |
| 1. De Vitis LA, Schivardi G, Grcevich L, Capasso I, Fumagalli D, Dahal S, et al. Diagnostic Algorithms for Adnexal Masses in the Hands of a Novice Operator. Obstetrics & Gynecology. 2025;145(4). |
| 1. Enakpene CA, Omigbodun AO, Goecke TW, Odukogbe A-T, Beckmann MW. Preoperative evaluation and triage of women with suspicious adnexal massesusing risk of malignancy index. The journal of obstetrics and gynaecology research. 2009;35(1):131-8. |
| 1. Esquivel Villabona AL, Rodríguez JN, Ayala N, Buriticá C, Gómez AC, Velandia AM, et al. Two-Step Strategy for Optimizing the Preoperative Classification ofAdnexal Masses in a University Hospital, Using International Ovarian TumorAnalysis Models: Simple Rules and Assessment of Different NEoplasias inthe adneXa Model. Journal of ultrasound in medicine : official journal of the AmericanInstitute of Ultrasound in Medicine. 2022;41(2):471-82. |
| 1. Giourga M, Pouliakis A, Vlastarakos P, Stavrou S, Tsiriva M, Gerede A, et al. Evaluation of IOTA-ADNEX Model and Simple Rules for Identifying Adnexal Masses by Operators with Varying Levels of Expertise: A Single-Center Diagnostic Accuracy Study. Ultrasound International Open. 2022;9(1):E11-E7. |
| 1. Guo Y, Zhao B, Zhou S, Wen L, Liu J, Fu Y, et al. A comparison of the diagnostic performance of the O-RADS, RMI4, IOTA LR2,and IOTA SR systems by senior and junior doctors. Ultrasonography. 2022;41(3):511-8. |
| 1. Hack K, Gandhi N, Bouchard-Fortier G, Chawla TP, Ferguson SE, Li S, et al. External Validation of O-RADS US Risk Stratification and Management System. Radiology. 2022;304(1):114-20. |
| 1. Hada A, Han LP, Chen Y, Hu QH, Yuan Y, Liu L. Comparison of the predictive performance of risk of malignancy indexes1-4, HE4 and risk of malignancy algorithm in the triage of adnexal masses. Journal of Ovarian Research. 2020;13(1). |
| 1. Harry VN, Narayansingh GV, Parkin DE. The risk of malignancy index for ovarian tumours in Northeast Scotland — a population based study. Scottish medical journal. 2009;54(2):21-3. |
| 1. Iatrakis GM, Zervoudis S, Tsikouras P, Iatrakis DG, Bothou A, Chardavelas T, et al. A new risk malignancy index to predict ovarian cancer: A bicentricpreliminary study. Journal of BUON. 2018;23(5):1380-3. |
| 1. Jha S, Singh A. Enhancing Diagnostic Accuracy in Ovarian Tumour Assessment: A Combined Approach of IOTA Simple Rules and CA125. Eurasian Journal of Medicine and Oncology. 2023;7(4):312-7. |
| 1. Kader Ali Mohan GR, Jaaback K, Proietto A, Robertson R, Angstetra D. Risk Malignancy Index (RMI) in patients with abnormal pelvic mass:Comparing RMI 1, 2 and 3 in an Australian population. The Australian & New Zealand journal of obstetrics & gynaecology. 2010;50(1):77-80. |
| 1. Kadooka M, Suemitsu T, Ashimoto K, Takesawa A, Matsui H, Otsuka I, et al. Validation of the IOTA ADNEX Model Among Japanese Women Performed by Gynecology Trainees and Ultrasound Specialists: A Retrospective Diagnostic Accuracy Study. Journal of ultrasound in medicine : official journal of the American Institute of Ultrasound in Medicine. 2024. |
| 1. Karimi-Zarchi M, Mojaver SP, Rouhi M, Hekmatimoghaddam SH, Moghaddam RN, Yazdian-Anari P, et al. Diagnostic Value of the Risk of Malignancy Index (RMI) for Detection ofPelvic Malignancies Compared with Pathology. Electronic physician. 2015;7(7):1505-10. |
| 1. Karlsen MA, Hogdall EVS, Christensen IJ, Borgfeldt C, Kalapotharakos G, Zdrazilova-Dubska L, et al. A novel diagnostic index combining HE4, CA125 and age may improve triageof women with suspected ovarian cancer - An international multicenterstudy in women with an ovarian mass. Gynecologic oncology. 2015;138(3):640-6. |
| 1. Lai H-W, Lyu G-R, Kang Z, Li L-Y, Zhang Y, Huang Y-J. Comparison of O-RADS, GI-RADS, and ADNEX for Diagnosis of Adnexal Masses:An External Validation Study Conducted by Junior Sonologists. Journal of ultrasound in medicine : official journal of the AmericanInstitute of Ultrasound in Medicine. 2022;41(6):1497-507. |
| 1. Ma S, Shen K, Lang J. A risk of malignancy index in preoperative diagnosis of ovarian cancer. Chinese medical journal. 2003;116(3):396-9. |
| 1. Mahale N, Kumar N, Mahale A, Ullal S, Fernandes M, Prabhu S. Validity of ultrasound with color Doppler to differentiate between benign and malignant ovarian tumours. Obstetrics & gynecology science. 2024;67(2):227-34. |
| 1. Manegold-Brauer G, Buechel J, Knipprath-Mészaros A, Schoetzau A, Hacker NF, Tercanli S, et al. Improved Detection Rate of Ovarian Cancer Using a 2-Step Triage Model ofthe Risk of Malignancy Index and Expert Sonography in an OutpatientScreening Setting. International Journal of Gynecological Cancer. 2016;26(6):1062-9. |
| 1. Manjunath AP, Pratapkumar, Sujatha K, Vani R. Comparison of three risk of malignancy indices in evaluation of pelvicmasses. Gynecologic oncology. 2001;81(2):225-9. |
| 1. Melo A, Verissimo R, Farinha M, Martins NN, Martins FN. Discriminative value of CA-125, HE4, Risk of Malignancy Index II (RMI-II)and Risk of Malignancy Algorithm (ROMA) in the differential diagnosis ofpelvic masses: conclusions from a referral Centre in Portugal. Journal of obstetrics and gynaecology : the journal of the Institute of Obstetrics and Gynaecology. 2018;38(8):1140-5. |
| 1. Moolthiya W, Yuenyao P. The risk of malignancy index (RMI) in diagnosis of ovarian malignancy. Asian Pacific journal of cancer prevention : APJCP. 2009;10(5):865-8. |
| 1. Moore RG, Jabre-Raughley M, Brown AK, Robison KM, Miller MC, Allard WJ, et al. Comparison of a novel multiple marker assay vs the Risk of MalignancyIndex for the prediction of epithelial ovarian cancer in patients with apelvic mass. American journal of obstetrics and gynecology. 2010;203(3):228.e1-6. |
| 1. Morgante G, La Marca A, Ditto A, De Leo V. Comparison of two malignancy risk indices based on serum CA125, ultrasoundscore and menopausal status in the diagnosis of ovarian masses. British Journal of Obstetrics and Gynaecology. 1999;106(6):524-7. |
| 1. Nam G, Lee SR, Jeong K, Kim SH, Moon H-S, Chae HD. Assessment of different NEoplasias in the adneXa model for differentiationof benign and malignant adnexal masses in Korean women. Obstetrics & gynecology science. 2021;64(3):293-9. |
| 1. Ning C-P, Ji X, Wang H-Q, Du X-Y, Niu H-T, Fang S-B. Association between the sonographer's experience and diagnosticperformance of IOTA simple rules. World journal of surgical oncology. 2018;16(1):179. |
| 1. Nohuz E, De Simone L, Chêne G. Reliability of IOTA score and ADNEX model in the screening of ovarianmalignancy in postmenopausal women. Journal of Gynecology Obstetrics and Human Reproduction. 2019;48(2):103-7. |
| 1. Nowak A, Soja M, Masternak M, Mokros Ł, Wilczyński J, Szubert M. Evaluation of adnexal tumours in the International Ovarian Tumor Analysissystem in reference to histopathological results. Przeglad Menopauzalny. 2019;18(3):141-5. |
| 1. Ong C, Biswas A, Choolani M, Low JJH. Comparison of risk of malignancy indices in evaluating ovarian masses in asoutheast asian population. Singapore Medical Journal. 2013;54(3):136-9. |
| 1. Oranratanaphan S, Wanishpongpan S, Termrungruanglert W, Triratanachat S. Assessment of Diagnostic Values among CA-125, RMI, HE4, and ROMA forCancer Prediction in Women with Nonfunctional Ovarian Cysts. Obstetrics and gynecology international. 2018;2018:7821574. |
| 1. Patel-Lippmann KK, Sadowski EA, Robbins JB, Paroder V, Barroilhet L, Maddox E, et al. Comparison of international ovarian tumor analysis simple rules to societyof radiologists in ultrasound guidelines for detection of malignancy inadnexal cysts. American Journal of Roentgenology. 2020;214(3):694-700. |
| 1. Pelayo M, Pelayo-Delgado IA-O, Sancho-Sauco J, Sanchez-Zurdo JA-O, Abarca-Martinez L, Corraliza-Galán V, et al. Comparison of Ultrasound Scores in Differentiating between Benign and Malignant Adnexal Masses. LID - 10.3390/diagnostics13071307 [doi] LID - 1307. (2075-4418 (Print)). |
| 1. Phinyo P, Patumanond J, Saenrungmuaeng P, Chirdchim W, Pipanmekaporn T, Tantraworasin A, et al. Early-Stage Ovarian Malignancy Score versus Risk of Malignancy Indices:Accuracy and Clinical Utility for Preoperative Diagnosis of Women withAdnexal Masses. Medicina (Kaunas, Lithuania). 2020;56(12). |
| 1. Prys Davies A, Jacobs I, Woolas R, Fish A, Oram D. The adnexal mass: Benign of malignant? Evaluation of a risk of malignancyindex. British Journal of Obstetrics and Gynaecology. 1993;100(10):927-31. |
| 1. Qiu L, Yang F, Luo H. A preliminary study: The sequential use of the risk malignancy index andcontrast-enhanced ultrasonography in differential diagnosis of adnexalmasses. Medicine. 2018;97(29):e11536. |
| 1. Rossi A, Forzano L, Romanello I, Ambrosini G, Iuri V, Marchesoni D. Comparison of Pelvic Masses Score (PMS) and Risk of Malignancy Index (RMI3) in the evaluation of pelvic masses. European Journal of Gynaecological Oncology. 2014;35(4):421-4. |
| 1. Simsek HS, Tokmak A, Ozgu E, Doganay M, Danisman N, Erkaya S, et al. Role of a risk of malignancy index in clinical approaches to adnexalmasses. Asian Pacific journal of cancer prevention : APJCP. 2014;15(18):7793-7. |
| 1. Stephens AN, Hobbs SJ, Kang SW, Oehler MK, Jobling TW, Allman R. Utility of a Multi-Marker Panel with Ultrasound for Enhanced Classification of Adnexal Mass. Cancers. 2024;16(11). |
| 1. Stiekema A, Lok CAR, Kenter GG, van Driel WJ, Vincent AD, Korse CM. A predictive model combining human epididymal protein 4 and radiologicfeatures for the diagnosis of ovarian cancer. Gynecologic oncology. 2014;132(3):573-7. |
| 1. Sukanya L. Risk of malignancy index (RMI) for prediction of malignancy in women withadnexal masses. International Journal of Research in Pharmaceutical Sciences. 2022;13(3):339-42. |
| 1. Tavoraite I, Kronlachner L, Opolskiene G, Bartkeviciene D. Ultrasound Assessment of Adnexal Pathology: Standardized Methods andDifferent Levels of Experience. Medicina (Kaunas, Lithuania). 2021;57(7). |
| 1. Tian C, Wen S-B, Zhao C-Y, Yan X-N, Du J-X. Comparative diagnostic accuracy of the IOTA SRR and LR2 scoring systems for discriminating between malignant and Benign Adnexal masses by junior physicians in Chinese patients: a retrospective observational study. BMC women's health. 2023;23(1):585. |
| 1. Tug N, Yassa M, Sargin MA, Taymur BD, Sandal K, Meg E. Preoperative discriminating performance of the IOTA-ADNEX model andcomparison with risk of malignancy index: An external validation in anon-gynecologic oncology tertiary center. European Journal of Gynaecological Oncology. 2020;41(2):200-7. |
| 1. Vaes E, Manchanda R, Nir R, Nir D, Bleiberg H, Autier P, et al. Mathematical models to discriminate between benign and malignant adnexalmasses: Potential diagnostic improvement using ovarian histoscanning. International Journal of Gynecological Cancer. 2011;21(1):35-43. |
| 1. Varsha K, Uma T, Pallavi LP, Hari K, Kolli NS. Accuracy of CA 125, USG, CECT, RMI 1 score in diagnosis of ovarian tumour. International Journal of Academic Medicine and Pharmacy. 2024;6(3):284-8. |
| 1. Viora E, Piovano E, Baima Poma C, Cotrino I, Castiglione A, Cavallero C, et al. The ADNEX model to triage adnexal masses: An external validation study andcomparison with the IOTA two-step strategy and subjective assessment by anexperienced ultrasound operator. European Journal of Obstetrics and Gynecology and Reproductive Biology. 2020;247:207-11. |
| 1. Vural F, Aka N, Ertaş S, Köse G, Tüfekçi EC. The ovarian cancers in geriatric population: the validity of inflammatory markers, malignancy risk indices 1, 2, 3, 4, and CA-125 levels in malignancy discrimination of adnexal masses. Eur J Gynaecol Oncol. 2016;37:846-51. |
| 1. Xie W, Wang Y, Xiang Z, Du Z, Huang S, Chen Y, et al. Efficacy of IOTA simple rules, O-RADS, and CA125 to distinguish benign andmalignant adnexal masses. Journal of Ovarian Research. 2022;15(1). |
| 1. Yamamoto Y, Yamada R, Oguri H, Maeda N, Fukaya T. Comparison of four malignancy risk indices in the preoperative evaluationof patients with pelvic masses. European journal of obstetrics, gynecology, and reproductive biology. 2009;144(2):163-7. |
| 1. Yang S, Tang J, Rong Y, Wang M, Long J, Chen C, et al. Performance of the IOTA ADNEX model combined with HE4 for identifying early-stage ovarian cancer. Frontiers in Oncology. 2022;12. |
| 1. Yavuzcan A, Caglar M, Ozgu E, Ustun Y, Dilbaz S, Ozdemir I, et al. Should cut-off values of the risk of malignancy index be changed forevaluation of adnexal masses in Asian and Pacific populations? Asian Pacific journal of cancer prevention : APJCP. 2013;14(9):5455-9. |
| 1. Yoeli-Bik R, Longman RE, Wroblewski K, Weigert M, Abramowicz JS, Lengyel E. Diagnostic Performance of Ultrasonography-Based Risk Models in Differentiating Between Benign and Malignant Ovarian Tumors in a US Cohort. JAMA Network Open. 2023;6(7):e2323289-e. |
| 1. Zhong D, Gao X-Q, Li H-X, Wang H-B, Liu Y. Analysis of Diagnostic Efficacy of the International Ovarian Tumor Analysis ADNEX Model and the ACR O-RADS US (Ovarian-Adnexal Reporting and Data System) for Benign and Malignant Ovarian Tumors: A Retrospective Study in a Tumor Center in Northeast China. Journal of imaging informatics in medicine. 2024. |
| 1. Zhou S, Guo Y, Wen L, Liu J, Fu Y, Xu F, et al. Comparison of the diagnostic efficiency between the O-RADS US risk stratification system and doctors' subjective judgment. BMC medical imaging. 2023;23(1):190. |
| **Exclusion reason: unclear eligibility status (n = 33)**  *Studies for which methodological details were insufficient to assess eligibility and for which no clarification was received from the authors, or for which eligibility remained unclear after contacting the authors. In particular, if it was unclear how borderline tumors were classified (benign , malignant or excluded) and whether ultrasound model variables were collected prospectively or retrospectively* |
| 1. Adilgereyeva AS, Abdelazim IA, Zhurabekova GA, El-Ghazaly TE. Morphological parameters of ovarian masses and accuracy of the risk ofmalignancy index in diagnosing ovarian malignancy. Przeglad Menopauzalny. 2022;21(2):81-91. |
| 1. Afroz N, Basharat N, Khan S, Khan A, Jan M, Muhammad G, et al. A Study Based on the Helpful tool in the Initial Assessment of Ovarian Masses: Risk of Malignancy Index. Pakistan Journal of Medical and Health Sciences. 2022;16(12):505-7. |
| 1. Al-Asadi JN, Al-Maliki SK, Al-Dahhhan F, Al-Naama L, Suood F. The accuracy of risk malignancy index in prediction of malignancy in womenwith adnexal mass in Basrah, Iraq. Nigerian journal of clinical practice. 2018;21(10):1254-9. |
| 1. Anbumalar S, Janani S, Dheebha V, Ashraf AM, Kalaivani K. Comparison of the diagnostic accuracy of the IOTA – Simple Rules with the RMI index to distinguish between benign and malignant adnexal masses. International Journal of Academic Medicine and Pharmacy. 2023;6(1):400-4. |
| 1. Ashrafgangooei T, Rezaeezadeh M. Risk of malignancy index in preoperative evaluation of pelvic masses. Asian Pacific journal of cancer prevention : APJCP. 2011;12(7):1727-30. |
| 1. Asif N, Sattar A, Dawood MM, Rafi T, Aamir M, Anwar M. Pre-operative evaluation of ovarian mass: Risk of malignancy index. Journal of the College of Physicians and Surgeons Pakistan. 2004;14(3):128-31. |
| 1. Baral G, Joshi R, Pandit B. Diagnostic Accuracy of Risk of Malignancy Indices in Ovarian Tumor. Journal of Nepal Health Research Council. 2020;18(2):253-8. |
| 1. Behnamfar F, Esmaeilian F, Adibi A, Rouholamin S. Comparison of Ultrasound and Tumor Marker CA125 in Diagnosis of AdnexalMass Malignancies. Advanced biomedical research. 2022;11:18. |
| 1. Bouzari Z, Yazdani S, Kelagar ZS, Abbaszadeh N. Risk of malignancy index as an evaluation of preoperative pelvic mass. Caspian J Intern Med. 2011;3(4):331-5. |
| 1. Dora SK, Dandapat AB, Pande B, Hota JP. A prospective study to evaluate the risk malignancy index and itsdiagnostic implication in patients with suspected ovarian mass. Journal of ovarian research. 2017;10(1):55. |
| 1. Garg S, Kaur A, Kaur Mohi J, Sibia P, Kaur N. Evaluation of IOTA simple ultrasound rules to distinguish benign andmalignant ovarian tumours. Journal of Clinical and Diagnostic Research. 2017;11(8):TC06-TC9. |
| 1. He X, Bai X-H, Chen H, Feng W-W. Machine learning models in evaluating the malignancy risk of ovarian tumors: a comparative study. Journal of Ovarian Research. 2024;17(1):219. |
| 1. Khoiwal K, Bahadur A, Kumari R, Bhattacharya N, Rao S, Chaturvedi J. Assessment of Diagnostic Value of Serum Ca-125 and Risk of Malignancy Index Scoring in the Evaluation of Adnexal Masses. Journal of mid-life health. 2019;10(4):192-6. |
| 1. Koneczny J, Czekierdowski A, Florczak M, Poziemski P, Stachowicz N, Borowski D. The use of sonographic subjective tumor assessment, IOTA logisticregression model 1, IOTA Simple Rules and GI-RADS system in thepreoperative prediction of malignancy in women with adnexal masses. Ginekologia polska. 2017;88(12):647-53. |
| 1. Lam Huong L, Thi Phuong Dung N, Hoang Lam V, Tran Thao Nguyen N, Minh Tam L, Vu Quoc Huy N. The Optimal Cut-Off Point of the ADNEX Model for the Prediction of theOvarian Cancer Risk. Asian Pacific journal of cancer prevention (APJCP). 2022;23(8):2713-8. |
| 1. Laskar NA, Chakrabartty DK, Islam MI, Abhishek L. A Comparative Study between O-RADS and IOTA Guidelines and Assessing the Efficacy of Diffusion-Weighted MRI in Differentiation of Benign and Malignant Ovarian and Adnexal Mass Lesion. International Journal of Pharmaceutical and Clinical Research. 2024;16(8):1347-61. |
| 1. Liu B, Liao J, Gu W, Wang J, Li G, Wang L. ADNEX model-based diagnosis of ovarian cancer using MRI images. Contrast Media and Molecular Imaging. 2021 Aug 18;2021:2146578. doi: 10.1155/2021/2146578. |
| 1. Mansour GM, El-Lamie IK, El-Sayed HM, Ibrahim AM, Laban M, Abou-Louz SK, et al. Adnexal mass vascularity assessed by 3-dimensional power doppler: Does itadd to the risk of malignancy index in prediction of ovarian malignancy?Four hundred-case study. International Journal of Gynecological Cancer. 2009;19(5):867-72. |
| 1. Peng XS, Ma Y, Wang LL, Li HX, Zheng XL, Liu Y. Evaluation of the diagnostic value of the ultrasound ADNEX model forbenign and malignant ovarian tumors. International Journal of General Medicine. 2021;14:5665-73. |
| 1. Radwan AM, Taema MI. Accuracy of the risk of malignancy index-I in diagnosing ovarian malignancy in menopausal women. Przeglad Menopauzalny. 2023;22(1):1-5. |
| 1. Ramya SR. Identification of Effective Model for Prediction of Ovarian MalignancyRisk using Models like Risk of Malignancy Index, Logistic Regression,International Ovarian Tumour Analysis- Simple Rules. Journal of Clinical and Diagnostic Research. 2022;16(4):QC01-QC5. |
| 1. Rani RS, P V, Durairaj J, H N. Utility of HE4 in the Evaluation of Adnexal Masses Among Premenopausal and Postmenopausal Women. Indian Journal of Surgical Oncology. 2025. |
| 1. Rashmi N, Singh S, Begum J, Sable MN. Diagnostic Performance of Ultrasound-Based International Ovarian Tumor Analysis Simple Rules and Assessment of Different NEoplasias in the adneXa Model for Predicting Malignancy in Women with Ovarian Tumors: A Prospective Cohort Study. Women's health reports (New Rochelle, NY). 2023;4(1):202-10. |
| 1. Rodríguez Pérez A, Caruso A, Pantoja Garrido M, Rodríguez Jiménez I, Polo Velasco A, Fernández Alba JJ. Diagnostic rentability of IOTA models for differentiating between benign and malignant complex adnexal masses. Clínica e Investigación en Ginecología y Obstetricia. 2025;52(1):101000. |
| 1. Romagnolo C, Trivella G, Bonacina M, Fornalè M, Maggino T, Ferrazzi E. Preoperative diagnosis of 221 consecutive ovarian masses: Scoring systemand expert evaluation. European Journal of Gynaecological Oncology. 2006;27(5):487-9. |
| 1. Rossi A, Braghin C, Soldano F, Isola M, Capodicasa V, Londero AP, et al. A proposal for a new scoring system to evaluate pelvic masses: Pelvic Masses Score (PMS). Eur J Obstet Gynecol Reprod Biol. 2011;157(1):84-8. |
| 1. Shahzad U, Shahzad K, Manzoor U, Zafar H, Ali S, Rehman A. Correlation of Risk of Malignancy Index to Malignant Nature of Ovarian Tumours. Pakistan Journal of Medical and Health Sciences. 2022;16(11):164-5. |
| 1. Sharma M, Kumar N, Saha S, Suri V, Prasad GR, Srinivasan R, et al. Role of HE4 in evaluation of adnexal masses and its comparison with CA125, ROMA and RMI in premenopausal women. (1729-0503 (Electronic)). |
| 1. Siddu S, Bharati S, Gond A, Kumar S. Co-relation of histopathological diagnosis with conventional RMI scoring in evaluation and differentiation of benign from malignant adnexal mass. Journal of Population Therapeutics and Clinical Pharmacology. 2024;31(9):1141-9. |
| 1. Wang LM, Song H, Song X, Zhou XB. An improved risk of malignancy index in diagnosis of adnexal mass. Chinese Medical Journal. 2012;125(3):533-5. |
| 1. Zahir N, Ali S, Rukhsana. Diagnostic Accuracy of Risk of Malignancy Index RMI in Patients with Adnexal Mass. Pakistan Journal of Medical and Health Sciences. 2023;17(1):666-8. |
| 1. Zareen H, Malik MM, Nafees R, Ali SI. Evaluation of risk of malignancy indices 1, 2, and 3 in pre-operative assessment of the ovarian masses. Journal of Pharmaceutical Negative Results. 2022;13:3275-82. |
| 1. Zhang Y, Zhao Y, Feng L. External Validation of the Assessment of Different NEoplasias in theadneXa Model Performance in Evaluating the Risk of Ovarian CarcinomaBefore Surgery in China: A Tertiary Center Study. Journal of ultrasound in medicine: official journal of the AmericanInstitute of Ultrasound in Medicine. 2022;41(9):2333-42. |
| **Exclusion reason: no full-text available (n = 3)** |
| 1. 1Abudia S. Evaluation of the risk of malignancy index based on serum Ca125,ultrasound findings and menopausal status in the pre-operative diagnosisof pelvic mass. Jamahiriya Medical Journal. 2010;10(4):286-9. |
| 1. Ali M, Jabeen R, Khurshid M. Preoperative diagnosis of malignancy in ovarian masses - A comparison ofrisk of malignancy indices I and II. Medical Forum Monthly. 2010;21(11):19-23. |
| 1. Liaquat F, Abbas HY, Ali N, Waheed A, Ghafoor S, Tahir A. Diagnostic accuracy of risk of malignancy index (RMI) in discrimination ofbenign from malignant ovarian masses. Pakistan Journal of Medical and Health Sciences. 2020;14(4):1170-2. |
| **Exclusion reason: Not in English/Dutch (n = 21)**  *Full-text not available in English or Dutch* |
| 1. Boichuk OH, Hulii DY. Diagnostic peculiarities of benign ovarian tumors during pregnancy. Reproductive Endocrinology. 2021(56):38-42. |
| 1. Davidsen MB, Nielsen SP, Sele V. Differentiation of benign and malignant ovarian tumors by transluminalultrasound scanning. Ugeskrift for laeger. 1994;156(46):6861-4. |
| 1. Egunova MA, Kutsenko IG. Comparative characteristics of the available laboratory tests and theircombinations used in differential diagnosis of ovarian neoplasms. Obstetrics, Gynecology and Reproduction. 2017;11(4):5-13. |
| 1. Famada AV, Pérez SP, Seguer JJ, Juanos JL, Pueyo JC. Validation of IOTA simple ultrasound rules in clinical practice with tumormarkers and pathology. Revista Peruana de Ginecologia y Obstetricia. 2020;66(3):19-24. |
| 1. Fathallah K, Huchon C, Bats AS, Metzger U, Lefrre-Belda MA, Bensaid C, et al. External validation of simple ultrasound rules of Timmerman on 122 ovariantumors. Gynecologie Obstetrique et Fertilite. 2011;39(9):477-81. |
| 1. Gasparov AS, Zhordania, Paianidi IG, Dubinskaia ED. [Oncogynecological aspects of adnexal masses]. Vestnik Rossiiskoi akademii meditsinskikh nauk. 2013(8):9-13. |
| 1. González-Burgos OM, Álvarez-Licona NE, Lever-Rosas CD. Comparison of three ultrasound index in evaluating the risk of malignancyof adnexal tumors. Ginecologia y Obstetricia de Mexico. 2018;86(8):519-29. |
| 1. Hagen B, Tingulstad S, Onsrud M, Moen M, Kiserud T, Eik-Nes S, et al. Preoperative identification of malignancy among women with a pelvic mass.Evaluation of a risk index based on ultrasound findings. CA 125 in serumand menopausal status. Tidsskrift for den Norske laegeforening. 1995;115(7):820-2. |
| 1. He P, Wu Q, Sun L, Wang J, Wang L, Han J, et al. Comparison of ADNEX model, simple rules risk model and risk of malignancyindex in diagnosis of benign and malignant ovarian tumors. Chinese Journal of Medical Imaging Technology. 2019;35(1):104-7. |
| 1. Krascsenits G, Balazs B, Dudnyikova A, Purcsi K, Orosz E, Pete I. [Investigating the predictive value of RMI and ROMA indices in patientswith ovarian tumors of uncertain dignity]. Az RMI- es a ROMA-index petefeszekrakelorejelzo hatekonysaganak vizsgalataadnextumorokban. 2016;60(4):320-7. |
| 1. Liu J, Chen Q, Lyu G. Comparison of ultrasound IOTA simple rules and GI-RADS ultrasonographicstratification in diagnosis of ovarian neoplasms. Chinese Journal of Medical Imaging Technology. 2017;33(5):739-42. |
| 1. Lou H-Y, Meng H, Zhu Q-L, Zhang Q, Jiang Y-X. [Application values of four risk of malignancy indices in the preoperativeevaluation of patients with adnexal masses]. Zhongguo yi xue ke xue yuan xue bao Acta Academiae Medicinae Sinicae. 2010;32(3):297-302. |
| 1. Ma S, Shen K, Lang J. Effect of a risk of malignancy index in preoperative diagnosis of ovariancancer. Zhonghua fu chan ke za zhi. 2001;36(3):162-4. |
| 1. Mekni K, Baba M, Haddad I, Aaraar M, Mejri O, ElFekih C. Applicability of the Adnex score in predicting the malignancy of ovarian cysts. Gynecologie Obstetrique Fertilite et Senologie. 2024;52(6):398-402. |
| 1. Sandal K, Polat M, Yassa M, Günay T, Erdem GY, Güzin K. Comparision of "risk of malignancy indices" and "assesment of differentneoplasia in the adnexa" (ADNEX) model as preoperative malignancyevaluation methods for adnexal masses. Zeynep Kamil Tip Bulteni. 2018;49(4):324-9. |
| 1. Smoleń A, Stachowicz N, Czekierowski A, Kotarski J. The estimation of the probability of tumor malignacy on the basis of testcombination in the primary diagnosis of adnexal tumors. Ginekologia polska. 2010;81(4):254-61. |
| 1. Tanriverdi HA, Sade H, Akbulut V, Barut A, Bayar Ü. Clinical and ultrasonographic evaluation of pelvic masses. Journal of the Turkish German Gynecology Association. 2007;8(1):67-70. |
| 1. Tehranian A, Nezamabadi AG, Yarmohammadi N, Ganjeh M, Maajaani K, Aghajani R. Evaluation of diagnostic accuracy of RMI and ROMA indices in comparison toHE4 and CA125 parameters for estimating the risk of malignancy of adnexalmasses. Tehran University Medical Journal. 2021;79(3):193-200. |
| 1. Trevino-Baez JD, Cantu-Cruz JA, Medina-Mercado J, Abundis A. [Diagnostic accuracy of malignancy risk index II in post-menopausal womenwith adnexal tumours]. Exactitud diagnostica del indice de riesgo de malignidad II en mujeresposmenopausicas con tumor anexial. 2016;84(2):109-14. |
| 1. Yang W, Lyu G, Chen Q. Ovarian-adnexal reporting and data system, gynecologic imaging reportingand data system and simple rules risk model for differentiating benign andmalignant ovarian tumors. Chinese Journal of Medical Imaging Technology. 2021;37(9):1368-72. |
| 1. Zhao B, Fu Y, Wen L, Wang Z, Fu C, Liu M. Comparison of diagnostic efficiency between IOTA LR2 model and doctors'experiences. IOTA LR2 2022;47(8):1082-8. |
| **Exclusion reason: unable to create 2x2 table (n = 21)** |
| 1. Abdulrahman GO, Jr., McKnight L, Lutchman Singh K. The risk of malignancy index (RMI) in women with adnexal masses in Wales. Taiwanese journal of obstetrics & gynecology. 2014;53(3):376-81. |
| 1. Barcroft JF, Linton-Reid K, Landolfo C, Al Memar M, Parker N, Kyriacou C, et al. The Use of Machine Learning Models and Radiomics for Segmentation and Classification of Adnexal Masses on Ultrasound: A multi-cohort retrospective study. K. Linton-Reid, Imperial College London, United Kingdom, 2023. |
| 1. Bouzari Z, Rahimi H, Gholinia H, Yazdani S, Hajian-Tilaki K, Soleimani MJ. Cancer antigen 125 (CA125), human epididymis protein 4 (HE4), risk ofmalignancy index (RMI), and risk of ovarian malignancy algorithm (ROMA) asdiagnostic tests in ovarian cancer. International Journal of Cancer Management. 2019;12(1). |
| 1. Dotlić J, Terzić M, Likić I, Atanacković J, Ladjević N. Evaluation of adnexal masses: Correlation between clinical, ultrasound andhistopathological findings. Vojnosanitetski Pregled. 2011;68(10):861-6. |
| 1. Engelen MJA, Bongaerts AHH, Sluiter WJ, de Haan HH, Bogchelman DH, Tenvergert EM, et al. Distinguishing benign and malignant pelvic masses: the value of differentdiagnostic methods in everyday clinical practice. European journal of obstetrics, gynecology, and reproductive biology. 2008;136(1):94-101. |
| 1. Grover S, Patra S, Grover H, Mittal P, Khanna G. Prospective revalidation of IOTA 'two-step', 'alternative two-step' and'three-step' strategies for characterization of adnexal masses - An Indianstudy focussing the radiology context. Indian Journal of Radiology and Imaging. 2020;30(3):304-18. |
| 1. Hakansson F, Hogdall EVS, Nedergaard L, Lundvall L, Engelholm SA, Pedersen AT, et al. Risk of malignancy index used as a diagnostic tool in a tertiary centrefor patients with a pelvic mass. Acta obstetricia et gynecologica Scandinavica. 2012;91(4):496-502. |
| 1. Hu Y, Chen B, Dong H, Sheng B, Xiao Z, Li J, et al. Comparison of ultrasound−based ADNEX model with magnetic resonance imaging for discriminating adnexal masses: a multi-center study. Frontiers in Oncology. 2023;13. |
| 1. Kansal N, Sultan S, Badkur P. Evaluation of Iota Adnex Model to Distinguish Benign and Malignant Ovarian Tumor. International Journal of Life Sciences Biotechnology and Pharma Research. 2024;13(4):320-9. |
| 1. Liu C, Li Y, Zhu Y, Lu M. The Value of IOTA Simple Rules Combined With CEUS Scoring System in theDiagnosis of Benign and Malignant Ovarian Masses and Its Correlation WithMVD and VEGF: A Preliminary Study. Journal of ultrasound in medicine : official journal of the AmericanInstitute of Ultrasound in Medicine. 2022;41(12):2983-92. |
| 1. Lu SJ, Tian YQ, He JX, Meng FL. The Predictive Value of the Combination of Copenhagen Index andSonographic Morphology Scores in the Detection of Ovarian Cancer in Womenwith Adnexal Masses. SN Comprehensive Clinical Medicine. 2020;2(3):265-71. |
| 1. McKendry K, Duff S, Huang Y, Redha M, Scanlon Á, Abu Saadeh F, et al. The value of human epididymis 4, D-dimer, and fibrinogen compared with CA125 alone in triaging women presenting with pelvic masses: a retrospectivecohort study. Acta Obstetricia et Gynecologica Scandinavica. 2021;100(7):1239-47. |
| 1. Mol BW, Boll D, De Kanter M, Heintz AP, Sijmons EA, Oei SG, et al. Distinguishing the benign and malignant adnexal mass: an externalvalidation of prognostic models. Gynecologic oncology. 2001;80(2):162-7. |
| 1. Moszynski R, Zywica P, Wojtowicz A, Szubert S, Sajdak S, Stachowiak A, et al. Menopausal status strongly influences the utility of predictive models indifferential diagnosis of ovarian tumors: an external validation ofselected diagnostic tools. Ginekologia polska. 2014;85(12):892-9. |
| 1. Nirupa S, Pavithra ND. Adnexal Masses: Evaluation And Comparison Of All The Risk Of MalignancyIndices. Research Journal of Pharmaceutical, Biological and Chemical Sciences. 2021;12(2):7-10. |
| 1. Radosa MP, Camara O, Vorwergk J, Diebolder H, Winzer H, Mothes A, et al. Preoperative multimodal strategies for risk assessment of adnexal masses: analysis of 1362 cases in a gynecologic cancer center. Int J Gynecol Cancer. 2011;21(6):1056-62. |
| 1. Raza A, Mould T, Wilson M, Burnell M, Bernhardt L. Increasing the effectiveness of referral of ovarian masses from cancerunit to cancer center by using a higher referral value of the risk ofmalignancy index. International journal of gynecological cancer : official journal of theInternational Gynecological Cancer Society. 2010;20(4):552-4. |
| 1. Sinha A, Drews F, Lim K, Pugh ND. Retrospective analysis of suspicious pelvic masses using the Pelvic MassIndex (PMI) scoring system from 2007 to 2014. European journal of obstetrics, gynecology, and reproductive biology. 2016;201:79-84. |
| 1. Szubert S, Szpurek D, Wójtowicz A, Żywica P, Stukan M, Sajdak S, et al. Performance of Selected Models for Predicting Malignancy in Ovarian Tumorsin Relation to the Degree of Diagnostic Uncertainty by SubjectiveAssessment With Ultrasound. Journal of ultrasound in medicine : official journal of the AmericanInstitute of Ultrasound in Medicine. 2020;39(5):939-47. |
| 1. Terzic MM, Dotlic J, Likic I, Ladjevic N, Brndusic N, Arsenovic N, et al. Current diagnostic approach to patients with adnexal masses: which toolsare relevant in routine praxis? Chinese journal of cancer research = Chung-kuo yen cheng yen chiu. 2013;25(1):55-62. |
| 1. Yörük P, Dündar Ö, Yildizhan B, Tütüncü L, Pekin T. Comparison of the risk of malignancy index and self-constructed logisticregression models in preoperative evaluation of adnexal masses. Journal of Ultrasound in Medicine. 2008;27(10):1469-77. |
| **Exclusion reason: Duplicate data (n = 26)** |
| 1. Ameye L, Timmerman D, Valentin L, Paladini D, Zhang J, Van Holsbeke C, et al. Clinically oriented three-step strategy for assessment of adnexalpathology. Ultrasound in obstetrics & gynecology : the official journal of theInternational Society of Ultrasound in Obstetrics and Gynecology. 2012;40(5):582-91. |
| 1. Ameye L, Valentin L, Testa AC, Van Holsbeke C, Domali E, Van Huffel S, et al. A scoring system to differentiate malignant from benign masses in specificultrasound-based subgroups of adnexal tumors. Ultrasound in Obstetrics and Gynecology. 2009;33(1):92-101. |
| 1. ISRCTN. The UK’s Clinical Study Registry. Comparison of two ultrasound assessment tools for the diagnosis of ovariantumours in asymptomatic post-menopausal women. <https://trialsearchwhoint/Trial2aspx?TrialID=ISRCTN89034131>. 2012. |
| 1. ISRCTN. The UK’s Clinical Study Registry. Phase 4 of the International Ovarian Tumour Analysis study group: tocompare the referral pattern and cost-effectiveness of using Risk ofMalignancy Index (RMI) versus Logistic Regression model (LR2) to diagnoseadnexal masses prior to surgery. <https://trialsearchwhoint/Trial2aspx?TrialID=ISRCTN20772153>. 2013. |
| 1. Kaijser J, Van Gorp T, Smet ME, Van Holsbeke C, Sayasneh A, Epstein E, et al. Are serum HE4 or ROMA scores useful to experienced examiners for improving characterization of adnexal masses after transvaginal ultrasonography? Ultrasound Obstet Gynecol. 2014;43(1):89-97. |
| 1. Kaijser J, Van Gorp T, Van Hoorde K, Van Holsbeke C, Sayasneh A, Vergote I, et al. A comparison between an ultrasound based prediction model (LR2) and theRisk of Ovarian Malignancy Algorithm (ROMA) to assess the risk ofmalignancy in women with an adnexal mass. Gynecologic Oncology. 2013;129(2):377-83. |
| 1. Karlsen MA, Hogdall EVS, Christensen IJ, Borgfeldt C, Kalapotharakos G, Zdrazilova-Dubska L, et al. A novel diagnostic index combining HE4, CA125 and age may improve triageof women with suspected ovarian cancer - An international multicenterstudy in women with an ovarian mass. Gynecologic oncology. 2015;138(3):640-6. |
| 1. Landolfo C, Bourne T, Froyman W, Van Calster B, Ceusters J, Testa AC, et al. Benign descriptors and ADNEX in two-step strategy to estimate risk ofmalignancy in ovarian tumors: retrospective validation on IOTA 5multicenter cohort. Ultrasound in obstetrics & gynecology : the official journal of theInternational Society of Ultrasound in Obstetrics and Gynecology. 2022. |
| 1. Lycke M, Ulfenborg B, Kristjansdottir B, Sundfeldt K. Increased diagnostic accuracy of adnexal tumors with a combination ofestablished algorithms and biomarkers. Journal of Clinical Medicine. 2020;9(2). |
| 1. Moszynski R, Szpurek D, Szubert S, Sajdak S. Analysis of false negative results of subjective ultrasonography assessment of adnexal masses. GinekolPol. 2013;84:102-7. |
| 1. Nunes N, Yazbek J, Ambler G, Hoo W, Naftalin J, Jurkovic D. Prospective evaluation of the IOTA logistic regression model LR2 for thediagnosis of ovarian cancer. Ultrasound in obstetrics & gynecology : the official journal of theInternational Society of Ultrasound in Obstetrics and Gynecology. 2012;40(3):355-9. |
| 1. Radosa MP, Vorwergk J, Fitzgerald J, Kaehler C, Schneider U, Camara O, et al. Sonographic discrimination between benign and malignant adnexal masses inpremenopause. Ultraschall in der Medizin (Stuttgart, Germany : 1980). 2014;35(4):339-44. |
| 1. Rafal M, Dariusz S, Sebastian S, Stefan S. Analysis of false negative results of subjective ultrasonographyassessment of adnexal masses. Ginekologia Polska. 2013;84(2):102-7. |
| 1. Sayasneh A, Kaijser J, Preisler J, Johnson S, Stalder C, Husicka R, et al. A multicenter prospective external validation of the diagnosticperformance of IOTA simple descriptors and rules to characterize ovarianmasses. Gynecologic Oncology. 2013;130(1):140-6. |
| 1. Sladkevicius P, Jokubkiene L, Timmerman D, Fischerova D, Van Holsbeke C, Franchi D, et al. Vessel morphology depicted by three-dimensional power Doppler ultrasoundas second-stage test in adnexal tumors that are difficult to classify:prospective diagnostic accuracy study. Ultrasound in obstetrics & gynecology : the official journal of theInternational Society of Ultrasound in Obstetrics and Gynecology. 2021;57(2):324-34. |
| 1. Tantipalakorn C, Tinnangwattana D, Lerthiranwong T, Luewan S, Tongsong T. Comparisons of Effectiveness in Differentiating Benign from Malignant Ovarian Masses between Conventional and Modified Risk of Malignancy Index (RMI). International Journal of Environmental Research and Public Health. 2023;20(1). |
| 1. Terzic M, Dotlic J, Brndusic N, Arsenovic N, Likic I, Ladjevic N, et al. Histopathological diagnoses of adnexal masses: which parameters arerelevant in preoperative assessment? Ginekologia polska. 2013;84(8):700-8. |
| 1. Timmerman D, Van Calster B, Testa AC, Guerriero S, Fischerova D, Lissoni AA, et al. Ovarian cancer prediction in adnexal masses using ultrasound-basedlogistic regression models: a temporal and external validation study bythe IOTA group. Ultrasound in obstetrics & gynecology : the official journal of theInternational Society of Ultrasound in Obstetrics and Gynecology. 2010;36(2):226-34. |
| 1. Vaes E, Manchanda R, Autier P, Nir R, Nir D, Bleiberg H, et al. Differential diagnosis of adnexal masses: sequential use of the risk ofmalignancy index and HistoScanning, a novel computer-aided diagnostic tool. Ultrasound in obstetrics & gynecology : the official journal of theInternational Society of Ultrasound in Obstetrics and Gynecology. 2012;39(1):91-8. |
| 1. Valentin L, Ameye L, Jurkovic D, Metzger U, Lécuru F, Van Huffel S, et al. Which extrauterine pelvic masses are difficult to correctly classify asbenign or malignant on the basis of ultrasound findings and is there a wayof making a correct diagnosis? Ultrasound in Obstetrics and Gynecology. 2006;27(4):438-44. |
| 1. Valentin L, Ameye L, Savelli L, Fruscio R, Leone FP, Czekierdowski A, et al. Adnexal masses difficult to classify as benign or malignant usingsubjective assessment of gray-scale and Doppler ultrasound findings:logistic regression models do not help. Ultrasound in obstetrics & gynecology : the official journal of theInternational Society of Ultrasound in Obstetrics and Gynecology. 2011;38(4):456-65. |
| 1. Valentin L, Jurkovic D, Van Calster B, Testa A, Van Holsbeke C, Bourne T, et al. Adding a single CA 125 measurement to ultrasound imaging performed by anexperienced examiner does not improve preoperative discrimination betweenbenign and malignant adnexal masses. Ultrasound in Obstetrics and Gynecology. 2009;34(3):345-54. |
| 1. van den Akker PA, Zusterzeel PL, Aalders AL, Snijders MP, Samlal RA, Vollebergh JH, et al. Use of risk of malignancy index to indicate frozen section analysis in the surgical care of women with ovarian tumors. Int J Gynaecol Obstet. 2016;133(3):355-8. |
| 1. van den Akker PAJ, Aalders AL, Snijders MPLM, Kluivers KB, Samlal RAK, Vollebergh JHA, et al. Evaluation of the risk of malignancy index in daily clinical management ofadnexal masses. Gynecologic Oncology. 2010;116(3):384-8. |
| 1. Van Gorp T, Veldman J, Van Calster B, Cadron I, Leunen K, Amant F, et al. Subjective assessment by ultrasound is superior to the risk of malignancyindex (RMI) or the risk of ovarian malignancy algorithm (ROMA) indiscriminating benign from malignant adnexal masses. European Journal of Cancer. 2012;48(11):1649-56. |
| 1. Velayo CL, Reforma KN, Sicam RVG, Diwa MH, Sy ADR, Tantengco OAG. Improving diagnostic strategies for ovarian cancer in Filipino women usingultrasound imaging and a multivariate index assay. Cancer Epidemiology. 2022;81. |
| **Exclusion reason: ineligible index test (n = 58)**  *Studies that did not evaluate one of the specified models (RMI 1, RMI 2, RMI 3, LR2, SR, ADNEX, SA), used an incorrect definition of the RMI or menopausal status, or studies in which the handling of inconclusive results from SR or SA was unclear or not reported* |
| 1. Abd Elsalam SM, Hamed ST, Sayed MA. Diagnostic performance of GI-RADS reporting system in evaluation ofadnexal masses. Egyptian Journal of Radiology and Nuclear Medicine. 2020;51(1). |
| 1. Alcazar JL, Iturra A, Sedda F, Auba M, Ajossa S, Guerriero S, et al. Three-dimensional volume off-line analysis as compared to real-timeultrasound for assessing adnexal masses. European journal of obstetrics, gynecology, and reproductive biology. 2012;161(1):92-5. |
| 1. Anton C, Carvalho FM, Oliveira EI, Maciel GA, Baracat EC, Carvalho JP. A comparison of CA125, HE4, risk ovarian malignancy algorithm (ROMA), and risk malignancy index (RMI) for the classification of ovarian masses. Clinics (Sao Paulo). 2012;67(5):437-41. |
| 1. Aslam N, Banerjee S, Carr JV, Savvas M, Hooper R, Jurkovic D. Prospective evaluation of logistic regression models for the diagnosis ofovarian cancer. Obstetrics and Gynecology. 2000;96(1):75-80. |
| 1. Bahadur A, Bhattacharya N, Mundhra R, Khoiwal K, Chawla L, Singh R, et al. Comparison of Human Epididymis Protein 4, Cancer Antigen 125, and Ultrasound Prediction Model in Differentiating Benign from Malignant Adnexal Masses. Journal of mid-life health. 2023;14(3):176-83. |
| 1. Bayoglu Tekin Y, Dede FS. What is the success of ultrasonography of benign adnexal masses? The journal of obstetrics and gynaecology research. 2014;40(2):473-8. |
| 1. Behtash N, Rahmani M, Ghotbizadeh F, Zarchi MK, Mousavi A. Ultrasonography and computed tomography for management of adnexal massesin Iranian patients with suspected ovarian cancer: Results of aprospective study. Asian Pacific Journal of Cancer Prevention. 2009;10(2):201-4. |
| 1. Benacerraf BR, Finkler NJ, Wojciechoski C, Knapp RC. Sonographic accuracy in the diagnosis of ovarian masses. The Journal of Reproductive Medicine. 1990;35(5):491-5. |
| 1. Bristow RE, Hodeib M, Smith A, Chan DW, Zhang Z, Fung ET, et al. Impact of a multivariate index assay on referral patterns for surgicalmanagement of an adnexal mass. American Journal of Obstetrics and Gynecology. 2013;209(6):581.e1-.e8. |
| 1. Crestani A, Theodore C, Thomassin I, Levaillant JM, Touboul C. Mr and ultrasound fusion imaging to characterize ovarian masses. International Journal of Gynecological Cancer. 2019;29:A452. |
| 1. Dabi Y, Rockall A, Razakamanantsoa L, Guerra A, Fournier LS, Fotopoulou C, et al. O-RADS MRI scoring system has the potential to reduce the frequency of avoidable adnexal surgery. European journal of obstetrics, gynecology, and reproductive biology. 2024;294:135-42. |
| 1. Di Legge A, Testa AC, Ameye L, Van Calster B, Lissoni AA, Leone FP, et al. Lesion size affects diagnostic performance of IOTA logistic regressionmodels, IOTA simple rules and risk of malignancy index in discriminatingbetween benign and malignant adnexal masses. Ultrasound in obstetrics & gynecology : the official journal of theInternational Society of Ultrasound in Obstetrics and Gynecology. 2012;40(3):345-54. |
| 1. Dierickx I, Valentin L, Van Holsbeke C, Jacomen G, Lissoni AA, Licameli A, et al. Imaging in gynecological disease (7): clinical and ultrasound features ofBrenner tumors of the ovary. Ultrasound in obstetrics & gynecology : the official journal of theInternational Society of Ultrasound in Obstetrics and Gynecology. 2012;40(6):706-13. |
| 1. DiSantis DJ, Scatarige JC, Kemp G, Given FT, Hsiu JG, Cramer MS. A prospective evaluation of transvaginal sonography for detection ofovarian disease. American Journal of Roentgenology. 1993;161(1):91-4. |
| 1. Finkler NJ, Benacerraf B, Lavin PT, Wojciechoski C, Knapp RC. Comparison of Serum CA 125, Clinical Impression, and Ultrasound in the Preoperative Evaluation of Ovarian Masses. Obstetrics & Gynecology. 1988;72(4):659-64. |
| 1. Goel R, Singhal S, Manchanda S, Rajan S, Meena J, Bharti J. Comparison of Two-Dimensional IOTA Simple Rules and Three-Dimensional Ultrasonography in Preoperative Assessment of Adnexal Masses. The Indian journal of radiology & imaging. 2024;34(4):588-95. |
| 1. Gramellini D, Fieni S, Sanapo L, Casilla G, Verrotti C, Nardelli GB. Diagnostic accuracy of IOTA ultrasound morphology in the hands of lessexperienced sonographers. Australian and New Zealand Journal of Obstetrics and Gynaecology. 2008;48(2):195-201. |
| 1. Ismailova M, Nigmatjonov A, Usmanova Z. Clinical review on the differential diagnosis of benign and malignantovarian cancer: Differential diagnosis of benign and malignant ovariancancer. Annals of Clinical and Analytical Medicine. 2020;11:306-8. |
| 1. Jain M, Arora D, Malhotra A, Chandak S, Deriya A. Sonoelastographic Evaluation of Pelvic Adnexal Masses and its Association with Clinicopathological Findings at a Tertiary Care Centre in Western Uttar Pradesh, India. Journal of Clinical and Diagnostic Research. 2023;17(3):TC01-TC7. |
| 1. Jetani N, Gandhi SD, Parikh U. Original Research Article: Clinical Study On Diagnostic Accuracy Of ROMA (Risk Of Malignancy Algorhithm) Score In Predicting Epithelial Ovarian Cancers For Ovarian Mass. European Journal of Molecular and Clinical Medicine. 2022;9(7):938-51. |
| 1. Kadija S, Stefanovic A, Jeremic K, Radojevic MM, Nikolic L, Markovic I, et al. The utility of human epididymal protein 4, cancer antigen 125, and risk for malignancy algorithm in ovarian cancer and endometriosis. Int J Gynecol Cancer. 2012;22(2):238-44. |
| 1. de Kroon CD, van der Sandt HAGM, van Houwelingen JC, Jansen FW. Sonographic assessment of non-malignant ovarian cysts: Does sonohistologyexist? Human Reproduction. 2004;19(9):2138-43. |
| 1. Ledger A, Ceusters J, Valentin L, Testa A, Van Holsbeke C, Franchi D, et al. Multiclass risk models for ovarian malignancy: an illustration of prediction uncertainty due to the choice of algorithm. BMC medical research methodology. 2023;23(1):276. |
| 1. Leelahakorn S, Tangjitgamol S, Manusirivithaya S, Thongsuksai P, Jaroenchainon P, Jivangkul C. Comparison of ultrasound score, CA125, menopausal status, and risk ofmalignancy index in differentiating between benign and borderline ormalignant ovarian tumors. Journal of the Medical Association of Thailand = Chotmaihet thangphaet. 2005;88:S22-30. |
| 1. Liu C, Zhu Y, Dai K, Tan B, Dong H, Lin J, et al. Accurate prediction of benign and malignant adnexal tumors in surgical resection and conservative treatment: construction and external validation of a diagnostic model based on CEUS, HE4, and O-RADS US v2022 evaluation. Journal of Ovarian Research. 2025;18(1):123. |
| 1. Liu L, Cai W, Zheng F, Tian H, Li Y, Wang T, et al. Automatic segmentation model and machine learning model grounded in ultrasound radiomics for distinguishing between low malignant risk and intermediate-high malignant risk of adnexal masses. Insights into Imaging. 2025;16(1):14. |
| 1. Massobrio R, Mariani LL, Conti D, De Grandis T, Buonomo F, Badellino E, et al. Ultrasonographic diagnosis of adnexal masses: interobserver agreement in the interpretation of videos, using IOTA terminology. Archives of Gynecology and Obstetrics. 2024;309(1):211-8. |
| 1. Maturen KE, Blaty AD, Wasnik AP, Patel-Lippmann K, Robbins JB, Barroilhet L, et al. Risk stratification of adnexal cysts and cystic masses: Clinical performance of society of radiologists in ultrasound guidelines. Radiology. 2017;285(2):650-9. |
| 1. Merz E, Weber G, Bahlmann F, Kießlich R. A new sonomorphologic scoring system (Mainz score) for the assessment ofovarian tumors using transvaginal ultrasonography. Part I: A comparisonbetween the scoring-system and the assessment by an experiencedsonographer. Ultraschall in der Medizin. 1998;19(3):99-107. |
| 1. Ozcan HC, Balat O, Ugur MG, Kul S, Bozdag Z, Sucu S, et al. The management of adnexal masses in premenopausal patients: A ten-yearretrospective study at a single center. European Journal of Gynaecological Oncology. 2017;38(3):372-7. |
| 1. Panichyawat N, Tanmahasamut P, Jaishuen A, Asumpinwong C, Chantrapanichkul P. Prevalence of ovarian mass and diagnostic performance of ultrasonographypattern recognition among women at the Gynaecologic Ultrasonography Unitat University Hospital in Thailand. Journal of Obstetrics and Gynaecology. 2022;42(6):2260-4. |
| 1. Pascual A, Guerriero S, Rams N, Juez L, Ajossa S, Graupera B, et al. Clinical and ultrasound features of benign, borderline, and malignantinvasive mucinous ovarian tumors. European Journal of Gynaecological Oncology. 2017;38(3):382-6. |
| 1. Priyanka MB, Panda J, Samantroy S, Panda SR, Jena P. Comparison of Four Risk of Malignancy Indices for Preoperative Evaluation of Ovarian Masses: A Prospective Observational Study. Cureus. 2023;15(7):e41539. |
| 1. Pushpam P, Paul V, Seema. Assessment of Risk of Malignancy Index Scoring and Histopathological Correlation in the Diagnosis and Management of Adnexal Mass. International Journal of Pharmaceutical and Clinical Research. 2024;16(8):1523-7. |
| 1. Rai R, Bhutia PC, Tshomo U. Clinicopathological profile of adnexal masses presenting to atertiary-care hospital in Bhutan. South Asian journal of cancer. 2019;8(3):168-72. |
| 1. Reles A, Wein U, Lichtenegger W. Transvaginal color doppler sonography and conventional sonography in thepreoperative assessment of adnexal masses. Journal of Clinical Ultrasound. 1997;25(5):217-25. |
| 1. Sayasneh A, Kaijser J, Preisler J, Smith AA, Raslan F, Johnson S, et al. Accuracy of ultrasonography performed by examiners with varied training and experience in predicting specific pathology of adnexal masses. Ultrasound Obstet Gynecol. 2015;45(5):605-12. |
| 1. Schutter EMJ, Sohn C, Kristen P, Möbus V, Crombach G, Kaufmann M, et al. Estimation of probability of malignancy using a logistic model combiningphysical examination, ultrasound, serum CA 125, and serum CA 72-4 inpostmenopausal women with a pelvic mass: An international multicenterstudy. Gynecologic Oncology. 1998;69(1):56-63. |
| 1. Schutter EMJ, Kenemans P, Sohn C, Kristen P, Crombach G, Westermann R, et al. Diagnostic value of pelvic examination, ultrasound, and serum CA 125 inpostmenopausal women with a pelvic mass: An international multicenterstudy. Cancer. 1994;74(4):1398-406. |
| 1. Shekar NC, Dasappa P, Rangaiah N, Nagothi NP. Evaluation of risk of malignancy index 5—a new indicator indifferentiating benign and malignant ovarian masses. Journal of SAFOG. 2019;11(4):258-62. |
| 1. Shetty J, Saradha A, Pandey D, Bhat R, Pratap K, Bharatnur S. IOTA Simple Ultrasound Rules for Triage of Adnexal Mass: Experience fromSouth India. Journal of obstetrics and gynaecology of India. 2019;69(4):356-62. |
| 1. Sokalska A, Timmerman D, Testa AC, Van Holsbeke C, Lissoni AA, Leone FPG, et al. Diagnostic accuracy of transvaginal ultrasound examination for assigning aspecific diagnosis to adnexal masses. Ultrasound in Obstetrics and Gynecology. 2009;34(4):462-70. |
| 1. Sutantawibul A, Ruangvutilert P, Sunsaneevithayakul P, Boriboonhirunsarn D. A model for malignancy probability prediction of adnexal masses. Journal of the Medical Association of Thailand. 2003;86(8):742-9. |
| 1. Teng F, Xie H, Wei H, Che D, Wang H, Wu C, et al. Diagnostic value of the ovarian adnexal reporting and data system ultrasound in ovarian masses: a 2-center study. British Journal of Radiology. 2025;98(1167):448-57. |
| 1. Timmerman D, Van Calster B, Jurkovic D, Valentin L, Testa AC, Bernard JP, et al. Inclusion of CA-125 does not improve mathematical models developed to distinguish between benign and malignant adnexal tumors. J Clin Oncol. 2007;25(27):4194-200. |
| 1. Timmerman D, Van Calster B, Testa A, Savelli L, Fischerova D, Froyman W, et al. Predicting the risk of malignancy in adnexal masses based on the SimpleRules from the International Ovarian Tumor Analysis group. American Journal of Obstetrics and Gynecology. 2016;214(4):424-37. |
| 1. Tinnangwattana D, Vichak-Ururote L, Tontivuthikul P, Charoenratana C, Lerthiranwong T, Tongsong T. IOTA Simple Rules in Differentiating between Benign and Malignant AdnexalMasses by Non-expert Examiners. Asian Pacific journal of cancer prevention : APJCP. 2015;16(9):3835-8. |
| 1. Tjokroprawiro BA, Sari NK, Yuliati I, Mardiyana L. Characteristics of suspected malignant ovarian tumor patients whoperformed surgery at Tertiary Referral Hospital. Systematic Reviews in Pharmacy. 2020;11(5):181-4. |
| 1. Tongsong T, Tinnangwattana D, Vichak-Ururote L, Tontivuthikul P, Charoenratana C, Lerthiranwong T. Comparison of Effectiveness in Differentiating Benign from MalignantOvarian Masses between IOTA Simple Rules and Subjective SonographicAssessment. Asian Pacific journal of cancer prevention : APJCP. 2016;17(9):4377-80. |
| 1. Topuz S, Saygili H, Akhan S, Yavuz E, Turfanda A, Berkman S. Differentiation of benign and malignant adnexal masses: value of a morphologic scoring system. Eur J Gynaecol Oncol. 2005;26(2):209-12. |
| 1. Torres JC, Derchain SF, Faundes A, Gontijo RC, Martinez EZ, Andrade LA. Risk-of-malignancy index in preoperative evaluation of clinicallyrestricted ovarian cancer. São Paulo medical journal = Revista paulista de medicina. 2002;120(3):72-6. |
| 1. Utrilla-Layna J, Alcazar JL, Auba M, Laparte C, Olartecoechea B, Errasti T, et al. Performance of three-dimensional power Doppler angiography as third-step assessment in differential diagnosis of adnexal masses. Ultrasound Obstet Gynecol. 2015;45(5):613-7. |
| 1. Weber G, Merz E, Bahlmann F, Leber AM. A new sonomorphologic scoring-system (Mainz Score) for the assessment ofovarian tumors using transvaginal ultrasonography. Part II: A comparisonbetween the scoring-system and the assessment by an experiencedsonographer in postmenopausal women. Ultraschall in der Medizin. 1999;20(1):2-8. |
| 1. Wilailak S, Chan KKL, Chen CA, Nam JH, Ochiai K, Aw TC, et al. Distinguishing benign from malignant pelvic mass utilizing an algorithmwith HE4, menopausal status, and ultrasound findings. Journal of gynecologic oncology. 2015;26(1):46-53. |
| 1. Woolas R, Young L, Brinkmann D, Gardner F, Hadwin R, Woolas T, et al. Exploration of Preliminary Objective Triage by Menopause Score and CA 125 Result Prior to Accelerating Fast-Track Booking for Suspected Ovarian Cancer-A Role for the Pathway Navigator? Diagnostics (Basel, Switzerland). 2024;14(5). |
| 1. Wu M, Wang Q, Zhang M, Cao J, Chen Y, Zheng J, et al. Does Combing O-RADS US and CA-125 Improve Diagnostic Accuracy in Assessing Adnexal Malignancy Risk in Women With Different Menopausal Status? Journal of ultrasound in medicine : official journal of the American Institute of Ultrasound in Medicine. 2023;42(3):675-85. |
| 1. Xu J, Huang Z, Zeng J, Zheng Z, Cao J, Su M, et al. Value of Contrast-Enhanced Ultrasound Parameters in the Evaluation of Adnexal Masses with Ovarian–Adnexal Reporting and Data System Ultrasound. Ultrasound in Medicine and Biology. 2023;49(7):1527-34. |
| 1. Yanaranop M, Anakrat V, Siricharoenthai S, Nakrangsee S, Thinkhamrop B. Is the Risk of Ovarian Malignancy Algorithm Better Than Other Tests forPredicting Ovarian Malignancy in Women with Pelvic Masses? Gynecologic and obstetric investigation. 2017;82(1):47-53. |
| **Exclusion reason: other study population (n = 25)**  *E.g. studies including only women with a specific risk profile based on other risk scores or specific tumor characteristics (e.g. only solid tumors), pregnant women, children, women with a history of ovarian cancer treatment, or populations with >20% concomitant malignancies.* |
| 1. Bensaid C, Belda MALF, Metzger U, Larousserie F, Clément D, Chatellier G, et al. Performance of laparoscopy in identifying malignant ovarian cysts. Surgical Endoscopy and Other Interventional Techniques. 2006;20(9):1410-4. |
| 1. Chacon E, Arraiza M, Manzour N, Benito A, Mínguez JÁ, Vázquez-Vicente D, et al. Ultrasound examination, MRI, or ROMA for discriminating between inconclusive adnexal masses as determined by IOTA Simple Rules: A prospective study. International Journal of Gynecological Cancer. 2023;33(6):951-6. |
| 1. Czekierdowski A, Stachowicz N, Smolen A, Łoziński T, Guzik P, Kluz T. Performance of IOTA Simple Rules Risks, ADNEX Model, Subjective Assessment Compared to CA125 and HE4 with ROMA Algorithm in Discriminating between Benign, Borderline and Stage I Malignant Adnexal Lesions. Diagnostics. 2023;13(5). |
| 1. Daponte A, Stergioti E, Messinis IE. Risk scoring for adnexal masses and endoscopic management. Int J Gynaecol Obstet. 2007;96(1):42-3. |
| 1. Demidov VN, Lipatenkova J, Vikhareva O, Van Holsbeke C, Timmerman D, Valentin L. Imaging of gynecological disease (2): Clinical and ultrasoundcharacteristics of Sertoli cell tumors, Sertoli-Leydig cell tumors andLeydig cell tumors. Ultrasound in Obstetrics and Gynecology. 2008;31(1):85-91. |
| 1. Di Legge A, Pollastri P, Mancari R, Ludovisi M, Mascilini F, Franchi D, et al. Clinical and ultrasound characteristics of surgically removed adnexal lesions with largest diameter <= 2.5 cm: a pictorial essay. Ultrasound in obstetrics & gynecology : the official journal of the International Society of Ultrasound in Obstetrics and Gynecology. 2017;50(5):648-56. |
| 1. Dochez V, Randet M, Renaudeau C, Dimet J, Thuaut AL, Winer N, et al. Efficacy of he4, ca125, risk of malignancy index and risk of ovarianmalignancy index to detect ovarian cancer in women with presumed benignovarian tumours: A prospective, multicentre trial. Journal of Clinical Medicine. 2019;8(11). |
| 1. Franchi D, Boveri S, Fruscio R, Fischerova D, Guerriero S, Moruzzi MC, et al. Imaging in gynecological disease (8): ultrasound characteristics ofrecurrent borderline ovarian tumors. Ultrasound in obstetrics & gynecology : the official journal of theInternational Society of Ultrasound in Obstetrics and Gynecology. 2013;41(4):452-8. |
| 1. Hassan RMA, Ebrahim SAM, Kamal MRA, Tantawy HFA. The impact of using ovarian-adnexal reporting data system magnetic resonance imaging (O-RADS MRI) score on risk stratification of sonographically indeterminate adnexal masses. Egyptian Journal of Radiology and Nuclear Medicine. 2024;55(1). |
| 1. Lee SJ, Kim YH, Lee MY, Ko HS, Oh SY, Seol HJ, et al. Ultrasonographic evaluation of ovarian mass for predicting malignancy inpregnant women. Gynecologic Oncology. 2021;163(2):385-91. |
| 1. Lennox GK, Eiriksson LR, Reade CJ, Leung F, Mojtahedi G, Atenafu EG, et al. Effectiveness of the risk of malignancy index and the risk of ovarianmalignancy algorithm in a cohort of women with ovarian cancer: doeshistotype and stage matter? International journal of gynecological cancer : official journal of theInternational Gynecological Cancer Society. 2015;25(5):809-14. |
| 1. Li Y, Shao G, Wu M, Zhang F, Zhang Y, Shao C. Evaluation of American College of Radiology Ovarian-Adnexal Reporting and Data System ultrasound to predict malignancy risk in adnexal lesions. The journal of obstetrics and gynaecology research. 2024;50(2):225-32. |
| 1. Lu B, Liu C, Qi J, He W, Shi T, Zhu Y, et al. Comparison of contrast-enhanced ultrasound, IOTA simple rules and O-RADS for assessing the malignant risk of sonographically appearing solid ovarian masses. Journal of Gynecology Obstetrics and Human Reproduction. 2023;52(4). |
| 1. Moro F, Vagni M, Tran HE, Bernardini F, Mascilini F, Ciccarone F, et al. Radiomics analysis of ultrasound images to discriminate between benign and malignant adnexal masses with solid ultrasound morphology. Ultrasound in obstetrics & gynecology : the official journal of the International Society of Ultrasound in Obstetrics and Gynecology. 2024. |
| 1. Nikolova T, Zivadinovic R, Evtimovska N, Klisarovska V, Stanojevic M, Georgievska J, et al. Diagnostic performance of human epididymis protein 4 compared to acombination of biophysical and biochemical markers to differentiateovarian endometriosis from epithelial ovarian cancer in premenopausalwomen. Journal of Obstetrics and Gynaecology Research. 2017;43(12):1870-9. |
| 1. Numanoglu C, Kuru O, Sakinci M, Akbayir O, Ulker V. Ovarian fibroma/fibrothecoma: Retrospective cohort study shows limitedvalue of risk of malignancy index score. Australian and New Zealand Journal of Obstetrics and Gynaecology. 2013;53(3):287-92. |
| 1. Rabiej-Wronska E, Wiechec M, Pitynski K, Wiercinska E, Kotlarz A. Ultrasound differentiation between benign versus malignant adnexal massesin pregnant patients. Ginekologia polska. 2022;93(8):643-9. |
| 1. Seth S, Saxena S, Verma V, Kanti V, Gupta UK. Study of diagnostic efficacy of risk of malignancy index(RMI-II),neutrophil lymphocyte ratio(NLR) and platelet lymphocyte ratio(PLR) inpreoperative assessment of adnexal masses in females and itshistopathological correlation: Running title - Comparison of. European Journal of Molecular and Clinical Medicine. 2021;8(2):1757-69. |
| 1. Stukan M, Alcazar JL, Gȩbicki J, Epstein E, Liro M, Sufliarska A, et al. Ultrasound and clinical preoperative characteristics for discriminationbetween ovarian metastatic colorectal cancer and primary ovarian cancer: Acase-control study. Diagnostics. 2019;9(4). |
| 1. Sukumar K, Supriya D, Usha P. Evaluation and Management of Suspicious Adnexal Masses in Pregnancy- ARetrospective Study. Journal of Clinical and Diagnostic Research. 2022;16(1):QC09-QC13. |
| 1. Valentin L, Ameye L, Savelli L, Fruscio R, Leone FPG, Czekierdowski A, et al. Unilocular adnexal cysts with papillary projections but no other solidcomponents: is there a diagnostic method that can classify them reliablyas benign or malignant before surgery? Ultrasound in obstetrics & gynecology : the official journal of theInternational Society of Ultrasound in Obstetrics and Gynecology. 2013;41(5):570-81. |
| 1. Wu M, Wang Y, Su M, Wang R, Sun X, Zhang R, et al. Integrating Contrast-enhanced US to O-RADS US for Classification of Adnexal Lesions with Solid Components: Time-intensity Curve Analysis versus Visual Assessment. Radiology Imaging cancer. 2024;6(6):e240024. |
| 1. Wu M, Zhang M, Qu E, Sun X, Zhang R, Mu L, et al. A modified CEUS risk stratification model for adnexal masses with solid components: prospective multicenter study and risk adjustment. European Radiology. 2024;34(9):5978-88. |
| 1. Yenen MC, Alanbay I, Akturk E, Ercan CM, Coksuer H, Karasahin E, et al. Comparison of risk of malignancy indices; RMI 1-4 in borderline ovariantumor. European journal of gynaecological oncology. 2012;33(2):168-73. |
| 1. Zhang S, Yu S, Hou W, Li X, Ning C, Wu Y, et al. Diagnostic extended usefulness of RMI: comparison of four risk ofmalignancy index in preoperative differentiation of borderline ovariantumors and benign ovarian tumors. Journal of ovarian research. 2019;12(1):87. |
| **Exlusion reason: other outcome (n = 10)**  *Studies in which the outcome was limited to a specific histological subtype of ovarian cancer (e.g. only non-epithelial ovarian cancer) or studies that used a reference standard other than histology or follow-up for at least one year* |
| 1. Alanbay I, Akturk E, Coksuer H, Ercan M, Karasahin E, Dede M, et al. Comparison of risk of malignancy index (RMI), CA125, CA 19-9, ultrasoundscore, and menopausal status in borderline ovarian tumor. Gynecological endocrinology : the official journal of the InternationalSociety of Gynecological Endocrinology. 2012;28(6):478-82. |
| 1. Bruchim I, Ben-Harim Z, Piura E, Tepper R, Fishman A. Preoperative clinical and radiological features of metastatic ovariantumors. Archives of Gynecology and Obstetrics. 2013;288(3):615-9. |
| 1. Czekierdowski A, Stachowicz N, Smoleń A, Kluz T, Łoziński T, Miturski A, et al. Sonographic assessment of complex ultrasound morphology adnexal tumors inpregnant women with the use of iota simple rules risk and adnex scoringsystems. Diagnostics. 2021;11(3). |
| 1. Epstein E, Van Calster B, Timmerman D, Nikman S. Subjective ultrasound assessment, the ADNEX model and ultrasound-guidedtru-cut biopsy to differentiate disseminated primary ovarian cancer frommetastatic non-ovarian cancer. Ultrasound in obstetrics & gynecology : the official journal of theInternational Society of Ultrasound in Obstetrics and Gynecology. 2016;47(1):110-6. |
| 1. Froyman W, Wynants L, Landolfo C, Bourne T, Valentin L, Testa A, et al. Validation of the performance of International Ovarian Tumor Analysis(IOTA) methods in the diagnosis of early stage ovarian cancer in a non-screening population. Diagnostics. 2017;7(2). |
| 1. Gaurilcikas A, Gedgaudaite M, Cizauskas A, Atstupenaite V, Paskauskas S, Gaurilcikiene D, et al. Performance of the IOTA ADNEX Model on Selected Group of Patients withBorderline Ovarian Tumours. Medicina (Kaunas, Lithuania). 2020;56(12). |
| 1. Pandey S, Singh S, Lal P, Gupta R, Dwivedi D, Yadav R. Aimed to compare IOTA (International ovarian tumor analysis) and RMI3 (Risk of malignancy index3) in evaluation of adnexal masses for prediction of ovarian malignancy in premenopausal and postmenopausal females. Indian Journal of Gynecologic Oncology. 2024;23(1):12. |
| 1. Rius M, Fusté P, Ros C, Martínez-Zamora Á, deGuirior C, Gracia M, et al. HE4 might be a more useful tumor biomarker to detect malignancy inpatients with ovarian endometrioma when malignancy is suspected. Journal of International Medical Research. 2021;49(9). |
| 1. Tongsong T, Wanapirak C, Tantipalakorn C, Tinnangwattana D. Sonographic Diagnosis of Tubal Cancer with IOTA Simple Rules Plus PatternRecognition. Asian Pacific journal of cancer prevention : APJCP. 2017;18(11):3011-5. |
| 1. Yazbek J, Aslam N, Tailor A, Hillaby K, Raju KS, Jurkovic D. A comparative study of the risk of malignancy index and the ovariancrescent sign for the diagnosis of invasive ovarian cancer. Ultrasound in Obstetrics and Gynecology. 2006;28(3):320-4. |
| **Exclusion reason: not peer-reviewed (n = 1)** |
| 1. Moro F, Momi M, Bertoldo V, Ledger A, Barreñada L, Ceusters J, et al. External validation of ultrasound-based models for discrimination between benign and malignant adnexal masses in Italy: the prospective multicenter IOTA phase 6 study. medRxiv. 2024:2024.12.23.24319517. |
| **Exclusion reason: model development study (n = 6)** |
| 1. Jacobs I, Oram D, Fairbanks J, Turner J, Frost C, Grudzinskas JG. A risk of malignancy index incorporating CA 125, ultrasound and menopausalstatus for the accurate preoperative diagnosis of ovarian cancer. Br J Obstet Gynaecol. 1990;97(10):922-9. |
| 1. Timmerman D, Testa AC, Bourne T, Ferrazzi E, Ameye L, Konstantinovic ML, et al. Logistic regression model to distinguish between the benign and malignant adnexal mass before surgery: a multicenter study by the International Ovarian Tumor Analysis Group. J Clin Oncol. 2005;23(34):8794-801. |
| 1. Timmerman D, Testa A, Bourne T, Ameye L, Jurkovic D, Holsbeke CV, et al. Simple ultrasound-based rules for the diagnosis of ovarian cancer. Ultrasound Obstet Gynecol. 2008;31:681-90. |
| 1. Tingulstad S, Hagen B, Skjeldestad FE, Onsrud M, Kiserud T, Halvorsen T, et al. Evaluation of a risk of malignancy index based on serum CA125, ultrasoundfindings and menopausal status in the pre-operative diagnosis of pelvicmasses. Br J Obstet Gynaecol. 1996;103(8):826-31. |
| 1. Tingulstad S, Hagen B, Skjeldestad FE, Halvorsen T, Nustad K, Onsrud M. The risk-of-malignancy index to evaluate potential ovarian cancers inlocal hospitals. Obstet Gynecol. 1999;93(3):448-52. |
| 1. Van Calster B, Van Hoorde K, Valentin L, Testa AC, Fischerova D, Van Holsbeke C, et al. Evaluating the risk of ovarian cancer before surgery using the ADNEX model to differentiate between benign, borderline, early and advanced stage invasive, and secondary metastatic tumours: prospective multicentre diagnostic study. BMJ : British Medical Journal. 2014;349:g5920. |

**Table S4** Individual study results not included in analyses

*Table S4.1. Studies that classified borderline tumors as benign or excluded borderline tumors*

| **Study** | **Model** | **Reported sensitivity**  **%** | **Reported specificity**  **%** | **Classification of borderline tumors** |
| --- | --- | --- | --- | --- |
| Akturk2011 | RMI 1 – 200 | 75.0 | 89.0 | Excluded |
| Akturk2011 | RMI 1 – 250 | 65.0 | 95.0 | Excluded |
| Akturk2011 | RMI 2 – 200 | 75.0 | 85.0 | Excluded |
| Akturk2011 | RMI 2 – 250 | 75.0 | 87.0 | Excluded |
| Akturk2011 | RMI 3 – 200 | 70.0 | 87.0 | Excluded |
| Akturk2011 | RMI 3 – 250 | 77.0 | 94.0 | Excluded |
| Lems2025* | ADNEX – 1% | 98.1 | 23.9 | Benign |
| Lems2025* | ADNEX – 5% | 98.1 | 58.1 | Benign |
| Lems2025* | ADNEX – 10% | 94.3 | 70.6 | Benign |
| Lems2025* | ADNEX – 15% | 86.8 | 79.0 | Benign |
| Lems2025* | ADNEX – 20% | 81.1 | 83.9 | Benign |
| Lems2025* | ADNEX – 25% | 81.1 | 85.5 | Benign |
| Lems2025* | ADNEX – 30% | 79.2 | 88.7 | Benign |
| Lems2025* | ADNEX – 40% | 75.5 | 91.9 | Benign |
| Lems2025* | ADNEX – 50% | 66.0 | 94.8 | Benign |
| Mahajan2020 | RMI 2 – 200 | 72.7 | 94.9 | Benign |
| VandenAkker2011 | RMI 3 – 200 | 76.0 | 82.0 | Benign |
| VandenAkker2011 | RMI 3 – 250 | 72.0 | 86.0 | Benign |
| Sundar2024* | RMI 1 – 200 | 78.2 | 84.4 | Benign |
| Sundar2024* | RMI 1 – 250 | 75.8 | 81.1 | Benign |
| Sundar2024* | Simple Rules + Malignant | 85.8 | 69.8 | Benign |
| Sundar2024* | ADNEX – 3% | 99.2 | 28.8 | Benign |
| Sundar2024* | ADNEX – 10% | 92.4 | 54.8 | Benign |

*Concerns additional data. The original data of the paper, classifying borderline tumors as malignant, are included in primary analysis.

*Table S4.2 Studies with alternative cut-off ADNEX model not included in primary analysis*

| **Study** | **Model** | **Reported sensitivity**  **% (95% CI)** | **Reported specificity** | **Classification of borderline tumors** |
| --- | --- | --- | --- | --- |
| ***Cut-off 1%*** | | | | |
| Lems2025 | ADNEX – 1% | 98.7  (94.4 – 99.0) | 25.9  (21.0 – 31.2) | Malignant |
| Pascual2024 | ADNEX – 1% | 99  (89 – 99) | 13  (12 – 15) | Malignant |
| Sayasneh2016 | ADNEX – 1% | 100  (97.4 – 100) | 11.9  (9.1 – 15.5) | Malignant |
| Spagnol2024 | ADNEX – 1% | 100  (96 – 100) | 7  (5 – 9) | Malignant |
| vanCalster2020 | ADNEX – 1% | 99.4 | 16.2 | Malignant |
| ***Cut-off 3%*** | | | | |
| Araujo2017 | ADNEX – 3% | 100.0 | 22.2 | Malignant |
| Borges2024 | ADNEX – 3% | 99.3 | 49.1 | Malignant |
| Chen2019 | ADNEX – 3% | 97.3  (91.0 – 100.0) | 32.5  (26.0 – 39.0) | Malignant |
| He2021 | ADNEX – 3% | 97.5  (95.0 – 99.5) | 69.5  (64.9 – 74.1) | Malignant |
| Pascual2024 | ADNEX – 3% | 88  (74 – 95) | 93  (92 – 94) | Malignant |
| Sayasneh2016 | ADNEX – 3% | 100  (97.4 – 100) | 30.6  (26.3 – 35.3) | Malignant |
| Spagnol2024 | ADNEX – 3% | 96  (90 -98) | 41  (37 – 46) | Malignant |
| Sundar2024 | ADNEX – 3% | 98.6  (96.6 – 99.6) | 30.8  (27.5 – 34.4) | Malignant |
| vanCalster2020 | ADNEX – 3% | 96.8 | 58.9 | Malignant |
| ***Cut-off 15%*** | | | | |
| Araujo2017 | ADNEX – 15% | 91.2 | 71.4 | Malignant |
| Borges2024 | ADNEX – 15% | 92.3 | 88.8 | Malignant |
| Chen2019 | ADNEX – 15% | 89.3  (80.0 – 95.0) | 83.7  (78.0 – 89.0) | Malignant |
| ElsnerHernández2024 | ADNEX – 15% | 94.0  (93.7 – 94.3) | 67.4  (67.3 – 67.6) | Malignant |
| He2021 | ADNEX – 15% | 87.6  (82.6 – 92.0) | 95.5  (93.9 – 97.7) | Malignant |
| Hiett2022 | ADNEX – 15% | 95.0  (81.8 – 99.1) | 72.7  (63.2 – 80.1) | Malignant |
| Lems2025 | ADNEX – 15% | 83.1  (73.7 – 90.3) | 83.6  (79.0 – 87.6) | Malignant |
| Pascual2024 | ADNEX – 15% | 80  (66 – 89) | 96  (95 – 97) | Malignant |
| Poonyakanok2021 | ADNEX – 15% | 98.4  (91.2 – 100) | 90.5  (86.6 – 93.6) | Malignant |
| Sayasneh2016 | ADNEX – 15% | 94.4  (90.0 – 97.0) | 75.2  (70.7 – 79.2) | Malignant |
| Spagnol2024 | ADNEX – 15% | 77  (68 – 84) | 95  (92 – 96) | Malignant |
| vanCalster2020 | ADNEX – 15% | 90.4 | 87.5 | Malignant |
| ***Cut-off 25%*** | | | | |
| Borges2024 | ADNEX – 25% | 84.6 | 92.8 | Malignant |
| Lems2025 | ADNEX – 25% | 71.4  (60.7 – 80.7) | 88.5  (84.4 – 91.8) | Malignant |
| Pascual2025 | ADNEX – 25% | 64  (50 – 76) | 98  (97 – 98) | Malignant |
| Spagnol2024 | ADNEX – 25% | 63  (53 – 72) | 96  (94 – 97) | Malignant |
| vanCalster2020 | ADNEX – 25% | 84.0 | 92.1 | Malignant |
| ***Cut-off 50%*** | | | | |
| Lems2025 | ADNEX – 50% | 57.1  (46.0 – 67.8) | 97.6  (95.3 – 98.9) | Malignant |
| Pascual2024 | ADNEX – 50% | 35  (23 – 48) | 99  (99 – 99) | Malignant |
| Spagnol2024 | ADNEX – 50% | 47  (38 – 57) | 98  (96 – 99) | Malignant |
| vanCalster2020 | ADNEX – 50% | 72.4 | 96.9 | Malignant |

**Table S5** Characteristics of included studies

| Study details | Index test(s) with cut-off | Selection criteria | Participant details |
| --- | --- | --- | --- |
| Akturk (2011) [^1^](#_ENREF_1)  Study design: prospective cohort  Setting: tertiary care  Number of centers: 1  Accrual: Oct 2008 – Feb 2010  Country: Turkey  Funding: NR | RMI 1 (200, 250)  RMI 2 (200, 250)  RMI 3 (200, 250) | *Inclusion:* Women with pelvic masses scheduled for laparotomy or laparoscopy  *Exclusion:* NR  Type of ultrasound: TVE + TAE in case of a large mass  Experience examiner: NR | Included masses: 100  Age: NR  %Postmenopausal: 23%  CA-125: NR  Prevalence of malignancy: 20%  Prevalence of malignancy excl borderline: NR  Histology / follow-up: 100% / 0%  Time between test and surgery: NR |
| Al Musalhi (2016) [^2^](#_ENREF_2)  Study design: prospective cohort  Setting: secondary care  Number of centers: 1  Accrual: Mar 2014 – Apr 2015  Country: Oman  Funding: None | RMI 2 (200) | *Inclusion:* Patients who attended the gynecology department for evaluation of an ovarian mass and underwent surgical intervention  *Exclusion:* NR  Type of ultrasound: NR  Experience examiner: specialized gynaecologists | Included masses: 213  Age: NR  %Postmenopausal: 24%  CA-125: NR  Prevalence of malignancy: 22.5%  Prevalence of malignancy excl borderline: 19.2%  Histology / follow-up: 100% / 0%  Time between test and surgery: NR |
| Alcazar (2013) [^3^](#_ENREF_3)  Study design: prospective cohort  Setting: tertiary care  Number of centers: 2  Accrual: Jan 2011 – Jun 2012  Country: Spain  Funding: NR | Simple Rules + Malignant  Simple Rules + Subjective Assessment | *Inclusion:* Women with an adnexal mass, referred to one of two Spanish university centers  *Exclusion:* Pregnancy, spontaneous resolution of the mass by the time of a 2- to 3-month follow-up scan, surgery not performed because of physician’s and/or patient’s decision at follow-up, or surgery performed in another center  Type of ultrasound: TVE + TAE in case of a large mass  Experience examiner: SR: one 4rth year trainee and one junior staff with less than 1 yr of experience in ultrasound; SA was performed by expert examiners | Included masses: 340  Age: mean 42.1 (SD 13.2), range 13-70  %Postmenopausal: 23%  CA-125: NR  Prevalence of malignancy: 16.2%  Prevalence of malignancy excl borderline: 11.5%  Histology / follow-up: 100% / 0%  Time between test and surgery: < 3 weeks |
| Alcazar (2016) [^4^](#_ENREF_4)  Study design: prospective cohort  Setting: tertiary care  Number of centers: 2  Accrual: Dec 2012 – Dec 2014  Country: Spain  Funding: NR | Simple Rules + Malignant  Simple Rules + Subjective Assessment | *Inclusion:* Patients >18 years presenting with at least one adnexal mass  *Exclusion:* Pregnancy at the time of the ultrasound evaluation, refusal to undergo transvaginal or transrectal ultrasound, surgical removal of the tumor more than 4 months after the ultrasound scan and follow-up of less than 12 months at the time of data analysis if expectant management was chosen.  Type of ultrasound: TVE + TAE in case of a large mass  Experience examiners: For SR: Non-expert examiners (staff gynaecologists trained in ultrasound but without special interest in gynecological ultrasound). SA was performed by expert examiners | Included masses: 666  Age: median 41, range 18-81  %Postmenopausal:  CA-125: NR  Prevalence of malignancy: 8.0%  Prevalence of malignancy excl borderline: 6.9%  Histology / follow-up: 54% / 46%  Time between test and surgery: < 4 months |
| Andersen (2003) [^5^](#_ENREF_5)  Study design: prospective cohort  Setting: mixed  Number of centers: 3  Accrual: Jul 1999 – Aug 2001  Country: Norway  Funding: NR | RMI 2 (200) | *Inclusion:* All women 30 years of age or older with a pelvic mass referred to the departments of gynecology in the Country of Northern Jutland  *Exclusion:* NR  Type of ultrasound: TVE  Experience examiner: EFSUMB level II | Included masses: 219  Age: mean 50.3 (SD 13.6), range 18-82  %Postmenopausal: 56.3%  CA-125 (U/ml): mean 67.3 (SD 102.1)  Prevalence of malignancy: 19.6%  Prevalence of malignancy excl borderline: 19.7%  Histology / follow-up: 100% / 0%  Time between test and surgery: NR |
| Antovska (2011) [^6^](#_ENREF_6)  Study design: prospective cohort  Setting: tertiary care  Number of centers: 1  Accrual: Jan 2009 – Jan 2010  Country: Macedonia  Funding: NR | RMI 2 (200) | *Inclusion:* The presence of an ovarian tumor requiring surgical treatment  *Exclusion:* Refusal to undergo surgery  Type of ultrasound: NR  Experience examiner: NR | Included masses: 115  Age: NR  %Postmenopausal: 36.5%  CA-125 (U/ml): NR  Prevalence of malignancy: 35.7%  Prevalence of malignancy excl borderline: NR  Histology / follow-up: 100% / 0%  Time between test and surgery: NR |
| Araujo (2017) [^7^](#_ENREF_7)  Study design: prospective cohort  Setting: tertiary care  Number of centers: 1  Accrual: Feb 2014 – Nov 2015  Country: Brasil  Funding: government (Research Support Foundation of the State of São Paulo) | ADNEX (3%; 5%; 10%; 15%) | *Inclusion:* Women who had been referred to the ovarian pelvic oncology outpatient clinic due to the presence of an adnexal mass  *Exclusion:* No indication for surgery, ultrasound performed by examiner not trained in IOTA terms, non-ovarian histopathology, ectopic pregnancy, incomplete ultrasound data, absence of CA-125, >120 days between ultrasound and surgery, previous history of ovarian cancer, death before surgery  Type of ultrasound: TAE + TVE  Experience examiner: ultrasonographers with > 10 years' experience and >5000 pelvic ultrasound assessments | Included masses: 131  Age: NR  %Postmenopausal: NR  CA-125 (U/ml): NR  Prevalence of malignancy: 51,9%  Prevalence of malignancy excl borderline: 39.7%  Histology / follow-up: 100% / 0%  Time between test and surgery: <120 days |
| Arun-Muthuvel (2014) [^8^](#_ENREF_8)  Study design: prospective cohort  Setting: tertiary care  Number of centers: 1  Accrual: Jul 2011 – Jul 2013  Country: India  Funding: NR | RMI 1 (200, 250) | *Inclusion:* Women of all ages admitted with an ovarian mass in the SRMC Hospital  *Exclusion:* Women that did not undergo surgery or did not have an ultrasound or CA-125 pre-operatively; women whose histopathology turned out to be leiomyoma  Type of ultrasound: TVE + TAE  Experience examiner: Sonologists specially trained in obstetrical and gynecological ultrasonography | Included masses: 467  Age: NR  %Postmenopausal: 22.1%  CA-125: NR  Prevalence of malignancy: 22.5%  Prevalence of malignancy excl borderline: 19.1%  Histology / follow-up: 100% / 0%  Time between test and surgery: NR |
| Aslam (2000) [^9^](#_ENREF_9)  Study design: prospective cohort  Setting: mixed  Number of centers: 2  Accrual: Jul 1997 – Sep 1998  Country: UK  Funding: NR | RMI 1 (200)  RMI 2 (200) | *Inclusion:* Women with known adnexal masses, due to be admitted for surgery  *Exclusion:* NR  Type of ultrasound: TVE  Experience examiner: NR | Included masses: 61  Age: NR  %Postmenopausal: 41%  CA-125: NR  Prevalence of malignancy: 37.7%  Prevalence of malignancy excl borderline: 31.1%  Histology / follow-up: 100% / 0%  Time between test and surgery: NR |
| Auekitrungrueng (2019) [^10^](#_ENREF_10)  Study design: retrospective cohort in which ultrasound model variables were collected at the time of ultrasound examination  Setting: tertiary care  Number of centers: 1  Accrual: Apr 2010 – Mar 2018  Country: Thailand  Funding: Government: Chiang Mai University Research Fund | RMI 1 (200)  RMI 2 (200) | *Inclusion:* Women diagnosed with an adnexal mass, either by previous pelvic ultrasound examination or by vaginal examination, without a known diagnosis of the ovarian mass prior to surgery  *Exclusion:* NR  Type of ultrasound: TVE + TAE  Experience examiner: Certified gynaecologists and residents in gynecology after training in ultrasound examination by an experienced sonographer | Included masses: 479  Age: mean 42.1 (SD 12.5), range 12-80  %Postmenopausal: 24%  CA-125: NR  Prevalence of malignancy: 30.3%  Prevalence of malignancy excl borderline: 27.1%  Histology / follow-up: 100% / 0%  Time between test and surgery: <24h |
| Borges (2024) [^11^](#_ENREF_11)  Study design: prospective cohort  Setting: tertiary care  Number of centers: 3  Accrual: Jan 2016 – Dec 2021  Country: Portugal  Funding: NR | RMI 1 (200 LR2 (10%)  ADNEX (3%, 5%, 10%, 15%, 20%, 25%, 30%) | *Inclusion:* Women with at least one adnexal mass, detected by transvaginal ultrasound, considered to not be clearly a physiological cyst, who underwent laparotomic or laparoscopic surgery with a time interval after ultrasound assessment no longer than 120days  *Exclusion:* Patients <18years, pregnancy at the time of ultrasound examination and refusal to participate or withdrawal of informed consent.  Type of ultrasound: TVE + TAE  Experience examiner: EFSUMB level II and III sonologists with IOTA certification | Included masses: 571  Age: median 47, range 18-88  %Postmenopausal: 40.1%  CA-125: median 16.9, range 2-30.000  Prevalence of malignancy: 25.0%  Prevalence of malignancy excl borderline: 17.7%  Histology / follow-up: 100% / 0%  Time between test and surgery: <120 days |
| Campos (2016) [^12^](#_ENREF_12)  Study design: prospective cohort  Setting: tertiary care  Number of centers: 1  Accrual: Jan 2010 – Jan 2014  Country: Brasil  Funding: Government: Research Support Foundation of the State of São Paulo - Fapesp and by Conselho Nacional de Desenvolvimento Ceintífico e Tecnológico (CNPq) | RMI 1 (200, 250)  RMI 2 (200, 250)  RMI 3 (200, 250) | *Inclusion:* Women who underwent surgery for an adnexal mass  *Exclusion:* NR  Type of ultrasound: TVE + TAE  Experience examiner: Gynaecologists with between 2 - 12 years experience | Included masses: 158  Age: mean 49.2  %Postmenopausal: 46.2%  CA-125: NR  Prevalence of malignancy: 32.3%  Prevalence of malignancy excl borderline: 27.2%  Histology / follow-up: 100% / 0%  Time between test and surgery: < 120 days |
| Chankrachang (2025) [^13^](#_ENREF_13)  Study design: prospective cohort  Setting: tertiary care  Number of centers: 1  Accrual: NR  Country: Thailand  Funding: None | ADNEX (10%) | *Inclusion:* Patients diagnosed with an adnexal mass or ovarian tumor detected by prior ultrasound or pelvic examination; with no known diagnosis of the adnexal mass before surgery; with available CA-125 test results obtained within three months before surgery  *Exclusion:* Patients who underwent surgery more than 24 h after the ultrasound examination; with incomplete ultrasound evaluation; without available CA-125 results  Type of ultrasound: TVE + TAE in case of a large mass  Experience examiner: second- or third year obsetetrics and gynecology residents | Included masses: 342  Age: mean 41.4  %Postmenopausal: 22.5%  CA-125: NR  Prevalence of malignancy: 28.7%  Prevalence of malignancy excl borderline: 26.3%  Histology / follow-up: 100% / 0%  Time between test and surgery: < 24h |
| Chen (2019) [^14^](#_ENREF_14)  Study design: retrospective cohort in which ultrasound model variables were collected at the time of ultrasound examination  Setting: tertiary care  Number of centers: 2  Accrual: May 2017 – Dec 2017  Country: China  Funding: Government (Shanghai Municipal Education Comission-Gaofeng Clinical Medicine Grant Support) | ADNEX (3%; 5%; 10%) | *Inclusion:* Women with at least one adnexal mass detected by transvaginal ultrasonography, that was determined not to be a physiological cyst, prepared to undergo surgery, with a time interval between ultrasound and surgery < 120 days, without previous history of ovarian cancer  *Exclusion:* NR  Type of ultrasound: TVE + TAE in case of a large mass  Experience examiner: Experts, >10 years experience | Included masses: 278  Age: NR  %Postmenopausal: 35,3%  CA-125 (U/ml): NR  Prevalence of malignancy: 27.0%  Prevalence of malignancy excl borderline: 20.5%  Histology / follow-up: 100% / 0%  Time between test and surgery: < 120 days |
| Chen (2022) [^15^](#_ENREF_15)  Study design: retrospective cohort in which ultrasound model variables were collected at the time of ultrasound examination  Setting: tertiary care  Number of centers: 1  Accrual: Jan 2019 – Nov 2019  Country: China  Funding: Government: Medical Innovation Project and Natural Science Foundation of Shanghai Science and Technology Commission, National Natural Science Foundation of China | Subjective Assessment | *Inclusion:* Patients with at least one persisting ovarian tumor detected at ultrasound, who underwent a surgical procedure with histopathologic results, with a maximum interval of 30 days between ultrasound examination and surgery, and without a previous history of ovarian cancer  *Exclusion:* Histopathologic analysis–confirmed uterine sarcomas or nongynecologic tumors, inconclusive histopathologic results, poor US image quality.  Type of ultrasound: TVE + TAE  Experience examiner: Expert with  11 years of clinical experience and 14 years of ultrasound experience | Included masses: 85  Age: mean 46.8 (SD 15.5), range 18-85  %Postmenopausal: NR  CA-125: median 21.3, IQR 14.5-19.2  Prevalence of malignancy: 28.2%  Prevalence of malignancy excl borderline: 22.4%  Histology / follow-up: 100% / 0%  Time between test and surgery: <30 days |
| Christiansen (2021) [^16^](#_ENREF_16)  Study design: retrospective cohort in which ultrasound model variables were collected at the time of ultrasound examination  Setting: mixed  Number of centers: 2  Accrual: 2015 – 2019  Country: Sweden  Funding: NR | Simple Rules + Malignant  Simple Rules + Subjective Assessment  Subjective Assessment | *Inclusion:* Women with ovarian lesions who had undergone structured expert ultrasound assessment with either surgery within 120 days after ultrasound examination or follow-up for a minimum of 3 years, or until resolution of the lesion  *Exclusion:* NR  Type of ultrasound: TVE  Experience examiner: all certified as second-opinion expert sonographers by the Swedish Society of Obstetrics and Gynecology. Experience 7 – 23 years | Included masses: 150  Age: NR  %Postmenopausal: NR  CA-125: NR  Prevalence of malignancy: 50%  Prevalence of malignancy excl borderline: 40%  Histology / follow-up: NR  Time between test and surgery: <120 days |
| Daemen (2011) [^17^](#_ENREF_17)  Study design: prospective cohort  Setting: mixed  Number of centers: 19  Accrual: Nov 2005 – Oct 2007  Country: Sweden, Belgium, Italy, Poland, UK, the Czech Republic, China, Canada  Funding: Government: Research Council KUL, (2) Flemish Government, (3) Belgian Federal Science Policy Office, (4) EU-RTD, (5) Swedish Medical Research Council | Subjective Assessment | *Inclusion:* Patients presenting with at least one adnexal mass who had an ultrasound examination by a principal investigator at one of the participating centers  *Exclusion:* Patients who were pregnant, refused transvaginal ultrasonography and those who did not have surgical removal of the mass within 120 days after the ultrasound examination  Type of ultrasound: TVE + TAE in case of a large mass  Experience examiner: EFSUMB level III expert examiners | Included masses: 1938  Age: mean 46, range 11-94  %Postmenopausal: 38.3%  CA-125: NR  Prevalence of malignancy: 28%  Prevalence of malignancy excl borderline: 22.2%  Histology / follow-up: 100% / 0%  Time between test and surgery: <120 days |
| Dakhly (2019) [^18^](#_ENREF_18)  Study design: prospective cohort  Setting: tertiary care  Number of centers: 1  Accrual: Apr 2016 – Oct 2018  Country: Egypt  Funding: NR | Simple Rules + Malignant  Simple Rules + Subjective Assessment  Subjective Assessment | *Inclusion:* Women aged between 20 and 70 years with adnexal masses measuring more than 5cm in size who were scheduled for abdominal exploration  *Exclusion:* Patients with complicated ovarian masses (e.g. torsion, infection of hemorrhage), pregnant women, surgically unfit or refusal to participate  Type of ultrasound: TVE + TAE in case of a large mass  Experience examiner: SR: level II examiner; SA: expert examiner, level III | Included masses: 396  Age: mean 37.1 (SD 13.2)  %Postmenopausal: NR  CA-125: NR  Prevalence of malignancy: 23.7%  Prevalence of malignancy excl borderline: 19.4%  Histology / follow-up: 100% / 0%  Time between test and surgery: NR |
| DangThiMinh (2024) [^19^](#_ENREF_19)  Study design: prospective cohort  Setting: unclear  Number of centers: 1  Accrual: Dec 2020 – May 2022  Country: Vietnam  Funding: None | Simple Rules + Malignant | *Inclusion:* Women scheduled for surgery due to an ovarian tumor  *Exclusion:* Women with emergency surgery due to torsion or ruptured ovarian tumors, recurrent ovarian cancer, ovarian tumors associated with other cancers, and women less than 12 weeks pregnant  Type of ultrasound: TVE + TAE  Experience examiner: Three resident gynaecologists with less than one year experience in gynecological ultrasound, who had training of an expert for two weeks | Included masses: 424  Age: 38.5 (SD 13.2)  %Postmenopausal: 15.6%  CA-125: NR  Prevalence of malignancy: 9.2%  Prevalence of malignancy excl borderline: 7.3%  Histology / follow-up: 100% / 0%  Time between test and surgery: NR |
| Dewangan (2024) [^20^](#_ENREF_20)  Study design: prospective cohort  Setting: unclear  Number of centers: 1  Accrual: Nov 2019 – Mar 2021  Country: India  Funding: None | RMI 1 (200)  Simple Rules + Malignant | *Inclusion:* Women with an adnexal mass or masses on examination scheduled for surgery  *Exclusion:* Women with an adnexal mass or masses on examination scheduled for surgery  Type of ultrasound: TVE + TAE  Experience examiner: unknown, radiologists | Included masses: 70  Age: mean 33, range 18-80  %Postmenopausal: 18.6%  CA-125: NR  Prevalence of malignancy: 15.7%  Prevalence of malignancy excl borderline: 10.0%  Histology / follow-up: 100% / 0%  Time between test and surgery: <120 days |
| Einig (2025) [^21^](#_ENREF_21)  Study design: retrospective cohort in which ultrasound model variables were collected at the time of ultrasound examination  Setting: tertiary care  Number of centers: 1  Accrual: Mar 2018 – Nov 2023  Country: Switzerland  Funding: Charity: Griesbach-Hallentstein-Foundation | RMI 1 (200) | *Inclusion:* Patients with a lesion and a histologic report that were systematically scanned in the University Hospital of Basel  *Exclusion:* NR  Type of ultrasound: NR  Experience examiner: mixed levels of experience | Included masses: 453  Age: 47.5 (SD 16.8)  %Postmenopausal: 40.2%  CA-125: median 19.2, range 2 – 12.247  Prevalence of malignancy: 24.5%  Prevalence of malignancy excl borderline: 19.9%  Histology / follow-up: 100% / 0%  Time between test and surgery: NR |
| Elsner Hernández (2024) [^22^](#_ENREF_22)  Study design: retrospective cohort in which ultrasound model variables were collected at the time of ultrasound examination  Setting: tertiary care  Number of centers: 1  Accrual: Jan 2016 – Dec 2021  Country: Spain  Funding: None | ADNEX (5%, 10%, 15%, 20%) | *Inclusion:* Patients with the presence of an adnexal mass, not judged to be a physiological cyst, that attended the ultrasound unit of the gynecologic oncology service at third-level care hospital and underwent surgical excision  *Exclusion:* Surgical removal of the mass more than 180 days after the ultrasound examination, absence of histopathology result  Type of ultrasound: TVE + TAE  Experience examiner: sonographers with >15 years experience | Included masses: 573  Age: mean 49 (SD15)  %Postmenopausal: 44.2%  CA-125: NR  Prevalence of malignancy: 31.9%  Prevalence of malignancy excl borderline: 21.1%  Histology / follow-up: 100% / 0%  Time between test and surgery: <180 days |
| Ertas (2016) [^23^](#_ENREF_23)  Study design: retrospective cohort in which ultrasound model variables were collected at the time of ultrasound examination  Setting: tertiary care  Number of centers: 1  Accrual: Jan 2010 – Feb 2014  Country: Turkey  Funding: NR | RMI 1 (200)  RMI 2 (200)  RMI 3 (200) | *Inclusion:* Patients with adnexal mass that underwent surgery  *Exclusion:* Adnexal mass with pregnancy and patients with unavailable data or absent detailed ultrasonographic findings  Type of ultrasound: TVE + TAE  Experience examiner: expert radiologist | Included masses: 408  Age: range 14 - 87  %Postmenopausal: 28.7%  CA-125: NR  Prevalence of malignancy: 16.4%  Prevalence of malignancy excl borderline: 13.5%  Histology / follow-up: 100% / 0%  Time between test and surgery: NR |
| Giourga *et al* (2024) [^24^](#_ENREF_24)  Study design: retrospective cohort in which ultrasound model variables were collected at the time of ultrasound examination  Setting: tertiary care  Number of centers: 1  Accrual: 2011 – 2023  Country: Greece  Funding: None | Subjective Assessment | *Inclusion:* Patients with ovarian tumors treated in a tertiary gynecological oncology center  *Exclusion:* NR  Type of ultrasound: NR  Experience examiner: 5 individual gynecologists with 6-21 years of experience, all IOTA certified and performing >600 ultrasound examinations per year | Included masses: NR  Age: NR  %Postmenopausal: NR  CA-125: NR  Prevalence of malignancy: 33.3%  Prevalence of malignancy excl borderline: NR  Histology / follow-up: 100% / 0%  Time between test and surgery: <120 days |
| Haliti (2024) [^25^](#_ENREF_25)  Study design: prospective cohort  Setting: tertiary care  Number of centers: 1  Accrual: Jun 2020 – Jun 2022  Country: Kosovo  Funding: None | Simple Rules + Malignant | *Inclusion:* Women aged 13 to 84 years with suspicious adnexal masses that were referred for surgery.  *Exclusion:* Women with metastatic cancer, subserosal myomas, endometriotic cysts, ectopic pregnancy, tubo-ovarian abscesses, and ruptured or twisted cysts  Type of ultrasound: TVE + TAE  Experience examiner: expert examiner | Included masses: 226  Age: mean 45.6  %Postmenopausal: 42.9%  CA-125: NR  Prevalence of malignancy: 29.2%  Prevalence of malignancy excl borderline: 23.0%  Histology / follow-up: 100% / 0%  Time between test and surgery: NR |
| Hartman (2012) [^26^](#_ENREF_26)  Study design: prospective cohort  Setting: tertiary care  Number of centers: 1  Accrual: Jan 2010 – Mar 2011  Country: Brasil  Funding: NR | Simple Rules + Malignant  Simple Rules + Subjective Assessment | *Inclusion:* Women who had been referred to the ovarian pelvic oncology outpatient clinic due to the identification, by clinical and/or imaging examination, of an adnexal tumor  *Exclusion:* Failure to appear for ultrasound evaluation, no detectable tumor on ultrasound evaluation, no surgical indication, lost to follow-up, histopathologic diagnosis of an extrapelvic tumor or an ectopic pregnancy, previous histopathologic diagnosis  Type of ultrasound: TVE + TAE  Experience examiner: SR: by physician with 2 years training in ultrasonography techniques; SA: by a physician in the ultrasound section who had 10 years’ experience in the sonographic evaluation of ovarian tumors, having performed at least 5000 examinations in a referral center. | Included masses: 110  Age: mean 46.8 (SD 14.8)  %Postmenopausal: 42.7%  CA-125: NR  Prevalence of malignancy: 28.2%  Prevalence of malignancy excl borderline: 24.5%  Histology / follow-up: 100% / 0%  Time between test and surgery: <112 days |
| He (2021) [^27^](#_ENREF_27)  Study design: retrospective cohort in which ultrasound model variables were collected at the time of ultrasound examination  Setting: tertiary care  Number of centers: 1  Accrual: Jan 2018 – Dec 2019  Country: China  Funding: None | ADNEX (3%, 5%, 10%, 15%) | *Inclusion:* Patients with at least one adnexal mass that underwent transvaginal or transrectal ultrasonography with an interval between operation and ultrasonography that did not exceed 120 days and no previous history of ovarian cancer.  *Exclusion:* Cysts that were deemed to be clearly physiological and < 3cm in maximum diameter, previous bilateral adnexectomy.  Type of ultrasound: TVE + TAE  Experience examiner: EFSUMB level II with IOTA certification | Included masses: 620  Age: median 44, IQR 34-52  %Postmenopausal: NR  CA-125: median 26, IQR 13-72  Prevalence of malignancy: 33%  Prevalence of malignancy excl borderline: 20%  Histology / follow-up: 100% / 0%  Time between test and surgery: <120 days |
| Hidalgo (2019) [^28^](#_ENREF_28)  Study design: prospective cohort  Setting: tertiary care  Number of centers: 2  Accrual: Sep 2015 – Aug 2017  Country: Spain  Funding: NR | Simple Rules + Malignant | *Inclusion:* Patients ≥ 18 years with at least one adnexal mass persistent for at least 3 months who underwent transvaginal or transrectal ultrasound examination at one of the participating centers.  *Exclusion:*  Pregnancy at the time of the initial ultrasound evaluation or during the follow-up period; presence or history of any neoplastic disease; declining to undergo transvaginal or transrectal ultrasound; and less than 12months of follow-up if expectant management was chosen.  Type of ultrasound: TVE + TAE in case of a large mass  Experience examiner: EFSUMB level II with IOTA certification | Included masses: 283  Age: mean 48.0 (SD 15.7), range 18-90  %Postmenopausal: 41.7%  CA-125: NR  Prevalence of malignancy: 21.9%  Prevalence of malignancy excl borderline: 20.5%  Histology / follow-up: 76% / 24%  Time between test and surgery: <2 months |
| Hidalgo (2021) [^29^](#_ENREF_29)  Study design: prospective cohort  Setting: tertiary care  Number of centers: 1  Accrual: Sep 2015 – Aug 2017  Country: Spain  Funding: NR | LR2 (10%) | *Inclusion:*  Patients ≥ 18 years diagnosed with at least one adnexal mass by transvaginal or transrectal ultrasound, either planned for surgery within 3 months or complete clinical and ultrasound follow-up for at least 12 months.  *Exclusion:*  Pregnancy, patients who could not complete the follow-up visits or had a history of a gynecological neoplastic process  Type of ultrasound: TVE + TAE in case of a large mass  Experience examiner: EFSUMB level II with IOTA certification | Included masses: 100  Age: mean 50.6 (SD 17.4), range 18-87  %Postmenopausal: 42%  CA-125: NR  Prevalence of malignancy: 17%  Prevalence of malignancy excl borderline: 15%  Histology / follow-up: 62% / 38%  Time between test and surgery: <3 months |
| Hiett (2022) [^30^](#_ENREF_30)  Study design: prospective cohort  Setting: tertiary care  Number of centers: 1  Accrual: Mar 2018 – Feb 2021  Country: USA  Funding: NR | ADNEX (5%, 10%, 15%, 20%) | *Inclusion:* (1) Presence of at least one adnexal lesion detected by transvaginal and/or transabdominal ultrasonography; (2) lesion removed surgically and evaluated histopathologically, (3) time interval between ultrasound detection and surgery <180days.  *Exclusion:* NR  Type of ultrasound: TVE + TAE in case of a large mass  Experience examiner: Expert IOTA-certified sonologists each with at least 25 years of experience in gynecological ultrasound | Included masses: 150  Age: NR  %Postmenopausal: 44%  CA-125: NR  Prevalence of malignancy: 26.7%  Prevalence of malignancy excl borderline: 18.7%  Histology / follow-up: 100% / 0%  Time between test and surgery: <180 days |
| Irshad (2013) [^31^](#_ENREF_31)  Study design: prospective cohort  Setting: tertiary care  Number of centers: 1  Accrual: Dec 2007 – Nov 2008  Country: Pakistan  Funding: NR | RMI 1 (250) | *Inclusion:* NR  *Exclusion:* Postmenopausal bleeding, positive family history of breast, endometrial or ovarian carcinoma  Type of ultrasound: NR  Experience examiner: NR | Included masses: 36  Age: mean 58.0 (SD 5.9), range 50-70  %Postmenopausal: 100%  CA-125: mean 60 (SD 29.7), range 20-125  Prevalence of malignancy: 63.9%  Prevalence of malignancy excl borderline: NR  Histology / follow-up: 100% / 0%  Time between test and surgery: NR |
| Jabeen (2015) [^32^](#_ENREF_32)  Study design: prospective cohort  Setting: tertiary care  Number of centers: 1  Accrual: Sep 2008 – Aug 2009  Country: Pakistan  Funding: NR | RMI 1 (200) | *Inclusion:* All patients ≥ 30 years admitted for surgical exploration of an adnexal mass  *Exclusion:* Patients in whom a non-ovarian malignancy was diagnosed and patients with an ovarian malignancy that was already diagnosed  Type of ultrasound: TVE + TAE  Experience examiner: NR | Included masses: 60  Age: mean 50.8  %Postmenopausal: NR  CA-125: NR  Prevalence of malignancy: 30%  Prevalence of malignancy excl borderline: 28.3%  Histology / follow-up: 100% / 0%  Time between test and surgery: NR |
| Javdekar (2015) [^33^](#_ENREF_33)  Study design: prospective cohort  Setting: tertiary care  Number of centers: 1  Accrual: NR  Country: India  Funding: NR | RMI 2 (200, 250) | *Inclusion:* Patients with an adnexal mass scheduled for surgical intervention  *Exclusion:* Patients with functional cysts less than 5 cm, and/or with evident signs of hepatic, peritoneal metastasis, or lung metastasis  Type of ultrasound: TVE + TAE  Experience examiner: NR | Included masses: 58  Age: NR  %Postmenopausal: 41.4%  CA-125: NR  Prevalence of malignancy: 29.3%  Prevalence of malignancy excl borderline: 25.9%  Histology / follow-up: 100% / 0%  Time between test and surgery: NR |
| Janas (2024) [^34^](#_ENREF_34)  Study design: retrospective cohort in which ultrasound model variables were collected at the time of ultrasound examination  Setting: tertiary care  Number of centers: 1  Accrual: 2011 – 2016  Country: Poland  Funding: Government: Ministry of Science and Higher Education's of Poland | RMI 1 (200)  Subjective Assessment | *Inclusion:*Women qualified for surgery due to a pelvic mass  *Exclusion: NR*  Type of ultrasound: TVE  Experience examiner: Single experienced sonographer | Included masses: 456  Age: NR  %Postmenopausal: 50.7%  CA-125: NR  Prevalence of malignancy: 26.8%  Prevalence of malignancy excl borderline: 23.2%  Histology / follow-up: 100% / 0%  Time between test and surgery: NR |
| Jeong (2020) [^35^](#_ENREF_35)  Study design: prospective cohort  Setting: tertiary care  Number of centers: 1  Accrual: Mar 2019 – Jul 2019  Country: South-Korea  Funding: Industry: Samsung Medicine | ADNEX (10%) | *Inclusion:* NR  *Exclusion:* NR  Type of ultrasound: TVE  Experience examiner: One gynaecologic radiologist, with more than 20 years' experience of gynecologic US | Included masses: 54  Age: NR  %Postmenopausal: NR  CA-125: NR  Prevalence of malignancy: 18.5%  Prevalence of malignancy excl borderline: 14.8%  Histology / follow-up: 91.5% / 8.5%  Time between test and surgery: <6 months |
| Kapoor (2024) [^36^](#_ENREF_36)  Study design: prospective cohort  Setting: tertiary care  Number of centers: 1  Accrual: Nov 2021 – Jun 2023  Country: India  Funding: None | Simple Rules + Malignant | *Inclusion:* All newly diagnosed women with adnexal masses planned for surgical management, aged ≥18 years  *Exclusion:* Women who were pregnant, had already received chemotherapy of failed to undergo surgical management within 30 days of ultrasound examination  Type of ultrasound: TVE + TAE in case of a large mass  Experience examiner: Gynaecologic oncologists and radiologists with >15 years of experience | Included masses: 80  Age: mean 37.8 (SD14)  %Postmenopausal: NR  CA-125: NR  Prevalence of malignancy: 45.0%  Prevalence of malignancy excl borderline: 38.8%  Histology / follow-up: 100% / 0%  Time between test and surgery: <30 days |
| Kaya (2020) [^37^](#_ENREF_37)  Study design: prospective cohort  Setting: tertiary care  Number of centers: 1  Accrual: Sep 2014 – Dec 2015  Country: Turkey  Funding: Government: Istanbul Medeniyet University Scientific Research Projects Coordination Unit | RMI 1 (200) | *Inclusion:* Patients scheduled for an operation with a diagnosis of an adnexal mass, non-pregnant, without a biopsy history from the adnexal mass, or undergoing ovulation induction process  *Exclusion:* History of malignancy  Type of ultrasound: TVE  Experience examiner: NR | Included masses: 116  Age: mean 45.9 (SD 11.9), range 21-79  %Postmenopausal: 36.2%  CA-125: NR  Prevalence of malignancy: 24.1%  Prevalence of malignancy excl borderline: 17.2%  Histology / follow-up: 100% / 0%  Time between test and surgery: NR |
| Knafel (2016) [^38^](#_ENREF_38)  Study design: prospective cohort  Setting: tertiary care  Number of centers: 1  Accrual: Jan 2011 – Oct 2012  Country: Poland  Funding: NR | Simple Rules + Malignant  Simple Rules + Subjective Assessment  Subjective Assessment | *Inclusion:* Patients ≥18 years with the presence of an adnexal tumor requiring surgical treatment  *Exclusion:* Pregnancy, withdrawal of consent to participate in the study, lack of histopathology result as an outcome and surgery performed more than 90 days after the initial diagnosis.  Type of ultrasound: TVE + TAE in case of a large mass  Experience examiner: SR: EFSUMB level II; SA: EFSUMG level III | Included masses: 226  Age: mean 47  %Postmenopausal: 36.7%  CA-125: NR  Prevalence of malignancy: 36.3%  Prevalence of malignancy excl borderline: 33.2%  Histology / follow-up: 100% / 0%  Time between test and surgery: <90 days |
| Kougioumtsidou (2025) [^39^](#_ENREF_39)  Study design: retrospective cohort in which ultrasound model variables were collected at the time of ultrasound examination  Setting: tertiary care  Number of centers: 1  Accrual: Jan 2019 – Dec 2022  Country: Greece  Funding: None | LR 2 (10%)  ADNEX (10%) | *Inclusion:* Patients with a sonographic finding of an adnexal lesion, age > 16 years old, who were scheduled to undergo surgery  *Exclusion:* Pregnancy at the time of diagnosis, refusal of transvaginal ultrasound, not undergoing surgery, presence of suspected non-gynecological pelvic masses  Type of ultrasound: TVE + TAE in case of a large mass  Experience examiner: IOTA certified sonographers with at least 5 years of experience in gynaecological ultrasound | Included masses: 136  Age: mean 52.3 (SD 11.4)  %Postmenopausal: 36.7%  CA-125: median 14 (IQR 14)  Prevalence of malignancy: 14.0%  Prevalence of malignancy excl borderline: 11.3%  Histology / follow-up: 100% / 0%  Time between test and surgery: <6 months |
| Lems (2025) [^40^](#_ENREF_40)  Study design: retrospective cohort in which ultrasound model variables were collected at the time of ultrasound examination  Setting: mixed  Number of centers: 4  Accrual: May 2020 – Dec 2021  Country: the Netherlands  Funding: Government: ZonMW | ADNEX (5%, 10%, 20%, 30%, 40%) | *Inclusion:* Patients aged ≥ 18 years who had a first ultrasound assessment for an ovarian tumor using the IOTA ADNEX model without a history of ovarian cancer  *Exclusion:* Non-ovarian pathology; if in case of conservative management follow-up was not performed in accordance with the current guideline  Type of ultrasound: TVE + TAE in case of a large mass  Experience examiner: Varying, all were IOTA certified. Majority EFSUMB level II examiners | Included masses: 363  Age: median 60 (IQR 57 – 71)  %Postmenopausal: 63.1%  CA-125: median 21 (IQR 12 – 55)  Prevalence of malignancy: 21.2%  Prevalence of malignancy excl borderline: 14.6%  Histology / follow-up: 62.5% / 37.6%  Time between test and surgery: median 37 days, IQR 19 – 74 days |
| Liest (2019) [^41^](#_ENREF_41)  Study design: prospective cohort  Setting: mixed  Number of centers: 9  Accrual: Dec 2012 – Jun 2016  Country: Sweden  Funding: Mixed: FORSS (Medical Research Council of Southeast Sweden), County Council of Östergötland, local fund Östgötaregionens cancerfond | RMI 2 (200) | *Inclusion:* Women aged ≥ 18 years with a pelvic mass of probable ovarian origin and scheduled for surgery.  *Exclusion:* NR  Type of ultrasound: NR  Experience examiner: NR | Included masses: 755  Age: NR  %Postmenopausal: 56.0%  CA-125: NR  Prevalence of malignancy: 19.1%  Prevalence of malignancy excl borderline: 13.4%  Histology / follow-up: 100% / 0%  Time between test and surgery: NR |
| Lycke (2018) [^42^](#_ENREF_42)  Study design: prospective cohort  Setting: mixed  Number of centers: 16  Accrual: Sep 2013 – Feb 2016  Country: Sweden  Funding: Industry: Roche Diagnostics Scandinavia | RMI 1 (200) | *Inclusion:* Women ≥18 years, planned for a surgical procedure at a gynecology center for a symptomatic or suspected malignant ovarian cyst or pelvic tumor and able to understand oral and written information in Swedish and to sign the informed consent  *Exclusion:* NR  Type of ultrasound: TVE  Experience examiner: Gynaecologic specialist or gynaecologist in training | Included masses: 611  Age: NR  %Postmenopausal: 57.0%  CA-125: NR  Prevalence of malignancy: 27.2%  Prevalence of malignancy excl borderline: 22.1%  Histology / follow-up: 100% / 0%  Time between test and surgery: NR |
| Mahajan (2020) [^43^](#_ENREF_43)  Study design: prospective cohort  Setting: unclear  Number of centers: 1  Accrual: Jan 2016 – Dec 2016  Country: India  Funding: None | RMI 2 (200) | *Inclusion:* Women with adnexal masses admitted for explorative laparatomy  *Exclusion:* ectopic pregnancy  Type of ultrasound: TVE + TAE in case of a large mass  Experience examiner: NR | Included masses: 50  Age: NR  %Postmenopausal: NR  CA-125: NR    Prevalence of malignancy: 22%  Prevalence of malignancy excl borderline: 22% (borderline tumors were considered benign)  Histology / follow-up: 100% / 0%  Time between test and surgery: NR |
| Malla (2018) [^44^](#_ENREF_44)  Study design: retrospective cohort in which ultrasound model variables were collected at the time of ultrasound examination  Setting: tertiary care  Number of centers: 1  Accrual: Jan 2009 – Jul 2011  Country: India  Funding: None | RMI 2 (200) | *Inclusion:* All women (age >30 years) with an adnexal mass planned for laparotomy  *Exclusion:* Patients with incomplete medical records and those with preoperative histological diagnosis of a malignant ovarian tumor  Type of ultrasound: TVE + TAE  Experience examiner: NR | Included masses: 78  Age: mean 33.8  %Postmenopausal: NR  CA-125: NR  Prevalence of malignancy: 20.5%  Prevalence of malignancy excl borderline: 17.9%  Histology / follow-up: 100% / 0%  Time between test and surgery: NR |
| Manodarshni (2023) [^45^](#_ENREF_45)  Study design: prospective cohort  Setting: tertiary care  Number of centers: 1  Accrual: Oct 2019 – Aug 2021  Country: India  Funding: None | RMI 1 (200) | *Inclusion:* Patients ≥ 18 years of age admitted for elective surgery for adnexal masses  *Exclusion:* Pregnancy, ovarian cancer diagnosis  Type of ultrasound: TVE + TAE  Experience examiner: Department consultants with certified basic training in obstetrics and gynecological ultrasound | Included masses: 121  Age: mean 37, range 18-73  %Postmenopausal: 16.5%  CA-125: mean 88.2 U/mL  Prevalence of malignancy: 9.1%  Prevalence of malignancy excl borderline: 7.4%  Histology / follow-up: 100% / 0%  Time between test and surgery: usually <1 week |
| Meray (2010) [^46^](#_ENREF_46)  Study design: prospective cohort  Setting: tertiary care  Number of centers: 1  Accrual: May 2008 – Aug 2009  Country: Turkey  Funding: NR | RMI 3 (200) | *Inclusion:* Patients scheduled for surgery with the diagnosis of an adnexal mass and written informed consent  *Exclusion:* NR  Type of ultrasound: TVE + TAE  Experience examiner: NR | Included masses: 100  Age: NR  %Postmenopausal: 25%  CA-125: NR  Prevalence of malignancy: 20%  Prevalence of malignancy excl borderline: 16%  Histology / follow-up: 100% / 0%  Time between test and surgery: NR |
| Meys (2017) [^47^](#_ENREF_47)    Study design: retrospective cohort in which ultrasound model variables were collected at the time of ultrasound examination  Setting: tertiary care  Number of centers: 1  Accrual: Jul 2011 – Jul 2015  Country: the Netherlands  Funding: Government: Academic Fund, Maastricht University Medical Center+, and CZ Fund, The Netherlands. | RMI 1 (200)  RMI 2 (200)  RMI 3 (200)  LR2 (10%)  Simple Rules + Malignant  Simple Rules + Subjective Assessment  ADNEX (10%)  Subjective Assessment | *Inclusion:* All patients with adnexal pathology that underwent ultrasound by a single expert examiner  *Exclusion:* No pathology result, pathology result known before ultrasound examination, pathology obtained > 120 days after the ultrasound examination, previous bilateral oophorectomy  Type of ultrasound: TVE + TAE in case of a large mass  Experience examiner: EFSUMB level III | Included masses: 326  Age: NR  %Postmenopausal: 60.7%  CA-125: NR  Prevalence of malignancy: 35.3%  Prevalence of malignancy excl borderline: 27.0%  Histology / follow-up: 100% / 0%  Time between test and surgery: <120 days |
| Mohammed (2014) [^48^](#_ENREF_48)  Study design: retrospective cohort in which ultrasound model variables were collected at the time of ultrasound examination  Setting: tertiary care  Number of centers: 1  Accrual: Jan 2010 – Dec 2012  Country: Qatar  Funding: NR | RMI 3 (200) | *Inclusion:* All patients that underwent surgery for adnexal mass  *Exclusion:* NR  Type of ultrasound: NR  Experience examiner: NR | Included masses: 172  Age: NR  %Postmenopausal: 18%  CA-125: NR  Prevalence of malignancy: 23.8%  Prevalence of malignancy excl borderline: 15.1%  Histology / follow-up: % / %  Time between test and surgery: |
| Moshina (2020) [^49^](#_ENREF_49)  Study design: prospective cohort  Setting: tertiary care  Number of centers: 1  Accrual: Jan 2018 – Jun 2019  Country: India  Funding: None | RMI 1 (200)  RMI 2 (200)  RMI 3 (200) | *Inclusion:* Women scheduled to undergo surgery for an ovarian mass  *Exclusion:* Women with a tubo-ovarian mass  Type of ultrasound: NR  Experience examiner: NR | Included masses: 60  Age: mean 38.85 (SD 13.2)  %Postmenopausal: 20%  CA-125: NR  Prevalence of malignancy: 10%  Prevalence of malignancy excl borderline: 6.7%  Histology / follow-up: 100% / 0%  Time between test and surgery: NR |
| Moszynski (2014) [^50^](#_ENREF_50)  Study design: prospective cohort  Setting: tertiary care  Number of centers: 1  Accrual: 2006 – 2012  Country: Poland  Funding: NR | RMI 1 (200)  LR2 (10%)  Subjective Assessment | *Inclusion:* Women referred to a tertiary center with an adnexal mass, with ultrasonographic appaeareance of a tumor that could not be classified as either 'certainly benign' or 'certainly malignant' based on a subjective ultrasonographic assessment performed by an experienced ultrasonography specialist  *Exclusion:* Tumors designated as 'certainly benign' or 'certainly malignant'  Type of ultrasound: TVE + TAE in case of a large mass  Experience examiner: experienced examiner | Included masses: 268  Age: NR  %Postmenopausal: 34.0%  CA-125: NR  Prevalence of malignancy: 37.7%  Prevalence of malignancy excl borderline: 32.5%  Histology / follow-up: 100% / 0%  Time between test and surgery: 1-3 days |
| Mulder (2021) [^51^](#_ENREF_51)  Study design: retrospective cohort in which ultrasound model variables were collected at the time of ultrasound examination  Setting: tertiary care  Number of centers:  Accrual: Jul 2015 – Feb 2019  Country: the Netherlands  Funding: None | RMI 1 (200)  Simple Rules + Malignant | *Inclusion:* women diagnosed with an ovarian tumor  *Exclusion:* NR  Type of ultrasound: TVE + TAE  Experience examiner: certified IOTA sonographers | Included masses: 168  Age: 57.2, range 18-89  %Postmenopausal: 68.5%  CA-125: NR  Prevalence of malignancy: 29.8%  Prevalence of malignancy excl borderline: 19.6%  Histology / follow-up: 74.4% / 25.6%  Time between test and surgery: NR |
| Ngu (2022) [^52^](#_ENREF_52)  Study design: prospective cohort  Setting: mixed  Number of centers: 4  Accrual: Apr 2018 – Aug 2020  Country: Hong Kong  Funding: Government: Health and Medical Research Fund (HMRF) | RMI 1 (200)  Simple Rules + Malignant  Simple Rules + Subjective Assessment | *Inclusion:* Women ≥18 years who with a pelvic mass on ultrasound, magnetic resonance imaging, computed tomography or positron emission tomography scan and scheduled for surgery  *Exclusion:* Women who declined a transvaginal ultrasound scan, were pregnant, with a previous history of ovarian, peritoneal or fallopian tube cancer or unknown malignancy, and history of bilateral oophorectomy  Type of ultrasound: TVE + TAE in case of a large mass  Experience examiner: RMI and SR: gynaecologists with basic ultrasound training, no experts; SA: expert sonographer with accreditation in Obstetric and Gynecological Ultrasonography and >5 years practice in gynecological ultrasound | Included masses: 690  Age: median 46, range 18-89  %Postmenopausal: 30.7%  CA-125: NR  Prevalence of malignancy: 28.7%  Prevalence of malignancy excl borderline: 23.5%  Histology / follow-up: 100% / 0%  Time between test and surgery: <120 days |
| Niemi (2017) [^53^](#_ENREF_53)  Study design: prospective cohort  Setting: tertiary care  Number of centers: 1  Accrual: Feb 2011 – Nov 2014  Country: Finland  Funding: Government: Finnish Cancer Society and Competitive Research Funding of the Tampere University Hospital | RMI 1 (200)  LR2 (10%)  Simple Rules + Subjective Assessment  Subjective Assessment | *Inclusion:* Women over 50 years of age presenting with an abnormal adnexal mass(es), destined for surgery  *Exclusion:* Overtly benign or malignant looking tumors like unilocular simple ovarian cysts and tumors associated with marked ascites (depth of the greatest pool >10 cm)  Type of ultrasound: TVE  Experience examiner: experienced gynaecologist | Included masses: 98  Age: median 61, range 50-84  %Postmenopausal: NR  CA-125: NR  Prevalence of malignancy: 32.7%  Prevalence of malignancy excl borderline: 25.5%  Histology / follow-up: 100% / 0%  Time between test and surgery: <2 weeks |
| Nunes (2013) [^54^](#_ENREF_54)  Study design: prospective cohort  Setting: tertiary care  Number of centers: 1  Accrual: May 2009 – Jan 2012  Country: UK  Funding: UK Department of Health’s NIHR Biomedical Research Centers | LR2 (10%)  Subjective Assessment | *Inclusion:* All women with ultrasound evidence of an adnexal tumor  *Exclusion:* If surgery was performed beyond 120 days, pregnancy  Type of ultrasound: TVE + TAE in case of large mass  Experience examiner: LR2: EFSUMB level II; SA: expert ultrasound operator | Included masses: 292  Age: mean 51, range 16-91  %Postmenopausal: 46.9%  CA-125: NR  Prevalence of malignancy: 45.2%  Prevalence of malignancy excl borderline: 39.4%  Histology / follow-up: 100% / 0%  Time between test and surgery: <120 days |
| Nunes (2014) [^55^](#_ENREF_55)  Study design: prospective cohort  Setting: tertiary care  Number of centers: 1  Accrual: May 2009 – Jan 2012  Country: UK  Funding: UK Department of Health’s NIHR Biomedical Research Centers | Simple Rules + Malignant  Simple Rules + Subjective Assessment | *Inclusion:* All women diagnosed with an adnexal tumor  *Exclusion:* Pregnant women, those unable to undergo transvaginal scan and those whose surgery date exceeded 120 days from the date of the ultrasound scan  Type of ultrasound: TVE + TAE  Experience examiner: SR: EFSUMB level II; SA: expert ultrasound operator | Included masses: 303  Age: mean 50, range 16-91  %Postmenopausal: 46.5%  CA-125: NR  Prevalence of malignancy: 44.6%  Prevalence of malignancy excl borderline: 38.3%  Histology / follow-up: 100% / 0%  Time between test and surgery: <120 days |
| Nunes (2018) [^56^](#_ENREF_56)  Study design: prospective cohort  Setting: tertiary care  Number of centers: 1  Accrual: May 2009 – Jan 2012  Country: UK  Funding: UK Department of Health’s NIHR Biomedical Research Centers | LR2 (10%)  Subjective Assessment | *Inclusion:* Women with evidence of adnexal tumors on ultrasound scan  *Exclusion:* Women with unilocular, anechoic cysts of mean diameter <2 cm, pregnant women and those unable to undergo a transvaginal scan  Type of ultrasound: TVE + TAE in case of large mass  Experience examiner: SR: EFSUMB level II; SA: expert ultrasound operator | Included masses: 544  Age: mean 50, range 16-91  %Postmenopausal: 43.6%  CA-125: NR  Prevalence of malignancy: 28%  Prevalence of malignancy excl borderline: 23.7%  Histology / follow-up: 62.9% / 27.1%  Time between test and surgery: NR |
| Pascual (2024) [^57^](#_ENREF_57)  Study design: prospective cohort  Setting: secondary care  Number of centers: 1  Accural: Jun 2012 – Sep 2016  Country: Spain  Funding: Reserach Foundation Flanders projects and Internal Funds KU Leuven | ADNEX (5%, 10%, 20%, 30%, 40%) | *Inclusion:* Women of 18 years and older diagnosed with a new adnexal mass  *Exclusion:* Cysts deemed to be physiological if the largest diameter was less than 3 cm  Type of ultrasound: TVE + TAE in case of large mass  Experience examiner: EFSUMB level II examiners | Included masses: 2039  Age: mean 39, IQR 33 – 46  %Postmenopausal: 12.2%  CA-125: mean 12, IQR 8 – 27  Prevalence of malignancy: 3.0%  Prevalence of malignancy excl borderline: NR  Histology / follow-up: 15.5% / 84.5%  Time between test and surgery: NR |
| PecesRama (2015) [^58^](#_ENREF_58)  Study design: prospective cohort  Setting: tertiary care  Number of centers: 1  Accrual: Nov 2013 – Jan 2014  Country: Spain  Funding: NR | Simple Rules + Subjective Assessment  Subjective Assessment | *Inclusion:* Women with a persistent adnexal mass that had been referred to a tertiary center  *Exclusion:* Women under 18 years of age, pregnant women and women unable to undergo transvaginal ultrasound  Type of ultrasound: TVE + TAE in case of large mass  Experience examiner: SR: inexperienced sonographer (3^rd^ years gyn-obs trainee); SR: expert sonographer | Included masses: 81  Age: mean 42, range 18-87  %Postmenopausal: 27.2%  CA-125: NR  Prevalence of malignancy: 9.9%  Prevalence of malignancy excl borderline: 7.4%  Histology / follow-up: 37% / 67%  Time between test and surgery: <120 days |
| Pereira (2020) [^59^](#_ENREF_59)  Study design: prospective cohort  Setting: tertiary care  Number of centers: 1  Accrual: Feb 2014 – Feb 2016  Country: Brasil  Funding: Government: São Paulo Research Foundation; Conselho Nacional de Desenvolvimento Científico e Tecnológico | Simple Rules + Malignant  Simple Rules + Subjective Assessment | *Inclusion:* Women referred to a tertiary center with an adnexal mass  *Exclusion:* Insufficient ultrasound data to apply Simple Rules  Type of ultrasound: TVE + TAE  Experience examiner: EFSUMB level III | Included masses: 171  Age: mean 50.8  %Postmenopausal: 50.3%  CA-125:  Prevalence of malignancy: 32.2%  Prevalence of malignancy excl borderline: 25.7%  Histology / follow-up: 70.2% / 29.8%  Time between test and surgery: NR |
| Phinyo (2021) [^60^](#_ENREF_60)  Study design: prospective cohort  Setting: tertiary care  Number of centers: 1  Accrual: Apr 2010 – Mar 2018  Country: Thailand  Funding: Government: Chiang Mai University Research Fund | Simple Rules + Malignant | *Inclusion:* Women with adnexal masses diagnosed by either pelvic ultrasonographic examination or by vaginal examination, scheduled for surgery, without a known diagnosis of the mass before surgery  *Exclusion:* Patients whose mass was surgically removed 24h after ultrasonographic examination and patients without preoperative CA-125  Type of ultrasound: TVE  Experience examiner: Non-expert sonographers | Included masses: 479  Age: mean 42.1  %Postmenopausal: 24%  CA-125: NR  Prevalence of malignancy: 30.3%  Prevalence of malignancy excl borderline: 27.1%  Histology / follow-up: 100% / 0%  Time between test and surgery: <24h |
| Piovano (2017) [^61^](#_ENREF_61)  Study design: prospective cohort  Setting: tertiary care  Number of centers: 1  Accrual: Feb 2013 – Jan 2015  Country: Italy  Funding: Government: University of Turin | Simple Rules + Subjective Assessment | *Inclusion:* Women ≥18 years with a clinical diagnosis of an adnexal mass  *Exclusion:* Women with previous malignant disease, any previous surgery for an adnexal mass or in-situ uterine cervical carcinoma, pregnancy, refusal of TVS or no surgical removal of the mass within 30 days after study enrollment  Type of ultrasound: TVE + TAE in case of a large mass  Experience examiner: SR: trainee; SA; expert, EFSUMB level III | Included masses: 391  Age: median 47, range 18-86  %Postmenopausal: 43.5%  CA-125: NR  Prevalence of malignancy: 21.5%  Prevalence of malignancy excl borderline: 15.9%  Histology / follow-up: 100% / 0%  Time between test and surgery: <30 days |
| Poonyakanok (2021) [^62^](#_ENREF_62)  Study design: prospective cohort  Setting: tertiary care  Number of centers: 1  Accrual: May 2018 – May 2019  Country: Thailand  Funding: Government: Siriraj Research Development Fund | ADNEX (5%, 10%, 15%, 20%) | *Inclusion:* Women who presented with an adnexal mass and were scheduled for surgical intervention  *Exclusion:* Patients who had a previous history of ovarian cancer or had undergone a bilateral oophorectomy; those who turned out to have an uterine lesion or abscess instead of an adnexal lesion, incomplete ultrasonography information, operated in another hospital, surgical intervention >120 days after the ultrasonography  Type of ultrasound: TVE + TAE in case of a large mass  Experience examiner: Gynaecologists with 5 - 18 years experience | Included masses: 357  Age: median 43, IQR 35-53  %Postmenopausal: 71.4%  CA-125: NR  Prevalence of malignancy: 17.1%  Prevalence of malignancy excl borderline: 14.0%  Histology / follow-up: 100% / 0%  Time between test and surgery: <120 days |
| Poonyakanok (2023) [^63^](#_ENREF_63)  Study design: prospective cohort  Setting: tertiary care  Number of centers: 1  Accrual: May 2018 – May 2019  Country: Thailand  Funding: Government: Siriraj Research Development Fund | RMI 2 (200) | *Inclusion:* Women who presented with an adnexal mass and were scheduled for surgical intervention  *Exclusion:* Patients who had a previous history of ovarian cancer or had undergone a bilateral oophorectomy; those who turned out to have an uterine lesion or abscess instead of an adnexal lesion, incomplete ultrasonography information, operated in another hospital, surgical intervention >120 days after the ultrasonography  Type of ultrasound: TVE + TAE in case of a large mass  Experience examiner: Gynaecologists with 5 - 18 years experience | Included masses: 357  Age: mean 44, range 13-85  %Postmenopausal: 71.4%  CA-125: NR  Prevalence of malignancy: 17.1%  Prevalence of malignancy excl borderline: 14.0%  Histology / follow-up: 100% / 0%  Time between test and surgery: <120 days |
| Richards (2015) [^64^](#_ENREF_64)  Study design: prospective cohort  Setting: tertiary care  Number of centers: 1  Accrual: Oct 2012 – Mar 2014  Country: Australia  Funding: Industry: Abbott Diagnostics Australasia Pty LTD | RMI 1 (200) | *Inclusion:* Women who were undergoing primary surgery for a complex pelvic mass, presumed to be arising from the ovary  *Exclusion:* Women with a known malignancy undergoing neoadjuvant chemotherapy prior to interval debulking surgery for advanced ovarian/primary peritoneal malignancy  Type of ultrasound: NR  Experience examiner: NR | Included masses: 50  Age: NR  %Postmenopausal: 58%  CA-125: NR  Prevalence of malignancy: 40.0%  Prevalence of malignancy excl borderline: 32.0%  Histology / follow-up: 100% / 0%  Time between test and surgery: NR |
| RuizdeGauna (2015) [^65^](#_ENREF_65)  Study design: prospective cohort  Setting: mixed  Number of centers: 2  Accrual: Jun 2012 – Dec 2013  Country: Spain  Funding: NR | Simple Rules + Malignant  Simple Rules + Subjective Assessment | *Inclusion:* All consecutive women diagnosed as having a persistent adnexal mass  *Exclusion:* Pregnant women, women with spontaneous resolution of the mass, masses removed surgically in another center  Type of ultrasound: TVE + TAE in case of a large mass  Experience examiner: center A: expert with >20 years experience in gynecological ultrasound; center B: 3th year Ob/gyn trainee who had a three-month period of real-time ultrasound training under the supervision of an expert examiner | Included masses: 247  Age: mean 43.6 (SD 14.1), range 14-83  %Postmenopausal: 27.9%  CA-125: NR  Prevalence of malignancy: 18.6%  Prevalence of malignancy excl borderline: NR%  Histology / follow-up: 100% / 0%  Time between test and surgery: NR |
| Sayasneh (2013) [^66^](#_ENREF_66)  Study design: prospective cohort  Setting: mixed  Number of centers: 3  Accrual: Sep 2010 – Feb 2015  Country: UK  Funding: Government:  Imperial College London | RMI 1 (200)  LR2 (10%)  Simple Rules + Malignant  Simple Rules + Subjective Assessment  Subjective Assessment | *Inclusion:* Patients presenting with at least one adnexal mass who underwent transvaginal ultrasonography at one of the participating centers  *Exclusion:* Pregnancy, patients examined by a consultant, refusal of transvaginal ultrasonography, cytology rather than histology as an outcome and failure to undergo surgery within 120 days of the ultrasound examination  Type of ultrasound: TVE + TAE in case of a large mass  Experience examiner: EFSUMB level II | Included masses: 255  Age: mean 46, 95% CI (34 – 57)  %Postmenopausal: 35.3%  CA-125: NR  Prevalence of malignancy: 29%  Prevalence of malignancy excl borderline: 22%  Histology / follow-up: 100% / 0%  Time between test and surgery: <120 days |
| Sayasneh (2016) [^67^](#_ENREF_67)  Study design: retrospective cohort in which ultrasound model variables were collected at the time of ultrasound examination  Setting: tertiary care  Number of centers: 3  Accrual: Sep 2010 – Feb 2015  Country: United Kingdom  Funding: Government: FWO Grants and Internal Funds KU Leuven, National Insititute for Health Research (NIHR) Biomedical Research Center) | ADNEX (1%; 3%; 5%; 10%; 15%; 20%; 30%) | *Inclusion:* Patients presenting with at least one adnexal mass who underwent transvaginal ultrasonography  *Exclusion:* Pregnancy, patients examined by a consultant, refusal of transvaginal ultrasonography, cytology rather than histology as an outcome and failure to undergo surgery within 120 days of the ultrasound examination  Type of ultrasound: TVE + TAE in case of a large mass  Experience examiner: EFSUMB level II | Included masses: 610  Age: median 47  %Postmenopausal: 42,3%  CA-125: NR  Prevalence of malignancy: 29,8%  Prevalence of malignancy excl borderline: 23.0%  Histology / follow-up: 100% / 0%  Time between test and surgery: <120 days |
| Shimada (2018) [^68^](#_ENREF_68)  Study design: prospective cohort  Setting: mixed  Number of centers: 2  Accrual: Feb 2014 – Dec 2015  Country: Japan  Funding: None | LR2 (10%) | *Inclusion:* Patients presenting with at least one adnexal mass, and having undergone an US examination by a principal investigator and MRI imaging at one of the participating centers  *Exclusion:* Patients who were pregnant, who refused to undergo transvaginal ultrasound or MRI, who failed to undergo surgical removal of the mass within 120 days of the US examination  Type of ultrasound: TVE + TAE in case of a large mass  Experience examiner: two gynaecologists, of one certified | Included masses: 265  Age: median 41, range 19-88  %Postmenopausal: NR  CA-125: NR  Prevalence of malignancy: 20.4%  Prevalence of malignancy excl borderline: 16.2%  Histology / follow-up: 100% / 0%  Time between test and surgery: <120 days |
| Silvestre (2015) [^69^](#_ENREF_69)  Study design: prospective cohort  Setting: tertiary care  Number of centers: 1  Accrual: Sep 2008 – Dec 2010  Country: Brasil  Funding: NR | Simple Rules + Malignant | *Inclusion:* Women that were scheduled for surgery to remove an adnexal mass  *Exclusion:* NR  Type of ultrasound: TVE  Experience examiner: NR | Included masses: 75  Age: NR  %Postmenopausal: NR  CA-125: NR  Prevalence of malignancy: 42.7%  Prevalence of malignancy excl borderline: 36.0%  Histology / follow-up: 100% / 0%  Time between test and surgery: <7 days |
| Solanki (2020) [^70^](#_ENREF_70)  Study design: prospective cohort  Setting: tertiary care  Number of centers: 1  Accrual: NR, “18 months”  Country: India  Funding: None | Simple Rules + Malignant | *Inclusion:* Women with adnexal masses planned for surgery  *Exclusion:* Young girls who have not attained menarche, pregnant women, already diagnosed cancer of the ovary by histopathology  Type of ultrasound: TVE + TAE  Experience examiner: three gynaecologists, of which one with IOTA certification and two senior gynaecologists with >8 years experience | Included masses: 174  Age: mean 40.9, range 19-63  %Postmenopausal: 19.5%  CA-125: NR  Prevalence of malignancy: 17.2%  Prevalence of malignancy excl borderline: 16.1%  Histology / follow-up: 100% / 0%  Time between test and surgery: NR |
| Spagnol (2023) [^71^](#_ENREF_71)  Study design: retrospective cohort in which ultrasound model variables were collected at the time of ultrasound examination  Setting: tertiary care  Number of centers: 1  Accrual: Jan 2018 – Dec 2021  Country: Italy  Funding: None | Simple Rules + Malignant | *Inclusion* Patients who underwent a surgical procedure for an adnexal mass, with pre-operative description of the adnexal mass with IOTA definitions, results of the ADNEX model, O-RADS and SRR calculated before surgery and available histopathology result  *Exclusion:* Carrier of genetic mutations/syndromes, previous confirmed diagnosis of ovarian cancer, pregnancy, > 3 months between ultrasound and Ca-125 and surgical procedure  Type of ultrasound: TVE + TAE in case of a large mass  Experience examiner: IOTA-certified gynaecologic specialists or a gynaecologist in training under supervision | Included masses: 514  Age: median 51, IQR 41-64  %Postmenopausal: 49.4%  CA-125: NR  Prevalence of malignancy: 22.2%  Prevalence of malignancy excl borderline: 17.3%  Histology / follow-up: 100% / 0%  Time between test and surgery: <3 months |
| Spagnol (2024) [^72^](#_ENREF_72)  Study design: retrospective cohort in which ultrasound model variables were collected at the time of ultrasound examination  Setting: tertiary care  Number of centers: 1  Accrual: Jan 2015 – Dec 2020  Country: Italy  Funding: None | RMI 1 (200)  ADNEX (5%, 10%, 20%, 30%, 40%) | *Inclusion:* Patients with diagnosis of an adenxal mass who underwent surgery and underwent TV-US and had CA-125 and HE4 levels measured within a maximum of three months prior to the surgical procedure  *Exclusion:* Age less than 18 years; ongoing pregnancy; previously confirmed diagnosis of malignancy; no consent for the use of data  Type of ultrasound: TVE + TAE in case of a large mass  Experience examiner: experienced gynaecologist or gynaecologist in training under supervision | Included masses: 581  Age: median 49, IQR 39 – 61  %Postmenopausal: 46.1%  CA-125: NR  Prevalence of malignancy: 17.2%  Prevalence of malignancy excl borderline: 10.7%  Histology / follow-up: 100% / 0%  Time between test and surgery: <3 months |
| Stukan (2019) [^73^](#_ENREF_73)  Study design: prospective cohort  Setting: tertiary care  Number of centers: 1  Accrual: Jun 2016 – Jun 2017  Country: Poland  Funding: None | RMI 3 (200)  LR2 (10%)  ADNEX (10%)  Subjective Assessment | *Inclusion:* Patients with ovarian tumors that underwent surgery within 60 days of the ultrasound examination  *Exclusion:* A prior bilateral oophorectomy, pregnancy, refusal to undergo ultrasonography  Type of ultrasound: TVE + TAE  Experience examiner: 8-13 years of experience | Included masses: 100  Age: median 51, IQR 40 - 62  %Postmenopausal: 51%  CA-125: median 65, IQR 24-447  Prevalence of malignancy: 52%  Prevalence of malignancy excl borderline: NR  Histology / follow-up: 100% / 0%  Time between test and surgery: <60 days |
| Sundar (2024) [^74^](#_ENREF_74)  Study design: prospective cohort  Setting: mixed  Number of centers: 23  Accrual: Jul 2015 – Nov 2018  Country: UK  Funding: Government: UK National Institute for Health and Care Research | RMI 1 (200, 250)  Simple Rules + Malignant  ADNEX (3%, 10%) | *Inclusion:* Newly presenting postmenopausal female patients who had been referred to a hospital with non-specific symptoms and raised CA-125 values or abnormal ultrasound findings, as interpreted by the primary care practicioner  *Exclusion:* Pregnancy, declining a transvaginal scan, active non-ovarian malignancy, or previous ovarian malignancy  Type of ultrasound: TVE + TAE  Experience examiner: Mainly EFSUMB level II ultrasound examiners; all were IOTA certified and passed the quality assessment by the IOTA team | Included masses: 1242  Age: median 65.3, IQR 57,9-73.3  %Postmenopausal: 100%  CA-125: NR  Prevalence of malignancy: 29.8%  Prevalence of malignancy excl borderline: 24.5%  Histology / follow-up: unknown  Time between test and surgery: NR |
| Szubert (2016) [^75^](#_ENREF_75)  Study design: retrospective cohort in which ultrasound model variables were collected at the time of ultrasound examination  Setting: tertiary care  Number of centers: 2  Accrual: Dec 2012 – Apr 2015  Country: Poland  Funding: None | ADNEX (10%)  Subjective Assessment | *Inclusion:* Patients that needed surgery for an ovarian tumor that were evaluated with ultrasonography by one of the PI's  *Exclusion:* NR  Type of ultrasound: TVE + TAE  Experience examiner: Two EFSUMB level II examiners and one EFSUMB level III examiner | Included masses: 204  Age: median 46, range 15-84  %Postmenopausal: 32.4%  CA-125: median 40, range 4-4909  Prevalence of malignancy: 31.8%  Prevalence of malignancy excl borderline: 26.9%  Histology / follow-up: 100% / 0%  Time between test and surgery: <1-5 days |
| Tangjanyatham 2025 [^76^](#_ENREF_76)  Study design: prospective cohort  Setting: tertiary care  Number of centers: 1  Accrual: Mar 2023 – Nov 2024  Country: Thailand  Funding: Navamindradhiraj University Research Fund | ADNEX (10%) | *Inclusion:* Patients aged 18-90 years with presence of adnexal masses identified by ultrasound and scheduled for surgery with the interval between ultrasound and surgery not exceeding 30 days  *Exclusion:* History or presence of other malignancies, current pregnancy and lack of a histopathological report  Type of ultrasound: TVE + TAE in case of a large mass  Experience examiner: Experienced gynaecologists (more than 5 years) or gynecology resident trainees under supervision | Included masses: 110  Age: mean 50.1  %Postmenopausal: 50.9%  CA-125: mean 173.4  Prevalence of malignancy: 33.6%  Prevalence of malignancy excl borderline: 28.2%  Histology / follow-up: 100% / 0%  Time between test and surgery: <30 days |
| Tantipalakorn (2014) [^77^](#_ENREF_77)  Study design: prospective cohort  Setting: tertiary care  Number of centers: 1  Accrual: Ap 2007 – Mrt 2012  Country: Thailand  Funding: Government: Faculty of Medicine Research Fund of Chiang Mai University; National Research University Project | Simple Rules + Malignant | *Inclusion:* Women scheduled for surgery because of the detection of an adnexal mass either by pelvic examination of previous ultrasonography or both  *Exclusion:* Known diagnoses of adnexal masses, such as ovarian cancers scheduled for second look operation, or endometrioma diagnosed by previous laparoscopy, etc, and patients undergoing surgery beyond 24 hours after ultrasound examination  Type of ultrasound: TVE + TAE  Experience examiner: NR | Included masses: 376  Age: mean 42.4 (SD 16.2), range 13-82  %Postmenopausal: 32.2%  CA-125: NR  Prevalence of malignancy: 32.9%  Prevalence of malignancy excl borderline: NR  Histology / follow-up: 100% / 0%  Time between test and surgery: <24 hours |
| Terzic (2011) [^78^](#_ENREF_78)  Study design: prospective cohort  Setting: secondary care  Number of centers: 1  Accrual: Jan 2010 – Jun 2010  Country: Serbia  Funding: NR | RMI 1 (200) | *Inclusion:* Patients with adnexal tumors hospitalized between Jan 2010 - Jun 2010  *Exclusion:* NR  Type of ultrasound: NR  Experience examiner: NR | Included masses: 81  Age: mean 43.2, range 16-82  %Postmenopausal: 37.0%  CA-125: range 2-13623  Prevalence of malignancy: 37.0%  Prevalence of malignancy excl borderline: 27.2%  Histology / follow-up: 100% / 0%  Time between test and surgery: NR |
| Terzic (2013) [^79^](#_ENREF_79)  Study design: prospective cohort  Setting: secondary care  Number of centers: 1  Accrual: Jul 2010 – Dec 20100  Country: Serbia  Funding: Government: Ministry of Science and Technological development of the Republic of Serbia | RMI 1 (250) | *Inclusion:* Patients who underwent surgery for an adnexal tumor  *Exclusion: NR*  Type of ultrasound: NR  Experience examiner: Expert | Included masses: 540  Age: mean 43.4 (SD 16.8), range 18-82  %Postmenopausal: 34.1%  CA-125: mean 68.3, range 4-963  Prevalence of malignancy: 19.4%  Prevalence of malignancy excl borderline: 15.7%  Histology / follow-up: 100% / 0%  Time between test and surgery: NR |
| Testa (2014) [^80^](#_ENREF_80)  Study design: prospective cohort  Setting: mixed  Number of centers: 18  Accrual: Oct 2009 – May 2012  Country: Belgium, Sweden, Italy, Poland, Spain and the Czech Republic  Funding: Government: FWO Grants and Internal Funds KU Leuven, IWT, Swedish Medical Research Council, Imperial Healthcare NHS Trust NIHR Biomedical Research Center | RMI 1 (200)  LR2 (10%)  Simple Rules + Malignant  Simple Rules + Subjective Assessment  Subjective Assessment | *Inclusion:* Patients presenting with at least one adnexal mass (ovarian, para-ovarian or tubal), who underwent transvaginal ultrasound examination by a principal investigator at one of the participating centers and who were selected for surgical intervention by the managing clinician  *Exclusion:* Surgical removal of the mass >120 days after the ultrasound examination, pregnancy at scan, data inconsistencies that persisted after final manual data checks  Type of ultrasound: TVE + TAE in case of a large mass  Experience examiner: EFSUMB level III expert examiners | Included masses: 2403  Age: NR  %Postmenopausal: 43.7%  CA-125: NR  Prevalence of malignancy: 40.8%  Prevalence of malignancy excl borderline: 34.4%  Histology / follow-up: 100% / 0%  Time between test and surgery: <120 days |
| Timmerman (1999) [^81^](#_ENREF_81)  Study design: prospective cohort  Setting: tertiary care  Number of centers: 1  Accrual: Aug 1994 – Aug 1996  Country: Belgium  Funding: NR | RMI 1 (200) | *Inclusion:* All patients referred for a preoperative assessment of an adnexal mass  *Exclusion:* Patients without preoperatively measured serum CA-125  Type of ultrasound: TVE  Experience examiner: NR | Included masses: 173  Age: mean 53, range 22-93  %Postmenopausal: 52.4%  CA-125: NR  Prevalence of malignancy: 28.3%  Prevalence of malignancy excl borderline: 25.4%  Histology / follow-up: 100% / 0%  Time between test and surgery: NR |
| Timmerman (1999) [^82^](#_ENREF_82)  Study design: prospective cohort  Setting: tertiary care  Number of centers: 1  Accrual: Aug 1995 – Jun 1997  Country: Belgium  Funding: NR | Subjective Assessment | *Inclusion:* Patients with an extrauterine pevlic tumor who were referred to a single institution, gave informed consent to undergo transvaginal color Doppler and had an adnexal mass removed at surgery  *Exclusion:* NR  Type of ultrasound: TVE + TAE  Experience examiner: EFSUMB level II | Included masses: 300  Age: range 18-93  %Postmenopausal: 44.7%  CA-125: NR  Prevalence of malignancy: 27.7%  Prevalence of malignancy excl borderline: 25.0%  Histology / follow-up: 100% / 0%  Time between test and surgery: NR |
| Timmerman (2011) [^83^](#_ENREF_83)  Study design: prospective cohort  Setting: mixed  Number of centers: 19  Accrual: Nov 2005 – Oct 2007  Country: Sweden, Belgium, Italy, Poland, UK, the Czech Republic, China, Canada  Funding: Government: Research Council KUL, (2) Flemish Government, (3) Belgian Federal Science Policy Office, (4) EU-RTD, (5) Swedish Medical Research Council | LR2 (10%)  Simple Rules + Malignant  Simple Rules + Subjective Assessment | *Inclusion:* Patients presenting with at least one adnexal mass who had an ultrasound examination by a principal investigator at one of the participating centers  *Exclusion:* Patients who were pregnant, refused transvaginal ultrasonography and those who did not have surgical removal of the mass within 120 days after the ultrasound examination  Type of ultrasound: TVE + TAE in case of a large mass  Experience examiner: EFSUMB level III expert examiners | Included masses: 1938  Age: mean 46, range 11-94  %Postmenopausal: 38.3%  CA-125: NR  Prevalence of malignancy: 28%  Prevalence of malignancy excl borderline: 22.2%  Histology / follow-up: 100% / 0%  Time between test and surgery: <120 days |
| Tongsong (2007) [^84^](#_ENREF_84)  Study design: prospective cohort  Setting: tertiary care  Number of centers: 1  Accrual: Nov 2003 – Nov 2004  Country: Thailand  Funding: National Center of Biotechnology and Genetic Engineering, Thailand; Faculty of Tropical Medicine, Mahidol University, Bangkok, Thailand | Subjective Assessment | *Inclusion:* All patients scheduled for elective surgery due to the clinical diagnosis of an ovarian tumor  *Exclusion:* NR  Type of ultrasound: TVE + TAE  Experience examiner: NR | Included masses: 174  Age: mean 46.9, range 14-82  %Postmenopausal: NR  CA-125: NR  Prevalence of malignancy: 37.9%  Prevalence of malignancy excl borderline: NR  Histology / follow-up: 100% / 0%  Time between test and surgery: <72 hours |
| Ulusoy (2007) [^85^](#_ENREF_85)  Study design: prospective cohort  Setting: tertiary care  Number of centers: 1  Accrual: Sep 2002 – Nov 2004  Country: Turkey  Funding: NR | RMI 1 (200, 250) | *Inclusion:* Patients undergoing surgery for an adnexal mass  *Exclusion:* known ovarian malignancy and pregnant patients  Type of ultrasound: TVE + TAE  Experience examiner: gynecological oncologists | Included masses: 296  Age: mean 43.8  %Postmenopausal: 37.2%  CA-125: range 1-5000  Prevalence of malignancy: 35.8%  Prevalence of malignancy excl borderline: 30.7%  Histology / follow-up: 100% / 0%  Time between test and surgery: NR |
| Valentin (1999) [^86^](#_ENREF_86)  Study design: prospective cohort  Setting: tertiary care  Number of centers: 1  Accrual: NR  Country: Sweden  Funding: Government: Malmö General Hospital Cancer Foundation, the Swedish Medical Research Council | Subjective Assessment | *Inclusion:* Women scheduled for laparotomy or laparoscopic surgery because of a pelvic mass judged clinically to be of adnexal origin  *Exclusion:* No surgery, no histopathological diagnosis, normal ovaries  Type of ultrasound: TVE + TAE  Experience examiner: EFSUMB level III expert examiner | Included masses: 173  Age: NR  %Postmenopausal: 40.5  CA-125: NR  Prevalence of malignancy: 13.9%  Prevalence of malignancy excl borderline: 11.0%  Histology / follow-up: 100% / 0%  Time between test and surgery: <8 days |
| Valentin (2001) [^87^](#_ENREF_87)  Study design: prospective cohort  Setting: tertiary care  Number of centers: 2  Accrual: NR  Country: Sweden  Funding: Government: Malmö General Hospital Cancer Foundation, Swedish Medical Research Council | Subjective Assessment | *Inclusion:* Women scheduled for laparotomy or laparoscopic surgery because of a pelvic mass judged clinically to be of adnexal origin  *Exclusion:* No surgery, no histopathological diagnosis  Type of ultrasound: TVE + TAE  Experience examiner: EFSUMB level III expert examiner | Included masses: 136  Age: NR  %Postmenopausal: NR  CA-125: NR  Prevalence of malignancy: 26.5%  Prevalence of malignancy excl borderline: NR  Histology / follow-up: 100% / 0%  Time between test and surgery: <2 weeks |
| Van Calster (2007) [^88^](#_ENREF_88)  Study design: prospective cohort  Setting: mixed  Number of centers: 9  Accrual: Jun 1999 – Jun 2002  Country: Sweden, Belgium, UK, Italy, France  Funding: Government: Research council of the Katholieke Universiteit Leuven, Belgium, Belgian Federal Science Policy Office, the EU, Swedish Medical Research Council | Subjective Assessment | *Inclusion:* Women with at least one adnexal mass that required surgery, not to be a physiological cyst, who were examined with transvaginal ultrasound by a principal investigator and later selected for surgical intervention  *Exclusion:* No surgery, no histopathological diagnosis  Type of ultrasound: TVE + TAE in case of a large mass  Experience examiner: EFSUMB level III expert examiner | Included masses: 809  Age: mean 48.8 (SD 15.6)  %Postmenopausal: 45%  CA-125: NR  Prevalence of malignancy: 29.9%  Prevalence of malignancy excl borderline: 23.5%  Histology / follow-up: 100% / 0%  Time between test and surgery: <120 days |
| Van Calster (2020) [^89^](#_ENREF_89)  Study design: prospective cohort  Setting: mixed  Number of centers: 17  Accrual: Jan 2012 – Mrt 2015  Country: Sweden, Italy, Greece, Belgium, Poland, the Czech Republic, Portugal, UK, Spain, China, Egypt, France, United States, Austria  Funding: Government: Research Foundation Flanders and KU Leuven | RMI 1 (200, 250)  LR2 (10%)  ADNEX (1%, 3%, 5%, 10%, 15%, 20%, 25%, 30%, 40%, 50%)  Subjective Assessment | *Inclusion:* ≥18 years at recruitment and presenting with at least one adnexal mass (ovarian, para-ovarian, or tubal) on ultrasound examination  *Exclusion:* Physiological lesions if the largest diameter was less than 3 cm, refusal to provide informed consent, or withdrawal of informed consent, adnexal mass already in follow-up before the start of the study.  Type of ultrasound: TVE + TAE  Experience examiner: all were IOTA certified | Included masses: 4905  Age: median 48, range 18-98  %Postmenopausal: 43.9%  CA-125: mean 318  Prevalence of malignancy: 21.1%  Prevalence of malignancy excl borderline: NR  Histology / follow-up: NR  Time between test and surgery: <120 days |
| Van den Akker (2011) [^90^](#_ENREF_90)  Study design: prospective cohort  Setting: mixed  Number of centers: 11  Accrual: Jan 205 – Sep 2009  Country: the Netherlands  Funding: NR | RMI 3 (200, 250) | *Inclusion:* Women admitted for a surgical procedure for an adnexal mass  *Exclusion: NR*  Type of ultrasound: TVE + TAE in case of a large mass  Experience examiner: experienced gynecological oncologists, general oncologists and registrars in gynecaology | Included masses: 643  Age: NR  %Postmenopausal: 62.2%  CA-125: NR  Prevalence of malignancy: 27.1%  Prevalence of malignancy excl borderline: 15.7%  Histology / follow-up: 100% / 0%  Time between test and surgery: NR |
| Van Holsbeke (2007) [^91^](#_ENREF_91)  Study design: prospective cohort  Setting: mixed  Number of centers: 9  Accrual: Jun 1999 – Jun 2002  Country: Sweden, Belgium, UK, Italy, France  Funding: Government: Research council of the Katholieke Universiteit Leuven, Belgium, Belgian Federal Science Policy Office, the EU, Swedish Medical Research Council | RMI 1 (200)  RMI 2 (200) | *Inclusion:* Patients presenting with at least one overt persistent adnexal mass who were assessed by a principal investigator at one of the participating centers  *Exclusion:* Pregnancy, refusal of transvaginal ultrasonography, failure undergo surgical removal of the mass within 120 days of the ultrasound examination, disagreement in the classification between the original pathology report and the report of an expert reviewer, incomplete submission of the data  Type of ultrasound: TVE + TAE in case of a large mass  Experience examiner: EFSUMB level III expert examiners | Included masses: 809  Age: mean 48.8 (SD 15.6)  %Postmenopausal: 45%  CA-125: NR  Prevalence of malignancy: 29.9%  Prevalence of malignancy excl borderline: 23.5%  Histology / follow-up: 100% / 0%  Time between test and surgery: <120 days |
| Van Holsbeke (2008) [^92^](#_ENREF_92)  Study design: prospective cohort  Setting: tertiary care  Number of centers: 1  Accrual: NR  Country: UK  Funding: NR | Subjective Assessment | *Inclusion:* Patients with a complex or difficult to classify adnexal mass who underwent surgery and were pre-operatively scanned by an expert sonologist  *Exclusion:* NR  Type of ultrasound: TVE + TAE  Experience examiner: expert sonologist, >10 years experience, senior clinician at tertiary center | Included masses: 166  Age: NR  %Postmenopausal: NR  CA-125: NR  Prevalence of malignancy: 42.2%  Prevalence of malignancy excl borderline: 21.7%  Histology / follow-up: 100% / 0%  Time between test and surgery: |
| Van Holsbeke (2009) [^93^](#_ENREF_93)  Study design: prospective cohort  Setting: mixed  Number of centers: 3  Accrual: Jun 2002 – Dec 2005  Country: Belgium, Sweden, Italy  Funding: Government: Research Council of the KU Leuven, Belgian Federal Science Policy Office, EU: BIOPATTERN, Swedish medical Research Council, Malmö University Hospital | LR2 (10%)  Subjective Assessment | *Inclusion:* Patients presenting with at least one overt persistent adnexal mass who were assessed by a principal investigator at one of the participating centers  *Exclusion:* Pregnancy or refusal of transvaginal sonography, surgery more than 120 days after sonographic assessment, disagreement in the classification between the original pathology report and the report of an expert reviewer, or incomplete submission of the data  Type of ultrasound: TVE + TAE in case of a large mass  Experience examiner: EFSUMB level III expert examiners | Included masses: 507  Age: NR  %Postmenopausal: NR  CA-125: NR  Prevalence of malignancy: 28.2%  Prevalence of malignancy excl borderline: 24.3%  Histology / follow-up: 100% / 0%  Time between test and surgery: <120 days |
| Van Holsbeke (2011) [^94^](#_ENREF_94)  Study design: prospective cohort  Setting: mixed  Number of centers: 12  Accrual: Nov 2005 – Oct 2007  Country: 12 centers from different countries  Funding: Research Council KU Leuven, Swedish Medical Research Council | RMI 1 (200)  RMI 2 (200)  RMI 3 (200) | *Inclusion:* Patients presenting with at least one adnexal mass who underwent ultrasound examination by principal investigator at one of the participating centers  *Exclusion:* Pregnancy, refusal of transvaginal ultrasonography, failure undergo surgical removal of the mass within 120 days of the ultrasound examination  Type of ultrasound: TVE + TAE in case of a large mass  Experience examiner: EFSUMB level III expert examiners | Included masses: 997  Age: median 44, range 11-94  %Postmenopausal: 35.3%  CA-125: NR  Prevalence of malignancy: 25.6%  Prevalence of malignancy excl borderline: 21.4%  Histology / follow-up: 100% / 0%  Time between test and surgery: <120 days |
| Van Trappen (2007) [^95^](#_ENREF_95)  Study design: prospective cohort  Setting: tertiary care  Number of centers: 1  Accrual: NR  Country: UK  Funding: NR | Subjective Assessment | *Inclusion:* Women aged between 15 - 92 years with a suspected adnexal mass  *Exclusion:* NR  Type of ultrasound: TVE + TAE  Experience examiner: SA: radiologist with an interest in gynecological scanning | Included masses: 142  Age: mean 49, range 15-92  %Postmenopausal: NR  CA-125: NR  Prevalence of malignancy: 28.2%  Prevalence of malignancy excl borderline: NR  Histology / follow-up: 84.5% /15.5 %  Time between test and surgery: NR |
| Velayo *et al* (2022) [^96^](#_ENREF_96)  Study design: prospective cohort  Setting: mixed  Number of centers: 2  Accrual: NR  Country: Philippines  Funding: Industry: Aspira Women's Health, Inc., USA, Hi-Precision Diagnostics, Philipines | LR2 (10%) | *Inclusion:* > 18 years, considered for surgery, not previously evaluated by a gynecologic oncologist, and not diagnosed with cancer in the past five years.  *Exclusion:* NR  Type of ultrasound: Combination  Experience examiner: level III specialist in ultrasonographic and Doppler evaluation | Included masses: 286  Age: mean 44.1 (SD 13.1), range 18-78  %Postmenopausal: 34.6%  CA-125: NR  Prevalence of malignancy: 46.5%  Prevalence of malignancy excl borderline: 35.7%  Histology / follow-up: 100% / 0%  Time between test and surgery: NR |
| Velayo *et al* (2022) [^97^](#_ENREF_97)  Study design: prospective cohort  Setting: mixed  Number of centers: 2  Accrual: NR  Country: Philippines  Funding: Industry: Aspira Women's Health, Inc., USA, Hi-Precision Diagnostics, Philipines | ADNEX (20%) | *Inclusion:* Non-pregnant females, ≥ 18 years of age, diagnosed with an ovarian mass, considered for surgery, who had not been previously evaluated by a gynecologic-oncologist, and had not been diagnosed with cancer in the past five years  *Exclusion:* Mental disabilities, severe co-morbid conditions, or were found to be pregnant during initial recruitment.  Type of ultrasound: Combination  Experience examiner: examiner: level III specialist in ultrasonographic and Doppler evaluation | Included masses: 260  Age: mean 44.2 (SD 13.0), range 18-78  %Postmenopausal: 35%  CA-125: NR  Prevalence of malignancy: 45.8%  Prevalence of malignancy excl borderline: NR  Histology / follow-up: 100% / 0%  Time between test and surgery: NR |
| Vilendecic (2023) [^98^](#_ENREF_98)  Study design: prospective cohort  Setting: tertiary care  Number of centers: 1  Accrual: Jan 2019 – Jun 2021  Country: Serbia  Funding: None | RMI (200)  Simple Rules + Subjective Assessment  ADNEX (10%)  Subjective Assessment | *Inclusion:* Patients ≥ 18 years of age with at least one adnexal mass of an ovarian origin scheduled for elective surgery based on clinical and laboratory findings  *Exclusion:* Pregnancy, a history of previous malignancies, malignancies diagnosed during preoperative workup, incomplete preoperative assessment, and failure to undergo surgical procedure  Type of ultrasound: TVE + TAE in case of a large mass  Experience examiner: EFSUMB level II, one with and one without IOTA certification | Included masses: 396  Age: NR  %Postmenopausal: 54.8%  CA-125: NR  Prevalence of malignancy: 29.8%  Prevalence of malignancy excl borderline: 24.5%  Histology / follow-up: 100% / 0%  Time between test and surgery: NR |
| Xu (2022) [^99^](#_ENREF_99)  Study design: prospective cohort  Setting: tertiary care  Number of centers: 1  Accrual: Nov 2016 – Jun 2020  Country: China  Funding: NR | Simple Rules + Malignant | *Inclusion:* Patients with a suspected malignant adnexal masses admitted to Lanzhou University Second Hospital. Who had conventional ultrasound (US) and CEUS examination before surgery, pathology results available  *Exclusion:* NR    Type of ultrasound: TVE + TAE  Experience examiner: >5 years of experience | Included masses: 180  Age: NR  %Postmenopausal: NR  CA-125: NR  Prevalence of malignancy: 41.1%  Prevalence of malignancy excl borderline: 33.3%  Histology / follow-up: 100% / 0%  Time between test and surgery: NR |

*****Only the index test(s) and cut-off value(s) included in the systematic review. Some articles reported other index test(s) and/or cutoff value(s), but these were excluded due to insufficient data or the presence of duplicate data from other included studies.

1. Akturk E, Karaca RE, Alanbay I, Dede M, Karasahin E, Yenen MC, et al. Comparison of four malignancy risk indices in the detection of malignantovarian masses. Journal of gynecologic oncology. 2011;22(3):177-82.

2. Al Musalhi K, Al Kindi M, Al Aisary F, Ramadhan F, Al Rawahi T, Al Hatali K, et al. Evaluation of HE4, CA-125, Risk of Ovarian Malignancy Algorithm (ROMA) and Risk of Malignancy Index (RMI) in the Preoperative Assessment of Patientswith Adnexal Mass. Oman Med J. 2016;31(5):336-44.

3. Alcázar JL, Pascual MA, Olartecoechea B, Graupera B, Aubá M, Ajossa S, et al. IOTA simple rules for discriminating between benign and malignant adnexalmasses: prospective external validation. Ultrasound in obstetrics & gynecology : the official journal of theInternational Society of Ultrasound in Obstetrics and Gynecology. 2013;42(4):467-71.

4. Alcazar JL, Pascual MA, Graupera B, Auba M, Errasti T, Olartecoechea B, et al. External validation of IOTA simple descriptors and simple rules forclassifying adnexal masses. Ultrasound in obstetrics & gynecology : the official journal of theInternational Society of Ultrasound in Obstetrics and Gynecology. 2016;48(3):397-402.

5. Andersen ES, Knudsen A, Rix P, Johansen B. Risk of Malignancy Index in the preoperative evaluation of patients withadnexal masses. Gynaecol Oncol. 2003;90(1):109-12.

6. Antovska VS, Bashevska N, Aleksioska N. Predictive values of the ultrasound parameters, CA-125 and risk ofmalignancy index in patients with ovarian cancer. Klinicka Onkologie. 2011;24(6):435-42.

7. Araujo KG, Jales RM, Pereira PN, Yoshida A, de Angelo Andrade L, Sarian LO, et al. Performance of the IOTA ADNEX model in preoperative discrimination of adnexal masses in a gynecological oncology center. Ultrasound Obstet Gynecol. 2017;49(6):778-83.

8. Arun-Muthuvel V, Jaya V. Pre-operative evaluation of ovarian tumors by risk of malignancy index,CA125 and ultrasound. Asian Pacific journal of cancer prevention : APJCP. 2014;15(6):2929-32.

9. Aslam N, Banerjee S, Carr JV, Savvas M, Hooper R, Jurkovic D. Prospective evaluation of logistic regression models for the diagnosis ofovarian cancer. Obstetrics and Gynecology. 2000;96(1):75-80.

10. Auekitrungrueng R, Tinnangwattana D, Tantipalakorn C, Charoenratana C, Lerthiranwong T, Wanapirak C, et al. Comparison of the diagnostic accuracy of International Ovarian TumorAnalysis simple rules and the risk of malignancy index to discriminatebetween benign and malignant adnexal masses. Int J Gynaecol Obstet. 2019;146(3):364-9.

11. Borges AL, Brito M, Ambrósio P, Condeço R, Pinto P, Ambrósio B, et al. Prospective external validation of IOTA methods for classifying adnexal masses and retrospective assessment of two-step strategy using benign descriptors and ADNEX model: Portuguese multicenter study. Ultrasound Obstet Gynecol. 2024;64(4):538-49.

12. Campos C, Sarian LO, Jales RM, Hartman C, Araújo KG, Pitta D, et al. Performance of the Risk of Malignancy Index for Discriminating MalignantTumors in Women With Adnexal Masses. J Ultrasound Med. 2016;35(1):143-52.

13. Chankrachang A, Lattiwongsakorn W, Tantipalakorn C, Tongsong T. Diagnostic Performance of ADNEX Model and IOTA Simple Rules in Differentiating Malignant from Benign Adnexal Masses When Assessed by Non-Expert Examiners. Journal of Clinical Medicine. J Clin Med. 2025;14(8):2776.

14. Chen H, Qian L, Jiang M, Du Q, Yuan F, Feng W. Performance of IOTA ADNEX model in evaluating adnexal masses in a gynecological oncology center in China. Ultrasound Obstet Gynecol. 2019;54(6):815-22.

15. Chen G-Y, Hsu T-F, Chan IS, Liu C-H, Chao W-T, Shih Y-C, et al. Comparison of the O-RADS and ADNEX models regarding malignancy rate andvalidity in evaluating adnexal lesions. European radiology. 2022;32(11):7854-64.

16. Christiansen F, Epstein EL, Smedberg E, Åkerlund M, Smith K, Epstein E. Ultrasound image analysis using deep neural networks for discriminating between benign and malignant ovarian tumors: comparison with expert subjective assessment. Ultrasound Obstet Gynecol. 2021;57(1):155-63.

17. Daemen A, Valentin L, Fruscio R, Van Holsbeke C, Melis GB, Guerriero S, et al. Improving the preoperative classification of adnexal masses as benign or malignant by second-stage tests. Ultrasound Obstet Gynecol. 2011;37(1):100-6.

18. Dakhly DMR, Gaafar HM, Sediek MM, Ibrahim MF, Momtaz M. Diagnostic value of the International Ovarian Tumor Analysis (IOTA) simple rules versus pattern recognition to differentiate between malignant and benign ovarian masses. Int J Gynaecol Obstet. 2019;147(3):344-9.

19. Dang Thi Minh N, Nguyen Van T, Duong Duc H, Nguyen Tuan M, Duong Thi Tra G, Do Tuan D, et al. IOTA simple rules: An efficient tool for evaluation of ovarian tumors by non-experienced but trained examiners - A prospective study. Heliyon. 2024;10(2):e24262.

20. Dewangan S, Gupta S, Chawla I. Comparison of Simple Ultrasound Rules by International Ovarian Tumor Analysis (IOTA) with RMI-1 and RMI-4 (Risk of Malignancy Index) in Preoperative Differentiation of Benign and Malignant Adnexal Masses. J Obstet Gynaecol India. 2024;74(2):158-64.

21. Einig S, Puls T, Reina H, Schoetzau A, Montavon C, Butenschön A, et al. External validation of the IOTA two-step strategy in the preoperative characterization of ovarian masses. Eur J Obstet Gynecol Reprod Biol. 2025;310:113981.

22. Elsner Hernández N, De Luis Escudero JF, Pérez Méndez LI, Báez Quintana DR, Bruno Santana E, Pérez Álvarez JA, et al. Evaluation of the incorporation of an IOTA-ADNEX model in the discrimination of adnexal masses in our third-level hospital centre, taking into account the menopausal status of patients. Five years of experience. Clinica e Investigacion en Ginecologia y Obstetricia. 2024;51(1).

23. Ertas S, Vural F, Tufekci EC, Ertas AC, Kose G, Aka N. Predictive Value of Malignancy Risk Indices for Ovarian Masses inPremenopausal and Postmenopausal Women. Asian Pac J Cancer Prev. 2016;17(4):2177-83.

24. Giourga M, Petropoulos I, Stavros S, Potiris A, Gerede A, Sapantzoglou I, et al. Enhancing Ovarian Tumor Diagnosis: Performance of Convolutional Neural Networks in Classifying Ovarian Masses Using Ultrasound Images. J Clin Med. 2024;13(14).

25. Haliti TI, Hoxha I, Mojsiu R, Mandal R, Goç G, Hoti KD. Diagnostic Accuracy of Biomarkers and International Ovarian Tumor Analysis Simple Rules in Diagnosis of Ovarian Cancer. Hematology/Oncology Clinics of North America. 2024;38(1):251-65.

26. Hartman CA, Juliato CR, Sarian LO, Toledo MC, Jales RM, Morais SS, et al. Ultrasound criteria and CA 125 as predictive variables of ovarian cancerin women with adnexal tumors. Ultrasound in obstetrics & gynecology : the official journal of theInternational Society of Ultrasound in Obstetrics and Gynecology. 2012;40(3):360-6.

27. He P, Wang J-J, Duan W, Song C, Yang Y, Wu Q-Q. Estimating the risk of malignancy of adnexal masses: validation of the ADNEX model in the hands of nonexpert ultrasonographers in a gynaecological oncology centre in China. J Ovarian Res. 2021;14(1):169.

28. Hidalgo JJ, Ros F, Aubá M, Errasti T, Olartecoechea B, Ruiz-Zambrana Á, et al. Prospective external validation of IOTA three-step strategy forcharacterizing and classifying adnexal masses and retrospective assessmentof alternative two-step strategy using simple-rules risk. Ultrasound in obstetrics & gynecology : the official journal of theInternational Society of Ultrasound in Obstetrics and Gynecology. 2019;53(5):693-700.

29. Hidalgo JJ, Llueca A, Zolfaroli I, Veiga N, Ortiz E, Alcázar JL. Comparison of IOTA three-step strategy and logistic regression model LR2for discriminating between benign and malignant adnexal masses. Medical Ultrasonography. 2021;23(2):168-75.

30. Hiett AK, Sonek JD, Guy M, Reid TJ. Performance of IOTA Simple Rules, Simple Rules risk assessment, ADNEX model and O-RADS in differentiating between benign and malignant adnexal lesions in North American women. Ultrasound Obstet Gynecol. 2022;59(5):668-76.

31. Irshad F, Irshad M, Naz M, Asim Ikram M. Accuracy of "risk of malignancy index" in the preoperative diagnosis of ovarian malignancy in post menopausal women. Rawal Medical Journal. 2013;38(3):266-70.

32. Jabeen R, Khan SA, Naveed S. Risk of Malignancy index in the preoperative evaluation of patients withovarian masses. Rawal Medical Journal. 2015;40(1):78-80.

33. Javdekar R, Maitra N. Risk of Malignancy Index (RMI) in Evaluation of Adnexal Mass. J Obstet Gynaecol India. 2015;65(2):117-21.

34. Janas L, Stachowiak G, Glowacka E, Piwowarczyk I, Kajdos M, Soja M, et al. The use of CA125, human epididymis protein 4 (HE4), risk of ovarian malignancy algorithm (ROMA), risk of malignancy index (RMI) and subjective assessment (SA) in preoperative diagnosing of ovarian tumors. Ginekol Pol. 2024;95(5):321-7.

35. Jeong SY, Park BK, Lee YY, Kim TJ. Validation of IOTA-ADNEX model in discriminating characteristics o fadnexal masses: A comparison with subjective assessment. J Clin Med. 2020;9(6):1-10.

36. Kapoor S, Singhal S, Dhamija E, Manchanda S, Malhotra N, Bhatla N. Diagnostic performance of ultrasound reporting systems in evaluation of adnexal masses: A prospective observational study. European Journal of Obstetrics and Gynecology and Reproductive Biology. 2024;301:186-93.

37. Kaya H, Sandal K, Gocmen A, Yilmaz İ. Comparison of CA 125, CA 72-4, risk of malignancy index and depriestscoring system in the differentiation between benign and malignant adnexalmasses. Medical Journal of Bakirkoy. 2020;16(2):95-102.

38. Knafel A, Banas T, Nocun A, Wiechec M, Jach R, Ludwin A, et al. The Prospective External Validation of International Ovarian TumorAnalysis (IOTA) Simple Rules in the Hands of Level I and II Examiners. Ultraschall in der Medizin. 2016;37(5):516-23.

39. Kougioumtsidou A, Karavida A, Mamopoulos A, Dagklis T, Tsakiridis I, Kopatsaris S, et al. Performance of International Ovarian Tumor Analysis (IOTA) predictive models in preoperative discrimination between benign and malignant adnexal lesions: preliminary outcomes in a Tertiary Care Hospital in Greece. Archives of Gynecology and Obstetrics. 2025;311(1):113-22.

40. Lems E, Mongula JE, Muntinga CLP, Leemans JC, Hoogstad-van Evert JS, Smit RA, et al. Real-world performance of the Assessment of Different NEoplasias in the adneXa (ADNEX) model for the pre-operative classification of ovarian tumors. Int J Gynaecol C. 2025;35(7):101917.

41. Liest AL, Omran AS, Mikiver R, Rosenberg P, Uppugunduri S. RMI and ROMA are equally effective in discriminating between benign and malignant gynecological tumors: A prospective population-based study. Acta Obstet Gynecol Scand. 2019;98(1):24-33.

42. Lycke M, Kristjansdottir B, Sundfeldt K. A multicenter clinical trial validating the performance of HE4, CA125,risk of ovarian malignancy algorithm and risk of malignancy index. Gynaecol Oncol. 2018;151(1):159-65.

43. Mahajan G, Bhatia R, Kaura M, Kaur P, Kaur M. Risk of malignancy index (Rmi-2) in the evaluation of adenexal masses. Indian Journal of Public Health Research and Development. 2020;11(8):64-70.

44. Malla VG, Tuteja A, Singh P. Rethinking the role of serum cancer antigen 125 and risk of malignancyindex in Indian women with ovarian masses: Newer perspectives and reviewof literature. Journal of SAFOG. 2018;10(2):110-7.

45. Manodarshni M, Pallavee P, Samal R. Comparison of International Ovarian Tumor Analysis Simple Rules with Risk of Malignancy Index for Preoperative Differentiation of Benign and Malignant Adnexal Masses. Journal of South Asian Federation of Obstetrics and Gynaecology. 2023;15(3):321-5.

46. Meray O, Turkcuoglu I, Meydanli MM, Kafkasli A. Risk of malignancy index is not sensitive in detecting non-epithelialovarian cancer and borderline ovarian tumor. J Turk Ger Gynecol Assoc. 2010;11(1):22-6.

47. Meys EMJ, Jeelof LS, Achten NMJ, Slangen BFM, Lambrechts S, Kruitwagen RFPM, et al. Estimating risk of malignancy in adnexal masses: external validation ofthe ADNEX model and comparison with other frequently used ultrasoundmethods. Ultrasound in obstetrics & gynecology : the official journal of theInternational Society of Ultrasound in Obstetrics and Gynecology. 2017;49(6):784-92.

48. Mohammed ABF, Ahuga VK, Taha M. Validation of the Risk of Malignancy Index in primary evaluation ofovarian masses. Middle East Fertility Society Journal. 2014;19(4):324-8.

49. Moshina B, Ghose S. Preoperative analysis of risk of malignancy indices in the distinction ofmalignant ovarian tumors. Clin Cancer Investig J. 2020;9(6):238-43.

50. Moszynski R, Zywica P, Wojtowicz A, Szubert S, Sajdak S, Stachowiak A, et al. Menopausal status strongly influences the utility of predictive models in differential diagnosis of ovarian tumors: an external validation of selected diagnostic tools. Ginekol Pol. 2014;Dec(12):892-9.

51. Mulder EE, Gelderblom ME, Schoot D, Vergeldt TFM, Nijssen DL, Piek JMJ. External validation of Risk of Malignancy Index compared to IOTA SimpleRules. Acta Radiologica. 2021;62(5):673-8.

52. Ngu SF, Chai YK, Choi KM, Leung TW, Li J, Kwok GST, et al. Diagnostic Performance of Risk of Malignancy Algorithm (ROMA), Risk ofMalignancy Index (RMI) and Expert Ultrasound Assessment in a Pelvic MassClassified as Inconclusive by International Ovarian Tumour Analysis (IOTA)Simple Rules. Cancers. 2022;14(3).

53. Niemi RJ, Saarelainen SK, Luukkaala TH, Mäenpää JU. Reliability of preoperative evaluation of postmenopausal ovarian tumors. J Ovarian Res. 2017;10(1).

54. Nunes N, Ambler G, Hoo WL, Naftalin J, Foo X, Widschwendter M, et al. A prospective validation of the IOTA logistic regression models (LR1 andLR2) in comparison to subjective pattern recognition for the diagnosis ofovarian cancer. Int J Gynaecol C. 2013;23(9):1583-9.

55. Nunes N, Ambler G, Foo X, Naftalin J, Widschwendter M, Jurkovic D. Use of IOTA simple rules for diagnosis of ovarian cancer: meta-analysis. Ultrasound in obstetrics & gynecology : the official journal of theInternational Society of Ultrasound in Obstetrics and Gynecology. 2014;44(5):503-14.

56. Nunes N, Ambler G, Foo X, Widschwendter M, Jurkovic D. Prospective evaluation of IOTA logistic regression models LR1 and LR2 incomparison with subjective pattern recognition for diagnosis of ovarian cancer in an outpatient setting. Ultrasound Obstet Gynecol. 2018;51(6):829-35.

57. Pascual MA, Vancraeynest L, Timmerman S, Ceusters J, Ledger A, Graupera B, et al. Validation of ADNEX and IOTA two-step strategy and estimation of risk of complications during follow-up of adnexal masses in low-risk population. Ultrasound in obstetrics & gynecology : the official journal of the International Society of Ultrasound in Obstetrics and Gynecology. 2024;64(3):395-404.

58. Peces Rama A, Llanos Llanos MC, Sánchez Ferrer ML, Alcázar Zambrano JL, Martínez Mendoza A, Nieto Díaz A. Simple descriptors and simple rules of the International Ovarian Tumor Analysis (IOTA) Group: A prospective study of combined use for the description of adnexal masses. Eur J Obstet Gynecol Reprod Biol. 2015;195:7-11.

59. Pereira PN, Sarian LO, Yoshida A, Araújo KG, Silva ACB, de Oliveira Barros RH, et al. Improving the performance of IOTA simple rules: sonographic assessment ofadnexal masses with resource-effective use of a magnetic resonance scoring(ADNEX MR scoring system). Abdominal Radiology. 2020;45(10):3218-29.

60. Phinyo P, Patumanond J, Saenrungmuaeng P, Chirdchim W, Pipanmekaporn T, Tantraworasin A, et al. Diagnostic Added-Value of Serum CA-125 on the IOTA Simple Rules andDerivation of Practical Combined Prediction Models (IOTA SR X CA-125). Diagnostics (Basel, Switzerland). 2021;11(2).

61. Piovano E, Cavallero C, Fuso L, Viora E, Ferrero A, Gregori G, et al. Diagnostic accuracy and cost-effectiveness of different strategies totriage women with adnexal masses: a prospective study. Ultrasound in obstetrics & gynecology : the official journal of theInternational Society of Ultrasound in Obstetrics and Gynecology. 2017;50(3):395-403.

62. Poonyakanok V, Tanmahasamut P, Jaishuen A, Wongwananuruk T, Asumpinwong C, Panichyawat N, et al. Preoperative Evaluation of the ADNEX Model for the Prediction of the Ovarian Cancer Risk of Adnexal Masses at Siriraj Hospital. Gynaecol Obstet Invest. 2021;86(1):132-8.

63. Poonyakanok V, Tanmahasamut P, Jaishuen A. Prospective comparative trial comparing O-RADS, IOTA ADNEX model, and RMI score for preoperative evaluation of adnexal masses for prediction of ovarian cancer. J Obstet Gynaecol Res. 2023;49(5):1412-7.

64. Richards A, Herbst U, Manalang J, Pather S, Saidi S, Tejada-Berges T, et al. HE4, CA125, the Risk of Malignancy Algorithm and the Risk of Malignancy Index and complex pelvic masses - a prospective comparison in the pre-operative evaluation of pelvic masses in an Australian population. Aust N Z J Obstet Gynaecol. 2015;55(5):493-7.

65. Ruiz de Gauna B, Rodriguez D, Olartecoechea B, Auba M, Jurado M, Gomez Roig MD, et al. Diagnostic performance of IOTA simple rules for adnexal massesclassification: a comparison between two centers with different ovariancancer prevalence. European journal of obstetrics, gynecology, and reproductive biology. 2015;191:10-4.

66. Sayasneh A, Preisler J, Stlader C, Husicka R, Naji O, Kaijser J, et al. A randomised controlled trial to compare the clinical impact of RMI versusLR2 to characterise adnexal masses: interim analysis of phase 4 IOTA study. BJOG. 2013;120:357‐8.

67. Sayasneh A, Ferrara L, De Cock B, Saso S, Al-Memar M, Johnson S, et al. Evaluating the risk of ovarian cancer before surgery using the ADNEX model: A multicentre external validation study. Br J Cancer. 2016;115(5):542-8.

68. Shimada K, Matsumoto K, Mimura T, Ishikawa T, Munechika J, Ohgiya Y, et al. Ultrasound-based logistic regression model LR2 versus magnetic resonance imaging for discriminating between benign and malignant adnexal masses: a prospective study. Int J Clin Oncol. 2018;23(3):514-21.

69. Silvestre L, Martins WP, Candido-Dos-Reis FJ. Limitations of three-dimensional power Doppler angiography in preoperativeevaluation of ovarian tumors. Journal of Ovarian Research. 2015;8(1).

70. Solanki V, Singh P, Sharma C, Ghuman N, Sureka B, Shekhar S, et al. Predicting Malignancy in Adnexal Masses by the International Ovarian TumorAnalysis-Simple Rules. Journal of mid-life health. 2020;11(4):217-23.

71. Spagnol G, Marchetti M, De Tommasi O, Vitagliano A, Cavallin F, Tozzi R, et al. Simple rules, O-RADS, ADNEX and SRR model: Single oncologic center validation of diagnostic predictive models alone and combined (two-step strategy) to estimate the risk of malignancy in adnexal masses and ovarian tumors. Gynaecol Oncol. 2023;177:109-16.

72. Spagnol G, Marchetti M, Carollo M, Bigardi S, Tripepi M, Facchetti E, et al. Clinical Utility and Diagnostic Accuracy of ROMA, RMI, ADNEX, HE4, and CA125 in the Prediction of Malignancy in Adnexal Masses. Cancers. 2024;16(22):3790.

73. Stukan M, Badocha M, Ratajczak K. Development and validation of a model that includes two ultrasoundparameters and the plasma D-dimer level for predicting malignancy inadnexal masses: An observational study. BMC Cancer. 2019;19(1).

74. Sundar S, Agarwal R, Davenport C, Scandrett K, Johnson S, Sengupta P, et al. Risk-prediction models in postmenopausal patients with symptoms of suspected ovarian cancer in the UK (ROCkeTS): a multicentre, prospective diagnostic accuracy study. Lancet Oncol. 2024;25(10):1371-86.

75. Szubert S, Wojtowicz A, Moszynski R, Zywica P, Dyczkowski K, Stachowiak A, et al. External validation of the IOTA ADNEX model performed by two independent gynecologic centers. Gynecol Oncol. 2016;142(3):490-5.

76. Tangjanyatham P, Chaowawanit W. Comparison of sensitivity for Risk of Ovarian Malignancy Algorithm (ROMA) and Assessment of Different NEoplasias in the adneXa (ADNEX) model for predicting ovarian cancer in a woman with adnexal masses. Int J Gynaecol C. 2025;35(6):101827.

77. Tantipalakorn C, Wanapirak C, Khunamornpong S, Sukpan K, Tongsong T. IOTA simple rules in differentiating between benign and malignant ovariantumors. Asian Pacific journal of cancer prevention : APJCP. 2014;15(13):5123-6.

78. Terzic M, Dotlic J, Ladjevic IL, Atanackovic J, Ladjevic N. Evaluation of the risk malignancy index diagnostic value in patients withadnexal masses. Vojnosanit Pregl. 2011;68(7):589-93.

79. Terzic M, Dotlic J, Likic I, Brndusic N, Pilic I, Ladjevic N, et al. Risk of malignancy index validity assessment in premenopausal andpostmenopausal women with adnexal tumors. Taiwan J Obstet Gynecol. 2013;52(2):253-7.

80. Testa A, Kaijser J, Wynants L, Fischerova D, Van Holsbeke C, Franchi D, et al. Strategies to diagnose ovarian cancer: New evidence from phase 3 of themulticentre international IOTA study. Br J Cancer. 2014;111(4):680-8.

81. Timmerman D, Verrelst H, Bourne TH, De Moor B, Collins WP, Vergote I, et al. Artificial neural network models for the preoperative discriminationbetween malignant and benign adnexal masses. Ultrasound Obstet Gynecol. 1999;13(1):17-25.

82. Timmerman D, Schwarzler P, Collins WP, Claerhout F, Coenen M, Amant F, et al. Subjective assessment of adnexal masses with the use of ultrasonography: an analysis of interobserver variability and experience. Ultrasound Obstet Gynecol. 1999;13(1):11-6.

83. Timmerman D, Ameye L, Fischerova D, Epstein E, Melis GB, Guerriero S, et al. Simple ultrasound rules to distinguish between benign and malignant adnexal masses before surgery: Prospective validation by IOTA group. BMJ. 2011;342(7788):94.

84. Tongsong T, Wanapirak C, Sukpan K, Khunamornpong S, Pathumbal A. Subjective sonographic assessment for differentiation between malignantand benign adnexal masses. Asian Pac J Cancer Prev. 2007;8(1):124-6.

85. Ulusoy S, Akbayir O, Numanoglu C, Ulusoy N, Odabas E, Gulkilik A. The risk of malignancy index in discrimination of adnexal masses. Int J Gynaecol Obstet. 2007;96(3):186-91.

86. Valentin L. Prospective cross-validation of Doppler ultrasound examination and gray-scale ultrasound imaging for discrimination of benign and malignant pelvic masses. Ultrasound Obstet Gynecol. 1999;14:273-83.

87. Valentin L, Hagen B, Tingulstad S, Eik-Nes S. Comparison of 'pattern recognition' and logistic regression models for discrimination between benign and malignant pelvic masses: a prospective cross validation. Ultrasound Obstet Gynecol. 2001;18(4):357-65.

88. Van Calster B, Timmerman D, Bourne T, Testa AC, Van Holsbeke C, Domali E, et al. Discrimination between benign and malignant adnexal masses by specialist ultrasound examination versus serum CA-125. J Natl Cancer Inst. 2007;99(22):1706-14.

89. Van Calster B, Valentin L, Froyman W, Landolfo C, Ceusters J, Testa AC, et al. Validation of models to diagnose ovarian cancer in patients managedsurgically or conservatively: multicentre cohort study. BMJ. 2020;370:m2614.

90. van den Akker PAJ, Zusterzeel PLM, Aalders AL, Snijders MPLM, Samlal RAK, Vollebergh JHA, et al. External validation of the adapted Risk of Malignancy Index incorporatingtumor size in the preoperative evaluation of adnexal masses. Eur J Obstet Gynecol Reprod Biol. 2011;159(2):422-5.

91. Van Holsbeke C, Van Calster B, Valentin L, Testa AC, Ferrazzi E, Dimou I, et al. External validation of mathematical models to distinguish between benignand malignant adnexal tumors: A multicenter study by the InternationalOvarian Tumor Analysis group. Clin Cancer Res. 2007;13(15):4440-7.

92. van Holsbeke C, Yazbek J, Holland TK, Daemen A, De Moor B, Testa AC, et al. Real-time ultrasound vs. evaluation of static images in the preoperative assessment of adnexal masses. Ultrasound Obstet Gynecol. 2008;32(6):828-31.

93. Van Holsbeke C, Van Calster B, Testa AC, Domali E, Lu C, Van Huffel S, et al. Prospective internal validation of mathematical models to predict malignancy in adnexal masses: Results from the international ovarian tumor analysis study. Clin Cancer Res. 2009;15(2):684-91.

94. Van Holsbeke C, Van Calster B, Bourne T, Ajossa S, Testa AC, Guerriero S, et al. External validation of Diagnostic Models to Estimate the Risk of Malignancy in Adnexal Masses. Clin Cancer Res. 2012;18(3):815-25.

95. Van Trappen PO, Rufford BD, Mills TD, Sohaib SA, Webb JAW, Sahdev A, et al. Differential diagnosis of adnexal masses: Risk of malignancy index, ultrasonography, magnetic resonance imaging, and radioimmunoscintigraphy. Int J Gynaecol C. 2007;17(1):61-7.

96. Velayo CL, Reforma KN, Sicam RVG, Diwa MH, Sy ADR, Tantengco OAG. Improving diagnostic strategies for ovarian cancer in Filipino women using ultrasound imaging and a multivariate index assay. Cancer Epidemiol. 2022;81:102253.

97. Velayo CL, Reforma KN, Sicam RVG, Diwa MH, Sy ADR, Tantengco OAG. Diagnostic Performances of Ultrasound-Based Models for Predicting Malignancy in Patients with Adnexal Masses. Healthcare (Basel). 2022;11(1).

98. Vilendecic Z, Radojevic M, Stefanovic K, Dotlic J, Likic Ladjevic I, Dugalic S, et al. Accuracy of IOTA Simple Rules, IOTA ADNEX Model, RMI, and Subjective Assessment for Preoperative Adnexal Mass Evaluation: The Experience of a Tertiary Care Referral Hospital. Gynaecol Obstet Invest. 2023;88(2):116-22.

99. Xu J, Huang Z, Zeng J, Zheng Z, Cao J, Su M, et al. Value of Contrast-Enhanced Ultrasound Parameters in the Evaluation of Adnexal Masses with Ovarian–Adnexal Reporting and Data System Ultrasound. Ultrasound in Medicine and Biology. 2023;49(7):1527-34.

**Table S6** Quality assessment of included studies

*S6.1, Quality assessment of all included studies regarding the RMI 1*


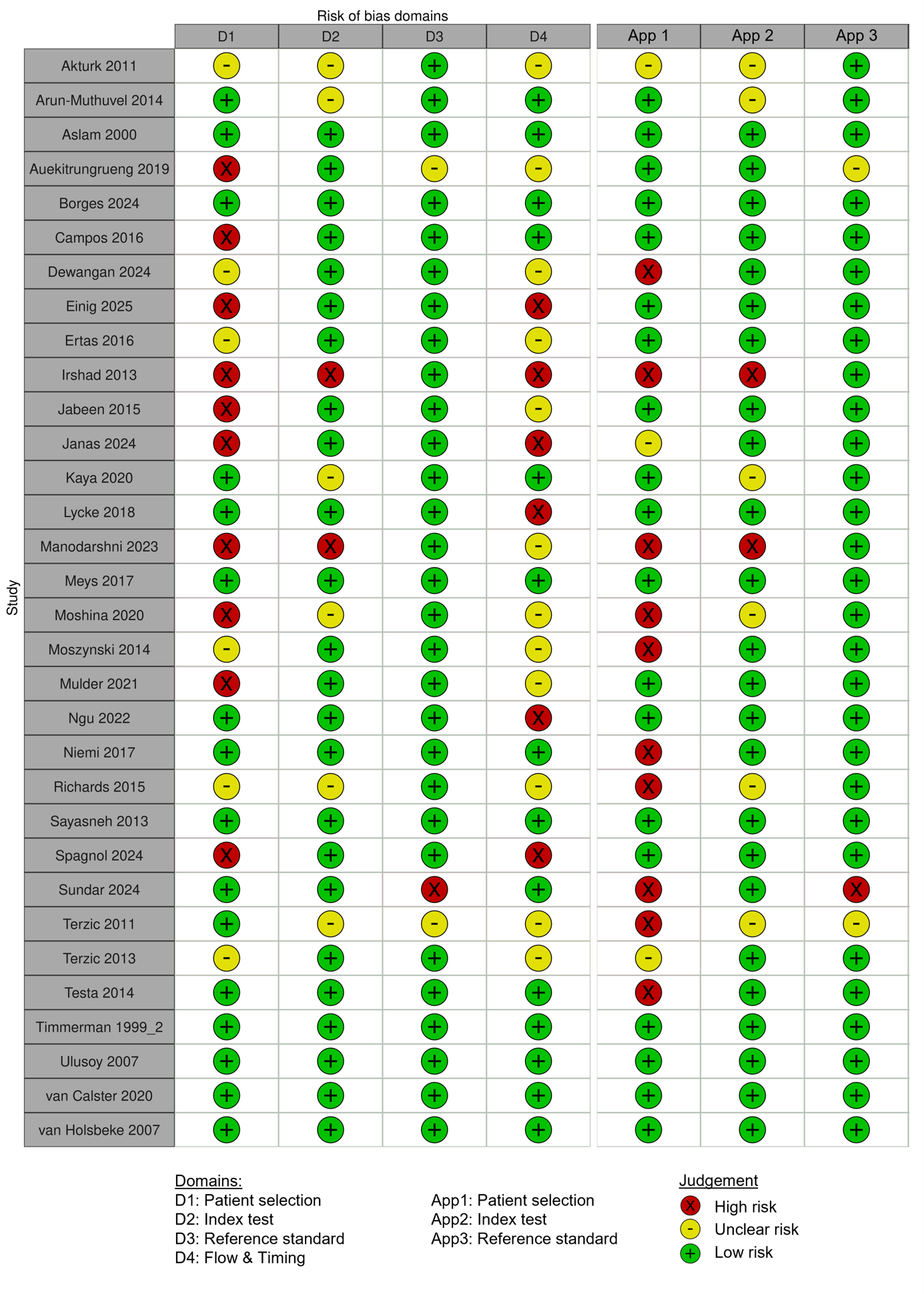


*S6.2, Quality assessment of all included studies regarding the RMI 2*

*
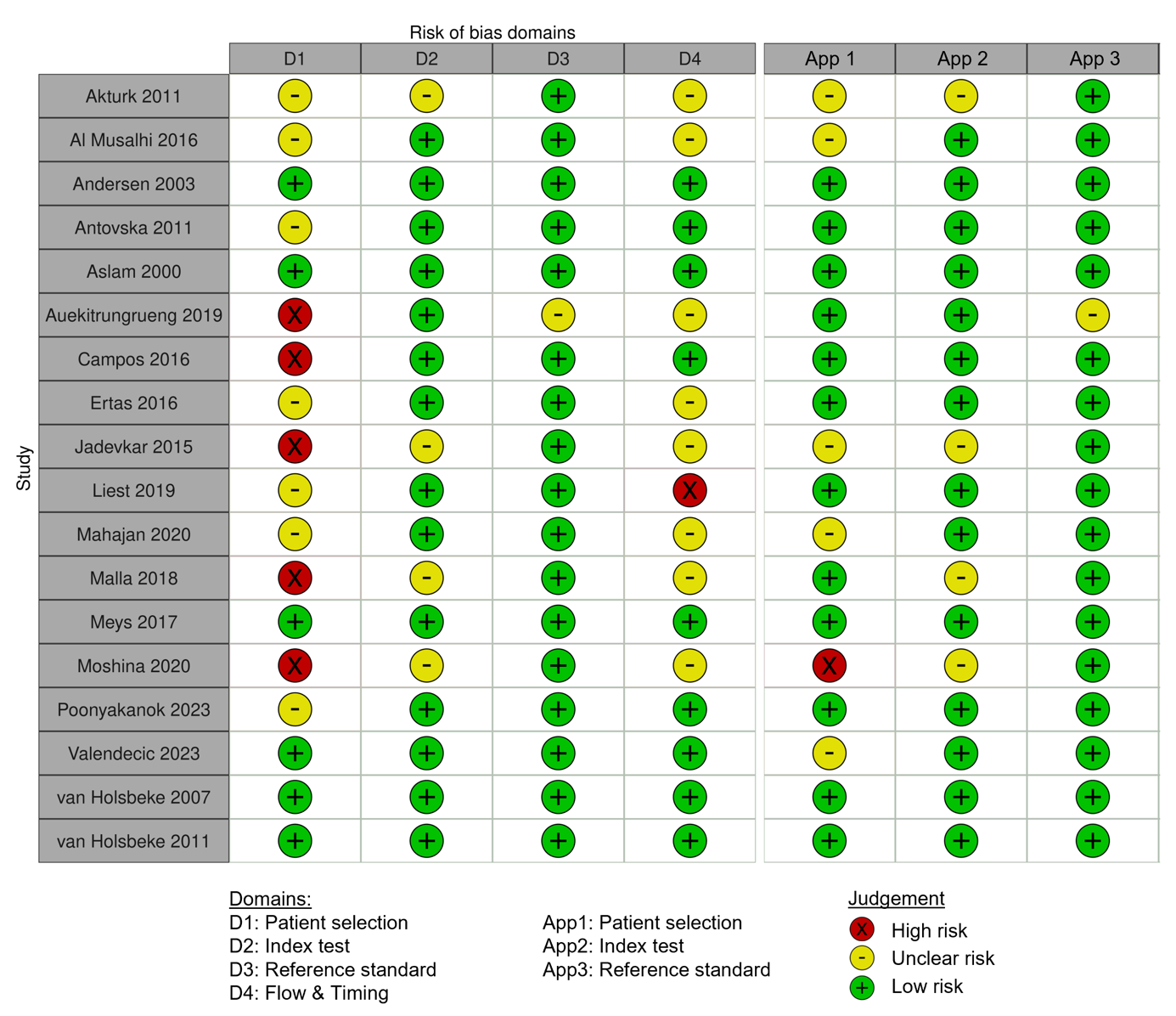
*

*S6.3, Quality assessment of all included studies regarding the RMI 3*

*
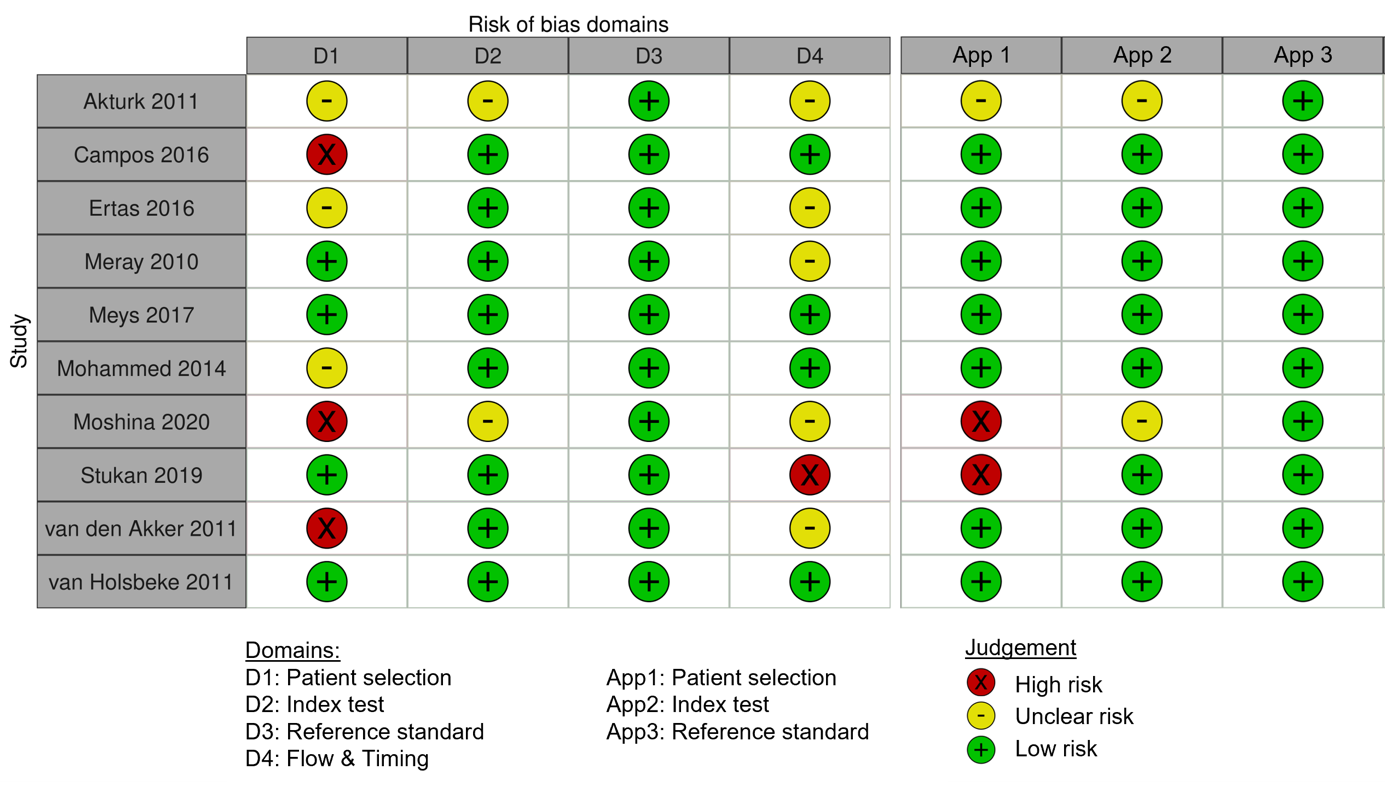
*

*S6.4, Quality assessment of all included studies regarding the LR2 model*

*
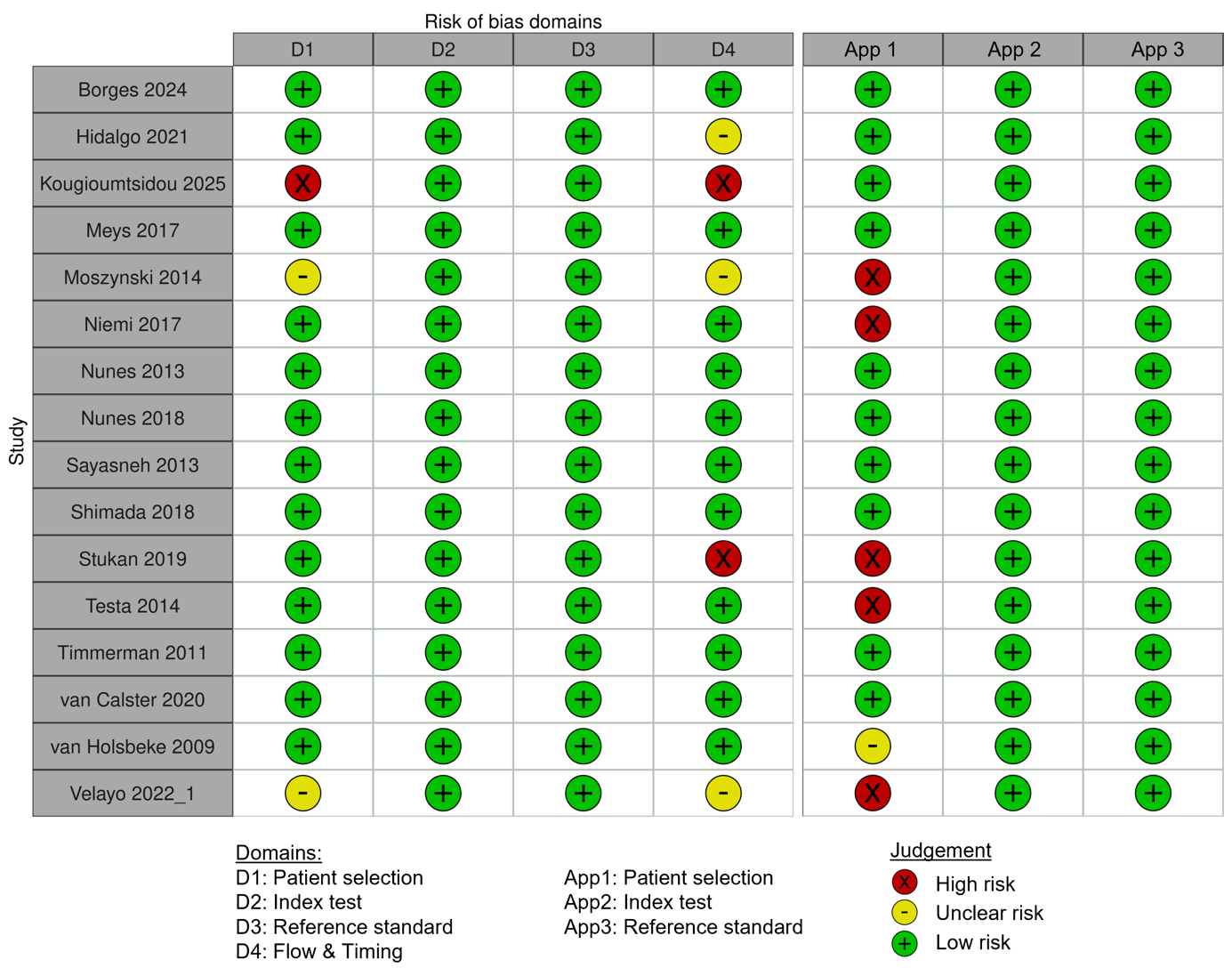
*

*S6.5, Quality assessment of all included studies regarding the Simple Rules + Malignant strategy*

*
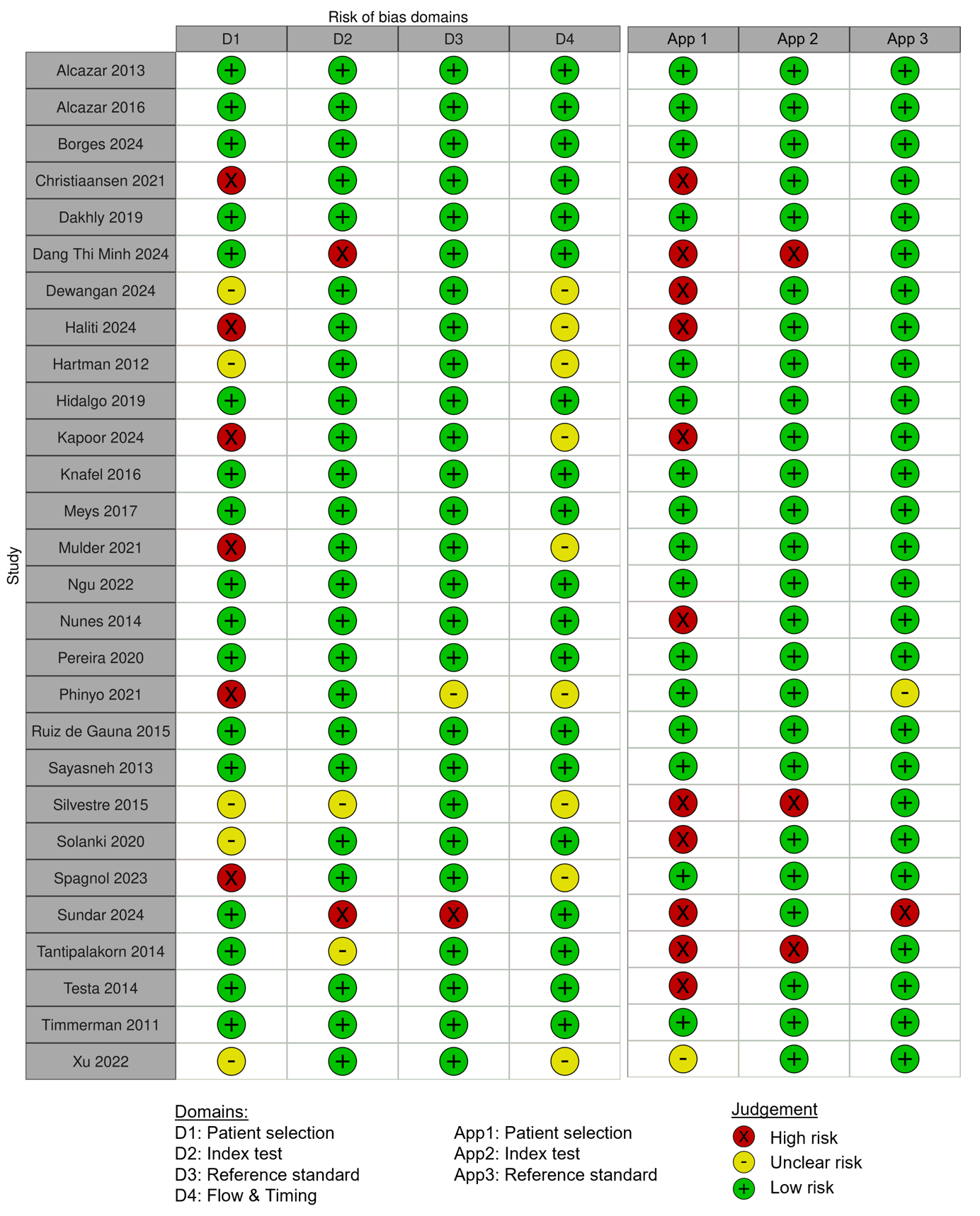
*

*S6.6, Quality assessment of all included studies regarding the Simple Rules + Subjective Assessment strategy*

*
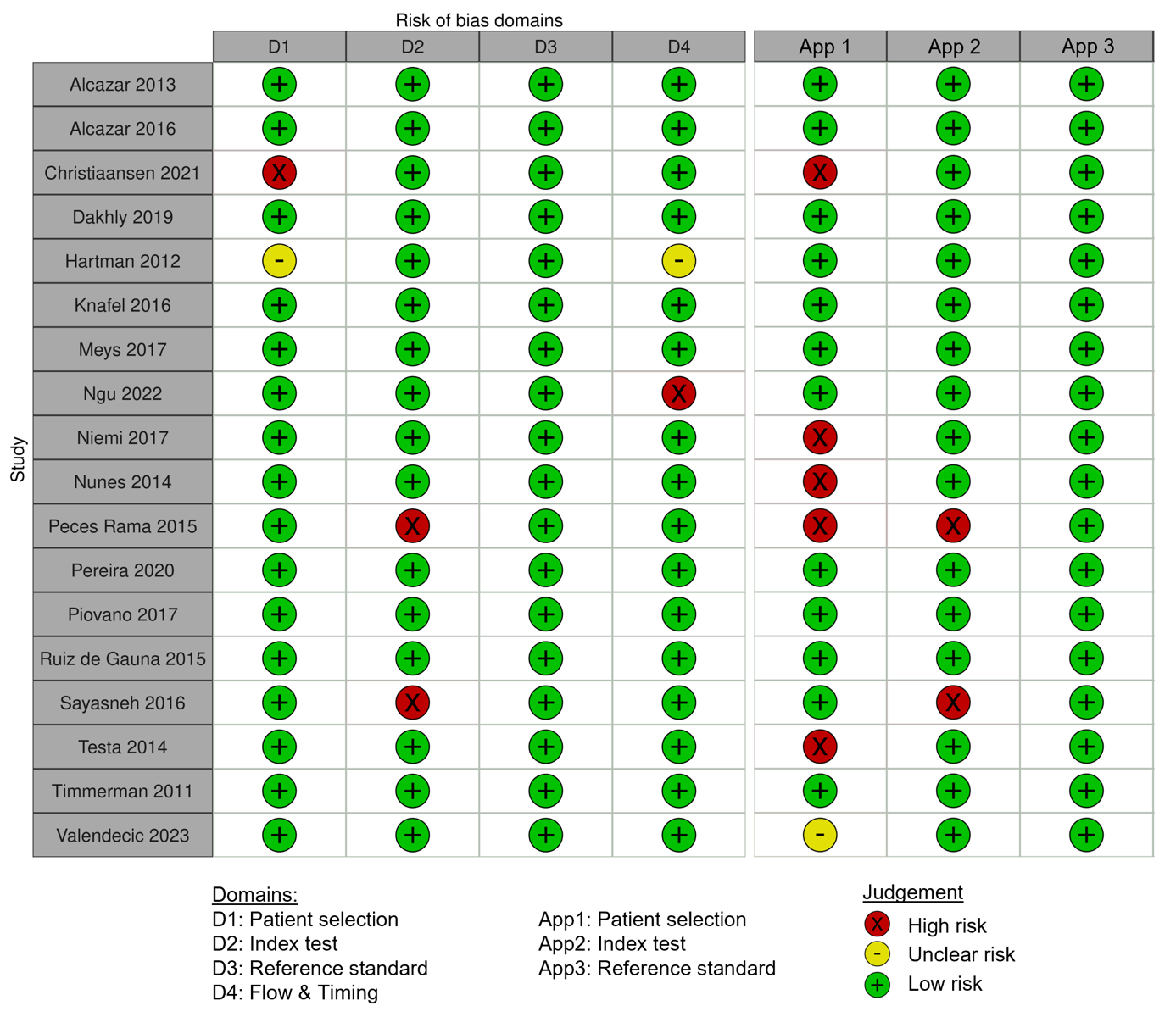
*

*S6.7, Quality assessment of all included studies regarding the ADNEX model*

*
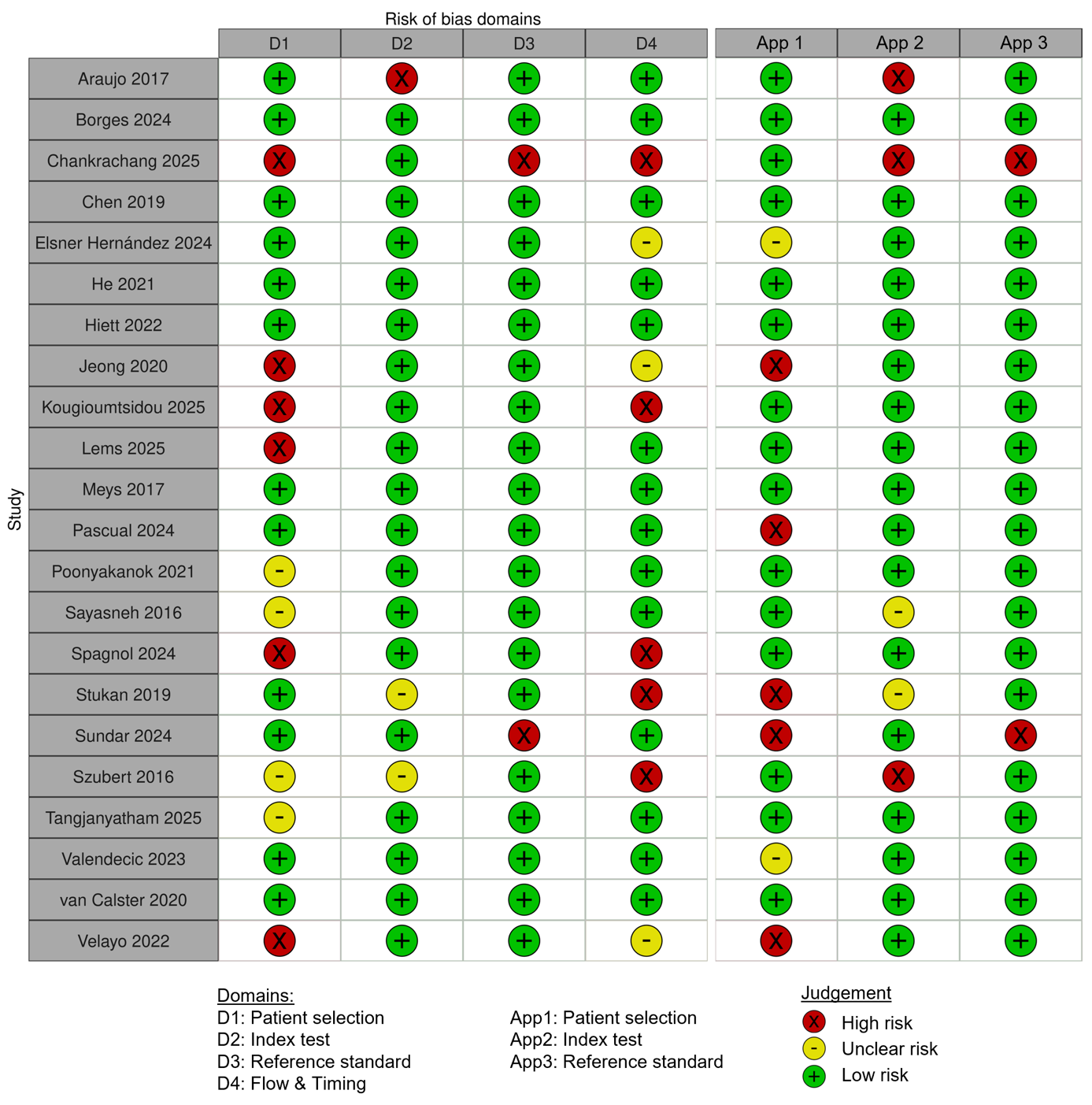
*

*S6.8, Quality assessment of all included studies regarding Subjective Assessment*

*
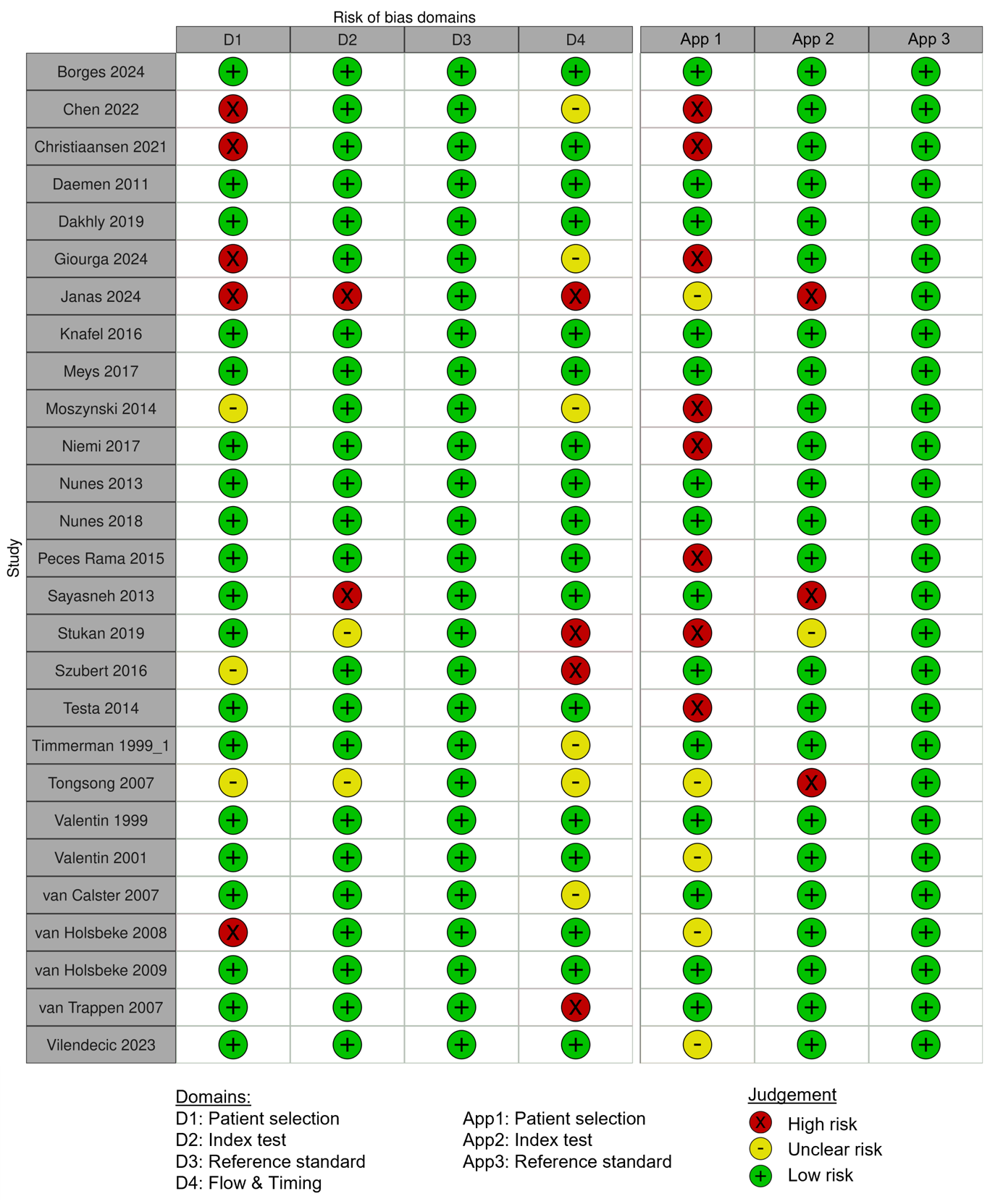
*

**Table S7** Meta-regression analysis for pre- *vs* postmenopausal women

*S7.1 Meta-regression analysis for all models with ≥3 studies for subgroup of premenopausal women*

|  | **RMI 2 – 200** | **LR2 – 10%** | **SR + Mal** | **SR + SA** | **ADNEX – 10%** | **SA** |
| --- | --- | --- | --- | --- | --- | --- |
| **RMI 1 – 200** | Sens:  p = 0.729  Spec:  p = 0.310 | Sens:  p < 0.001  Spec:  p = 0.024 | Sens:  p < 0.001  Spec:  p = 0.001 | Sens:  p < 0.001  Spec:  p = 0.315 | Sens:  p < 0.001  Spec:  p < 0.000 | Sens:  p < 0.001  Spec:  p = 0.839 |
| **RMI 2 – 200** |  | Sens:  p < 0.001  Spec  p = 0.324 | Sens:  p < 0.001  Spec:  p = 0.065 | Sens:  p < 0.001  Spec:  p = 0.081 | Sens:  p < 0.001  Spec:  p = 0.036 | Sens:  p< 0.001  Spec:  p = 0.261 |
| **LR2 – 10%** |  |  | Sens:  p < 0.001  Spec:  p = 0.241 | Sens:  p = 0.041  Spec:  p = 0.005 | Sens:  p = 0.070  Spec:  p = 0.241 | Sens:  p = 0.200  Spec:  p = 0.022 |
| **SR + Mal** |  |  |  | Sens:  p = 0.080  Spec:  p < 0.001 | Sens:  p = 0.114  Spec:  p = 0.855 | Sens:  p = 0.013  Spec:  p = 0.001 |
| **SR + SA** |  |  |  |  | Sens:  p = 0.992  Spec:  p < 0.001 | Sens:  p = 0.451  Spec:  p = 0.439 |
| **ADNEX – 10%** |  |  |  |  |  | Sens:  p = 0.494  Spec:  p < 0.001 |

*All displayed values are p-values. Statistically significant differences in methods are displayed in color: a green color indicates a higher sensitivity or specificity of the model in the column compared to the model in the row, a red color indicates a lower sensitivity or specificity of the model in the column compared to the model in the row.*

*S7.2 Meta-regression analysis for all models with ≥3 studies for subgroup of postmenopausal women*

|  | **RMI 2 – 200** | **LR2 – 10%** | **SR + Mal** | **SR + SA** | **ADNEX – 10%** | **SA** |
| --- | --- | --- | --- | --- | --- | --- |
| **RMI 1 – 200** | Sens:  p = 0.308  Spec:  p = 0.026 | Sens:  p = 0.101  Spec  p = 0.001 | Sens:  p < 0.000  Spec:  p = 0.011 | Sens:  p = 0.014  Spec:  p = 0.491 | Sens:  p < 0.001  Spec:  p < 0.001 | Sens:  p < 0.001  Spec:  p = 0.631 |
| **RMI 2 – 200** |  | Sens:  p = 0.667  Spec  p = 0.410 | Sens:  p = 0.036  Spec:  p = 0.914 | Sens:  p = 0.280  Spec:  p = 0.012 | Sens:  p = 0.006  Spec:  p = 0.062 | Sens:  p = 0.068  Spec:  p = 0.090 |
| **LR2 – 10%** |  |  | Sens:  p = 0.077  Spec:  p = 0.443 | Sens:  p = 0.492  Spec:  p < 0.001 | Sens:  p = 0.014  Spec:  p = 0.280 | Sens:  p = 0.144  Spec:  p = 0.006 |
| **SR + Mal** |  |  |  | Sens:  p = 0.273  Spec:  p = 0.005 | Sens:  p = 0.495  Spec:  p = 0.059 | Sens:  p = 0.661  Spec:  p = 0.052 |
| **SR + SA** |  |  |  |  | Sens:  p = 0.074  Spec:  p < 0.001 | Sens:  p = 0.462  Spec:  p = 0.295 |
| **ADNEX – 10%** |  |  |  |  |  | Sens:  p = 0.243  Spec:  p < 0.001 |

*All displayed values are p-values. Statistically significant differences in methods are displayed in color: a green color indicates a higher sensitivity or specificity of the model in the column compared to the model in the row, a red color indicates a lower sensitivity or specificity of the model in the column compared to the model in the row.*

**Table S8** Meta-regression analysis for studies with low (<21.1%) *vs* high (≥21.1%) prevalence of ovarian cancer

*S8.1 Meta-regression analysis for all models with ≥3 studies with low (<21.1%)* prevalence

|  | **RMI 2 – 200** | **RMI 3 – 200** | **LR2 – 10%** | **SR + Mal** | **SR + SA** | **ADNEX – 5%** | **ADNEX – 10%** | **ADNEX – 20%** |
| --- | --- | --- | --- | --- | --- | --- | --- | --- |
| **RMI 1 – 200** | Sens:  p = 0.068  Spec  p = 0.090 | Sens:  p = 0.498  Spec:  p = 0.220 | Sens:  p < 0.001  Spec:  p = 0.257 | Sens:  p < 0.001  Spec:  p = 0.104 | Sens:  p < 0.001  Spec:  p = 0.954 | Sens:  p < 0.001  Spec:  p = 0.010 | Sens:  p = 0.001  Spec:  p = 0.078 | Sens:  p = 0.128  Spec:  p = 0.808 |
| **RMI 2 – 200** |  | Sens:  p = 0.406  Spec:  p = 0.801 | Sens:  p = 0.010  Spec:  p = 0.728 | Sens:  p < 0.001  Spec:  p = 0.883 | Sens:  p = 0.006  Spec:  p = 0.095 | Sens:  p < 0.001  Spec:  p = 0.321 | Sens:  p = 0.070  Spec:  p = 0.959 | Sens:  p = 0.910  Spec:  p = 0.066 |
| **RMI 3 – 200** |  |  | Sens:  p = 0.003  Spec:  p = 0.932 | Sens:  p < 0.001  Spec:  p = 0.892 | Sens:  p = 0.002  Spec:  p = 0.218 | Sens:  p < 0.001  Spec:  p = 0.271 | Sens:  p = 0.022  Spec:  p = 0.766 | Sens:  p = 0.500  Spec:  p = 0.165 |
| **LR2 – 10%** |  |  |  | Sens:  p = 0.956  Spec:  p = 0.815 | Sens:  p = 0.784  Spec:  p = 0.253 | Sens:  p = 0.665  Spec:  p = 0.233 | Sens:  p = 0.195  Spec:  p = 0.694 | Sens:  p = 0.011  Spec:  p = 0.194 |
| **SR + Mal** |  |  |  |  | Sens:  p = 0.685  Spec:  p = 0.110 | Sens:  p = 0.620  Spec:  p = 0.240 | Sens:  p = 0.076  Spec:  p = 0.840 | Sens:  p = 0.001  Spec:  p = 0.077 |
| **SR + SA** |  |  |  |  |  | Sens:  p = 0.376  Spec:  p = 0.012 | Sens:  p = 0.236  Spec:  p = 0.095 | Sens:  p = 0.008  Spec:  p = 0.858 |
| **ADNEX – 5%** |  |  |  |  |  |  | Sens:  p = 0.012  Spec:  p = 0.341 | Sens:  p < 0.001  Spec:  p = 0.008 |
| **ADNEX – 10%** |  |  |  |  |  |  |  | Sens:  p = 0.081  Spec:  p = 0.058 |

*All displayed values are p-values. Statistically significant differences in methods are displayed in color: a green color indicates a higher sensitivity or specificity of the model in the column compared to the model in the row, a red color indicates a lower sensitivity or specificity of the model in the column compared to the model in the row.*

*S8.2 Meta-regression analysis for all models with ≥3 studies with high (≥21.1%) prevalence*

|  | **RMI 1 – 250** | **RMI 2 – 200** | **RMI 3 – 200** | **LR2 – 10%** | **SR + Mal** | **SR + SA** | **ADNEX – 5%** | **ADNEX – 10%** | **ADNEX – 20%** | **ADNEX – 30%** | **SA** |
| --- | --- | --- | --- | --- | --- | --- | --- | --- | --- | --- | --- |
| **RMI 1 – 200** | Sens:  p = 0.869  Spec:  p = 0.564 | Sens:  p = 0.326  Spec  p = 0.011 | Sens:  p = 0.771  Spec:  p = 0.655 | Sens:  p < 0.001  Spec:  p < 0.001 | Sens:  p < 0.001  Spec:  p < 0.001 | Sens:  p < 0.001  Spec:  p = 0.847 | Sens:  p < 0.001  Spec:  p < 0.001 | Sens:  p < 0.001  Spec:  p < 0.001 | Sens:  p < 0.001  Spec:  p = 0.014 | Sens:  p = 0.062  Spec:  p = 0.588 | Sens:  p < 0.001  Spec:  p = 0.409 |
| **RMI 1 – 250** |  | Sens:  p = 0.574  Spec:  p = 0.020 | Sens:  p = 0.910  Spec:  p = 0.425 | Sens:  p < 0.001  Spec:  p = 0.002 | Sens:  p < 0.001  Spec:  p < 0.001 | Sens:  p < 0.001  Spec:  p = 0.679 | Sens:  p < 0.001  Spec:  p < 0.001 | Sens:  p < 0.001  Spec:  p = 0.001 | Sens:  p < 0.001  Spec:  p = 0.020 | Sens:  p = 0.155  Spec:  p = 0.987 | Sens:  p < 0.001  Spec:  p = 0.953 |
| **RMI 2 – 200** |  |  | Sens:  p = 0.689  Spec:  p = 0.195 | Sens:  p < 0.001  Spec:  p = 0.413 | Sens:  p < 0.001  Spec:  p = 0.047 | Sens:  p < 0.001  Spec:  p = 0.013 | Sens:  p < 0.001  Spec:  p < 0.001 | Sens:  p < 0.001  Spec:  p = 0.033 | Sens:  p < 0.001  Spec:  p = 0.851 | Sens:  p = 0.287  Spec:  p = 0.031 | Sens:  p < 0.001  Spec:  p = 0.001 |
| **RMI 3 – 200** |  |  |  | Sens:  p < 0.001  Spec:  p = 0.051 | Sens:  p < 0.001  Spec:  p = 0.004 | Sens:  p < 0.001  Spec:  p = 0.584 | Sens:  p < 0.001  Spec:  p < 0.001 | Sens:  p < 0.001  Spec:  p = 0.003 | Sens:  p < 0.001  Spec:  p = 0.171 | Sens:  p = 0.211  Spec:  p = 0.443 | Sens:  p < 0.001  Spec:  p = 0.351 |
| **LR2 – 10%** |  |  |  |  | Sens:  p = 0.384  Spec:  p = 0.271 | Sens:  p = 0.588  Spec:  p = 0.001 | Sens:  p < 0.001  Spec:  p = 0.001 | Sens:  p = 0.019  Spec:  p = 0.192 | Sens:  p = 0.782  Spec:  p = 0.586 | Sens:  p = 0.011  Spec:  p = 0.005 | Sens:  p = 0.639  Spec:  p < 0.001 |
| **SR + Mal** |  |  |  |  |  | Sens:  p = 0.133  Spec:  p < 0.001 | Sens:  p = 0.001  Spec:  p = 0.004 | Sens:  p = 0.092  Spec:  p = 0.753 | Sens:  p = 0.300  Spec:  p = 0.127 | Sens:  p = 0.001  Spec:  p < 0.001 | Sens:  p = 0.630  Spec:  p < 0.001 |
| **SR + SA** |  |  |  |  |  |  | Sens:  p < 0.001  Spec:  p < 0.001 | Sens:  p = 0.003  Spec:  p < 0.001 | Sens:  p = 0.853  Spec:  p = 0.016 | Sens:  p = 0.030  Spec:  p = 0.692 | Sens:  p = 0.270  Spec:  p = 0.599 |
| **ADNEX – 5%** |  |  |  |  |  |  |  | Sens:  p = 0.046  Spec:  p = 0.013 | Sens:  p < 0.001  Spec:  p < 0.001 | Sens:  p < 0.001  Spec:  p < 0.001 | Sens:  p < 0.001  Spec:  p < 0.001 |
| **ADNEX – 10%** |  |  |  |  |  |  |  |  | Sens:  p = 0.021  Spec:  p = 0.090 | Sens:  p < 0.001  Spec:  p < 0.001 | Sens:  p = 0.030  Spec:  p < 0.001 |
| **ADNEX – 20%** |  |  |  |  |  |  |  |  |  | Sens:  p = 0.035  Spec:  p = 0.030 | Sens:  p = 0.486  Spec:  p = 0.002 |
| **ADNEX – 30%** |  |  |  |  |  |  |  |  |  |  | Sens:  p = 0.002  Spec:  p = 0.941 |

*All displayed values are p-values. Statistically significant differences in methods are displayed in color: a green color indicates a higher sensitivity or specificity of the model in the column compared to the model in the row, a red color indicates a lower sensitivity or specificity of the model in the column compared to the model in the row.*

**Appendix S1** QUADAS-2 and QUADAS-C tools

**QUADAS-2 AND QUADAS-C TOOL**

The Quality Assessment of Diagnostic Accuracy Studies (QUADAS)-2 tool is a standardized tool recommended by Cochrane to evaluate the quality of diagnostic accuracy studies (1). The QUADAS-C tool is an extension to assess the risk of bias in comparative diagnostic test accuracy studies (2).

For the purposes of this review, we will define the following terms. This document is based on Appendix C of the article of *Meys et al,* 2016 (3).

***Patient selection***

A. Risk of bias

Could the selection of patients have introduced bias?

Signaling questions:

1. Describe methods of patient selection
   1. Describe how patients were selected, either prospectively or retrospectively.
2. Was a consecutive or random sample of patients enrolled?
   1. Yes: the study clearly states that either a consecutive or a random sample of patients is enrolled.
   2. No: the study specifically states that a non-consecutive sample of patients was used, or this can be inferred from the study design.
   3. Unclear: If it is not clear, or if it is stated that patients are enrolled consecutively, but the study does not mention the number of eligible patients or the number of consented patients.
3. Was a case-control design avoided?

Studies using a case-control design were not included in the review because this study design

tend to overestimate diagnostic test performance. We therefore scored ‘Yes’ for all studies.

1. Did the study avoid inappropriate exclusions? *NB. this concerns the moment of patient selection!*
   1. Yes: the study avoided inappropriate exclusions, such as exclusion from patients who will be difficult to diagnose.
   2. No: inappropriate exclusions, such as not including patients who will be difficult to diagnose, are made.
   3. Unclear: it is not possible to deduct from the report if inappropriate exclusions are made.
      - Note: In case a study did include a large study group without mentioning restrictions, it can be deducted that inappropratie exclusions are avoided (as all type of patients are included). If however it is unclear if a selection took place, e.g. no exclusion criteria are mentioned, but there is the concern that the study population is a selection, the risk is judged ‘Unclear’.

*Risk of bias:*

- Low risk: at least two of the signaling questions are answered with ‘Yes’, it was a prospective study and there are no major concerns.
- High risk: at least two signaling questions are answered ‘No’; or two signaling questions are answered with ‘unclear’ in case of a retrospective study or in case of major concerns in a prospective study; or all signaling questions are answered differently (1 ‘Yes’, 1 ‘No’, 1 ‘Unclear’).
- Unclear: at least two signaling questions are answered with ‘Unclear’

B. Concerns regarding applicability

Is there concern that the included patients do not match the review question?

In general only studies were included in this review that met our inclusion criteria concerning participants (i.e. women diagnosed in a second or third line hospital with at least one adnexal mass that is suspected to be of adnexal origin and are scheduled for surgery in order to obtain a final histological diagnosis). This group of participants will represent the patients in whom the test will be used in clinical practice very well and therefore the concerns regarding applicability is in general considered ‘LOW’ in included studies.

However, if the study population does not resemble the normal distribution of patients, i.e.

- the study investigated only women of a certain age or menopausal status;
- the study only investigated patients in one specific FIGO-stage; or
- the prevalence of malignancy in the study is <10 or ≥ 40%;

-> the applicability is scored ‘high concern’.

Although settings with prevalences below 10% and above 40% do occur, such extremes may not reflect the average patient mix encountered in general practice.

If no baseline characteristics or other descriptive information is available concerning the patient population, e.g. lack of information on patients' age and menopausal status, the applicability is scored 'unclear'.

***Index test***

A. Risk of bias

Could the conduct or interpretation of the index test have introduced bias?

Signaling questions:

1. Describe the index test and how it was conducted and interpreted. (if more than index test was used, describe all).
   1. For the risk of bias judgement of this item, this can be judged as a ‘Yes’, ‘No’ or ‘Unclear’ based on the following.
      - *A. Yes:*
      - the expertise of the performing clinician(s) was recorded, especially when the study is about subjective assessment;
      - it is clearly stated which version of the RMI is used or this can be referred, if the study examines the diagnostic accuracy of RMI;
      - It is clearly stated what was considered a ‘positive’ result.
      - *B. No:*

the following items are not specified and cannot be referred from the study design: the expertise of the performing clinician(s) performing subjective assessment, the version of the RMI used and what is considered a ‘positive’ result.

- - - *c. Unclear:*

One or more of the following items are not specified and cannot be referred from the study design: the expertise of the performing clinician(s) performing ultrasound, especially in the case of subjective assessment, the version of the RMI used and what is considered a ‘positive’ result.

1. Were the index results interpreted without knowledge of the results of the reference test?
   1. *a. Yes:*

For all prospective studies.

For all retrospective studies: the study clearly states that assessment of the index test results is blinded to the results of histology.

- 1. *b. No:*

For retrospective studies: the study clearly states that assessment of the index test results is not blinded to histology.

- 1. *c. Unclear:*

For retrospective studies: the study does not state if the assessment of the index test is blinded to the results of histology and this cannot be inferred either.

1. If a threshold was used, was it pre-specified?
   1. *a. Yes*:

the study either investigates one or more tests with a threshold for diagnosing malignancy (i.e. RMI, LR2 and ADNEX) and this threshold is pre-specified, or the study does not include tests that use a threshold (i.e. simple rules and subjective assessment), or both.

- 1. *b. No:*

the study investigates one or more tests with a threshold for diagnosing malignancy (RMI, LR2 and ADNEX) and this threshold is not pre-specified.

- 1. *c. Unclear:*

insufficient data were reported to permit a judgment.

Risk of bias:

- Low risk: The conduction and interpretation of the index test is clear. In general this is the case for most included studies, as for the calculation of all index tests included in this study a protocol needs to be followed. Because of the nature of the study, not pre-specifying the threshold is not considered a major concern for this study.
- High risk: the conduction and interpretation for the index test is for all items unclear or high risk and likely to have introduced bias.
- Unclear: the conduction and interpretation of the index test are unclear.

NB. Especially for index tests using ‘subjective assessment’ and to a latter extent for the IOTA models the conduction and interpretation of the index test is important, as the RMI is less dependent on the operator than the other models.

B. Concerns regarding applicability
Are there concerns that the index test, its conduct, or its interpretation differ from the review question?

- Low: the conduct or interpretation of the index test probably does not differ from the way it is likely to be used in clinical practice, i.e. sonographers performing subjective assessment had level III experience according to the guidelines of the European Federation of Societies for Ultrasound in Medicine and Biology and transvaginal ultrasound was mostly used.
- High: the conduct or interpretation of the index test will probably differ from the way it is likely to be used in clinical practice.
- Unclear: it is unclear of the conduction and interpretation of the index test differ from the way it is used in clinical practice.

Being an expert in gynaecological ultrasound is defined by the guidelines of the European Federation of Societies for Ultrasound in Medicine and Biology.1 According to this guideline experts (Level III examiners) have to work in tertiary referral centers, have an academic record and a high level of experience and expertise. In articles not defining the level of expertise by EFSUMB guidelines, we will consider ultrasonographers with at least 10 years of experience, who are working in a tertiary referral center, as Level III experts for subjective assessment.

***Reference standard***

A. Risk of Bias

Could the reference standard, its conduct, or its interpretation have introduced bias?

Signaling questions:

1. Describe the reference standard and how it was conducted and interpretated
   1. Shortly describe reference standard
2. Is the reference standard likely to correctly classify the target condition?
   1. Yes: histology is used as reference standard and/or follow-up is used in case of an ovarian tumor that is considered benign. A condition is that follow-up has taken place for at least one year with the selection of the follow-up group and method of follow-up clearly described and in accordance with current guidelines.
   2. No: follow-up is (partly) used as reference standard, but selection and follow-up of this group is not clearly described and differs from current guidelines.
   3. Unclear: the reference standard is not specified or cannot be inferred.
3. Were the reference standard results interpreted without knowledge of the results of the index test?
   1. Yes: the study clearly states that assessment of histology is blinded to the index test results.
   2. No: the study clearly states that assessment of histology is not blinded to the index test results.
   3. Unclear: the study does not state if the assessment of histology is blinded to the index test results and this cannot be inferred either.

Risk of bias:

- Low risk: all of the signaling questions are answered with ‘Yes’ or one of the questions is answered ‘Yes’ and the other question is answered ‘Unclear’ .
- High risk: at least one signaling question is answered ‘No’.
- Unclear: both signaling questions are answered with ‘Unclear’.

B. Concerns regarding applicability

Is there concern that the target condition as defined by the reference standard does not match the review question?
We judged applicability to be mostly scored ‘low concern’ for all studies, because studies not using histology as a reference standard or studies including follow-up for < 1 year, were not included in this review.
However if a study did use follow-up as a reference standard and if follow-up was not conducted for at least one year, including at least one clinical consultation beyond one year after the initial ultrasound, the applicability is scored ‘high concern’.

***Flow and timing***

A. Risk of bias

Could the patient flow have introduced bias?

Signaling questions:

1. Was there an appropriate interval between index test and reference standard?
   1. Yes: if the time period between reference standard and index test is less than 120 days.
   2. No: the interval between the index test and reference standard is more than 120 days.
   3. Unclear: the interval between index test and reference standard is not stated and cannot be inferred.
2. Did all patients receive a reference standard?
   1. Yes: all patients did receive either surgery or follow-up
   2. No: not all patients did receive either surgery or follow-up
   3. Unclear: it is unclear whether all patients did receive a reference standard
3. Did all patients receive the same reference standard?
   1. Yes: all patients had histology as reference standard.
   2. No: different reference standards were used, for example histology and follow-up, or not all patients received a reference standard.
   3. Unclear: the study does not state if all patients received the same reference standard.
4. For comparative studies, did all patients receive all index tests?
   1. Yes: all patients did receive all index tests
   2. No: not all patients did receive all index tests
   3. Unclear: it is unclear whether all patients did receive all index tests.
5. Were all patients included in the analysis?
   1. Yes: All participants recruited in the study are included in the analysis. Loss of patients for which no test result or reference test outcome could be obtained is recorded and explained.
   2. No: Not all participants recruited in the study are included in the analysis. Loss of patients for which no test result or reference test outcome could be obtained is not recorded and explained.
   3. Unclear: the study does not state whether all participants recruited in the study are included in the analysis and this cannot be referred either. It is unclear if there is a loss of patients for which no test result or reference test outcome could be obtained.

Risk of bias:

- Low risk: at least two of the signaling questions are answered with ‘Yes’.
- High risk: at least two signaling questions are answered ‘No’, or at least one signaling question is answered with ‘No’ and at least two are answered with ‘Unclear’ and there are major concerns, or all signaling questions are answered differently (one with ‘Yes’, one with ‘No’, one with ‘Unclear’).
- Unclear: at least two signaling questions are answered with ‘Unclear’.

**QUADAS-C TOOL**

The QUADAS-C tool is applicated to all studies that investigated the diagnostic accuracy of different risk-assessment strategies. As in general all included studies did test apply all risk-assessment strategies to all patients, no additional risk is introduced.

If a study investigated multiple risk-assessment strategies but only one strategy is included in the systematic review, the QUADAS-C is not applied and the study is judged in general for this specific risk-assessment strategy with the QUADAS-2.

***Patient selection***

- - - Was the risk of bias for each index text judged ‘low’ for this domain?
      1. Yes: if the risk of bias for each index test was judged ‘low’ for this domain
      2. No: if the risk of bias for one or multiple index test(s) was judged ‘high’ for this domain
    - Was a fully paired or randomized design used?
      1. Yes: if all participants did undergo all index tests, which is in general the case for all included studies
      2. No: if not all participants did undergo all index tests
      3. Unclear: if it is not clear of all participants did undergo all index tests
    - Was the allocation sequence random?
      1. Only applicable to randomized designs, therefore ‘not applicable’ for all studies included in this systematic review.
    - Was the allocation sequence concealed until patients were enrolled and assigned to index tests?
      1. Only applicable to randomized designs, therefore ‘not applicable’ for all studies included in this systematic review.

RISK

Could the selection of patients have introduced bias in the comparison?

- Low risk: at least two of the signaling questions are answered with ‘Yes’
- High risk: at least two signaling questions are answered ‘No’
- Unclear: two signaling questions are answered with ‘Unclear’ or one with ‘No’ and one with ‘Unclear’.

***Index test***

1. Was the risk of bias for each index text judged ‘low’ for this domain?
   - - 1. Yes: if the risk of bias for each index test was judged ‘low’ for this domain
       2. No: if the risk of bias for one or multiple index test(s) was judged ‘high’ for this domain
2. Were the index test results interpreted without knowledge of the results of the other index test(s)? (only for randomized designs)
   1. Not applicable for this study, since no randomized studies are included.
3. Is undergoing one index test unlikely to affect the performance of the other index test(s)? (only for randomized designs).
   1. For all studies in this systematic review the tests will not influence each other. No randomized designs are included in this review. Therefore the answer to this question will be ‘Not applicable’ for all included studies.
4. Were the index tests conducted and interpreted without advantaging one of the test(s)?
   1. Yes/No/Unclear.
   2. In general, for all included studies the answer to this question will be ‘Yes’, because of the nature of the diagnostic tests included in this study. In all included studies, an ultrasound is performed, followed by all diagnostic tests at one time point. Therefore, this question is not applicable and is assessed as 'Yes'.

RISK

Could the conduct or interpretation of the index tests have introduced bias in the comparison?

- Low risk: at least two of the signaling questions are answered with ‘Yes’. It is unlikely that the comparison is influenced by the conduct or interpretation of the index test(s).
- High risk: at least two signaling questions are answered ‘No’ and it is likely that the knowledge of the result of one test has influenced the results of other Index test(s).
- Unclear: two signaling questions are answered with ‘Unclear’ or one with ‘No’ and one with ‘Unclear’.

***Reference standard***

1. Was the risk of bias for each index text judged ‘low’ for this domain?
   - - 1. Yes: if the risk of bias for each index test was judged ‘low’ for this domain
       2. No: if the risk of bias for one or multiple index test(s) was judged ‘high’ for this domain
     1. Did the reference standard avoid incorporating any of the index tests?
        1. Yes/No/Unclear.
        2. Because of the nature of the index and reference tests included in this systematic review, the answer will be ‘Yes’ for all included studies.

RISK

Could the reference standard, its conduct, or its interpretation have introduced bias in the comparison?

- Low risk: at least two of the signaling questions are answered with ‘Yes’. It is unlikely that the comparison is influenced by the conduct or interpretation of the index test(s).
- High risk: at least two signaling questions are answered ‘No’ and it is likely that the knowledge of the result of one test has influenced the results of other Index test(s).
- Unclear: two signaling questions are answered with ‘Unclear’ or one with ‘No’ and one with ‘Unclear’.
- In general this will be ‘LOW’ risk for all included studies because of the nature of the index and reference tests included in this systematic review.

***Flow and timing***

1. Was the risk of bias for each index text judged ‘low’ for this domain?
   - - 1. Yes: if the risk of bias for each index test was judged ‘low’ for this domain
       2. No: if the risk of bias for one or multiple index test(s) was judged ‘high’ for this domain
2. Was there an appropriate interval between the index tests?
   1. Because of the nature of the studies index tests, all index tests are performed during the same ultrasound. Therefore this is considered appropriate for this systematic review and all questions are answered with ‘Yes’.
3. Was the same reference standard used for all index tests?
   1. Yes: the same reference standard was used for all index tests. In case a study used both surgery and follow-up as reference standard, but this was similar for all index tests, this is considered as the same reference standard for all index tests.
   2. No: different reference standards were used for different index tests
   3. Unclear: it is unclear whether all index tests did have the same reference tests
4. Are the proportions and reasons for missing data similar across index tests?
   1. Yes: this is automatically the case if there is a fully-paired study design, in other words if all patients had all index tests. Reasons for missing data are similar across all index tests.
   2. No: Proportions and reasons for missing data are different across index tests.
   3. Unclear: It is not clear if proportions and reasons for missing data are similar across index tests.

RISK

Could the patient flow have introduced bias in the comparison?

- Low risk: at least two of the signaling questions are answered with ‘Yes’.
- High risk: at least two signaling questions are answered ‘No’.
- Unclear: two signaling questions are answered with ‘Unclear’ or one with ‘No’ and one with ‘Unclear’.

**References**

1. Penny F. Whiting AWSR, Marie E. Westwood, et al; the QUADAS-2 Group. QUADAS-2: A Revised Tool for the Quality Assessment of Diagnostic Accuracy Studies. Ann Intern Med. 2011;155(8):529-36.

2. Yang B MS, Takwoingi Y, Davenport CF, Hyde CJ, Whiting PF, Deeks JJ, Leeflang MMG; QUADAS-C Group†; Bossuyt PMM, Brazzelli MG, Dinnes J, Gurusamy KS, Jones HE, Lange S, Langendam MW, Macaskill P, McInnes MDF, Reitsma JB, Rutjes AWS, Sinclair A, de Vet HCW, Virgili G, Wade R, Westwood ME. QUADAS-C: A Tool for Assessing Risk of Bias in Comparative Diagnostic Accuracy Studies. Ann Intern Med. 2021 Nov;174(11):1592-9.

3.

Meys EM, Kaijser J, Kruitwagen RF, Slangen BF, Van Calster B, Aertgeerts B, et al. Subjective assessment versus ultrasound models to diagnose ovarian cancer: A systematic review and meta-analysis. Eur J Cancer. 2016;58:17-29.

**Figure S1** Prevalence and size of included studies

**
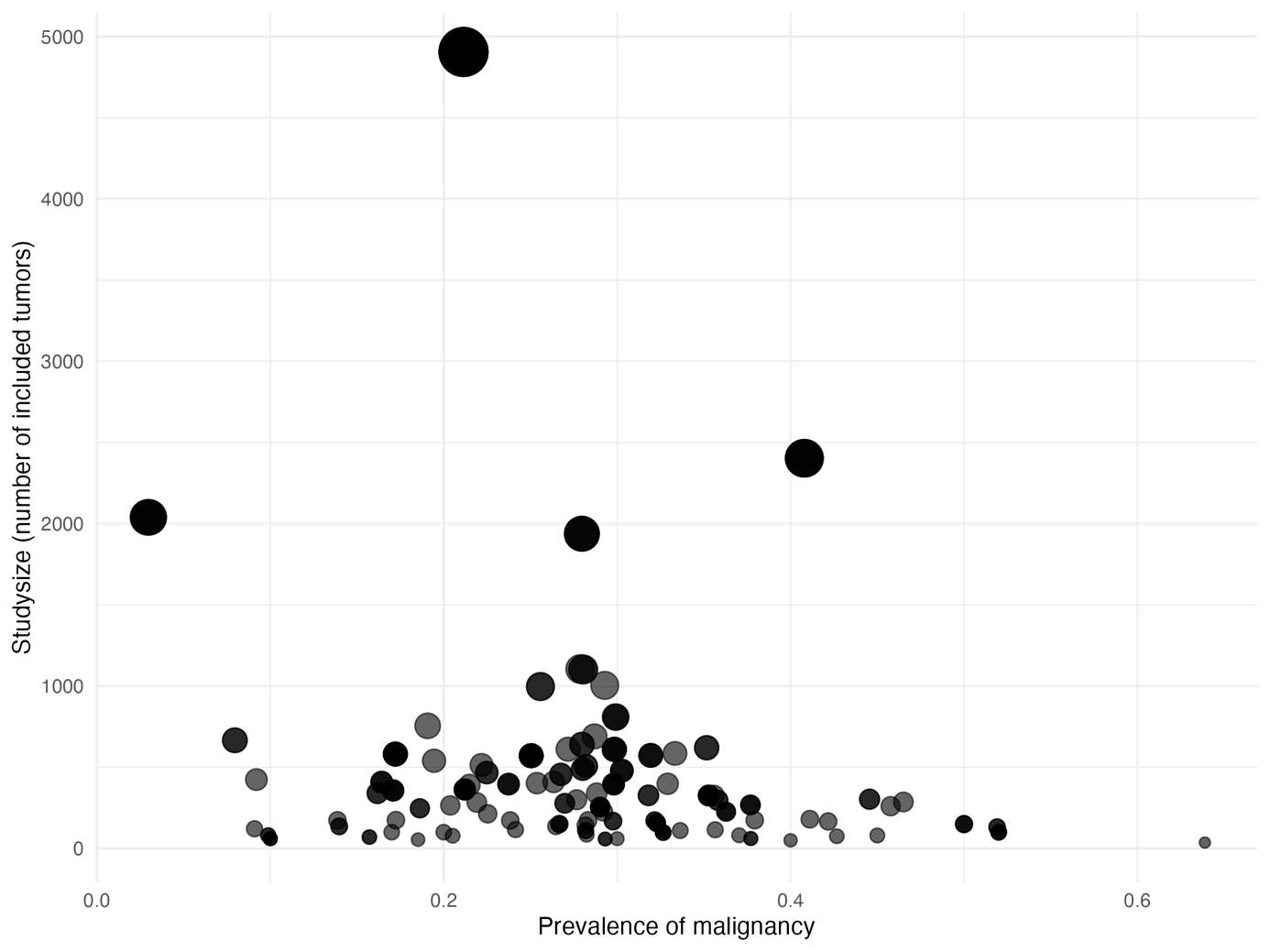
**

**Figure S2** Funnel plots for sensitivity and specificity.

*S2.1 Funnel plot for sensitivity, all models included*


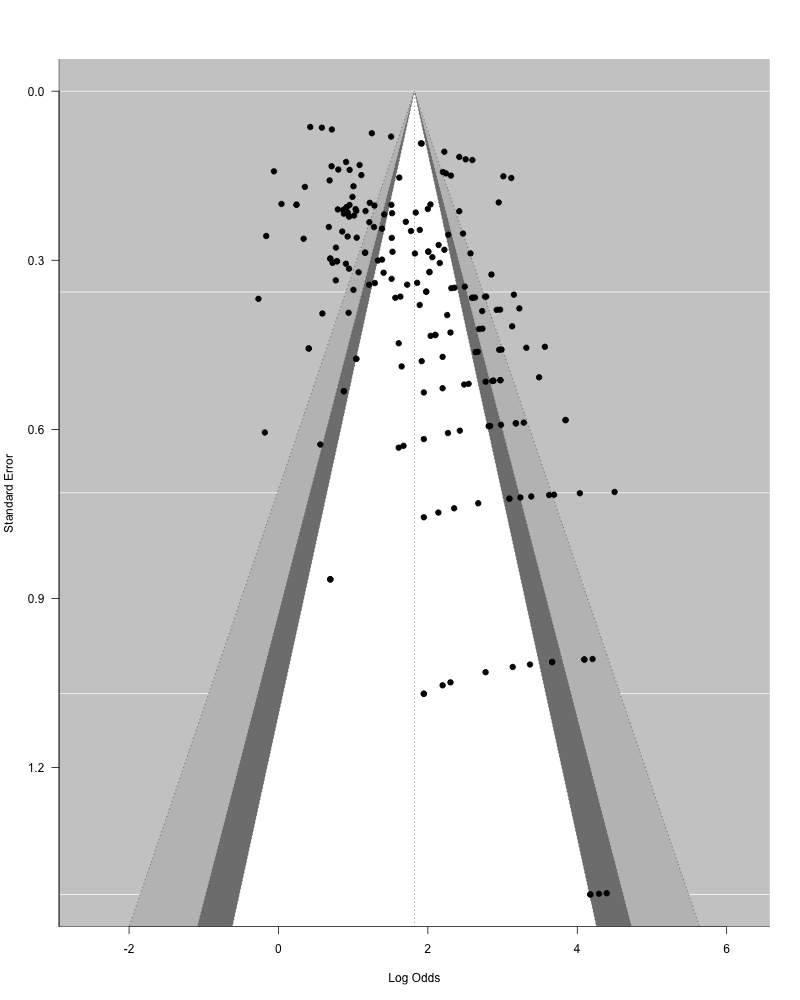


*S2.2 Funnel plot for specificity, all models included*

**
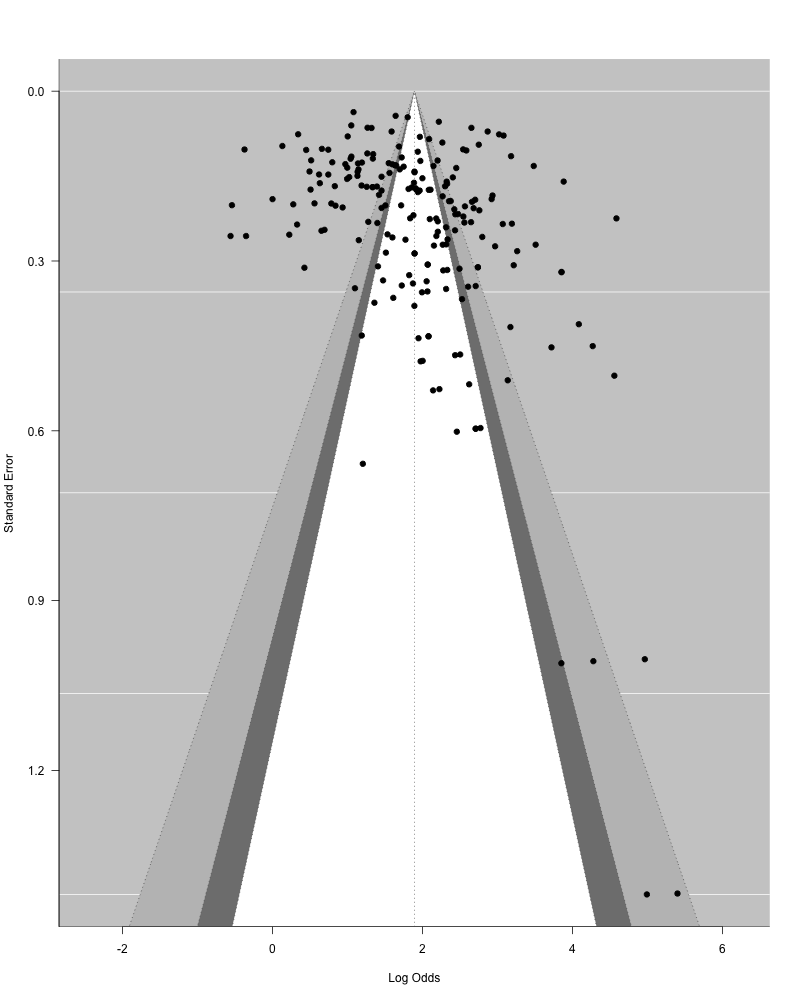
**

*S2.3 Funnel plot for sensitivity, RMI 1*

*
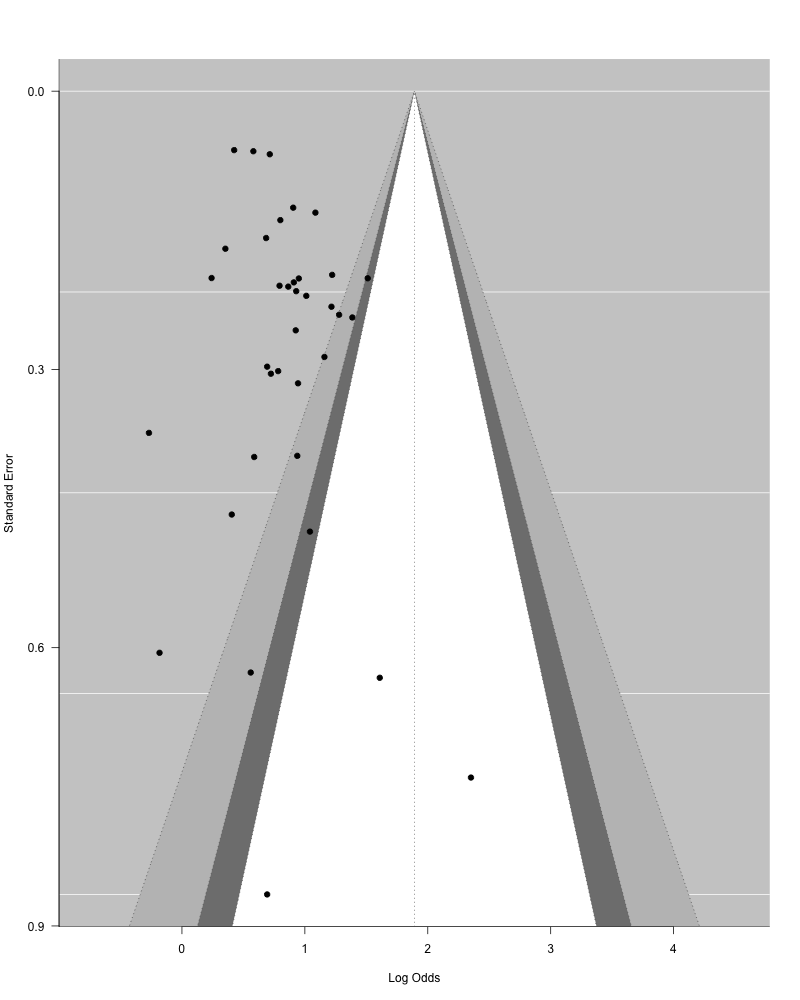
*

*S2.4 Funnel plot for specificity, RMI 1*

**
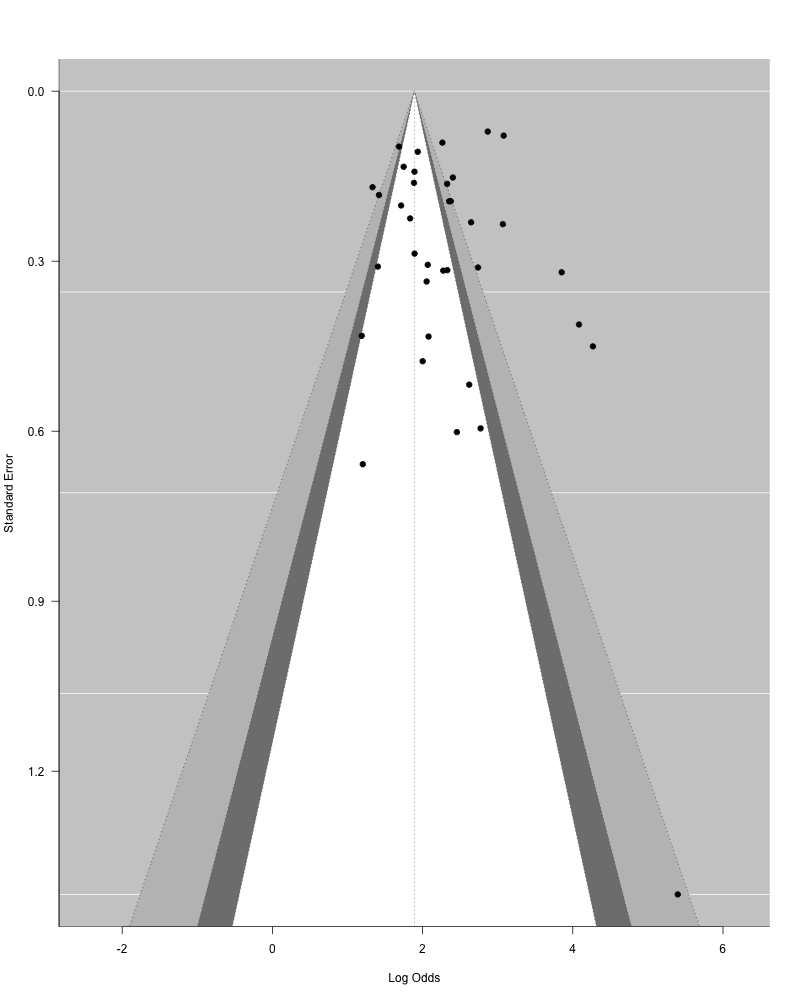
**

*S2.5 Funnel plot for sensitivity, RMI 2*

*
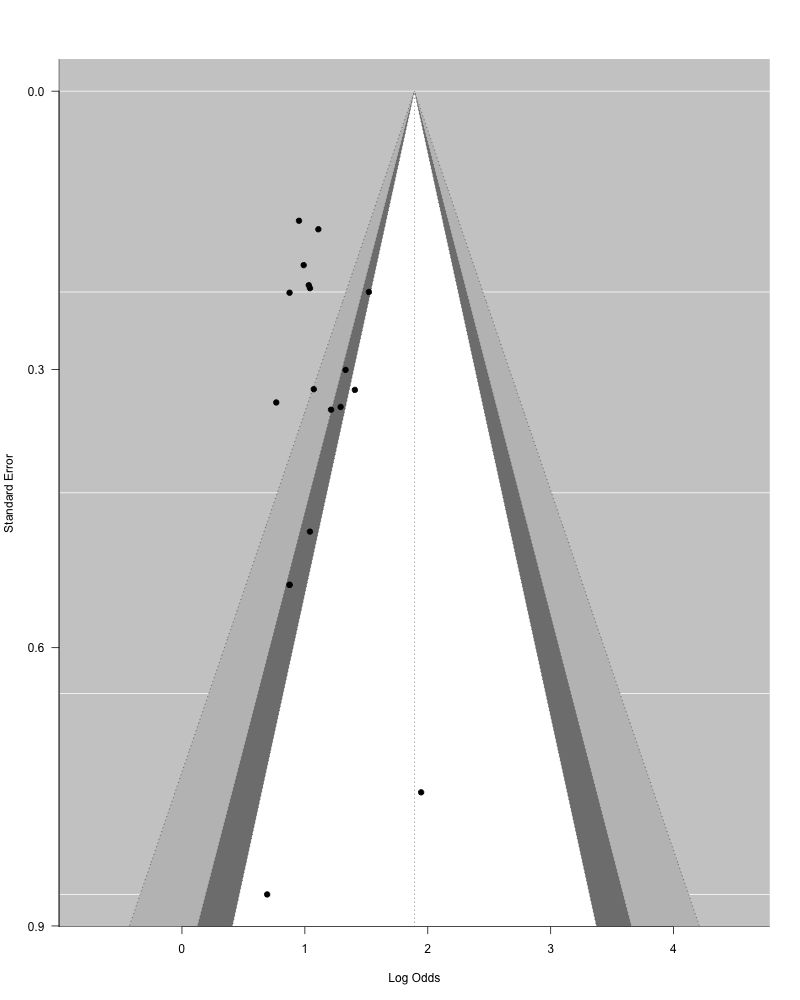
*

*S2.6 Funnel plot for specificity, RMI 2*

*
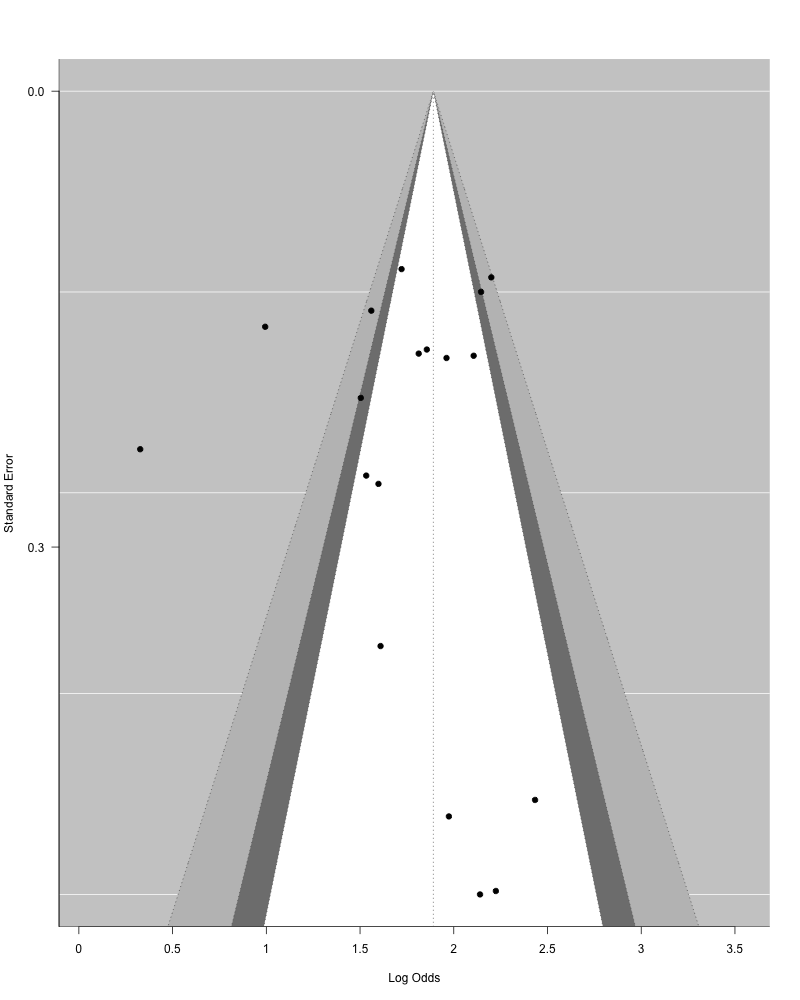
*

*S2.7 Funnel plot for sensitivity, RMI 3*

*
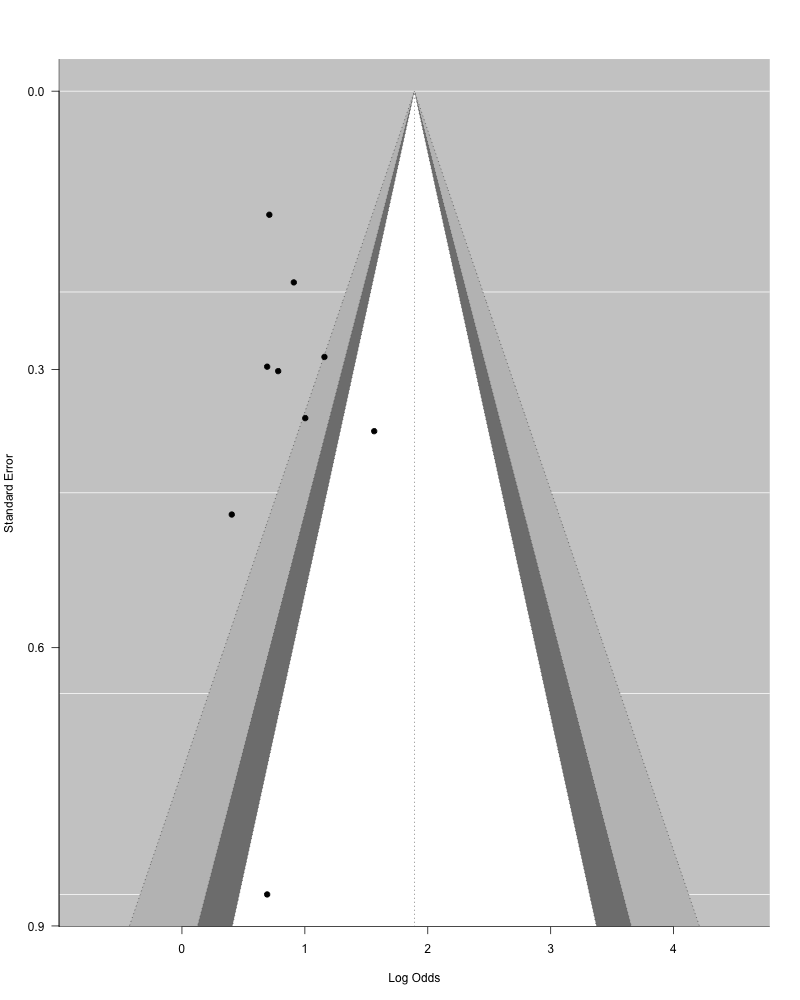
*

*S2.8 Funnel plot for specificity, RMI 3*

*
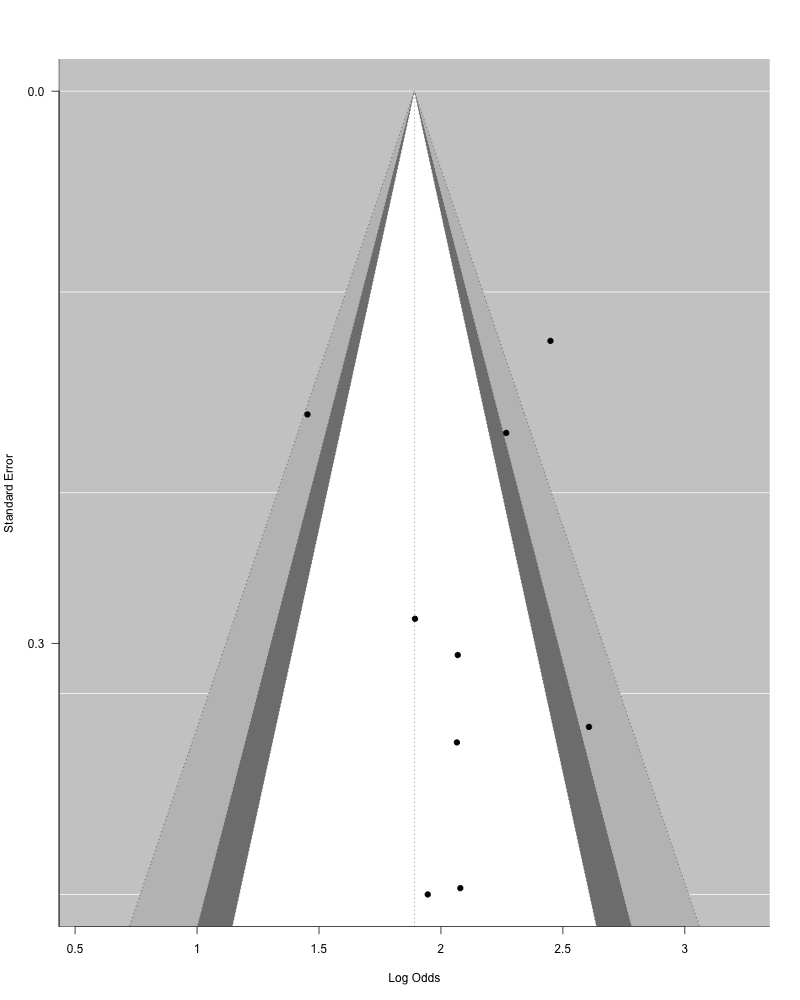
*

*S2.9 Funnel plot for sensitivity, LR2*

*
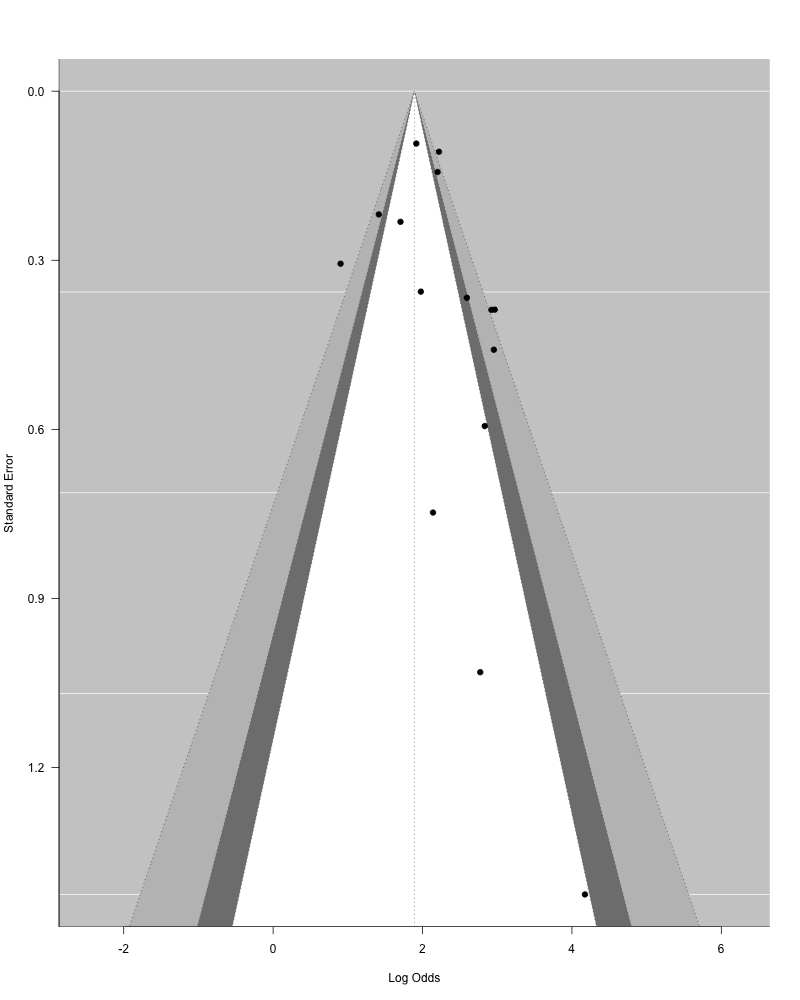
*

*S2.10 Funnel plot for specificity, LR2*

*
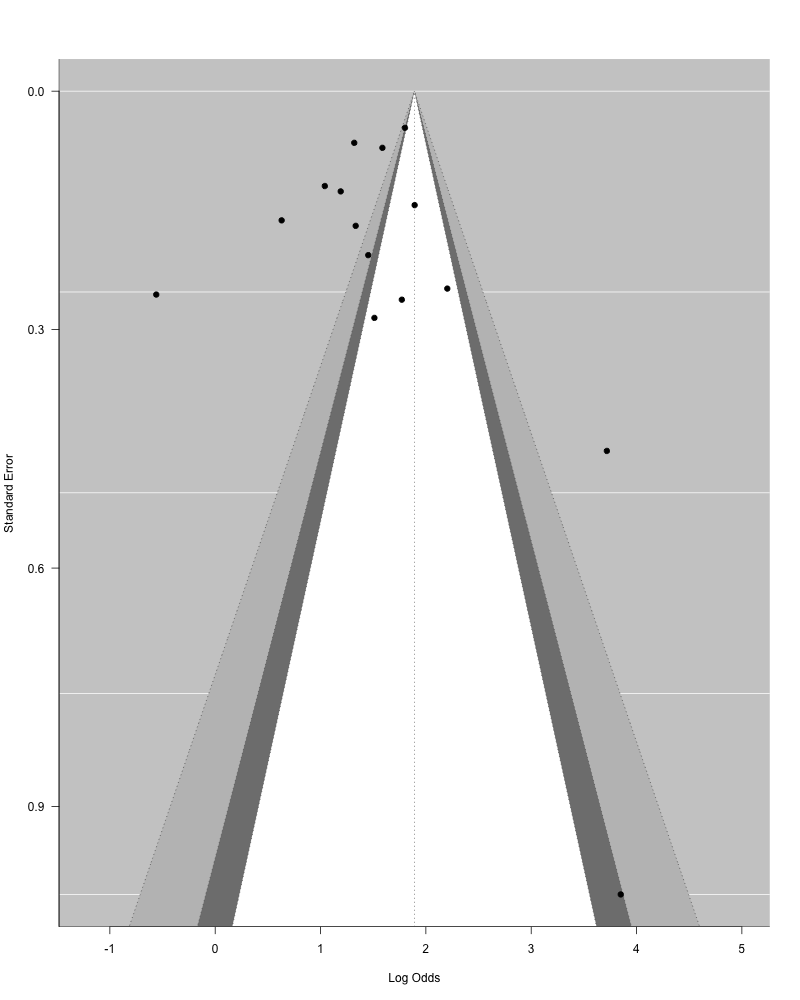
*

*S2.11 Funnel plot for sensitivity, SR + Mal*

*
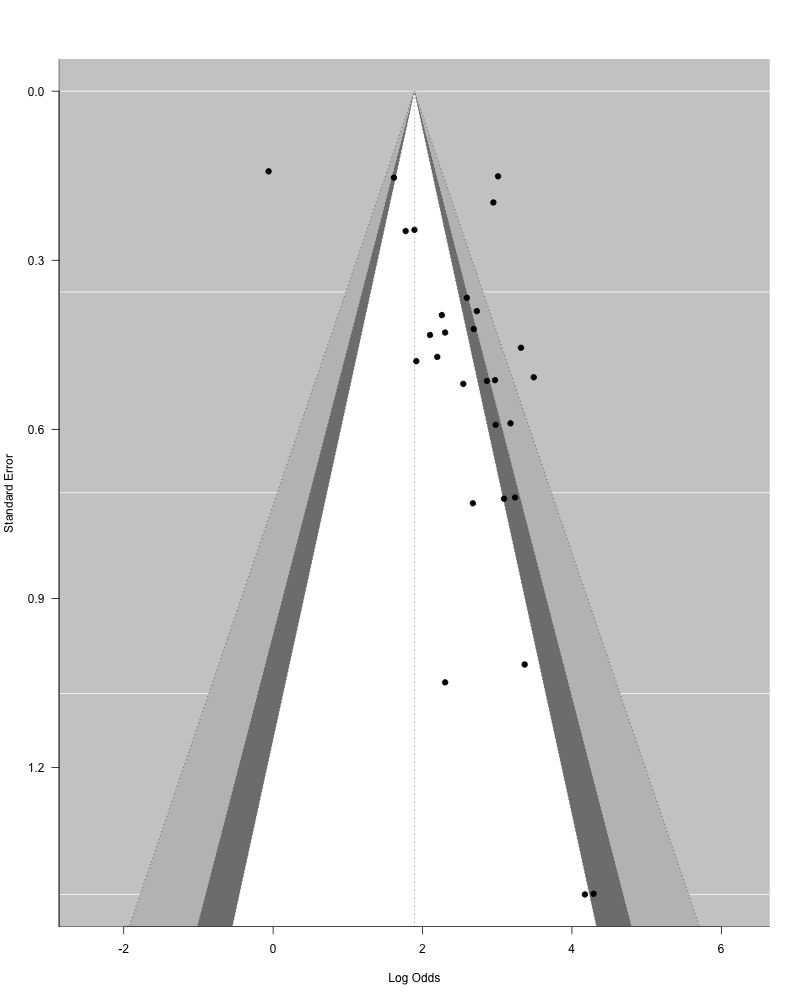
*

*S2.12 Funnel plot for specificity, SR + Mal*

*
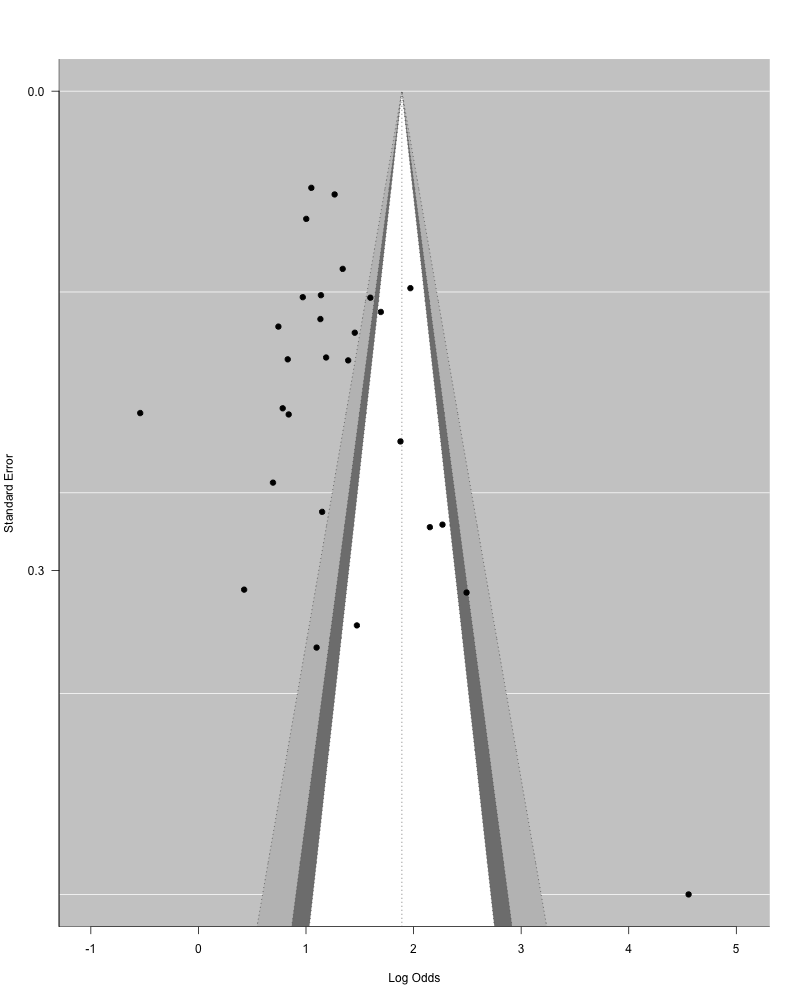
*

*S2.13 Funnel plot for sensitivity, SR + SA*

*
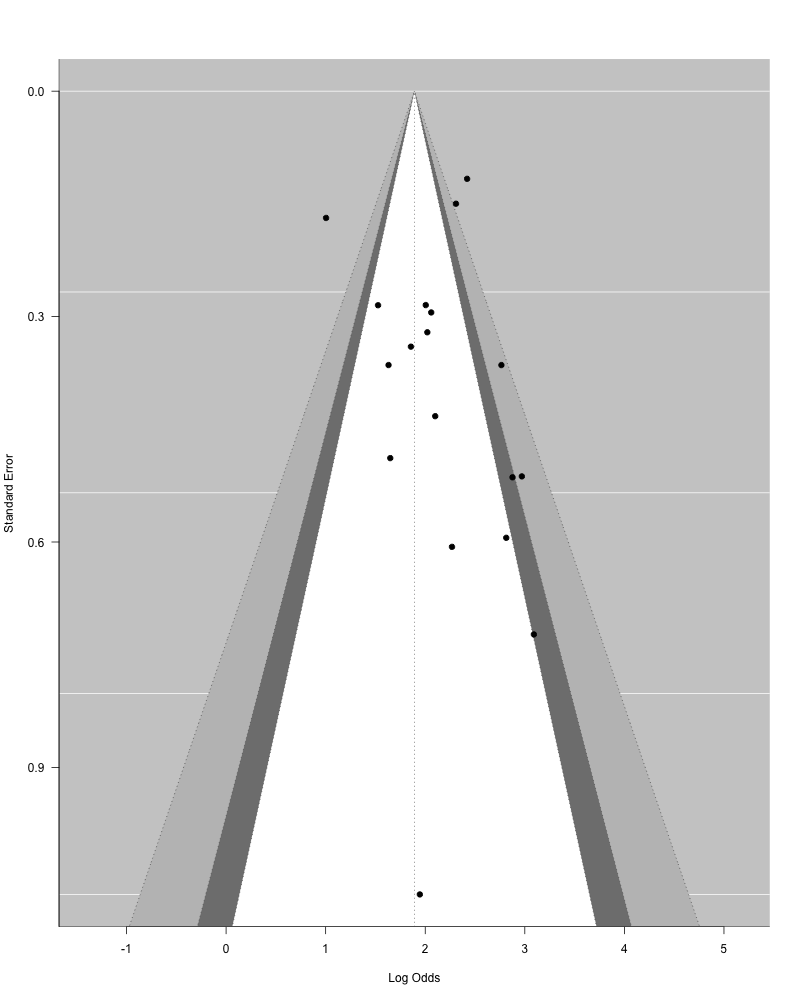
*

*S2.14 Funnel plot for specificity, SR + SA*

*
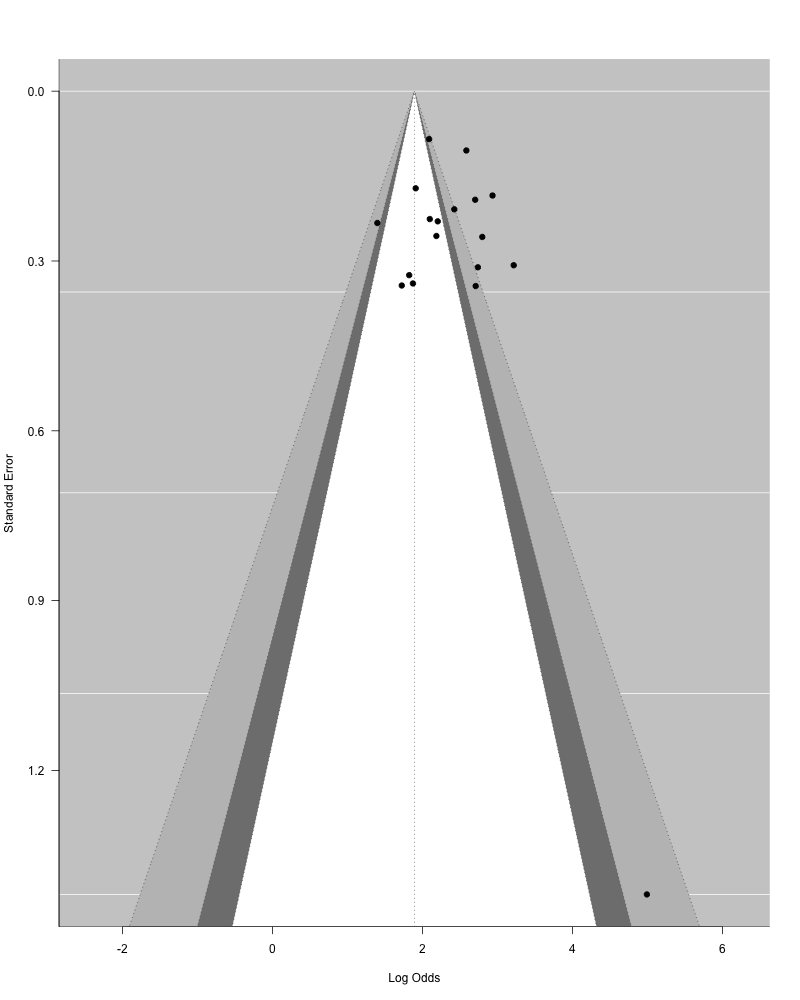
*

*S2.15 Funnel plot for sensitivity, ADNEX*

*
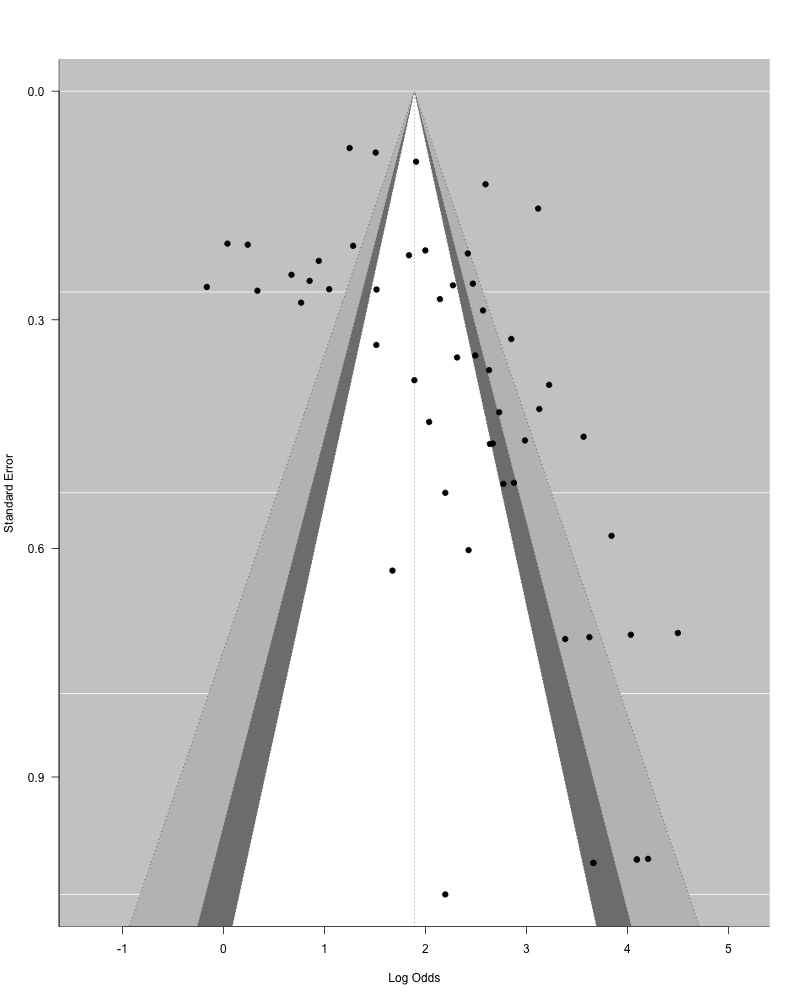
*

*S2.16 Funnel plot for specificity, ADNEX*

*
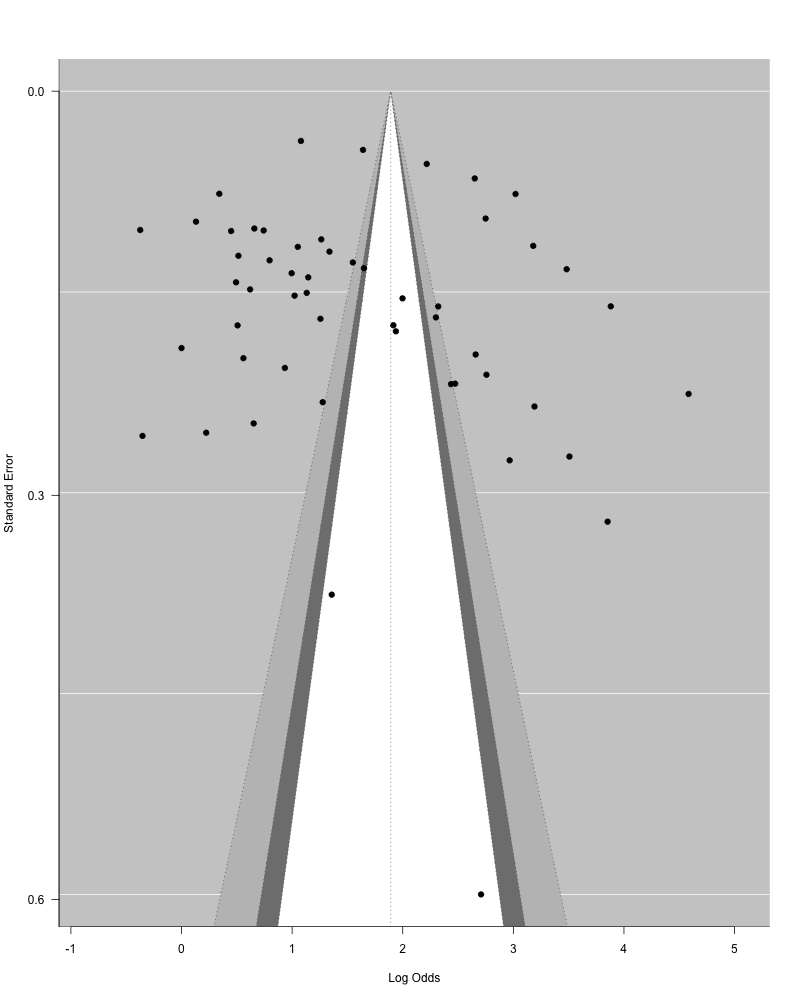
*

*S2.17 Funnel plot for sensitivity, SA*

*
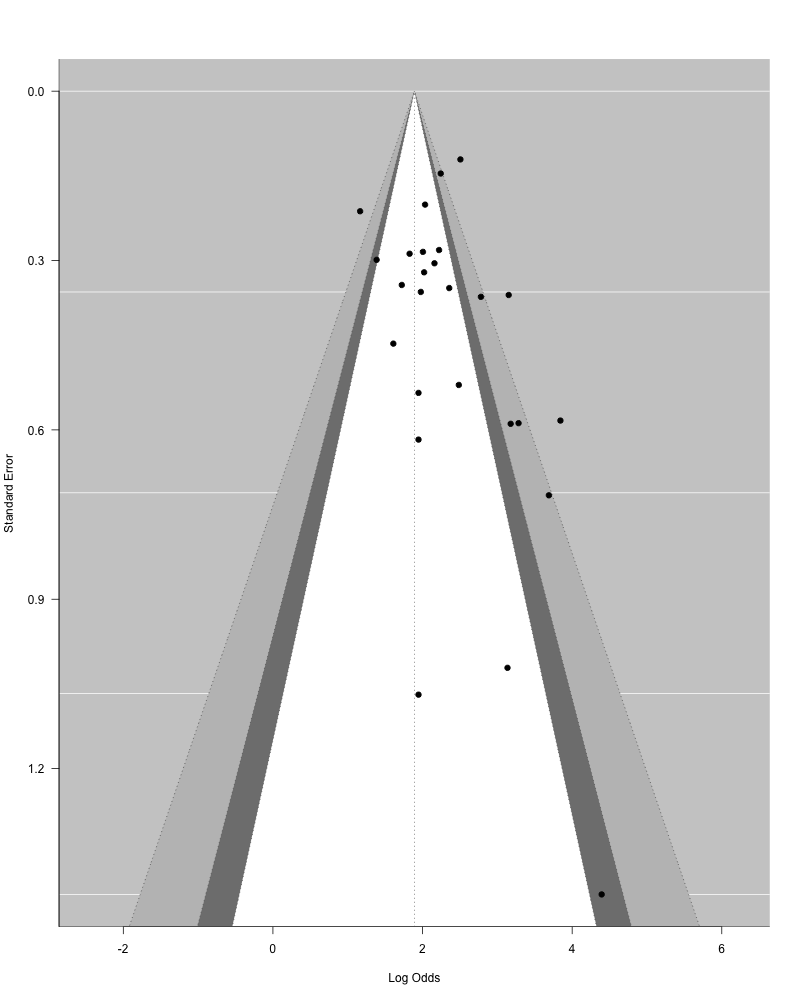
*

*S2.18 Funnel plot for specificity, SA*

*
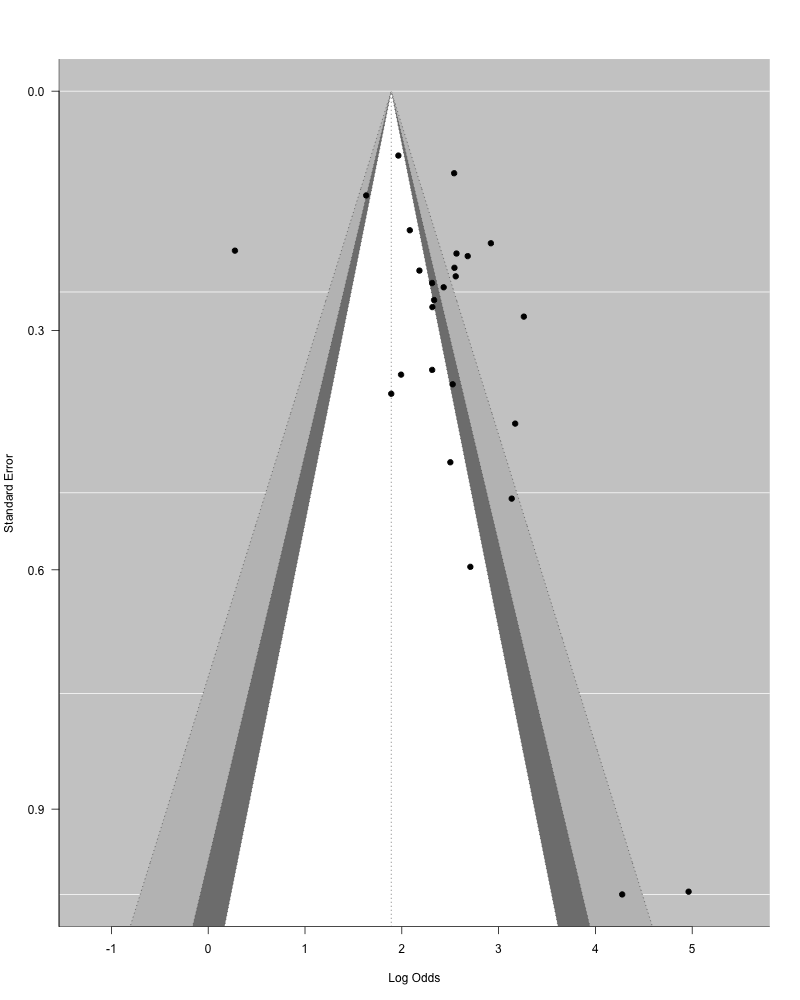
*

**Figure S3** Forest plots of all included models and cut-off values

*
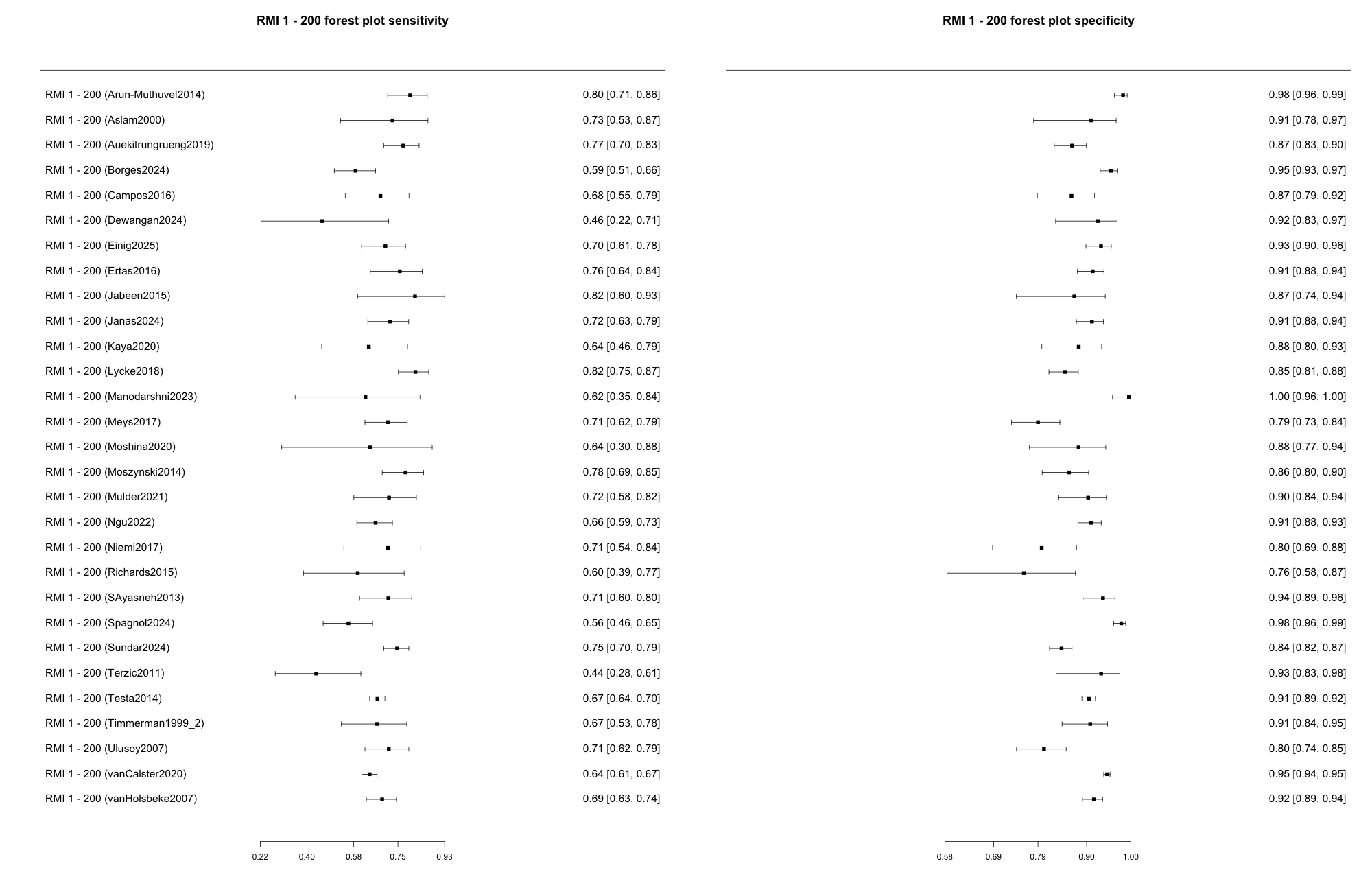
S3.1, Forest plot RMI 1 – 200*

*S3.2, Forest plot RMI 1 – 250*

*
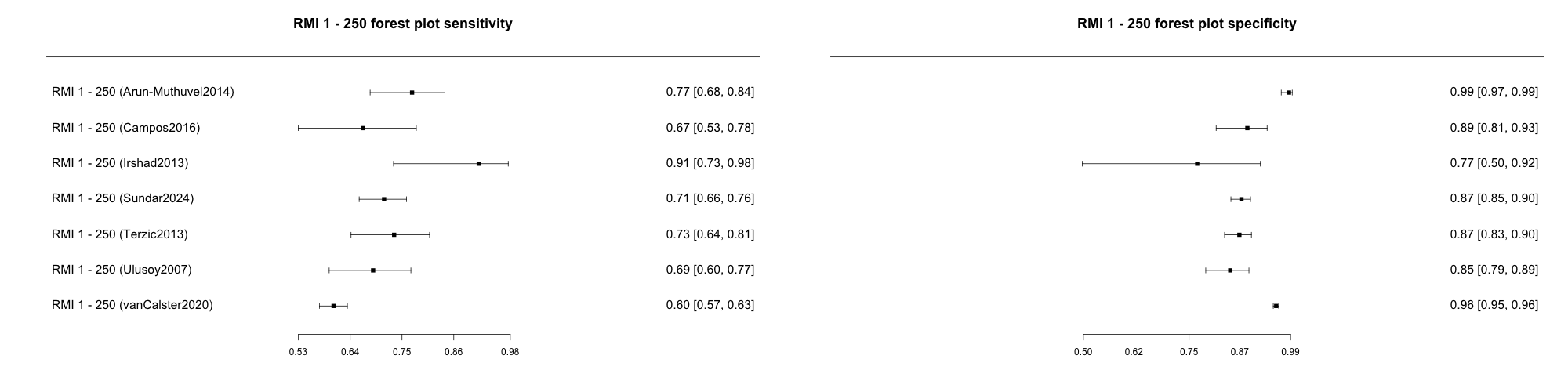
*

*S3.3, Forest plot RMI 2 – 200*

*
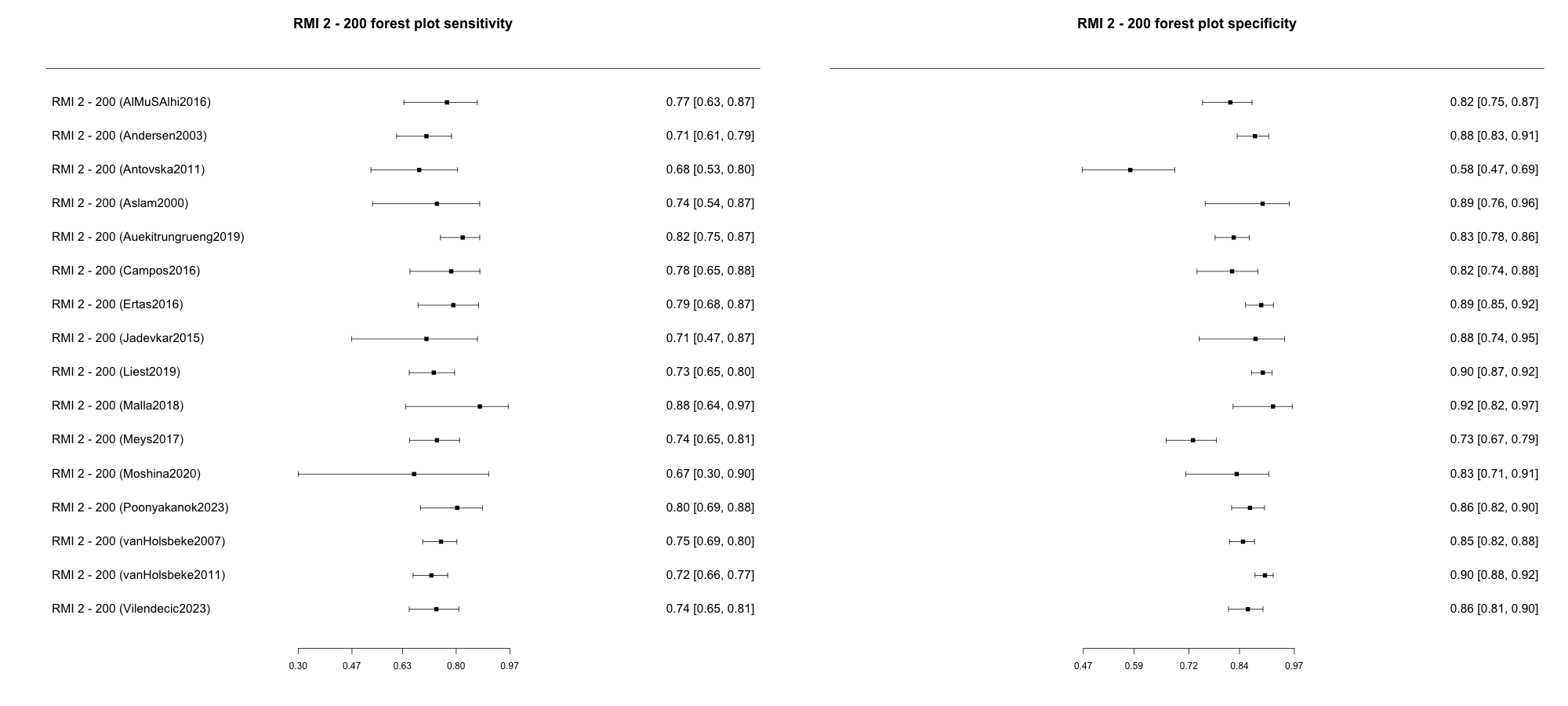
*

*S3.4, Forest plot RMI 2 – 250*

*
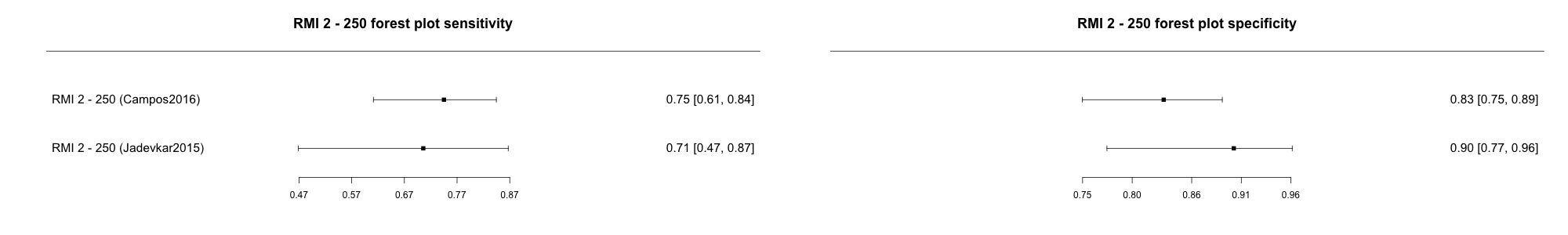
*

*S3.5, Forest plot RMI 3 – 200*

*
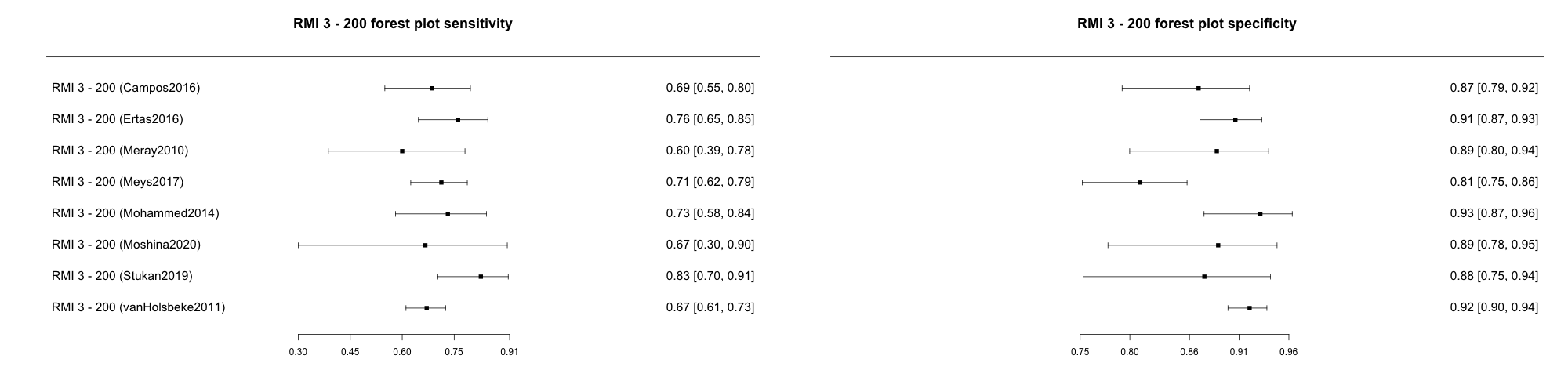
*

*S3.6, Forest plot RMI 3 – 250*

*
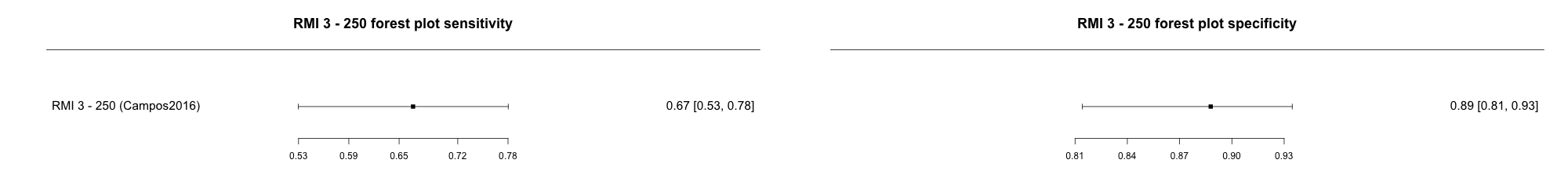
*

*S3.7, Forest plot LR2 – 10%*

*
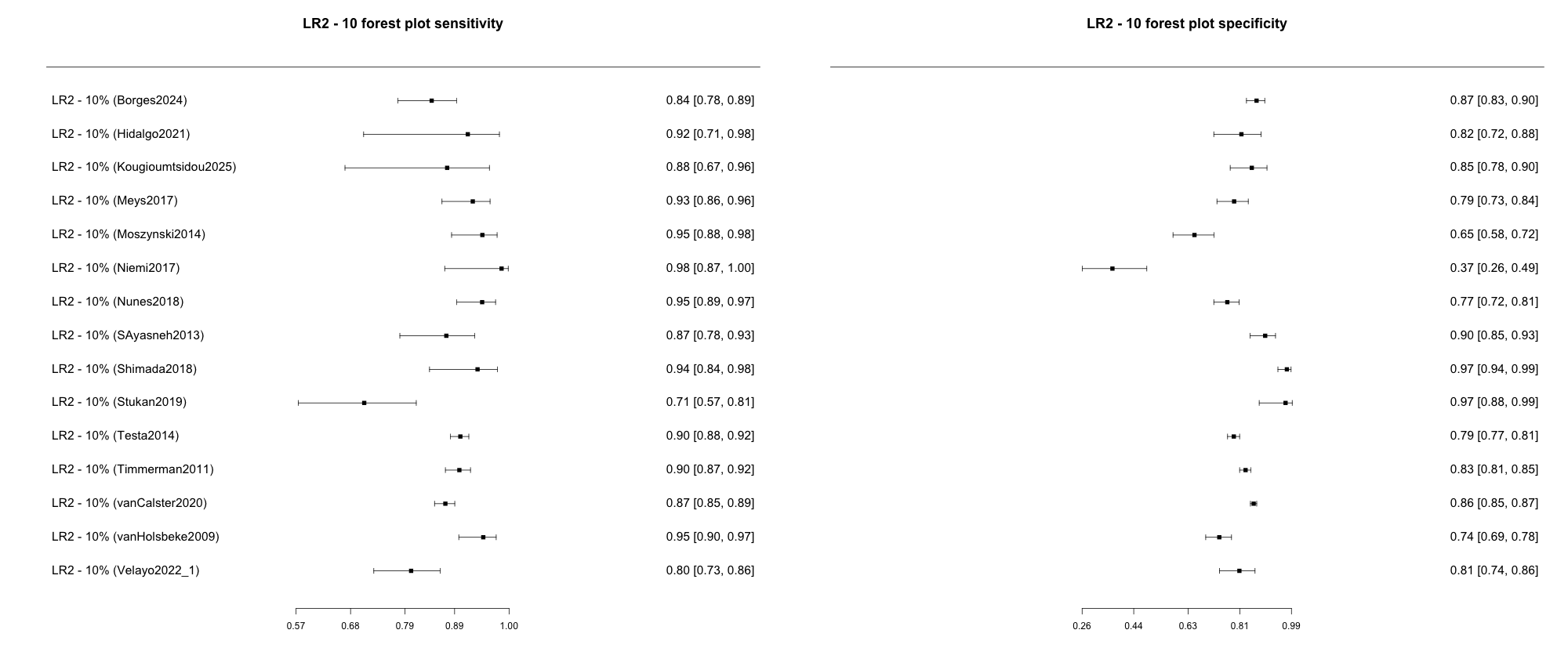
*

*S3.8, Forest plot Simple Rules + Malignant*

*
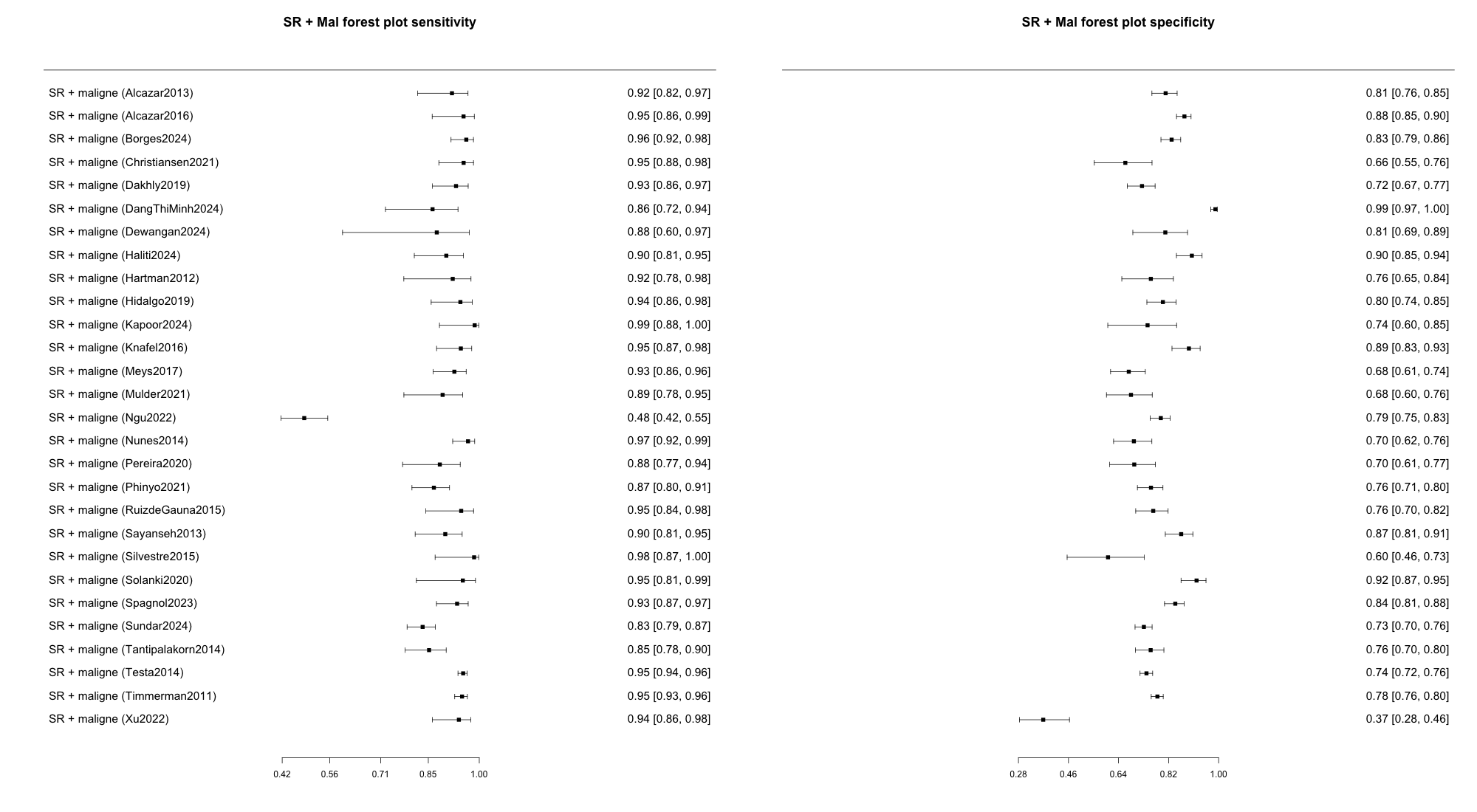
*

*S3.9, Forest plot Simple Rules + Subjective Assessment*

*
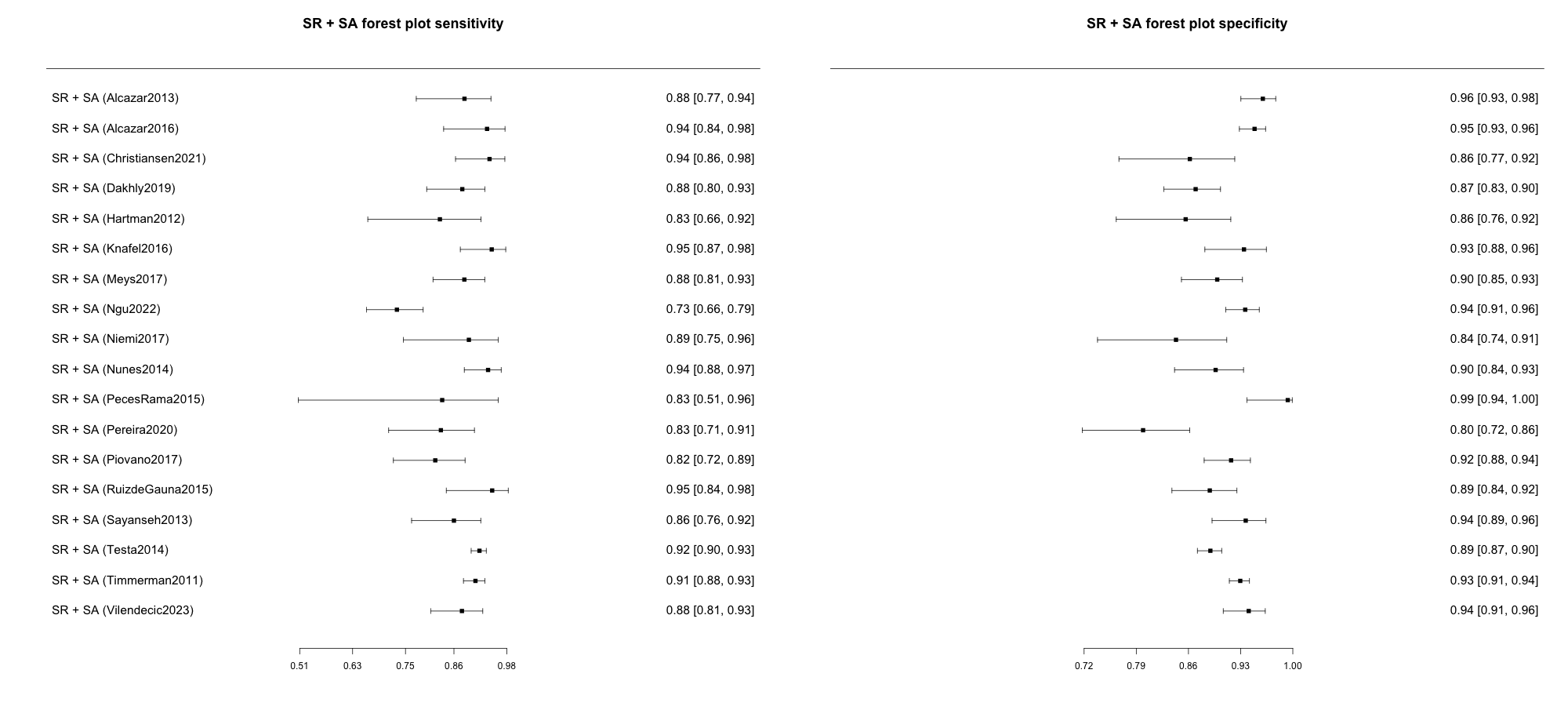
*

*S3.10, Forest plot ADNEX – 5%*

*
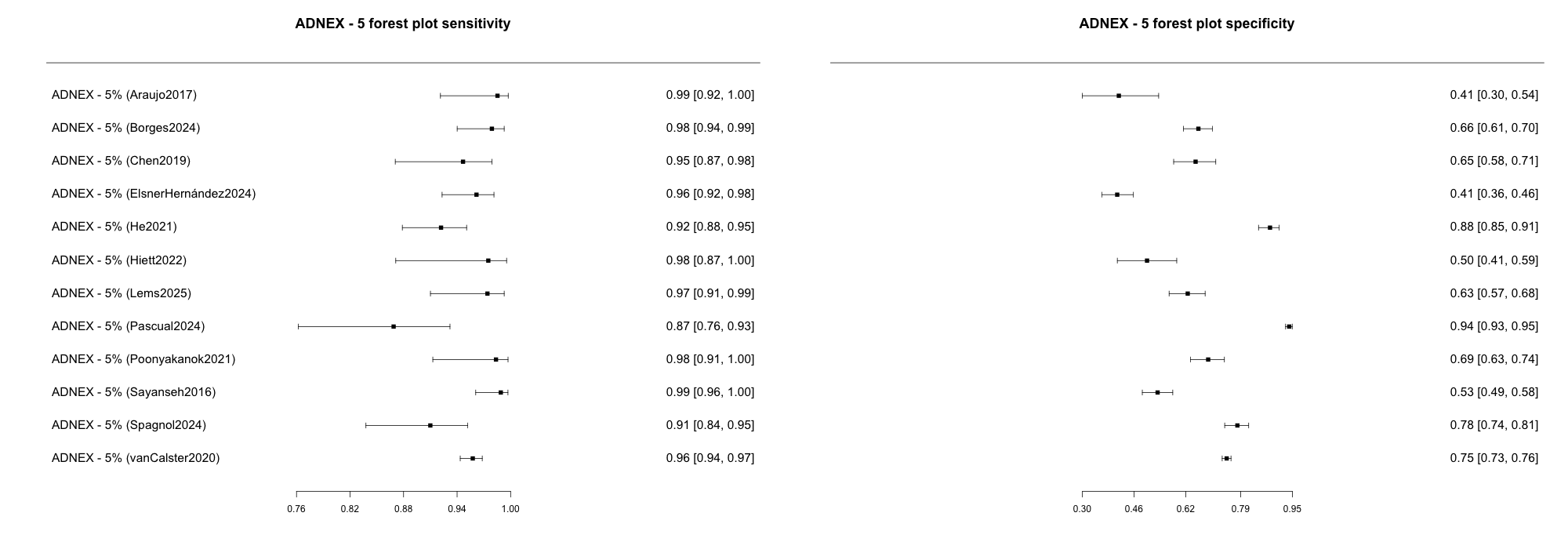
*

*S3.11, Forest plot ADNEX – 10%*

*
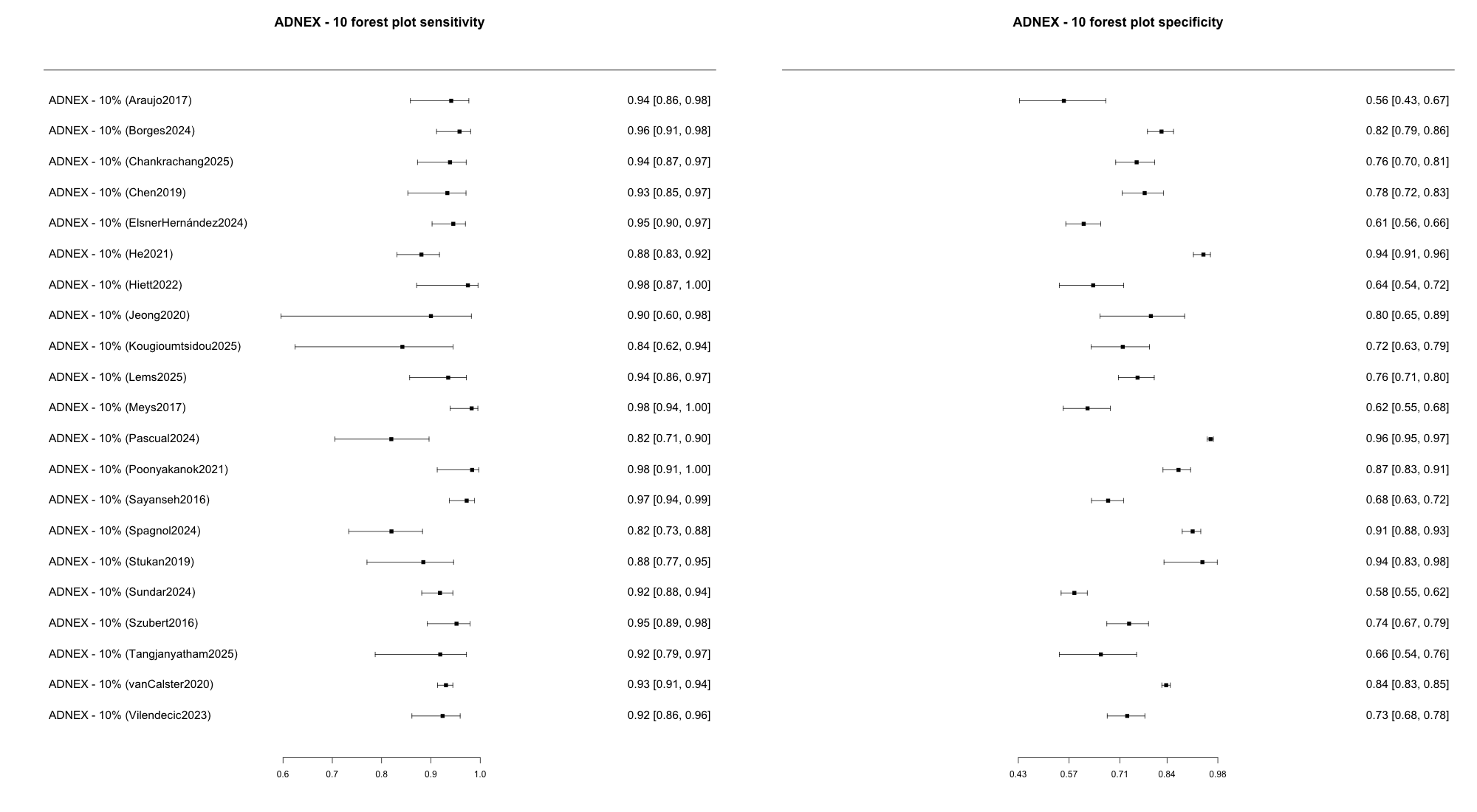
*

*S3.12, Forest plot ADNEX – 20%*

*
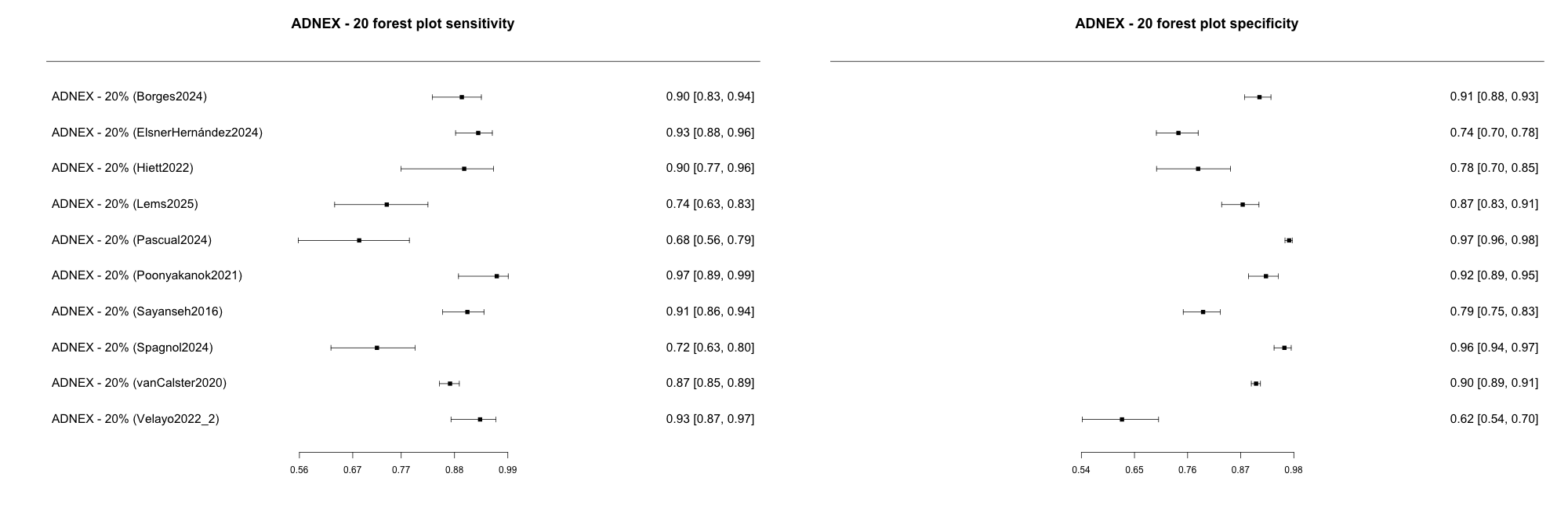
*

*S3.13 Forest plot ADNEX – 30%*

*
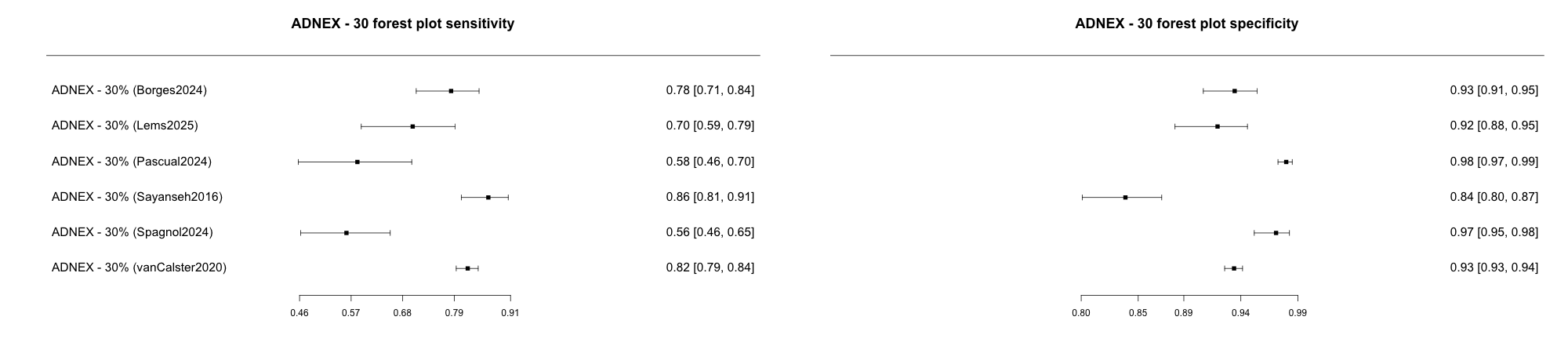
*

*S3.14, Forest plot ADNEX – 40%*

*
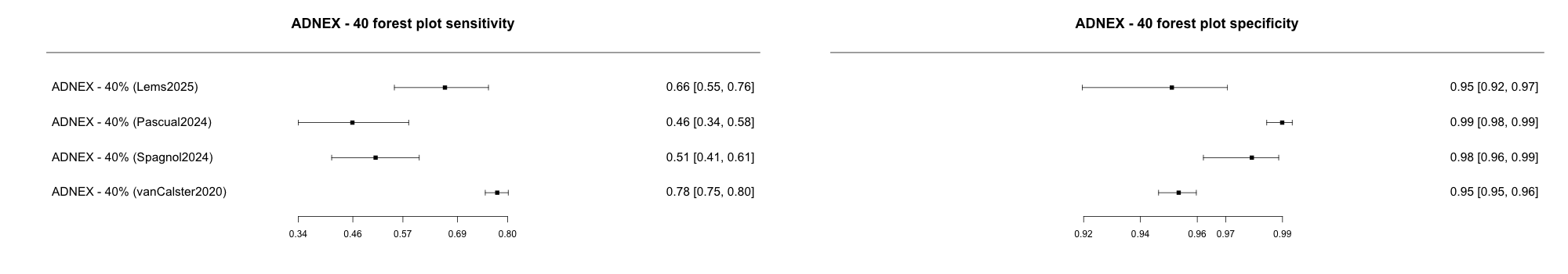
*

*S3.15, Forest plot Subjective Assessment*

*
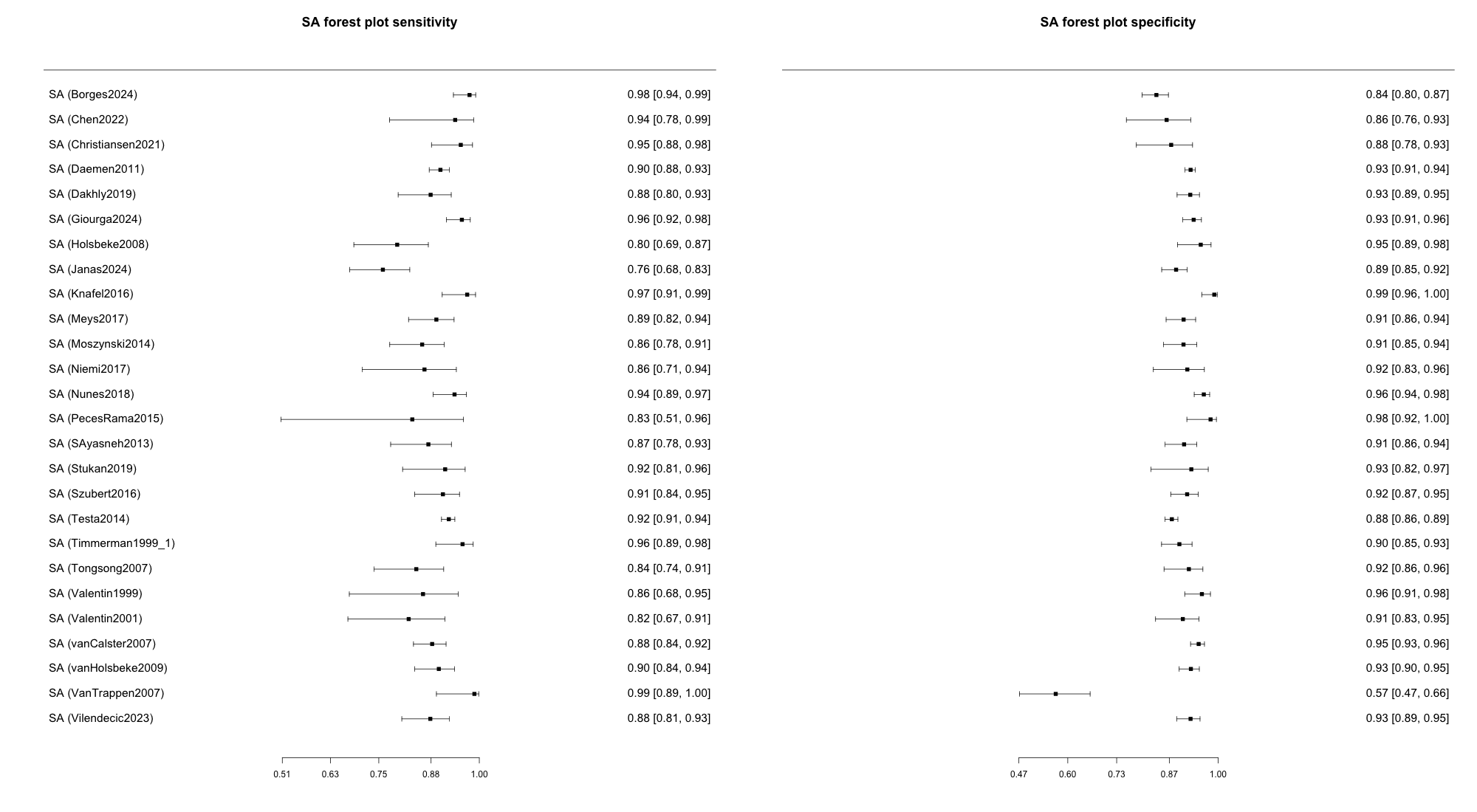
*

**Figure S4** Estimated pooled predictive values of included models.

*S4.1, Estimated pooled predictive values of RMI 1 – 200*

*
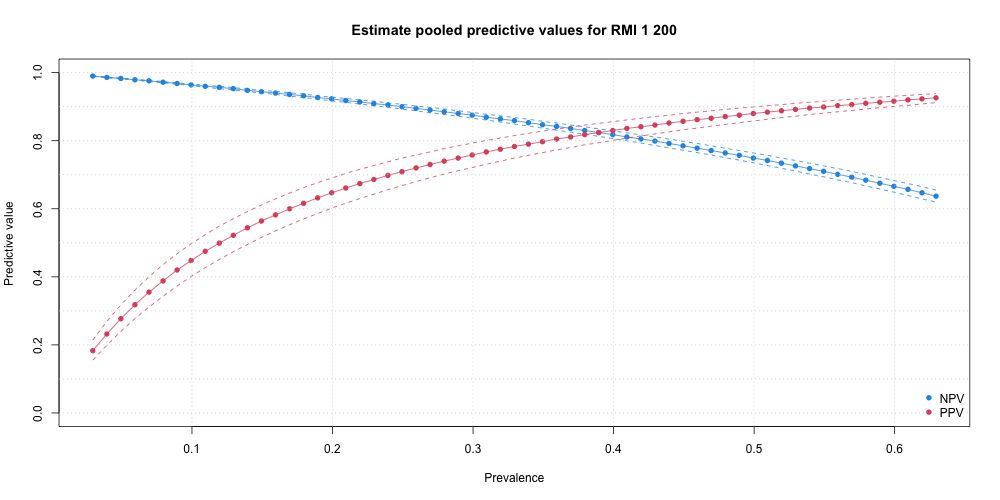
*

*S4.2, Estimated pooled predictive values of RMI 1 – 250*

*
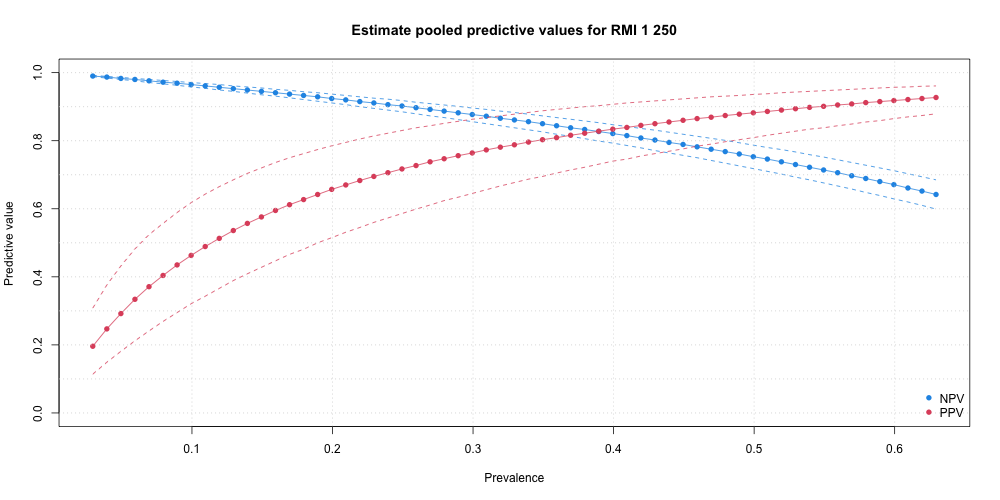
*

*S4.3, Estimated pooled predictive values of RMI 2 – 200*


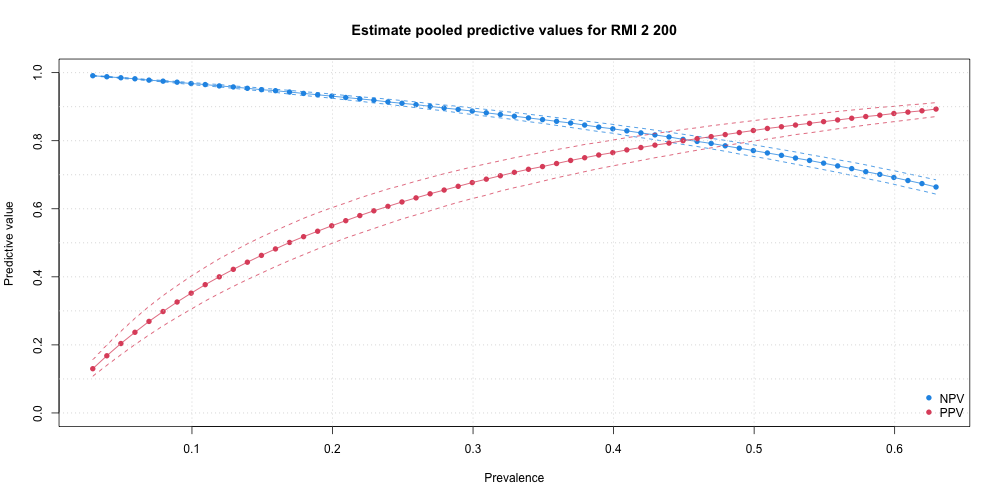


*S4.4, Estimated pooled predictive values of RMI 2 – 250*

*
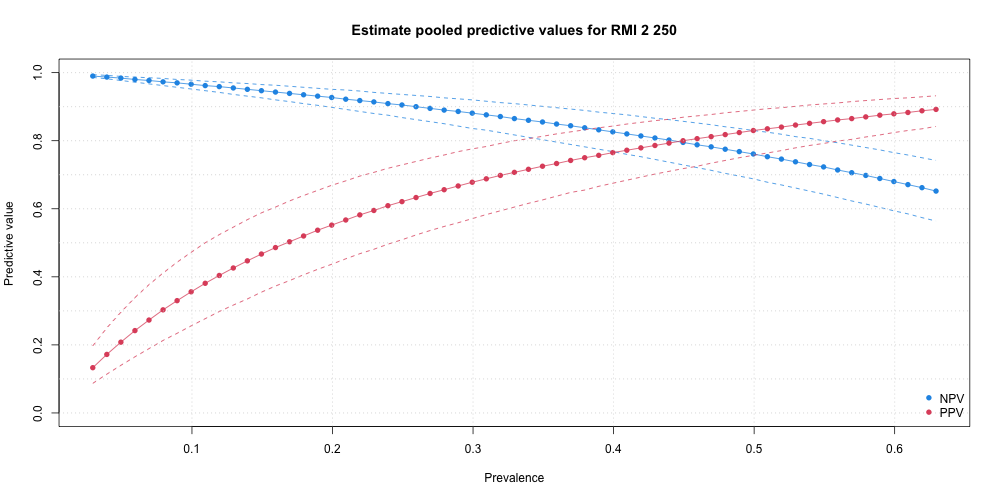
*

*S4.5, Estimated pooled predictive values of RMI 3 – 200*

*
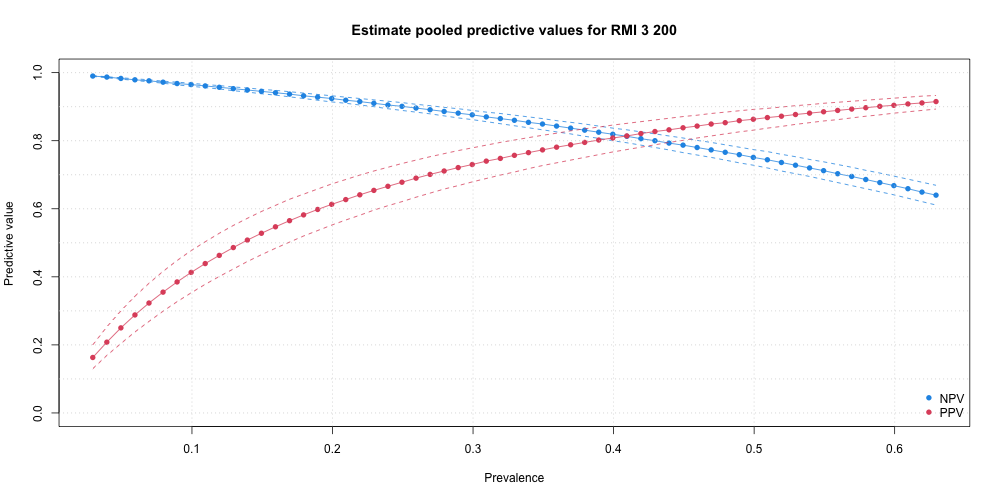
*

*S4.6, Estimated pooled predictive values of RMI 3 – 250*

*
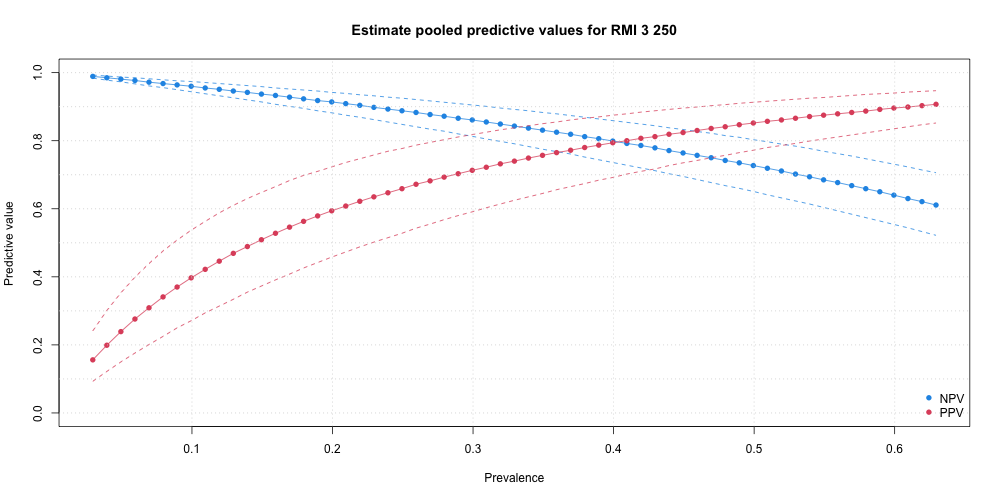
*

*S4.7, Estimated pooled predictive values of LR2 – 10%*

*
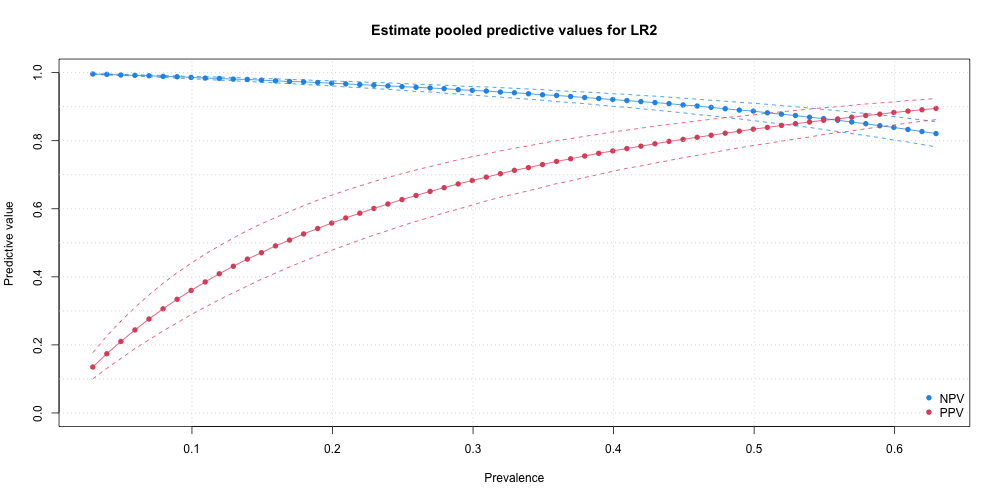
*

*S4.8, Estimated pooled predictive values of Simple Rules + Malignant*

*
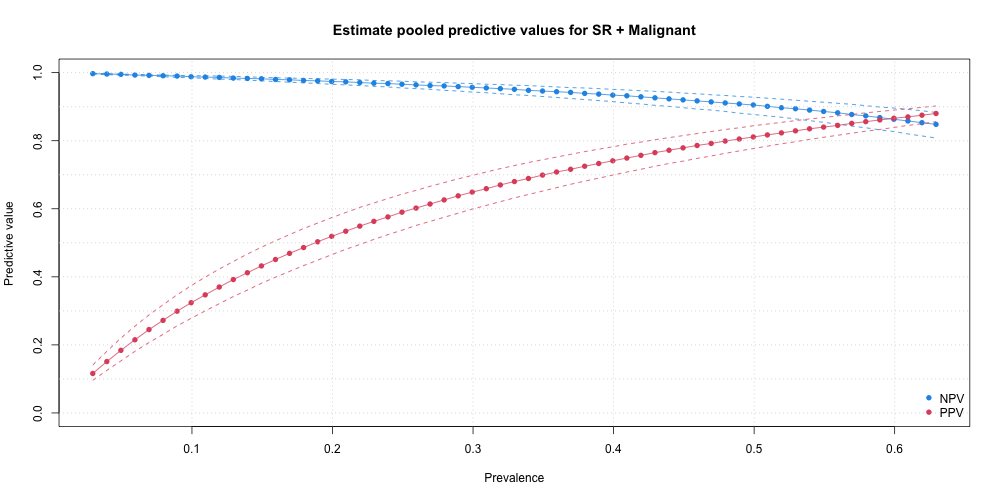
*

*S4.9, Estimated pooled predictive values of Simple Rules + Subjective Assessment*

*
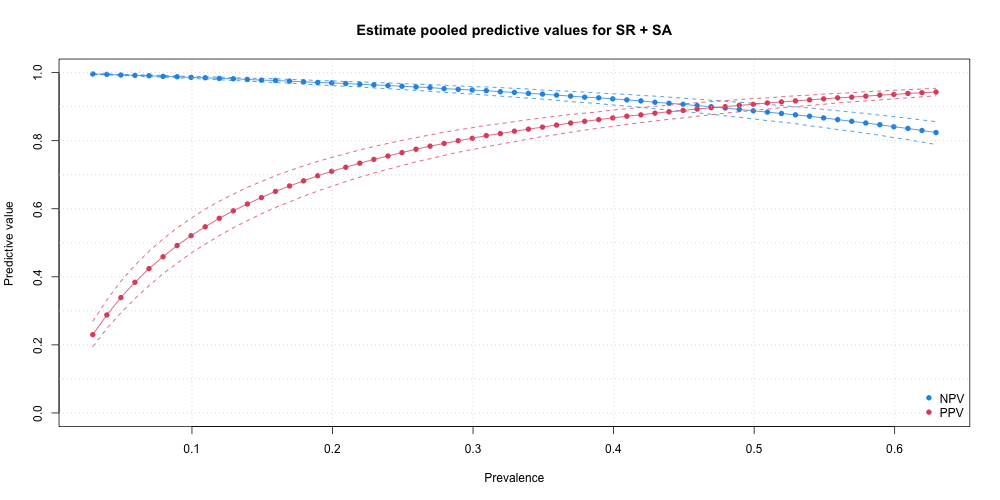
*

*S4.10, Estimated pooled predictive values of ADNEX – 5%*

*
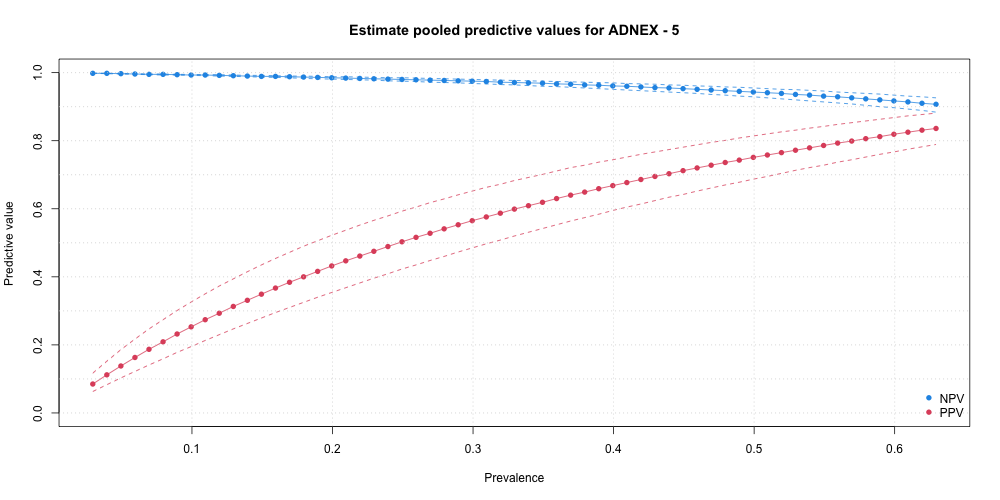
*

*S4.11, Estimated pooled predictive values of ADNEX – 10%*

*
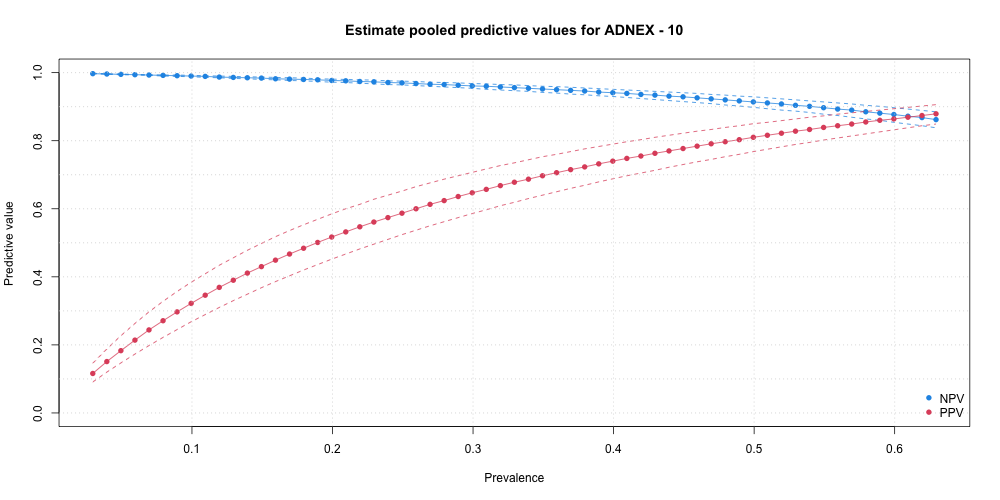
*

*S4.12, Estimated pooled predictive values of ADNEX – 20%*

*
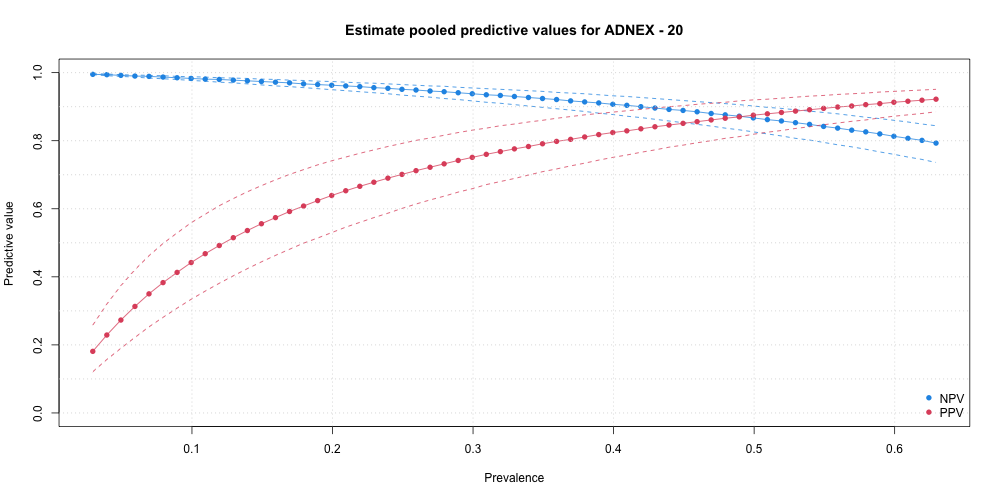
*

*S4.13, Estimated pooled predictive values of ADNEX – 30%*

*
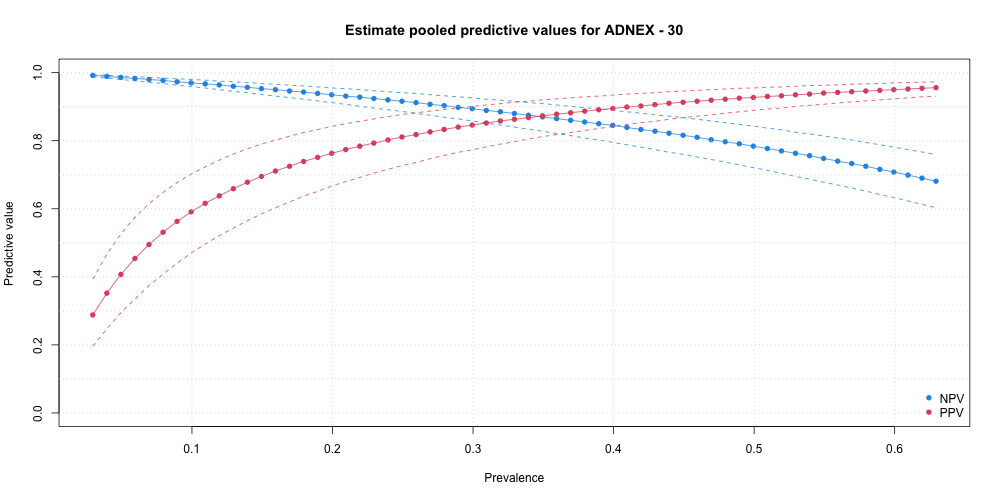
*

*S4.14, Estimated pooled predictive values of ADNEX – 40%*

*
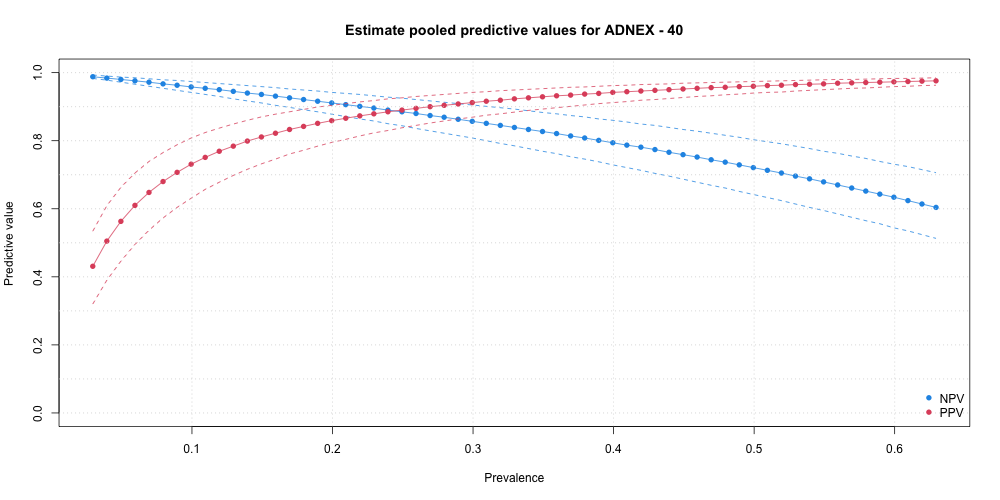
*

*S4.15, Estimated pooled predictive values of Subjective Assessment*

*
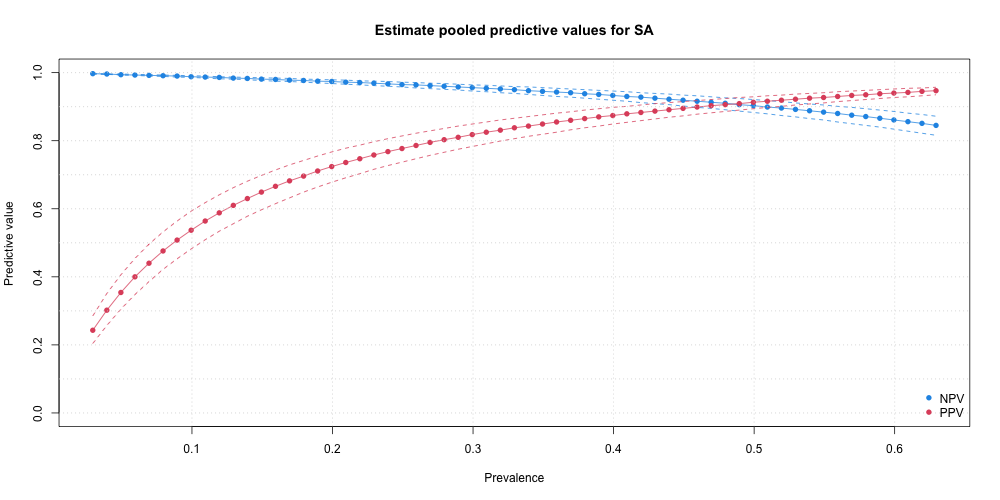
*

**Figure S5** Forest plots with individual study results for subgroup of premenopausal women

*S5.1, Forest plot RMI 1 – 200, premenopausal women*

*
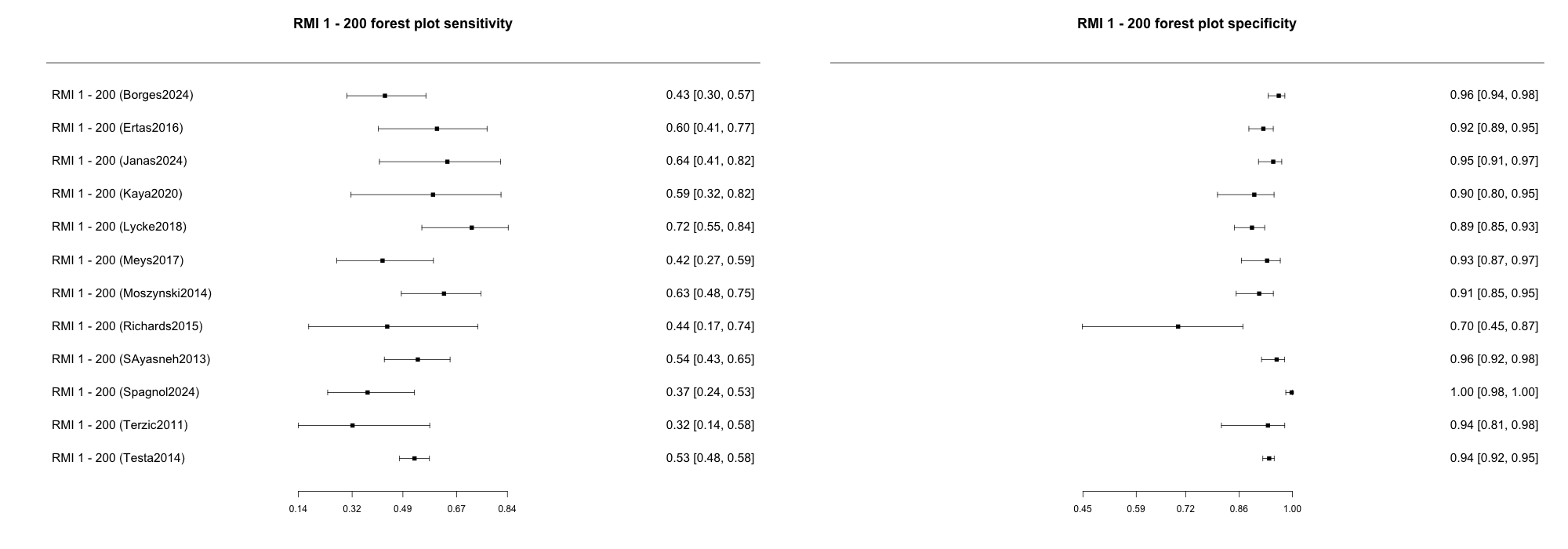
*

*S5.2, Forest plot RMI 1 – 250, premenopausal women*

*
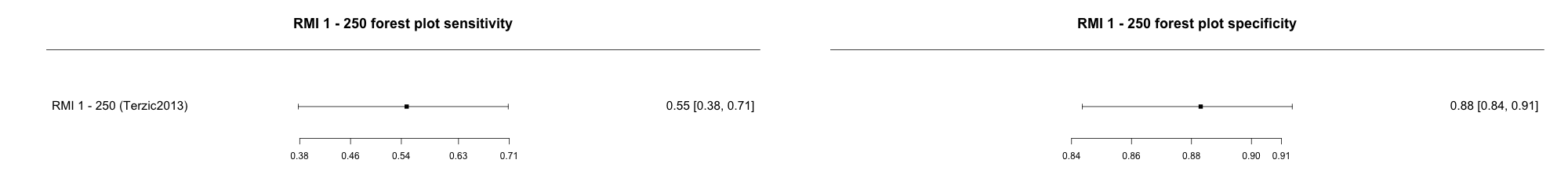
*

*S5.3 Forest plot RMI 2 – 200, premenopausal women*


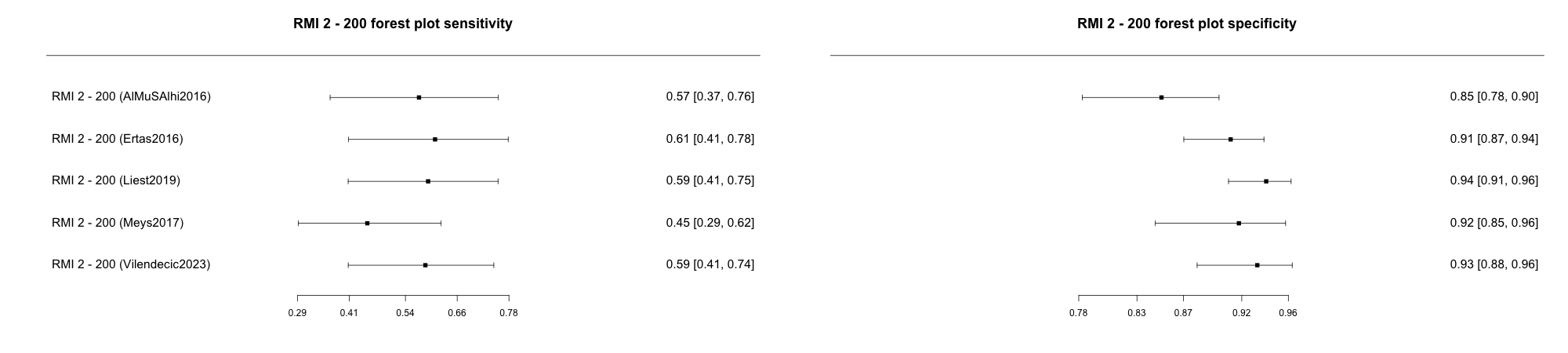


*S5.4, Forest plot RMI 3 – 200, premenopausal women*

*
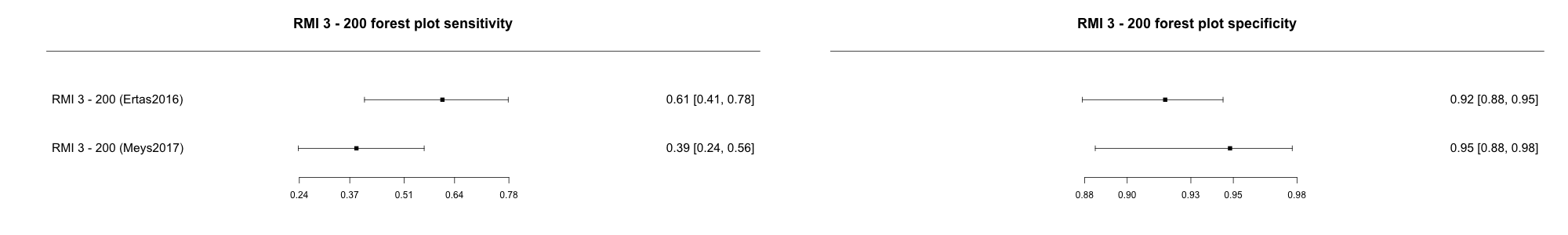
*

*S5.5 Forest plot LR2 – 10%, premenopausal women*

*
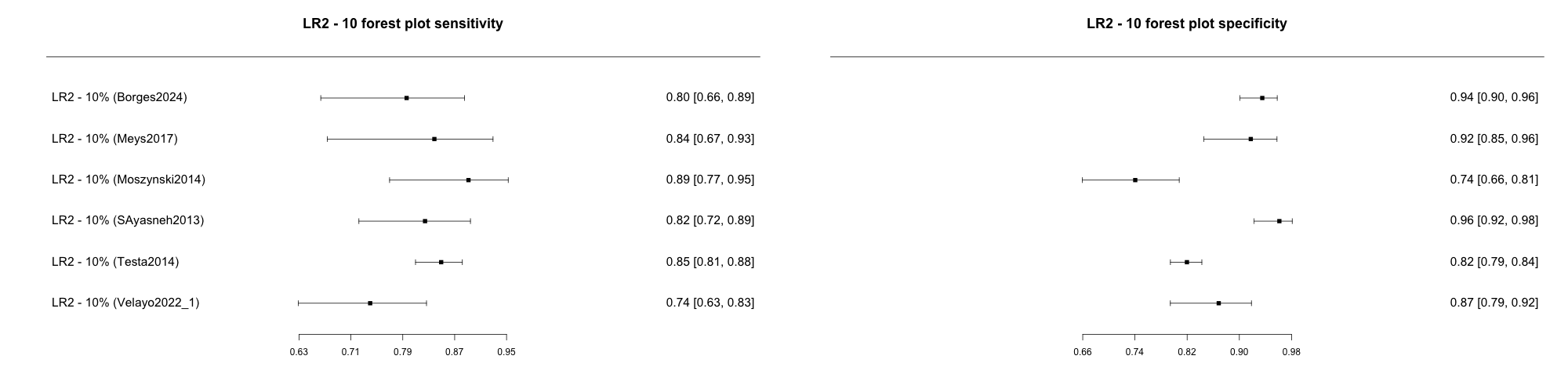
*

*S5.6, Forest plot Simple Rules + Malignant, premenopausal women*

*
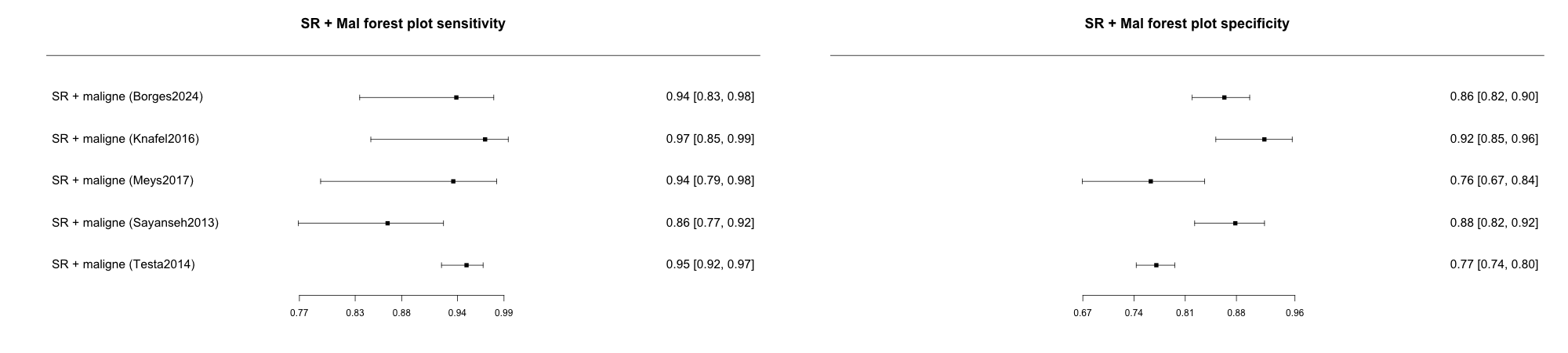
*

*S5.7, Forest plot Simple Rules + Subjective Assessment, premenopausal women*


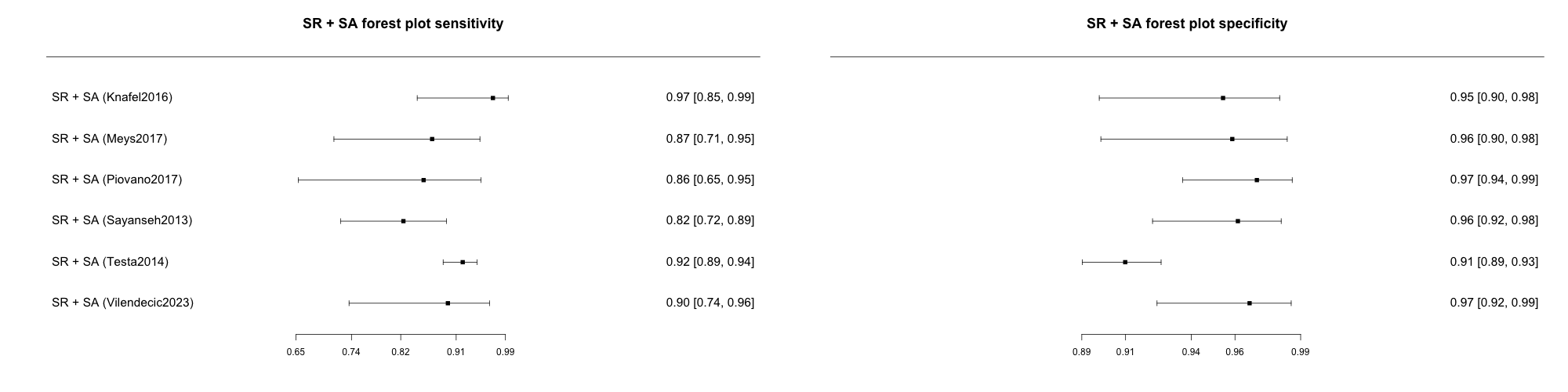


*S5.8, Forest plot ADNEX – 5%, premenopausal women*


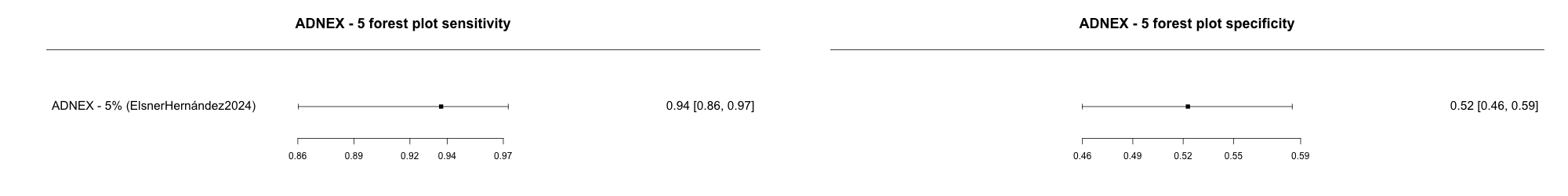


*S5.9, Forest plot ADNEX – 10%, premenopausal women*


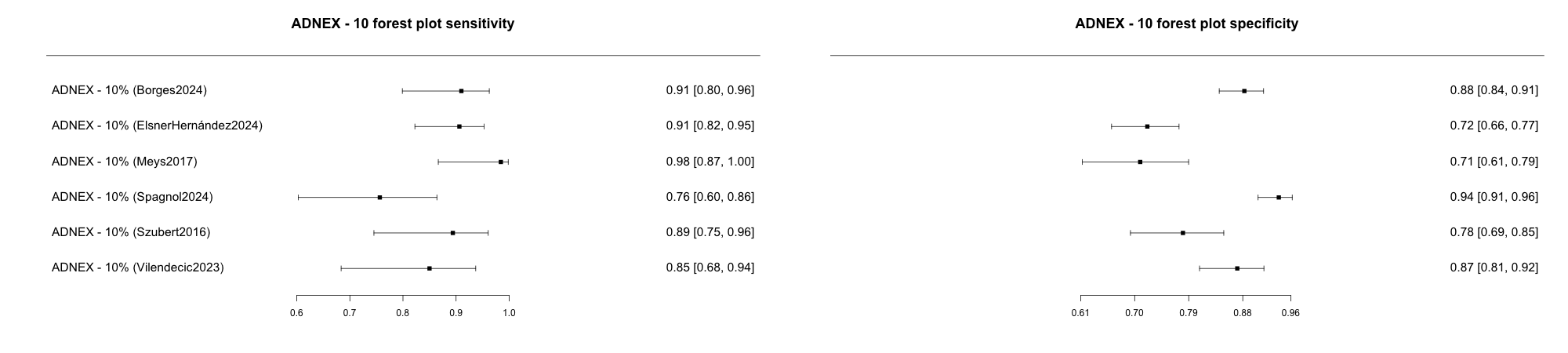


*S5.10, Forest plot ADNEX – 20%, premenopausal women*


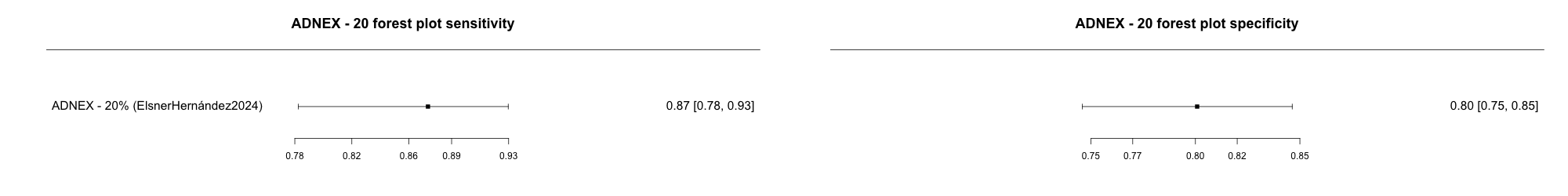


*S5.11, Forest plot Subjective Assessment, premenopausal women*


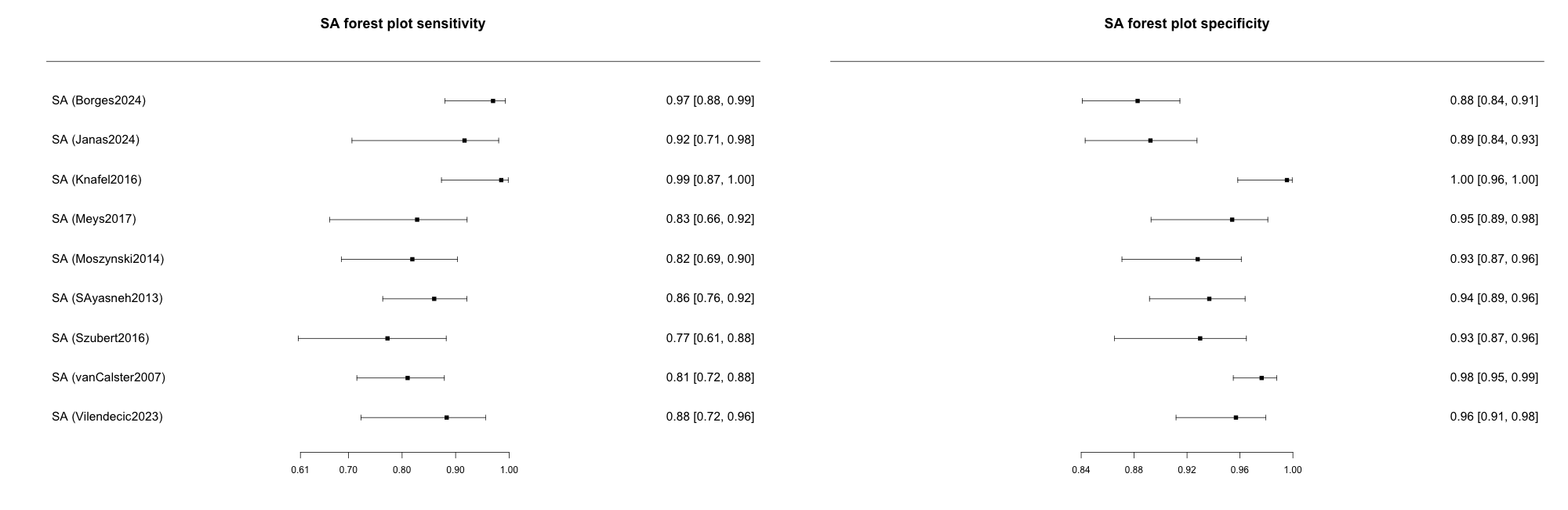


**Figure S6** Forest plots with individual study results for subgroup of postmenopausal women

*S6.1, Forest plot RMI 1 – 200, postmenopausal women*

*
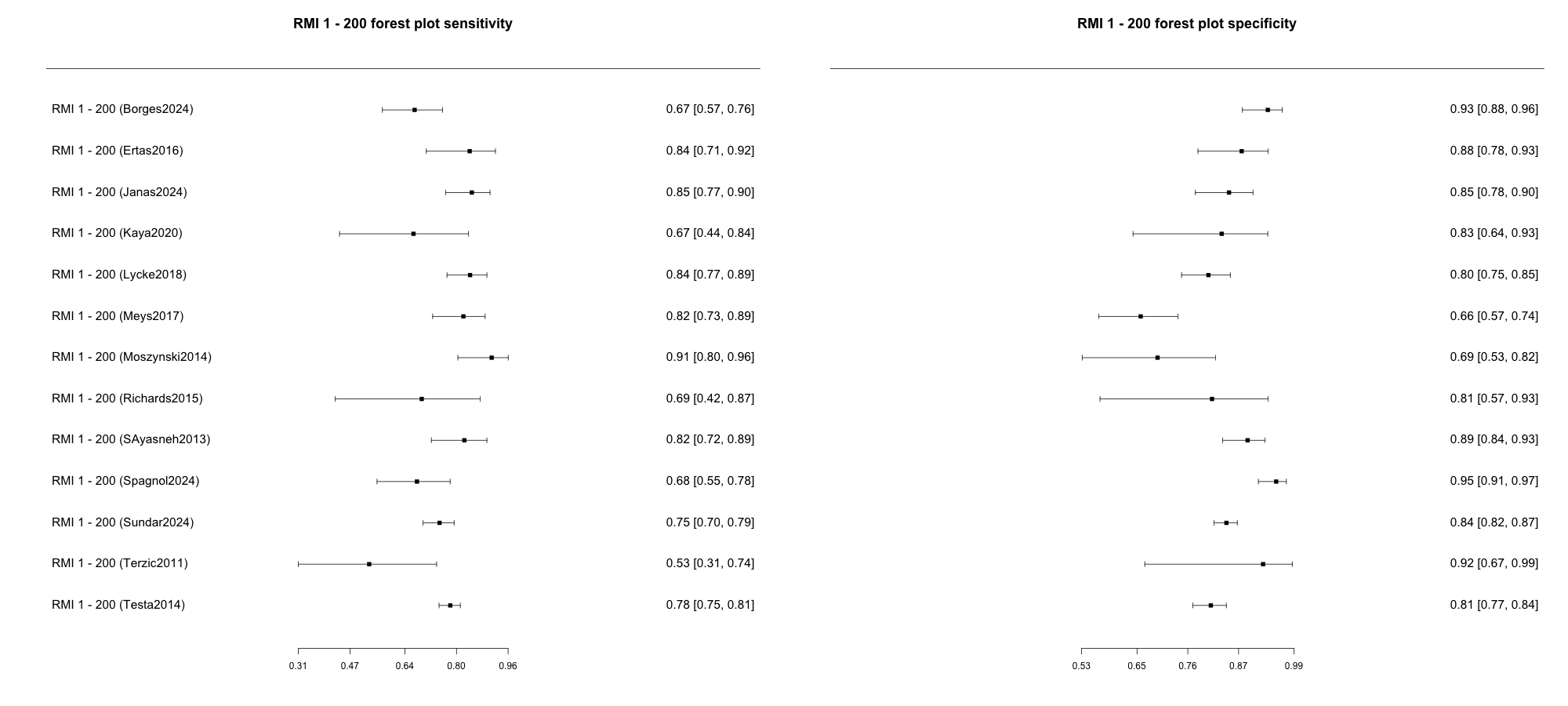
*

*S6.2, Forest plot RMI 1 – 250, postmenopausal women*

*
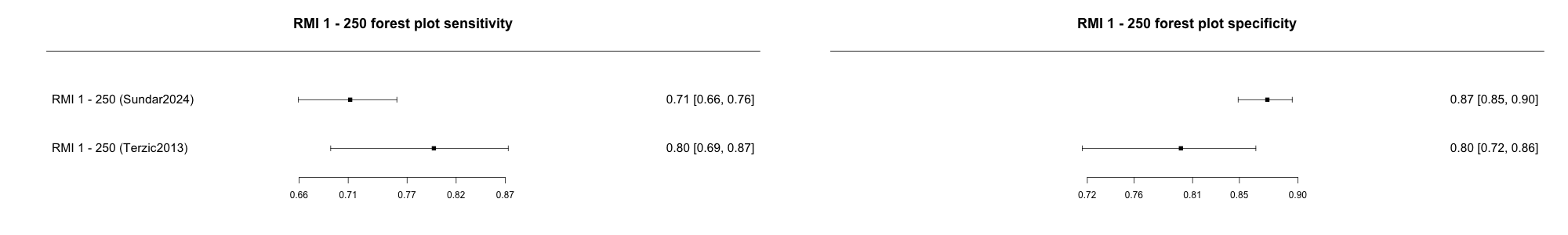
*

*S6.3 Forest plot RMI 2 – 200, postmenopausal women*


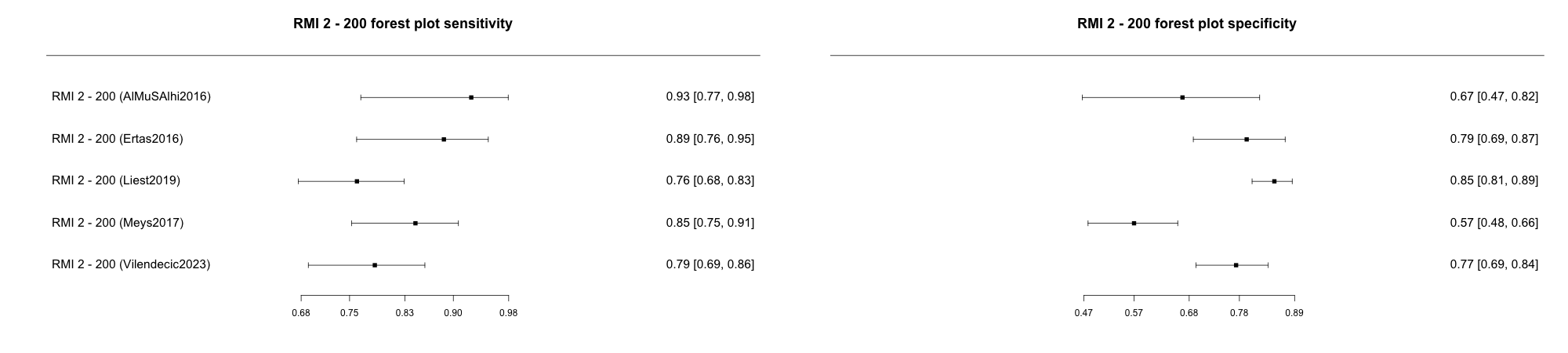


*S6.4, Forest plot RMI 3 – 200, postmenopausal women*

*
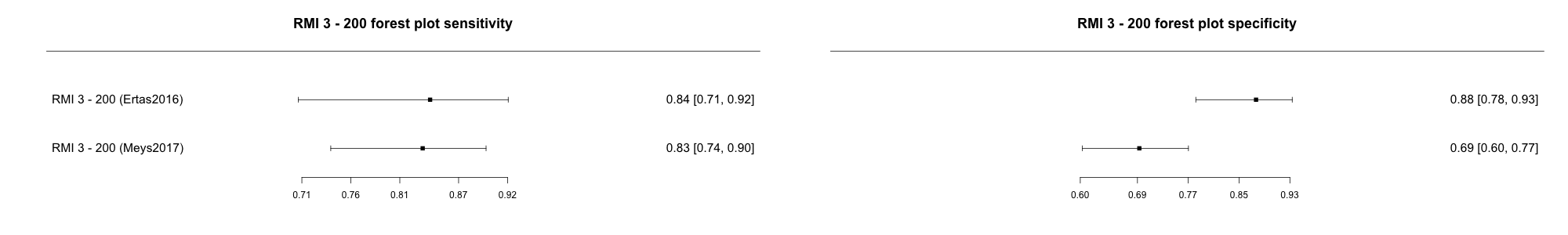
*

*S6.5 Forest plot LR2 – 10%, postmenopausal women*

*
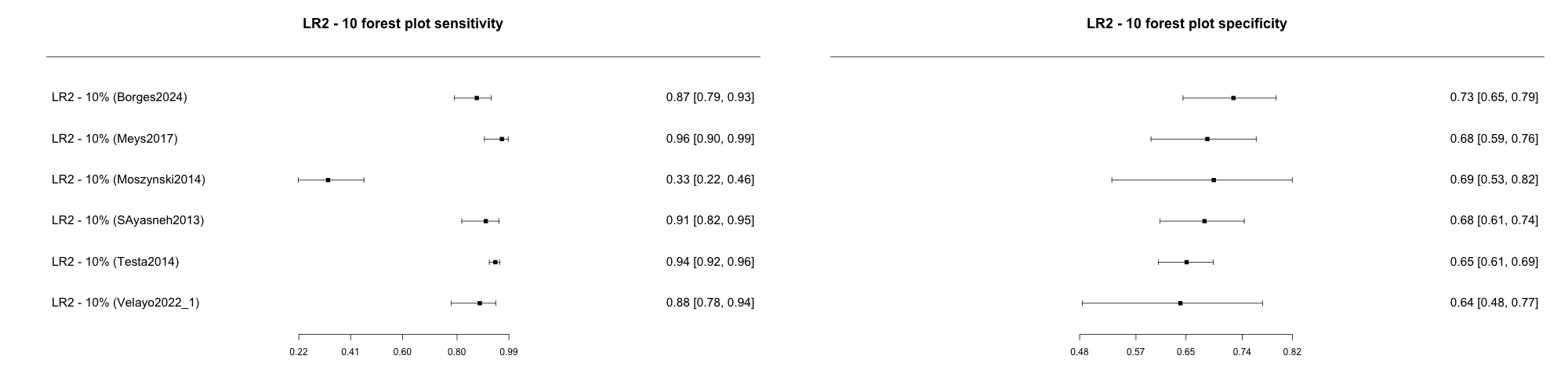
*

*S6.6, Forest plot Simple Rules + Malignant, postmenopausal women*

*
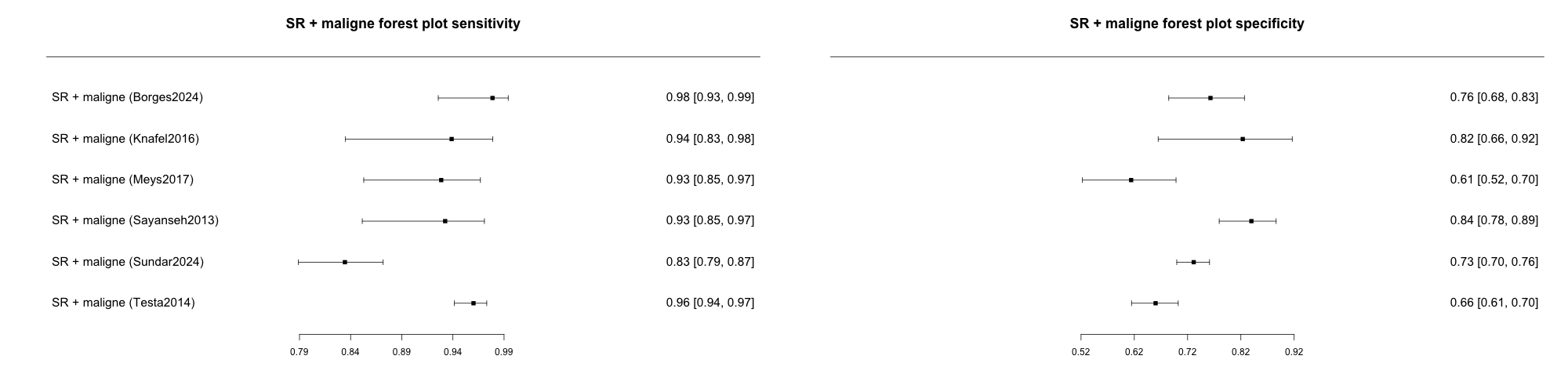
*

*S6.7, Forest plot Simple Rules + Subjective Assessment, postmenopausal women*


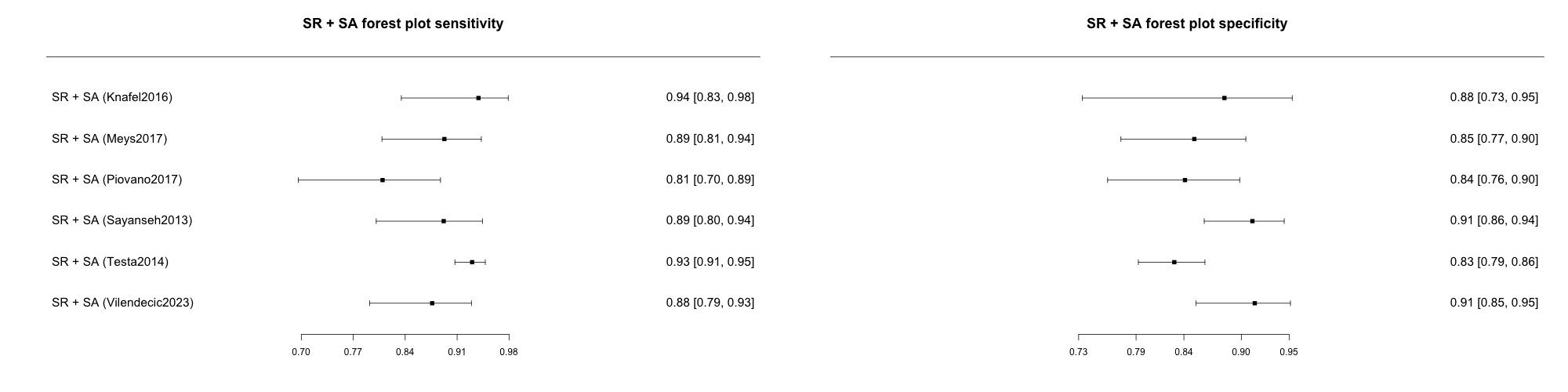


*S6.8, Forest plot ADNEX – 5%, postmenopausal women*


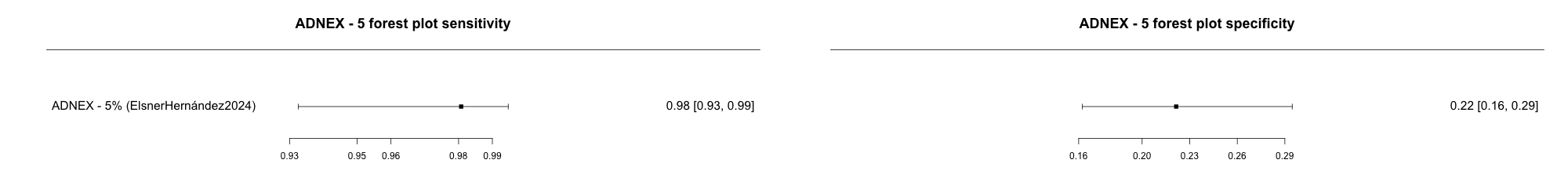


*S6.9, Forest plot ADNEX – 10%, postmenopausal women*


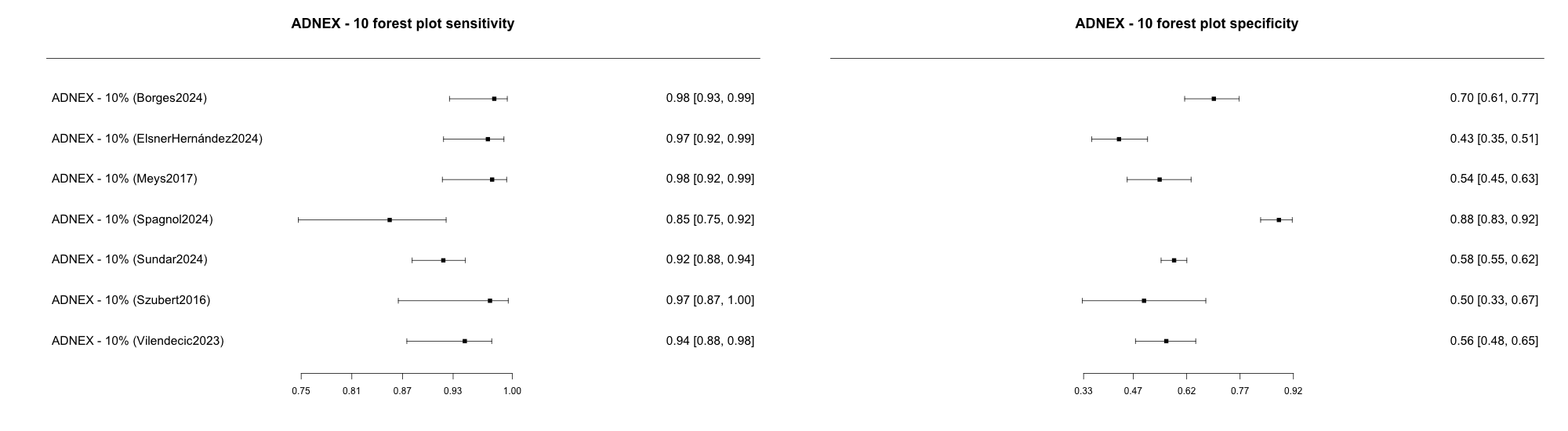


*S6.10, Forest plot ADNEX – 20%, postmenopausal women*


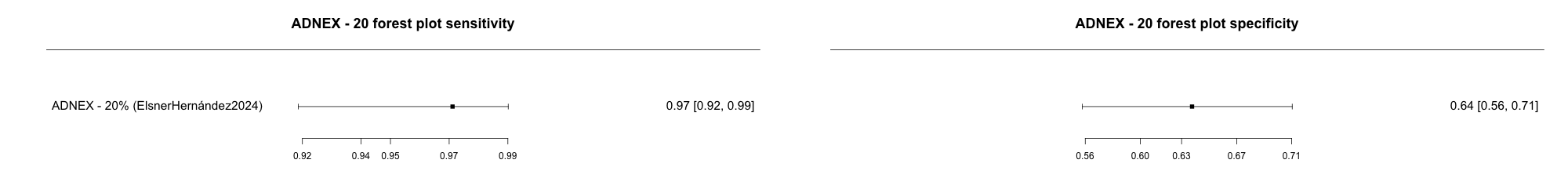


*S6.11, Forest plot Subjective Assessment, postmenopausal women*


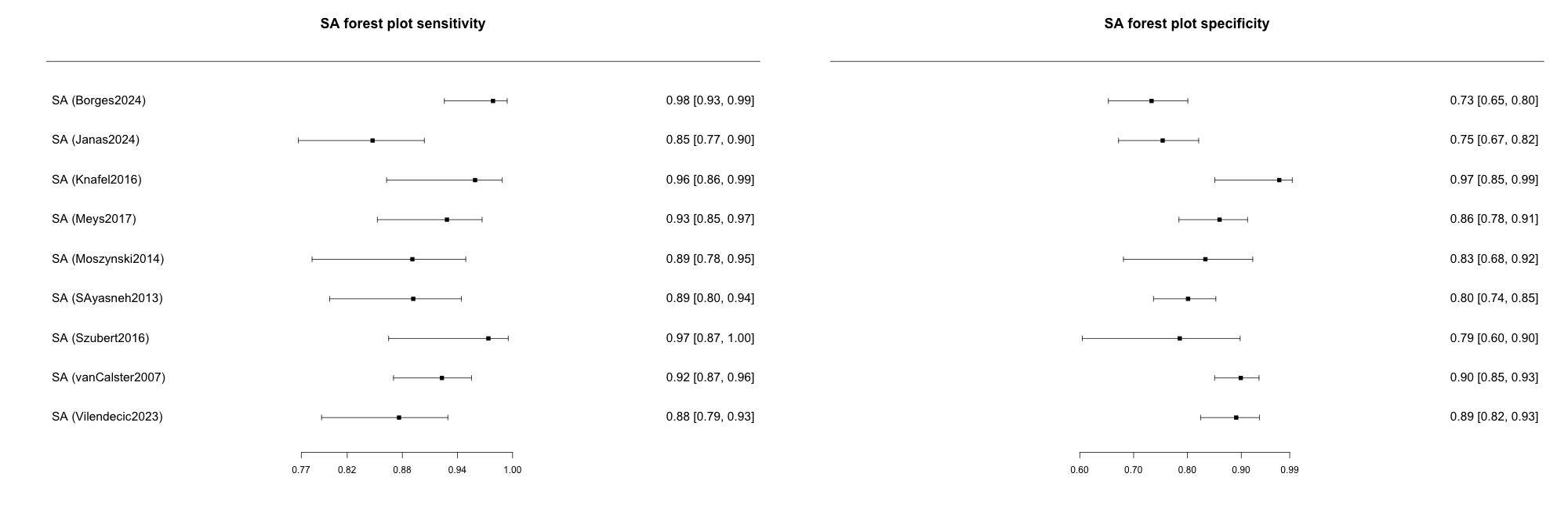


**Figure S7** Summary point estimates of sensitivity and specificity and hierarchical summary receiver-operating-characteristics curves for subgroups of pre- and postmenopausal women

*S7.1, Summary point estimates of sensitivity and specificity and HSROC curves for subgroup of premenopausal women*

*
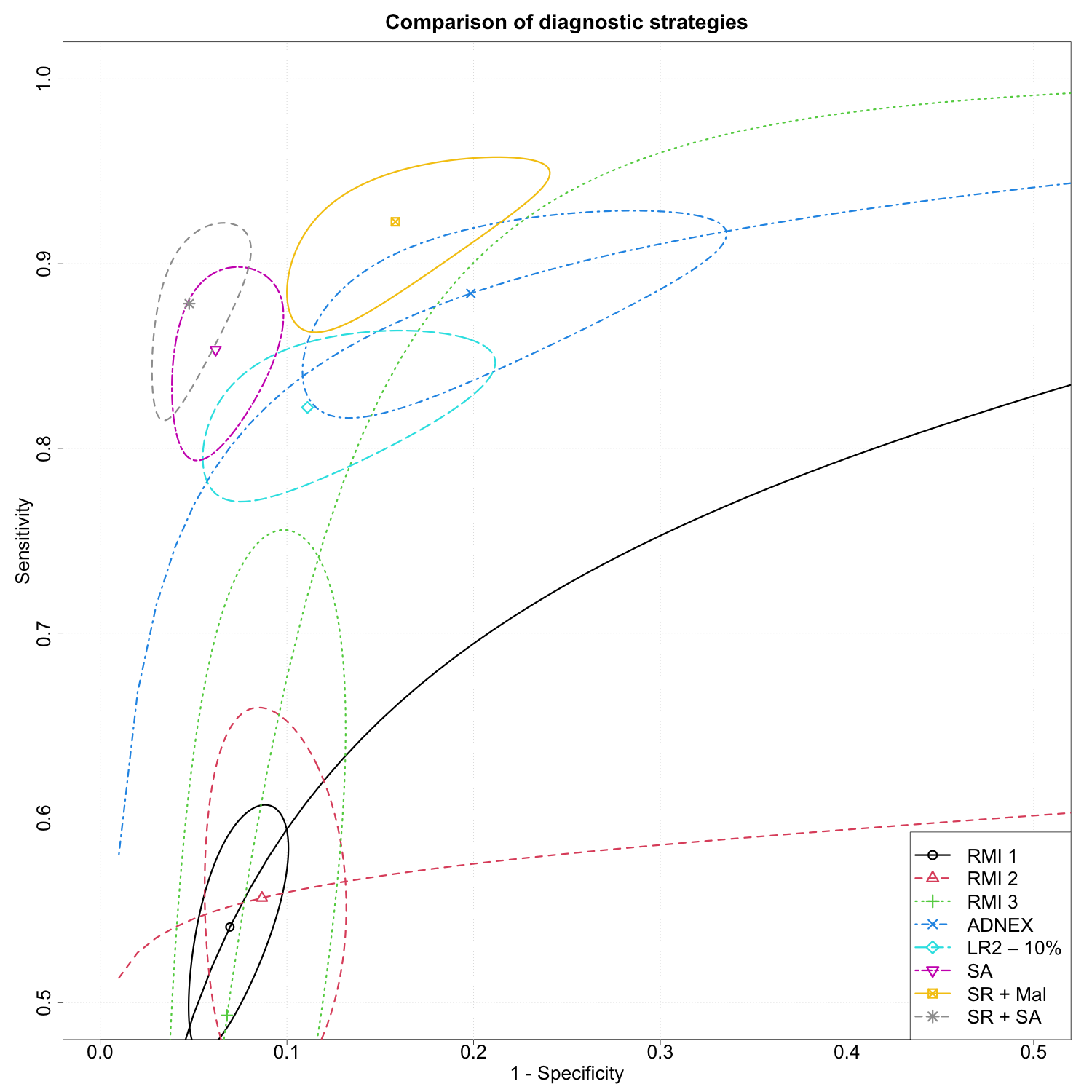
*

*RMI, Risk of Malignancy Index; LR2, Logistic Regression model 2; ADNEX, Assessment of Different NEoplasias in the adneXa; SA, Subjective Assessment; SR, Simple Rules*

*S7.2, Summary point estimates of sensitivity and specificity and HSROC curves for subgroup of postmenopausal women*

*
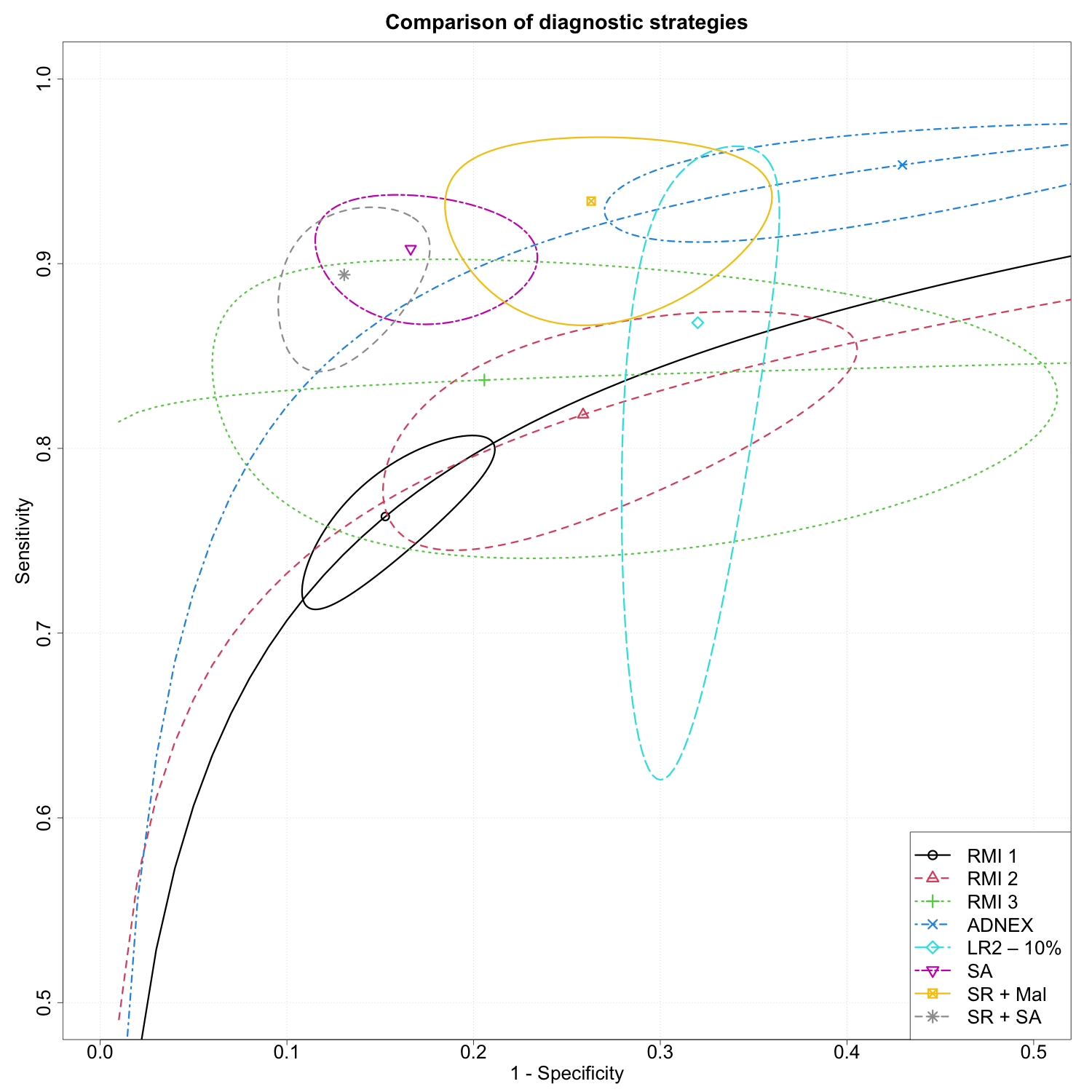
*

*RMI, Risk of Malignancy Index; LR2, Logistic Regression model 2; ADNEX, Assessment of Different NEoplasias in the adneXa; SA, Subjective Assessment; SR, Simple Rules*

**Figure S8** Sensitivity, specificity and prevalence of ovarian cancer in included studies

*S8.1 Sensitivity and prevalence of included studies*

*
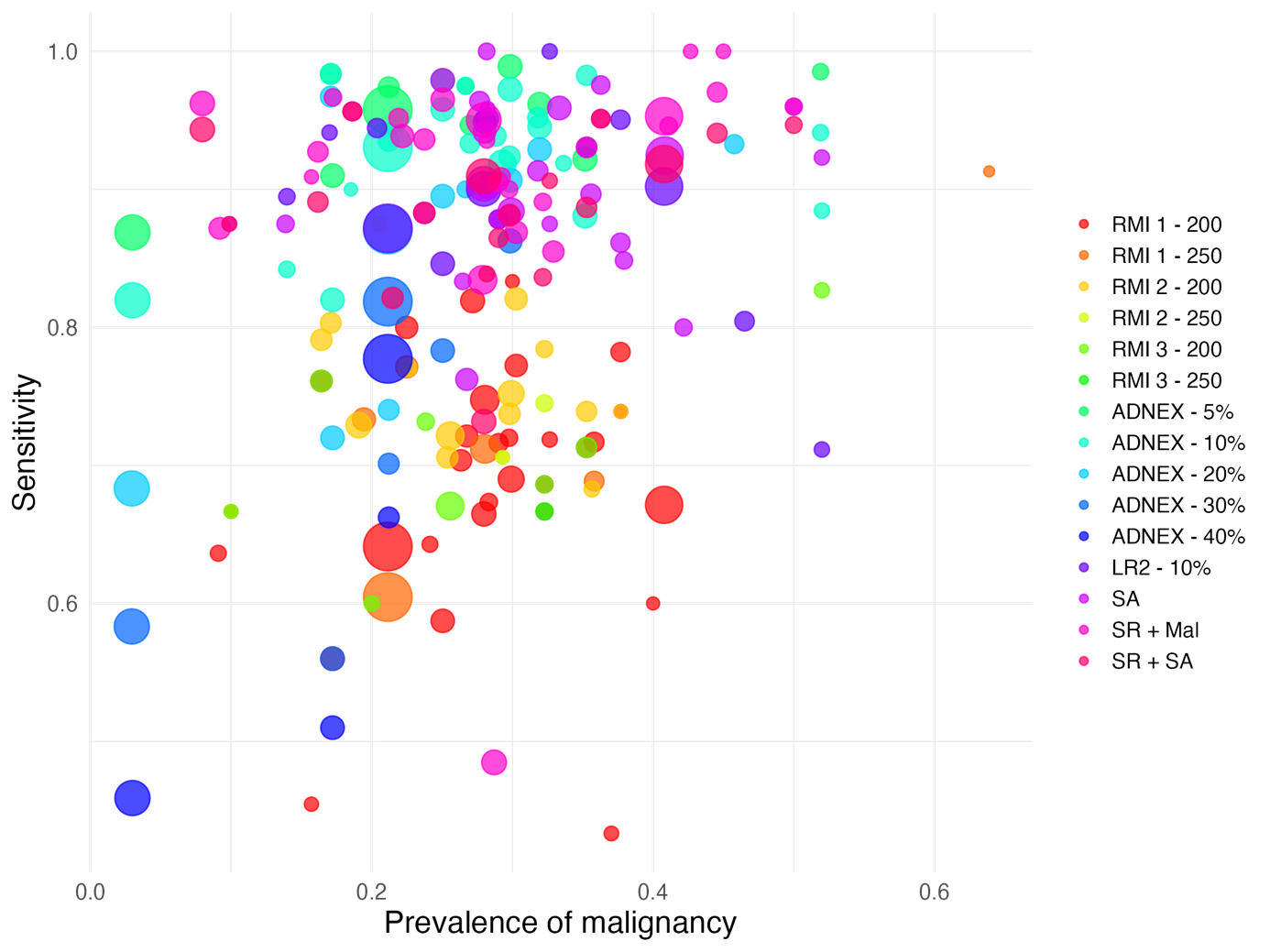
*

*S8.2 Specificity and prevalence of included studies*


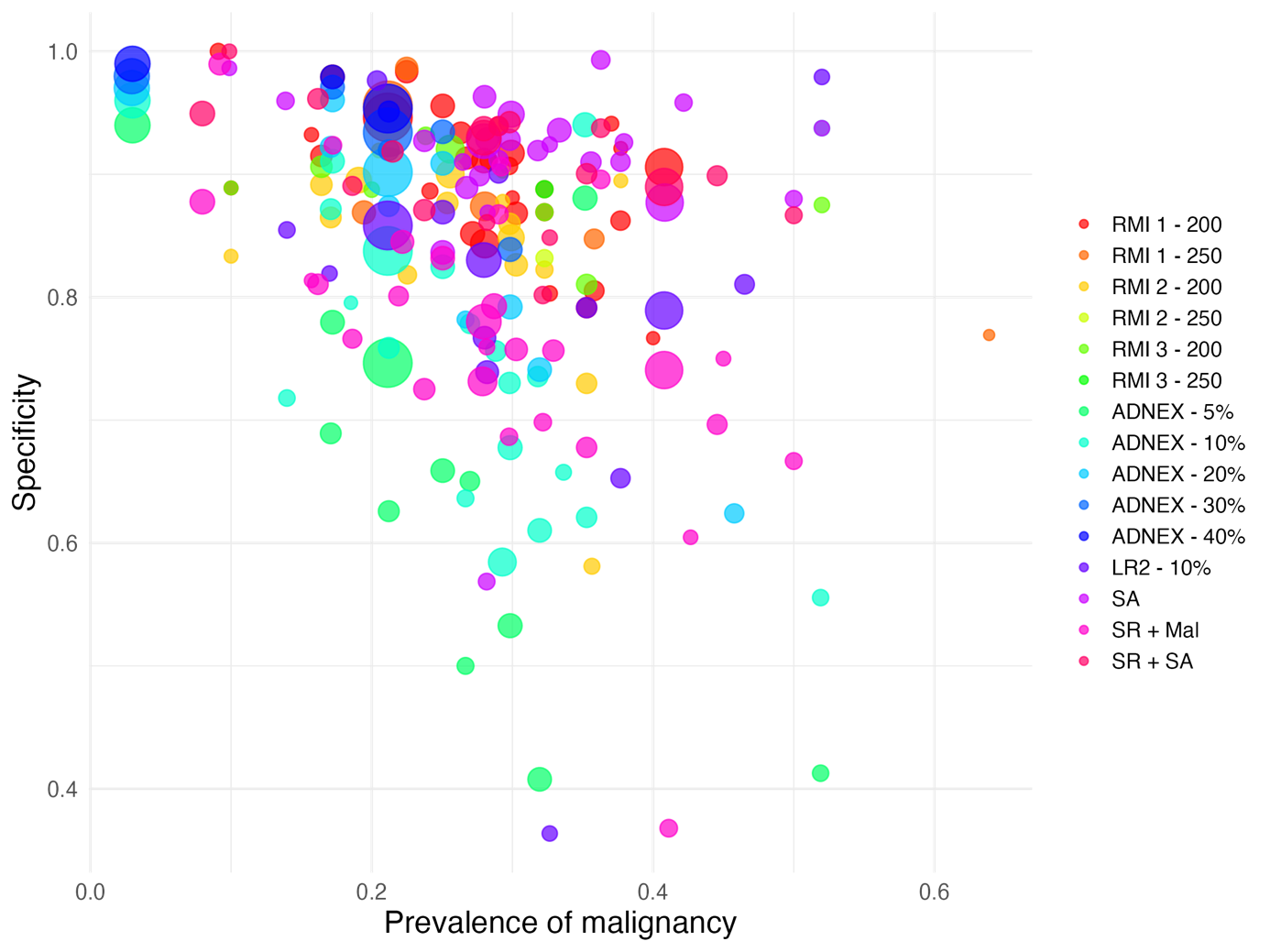


**Figure S9** Forest plots with individual study results for studies with low prevalence (<21.1%) of ovarian cancer

*S9.1, Forest plot RMI 1 – 200, studies with low prevalence*

*
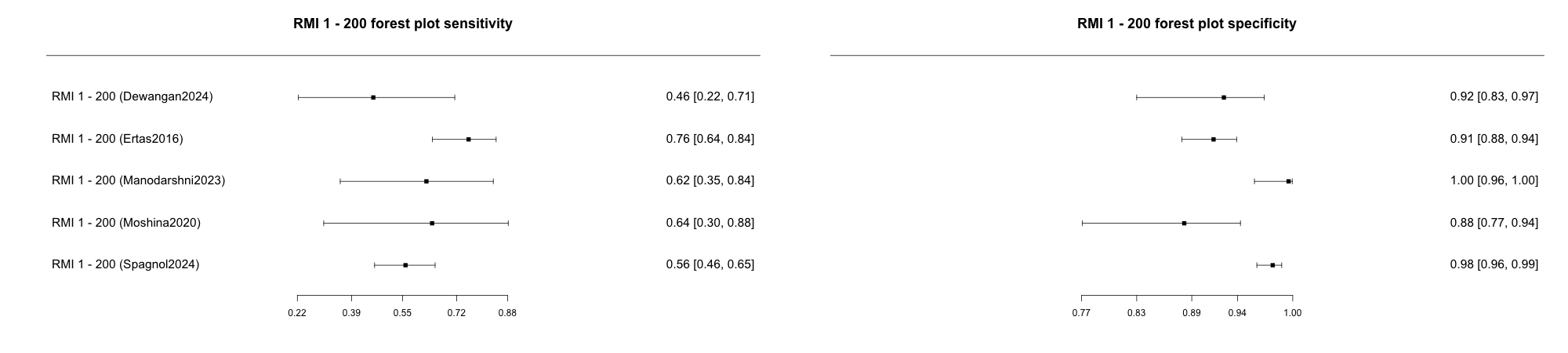
*

*S9.2, Forest plot RMI 1 – 250, studies with low prevalence*

*
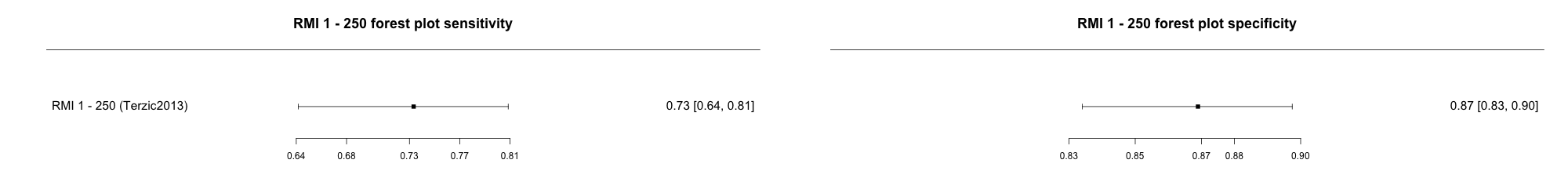
*

*S9.3 Forest plot RMI 2 – 200, studies with low prevalence*

*
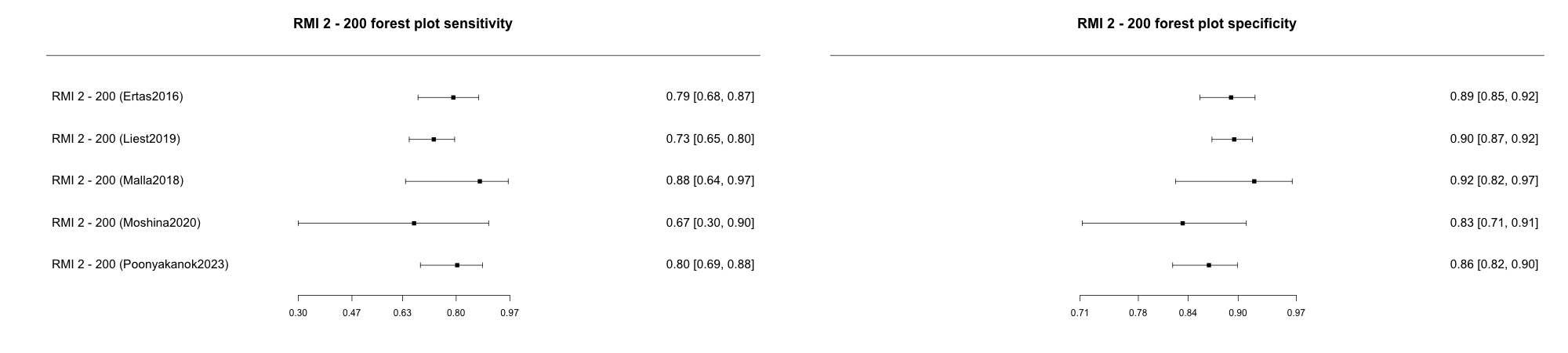
*

*S9.4, Forest plot RMI 3 – 200, studies with low prevalence*

*
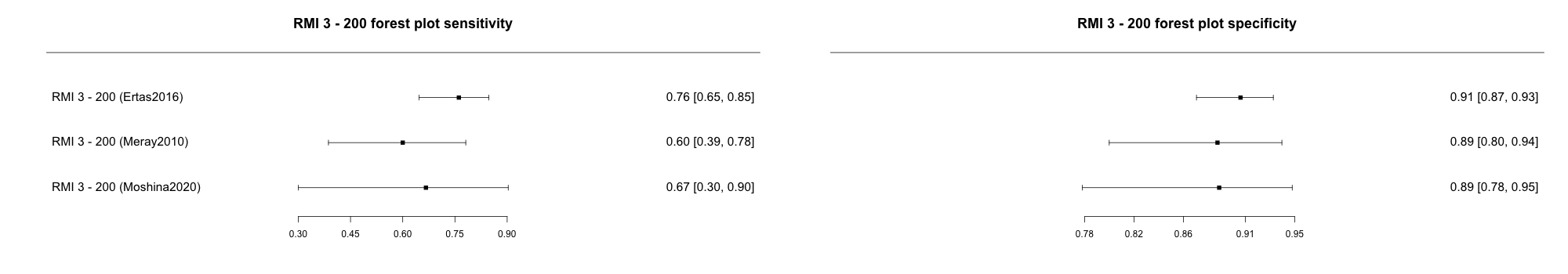
*

*S9.5, Forest plot LR2 – 10%, studies with low prevalence*

*
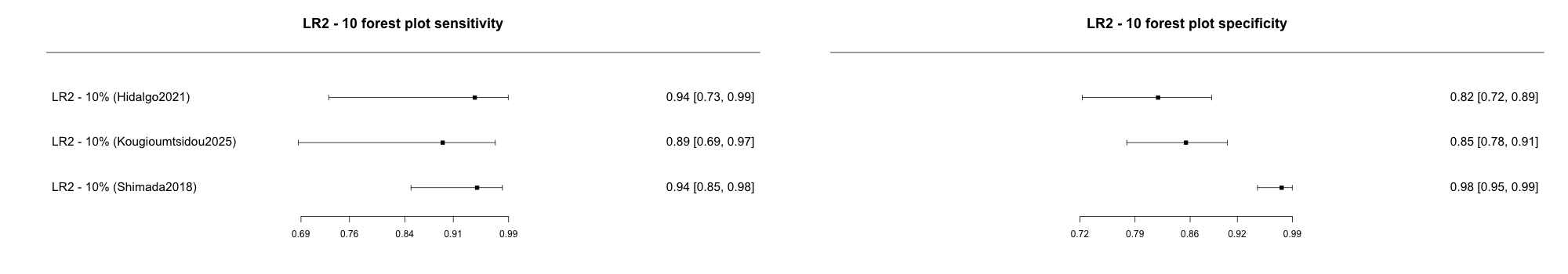
*

*S9.6, Forest plot Simple Rules + Malignant, studies with low prevalence*

*
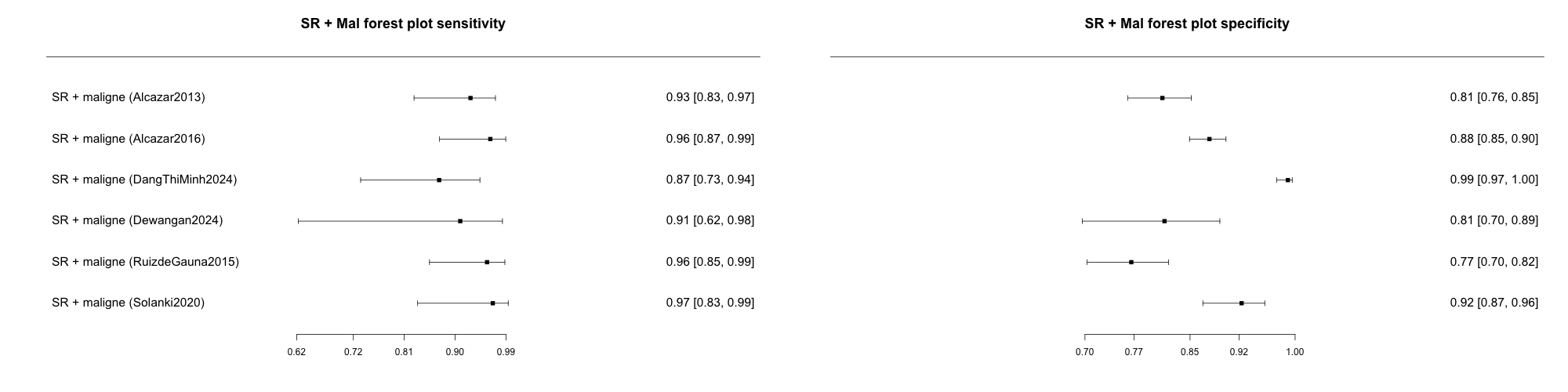
*

*S9.7, Forest plot Simple Rules + Subjective Assessment, studies with low prevalence*

*
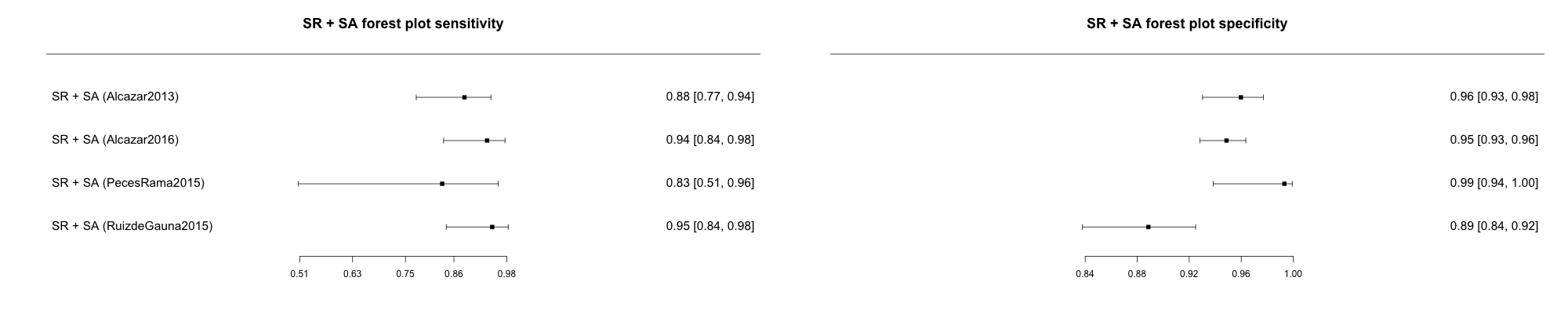
*

*S9.8, Forest plot ADNEX – 5%, studies with low prevalence*

*
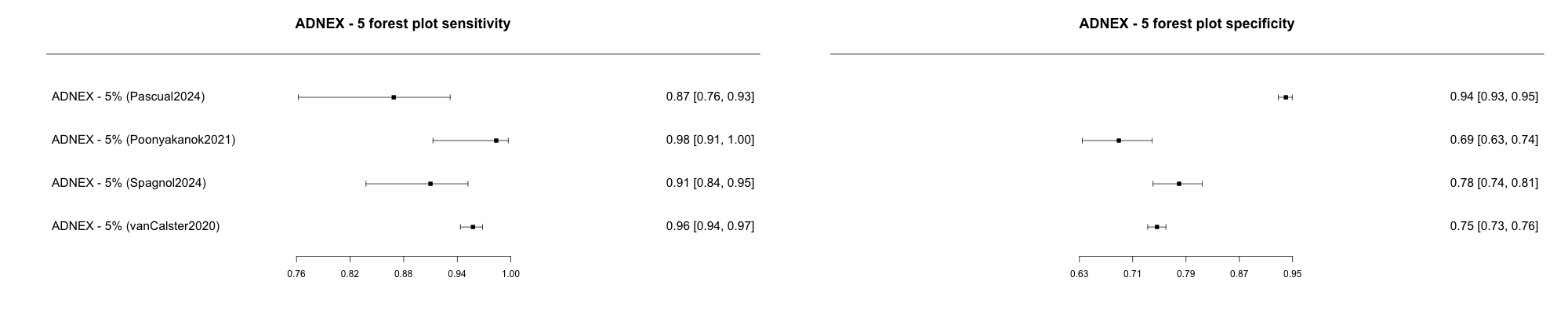
*

*S9.9, Forest plot ADNEX – 10%, studies with low prevalence*

*
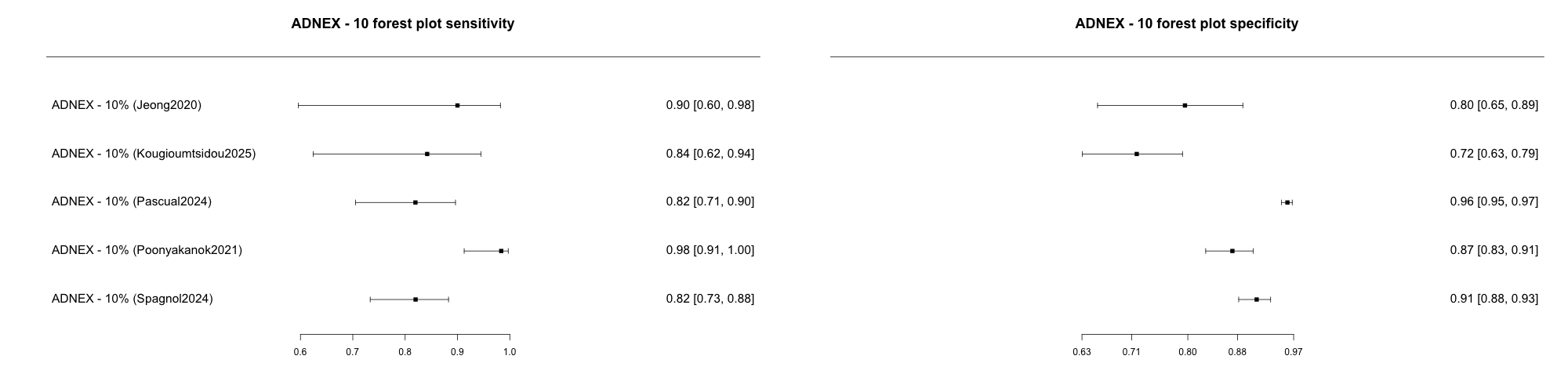
*

*S9.10, Forest plot ADNEX – 20%, studies with low prevalence*

*
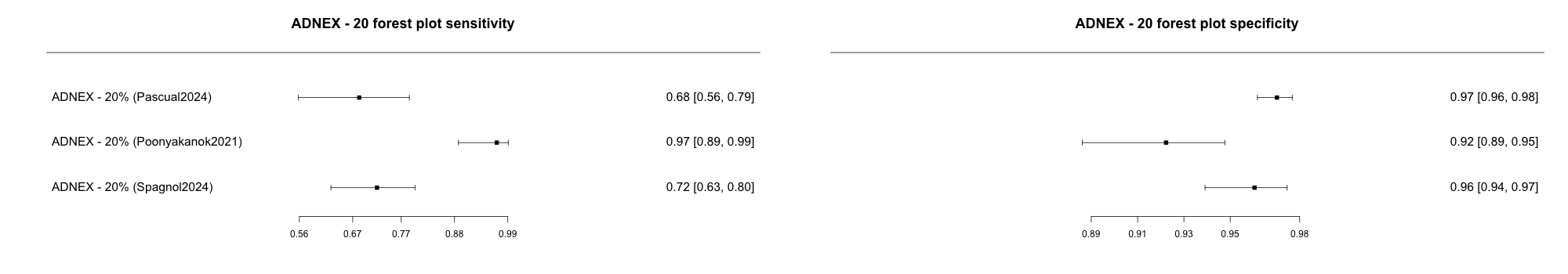
*

*S9.11 Forest plot ADNEX – 30%, studies with low prevalence*

*
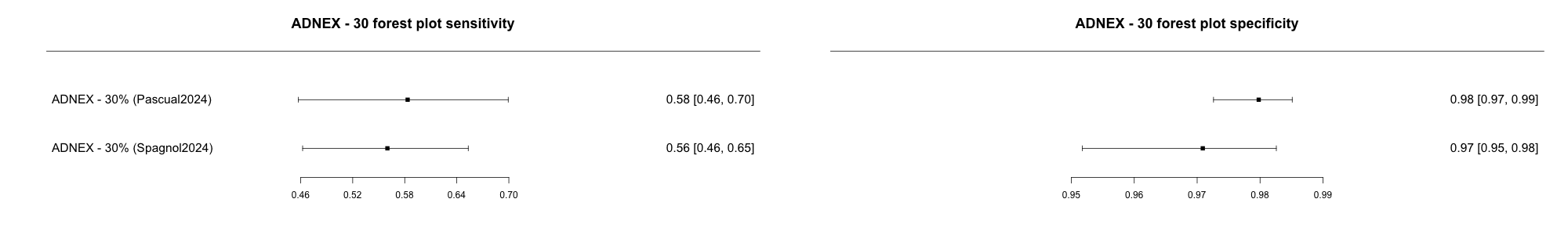
*

*S9.12, Forest plot ADNEX – 40%, studies with low prevalence*

*
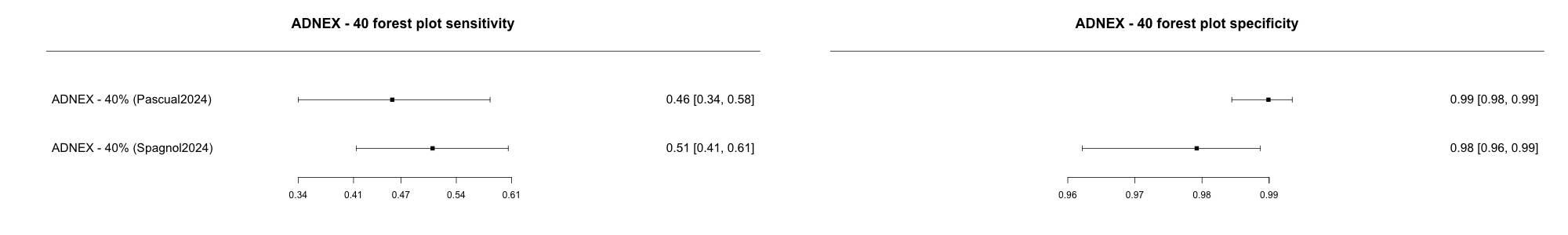
*

*S9.13, Forest plot Subjective Assessment, studies with low prevalence*


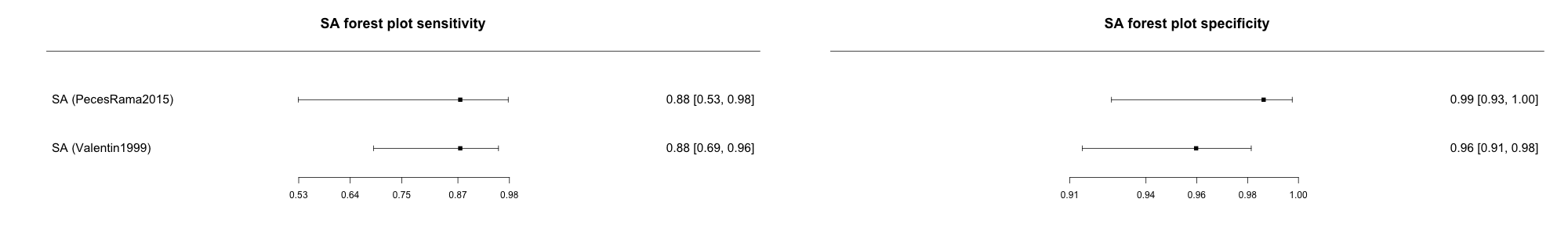


**Figure S10** Forest plots with individual study results for studies with high prevalence (≥21.1%) of ovarian cancer

*S10.1, Forest plot RMI 1 – 200, studies with high prevalence*


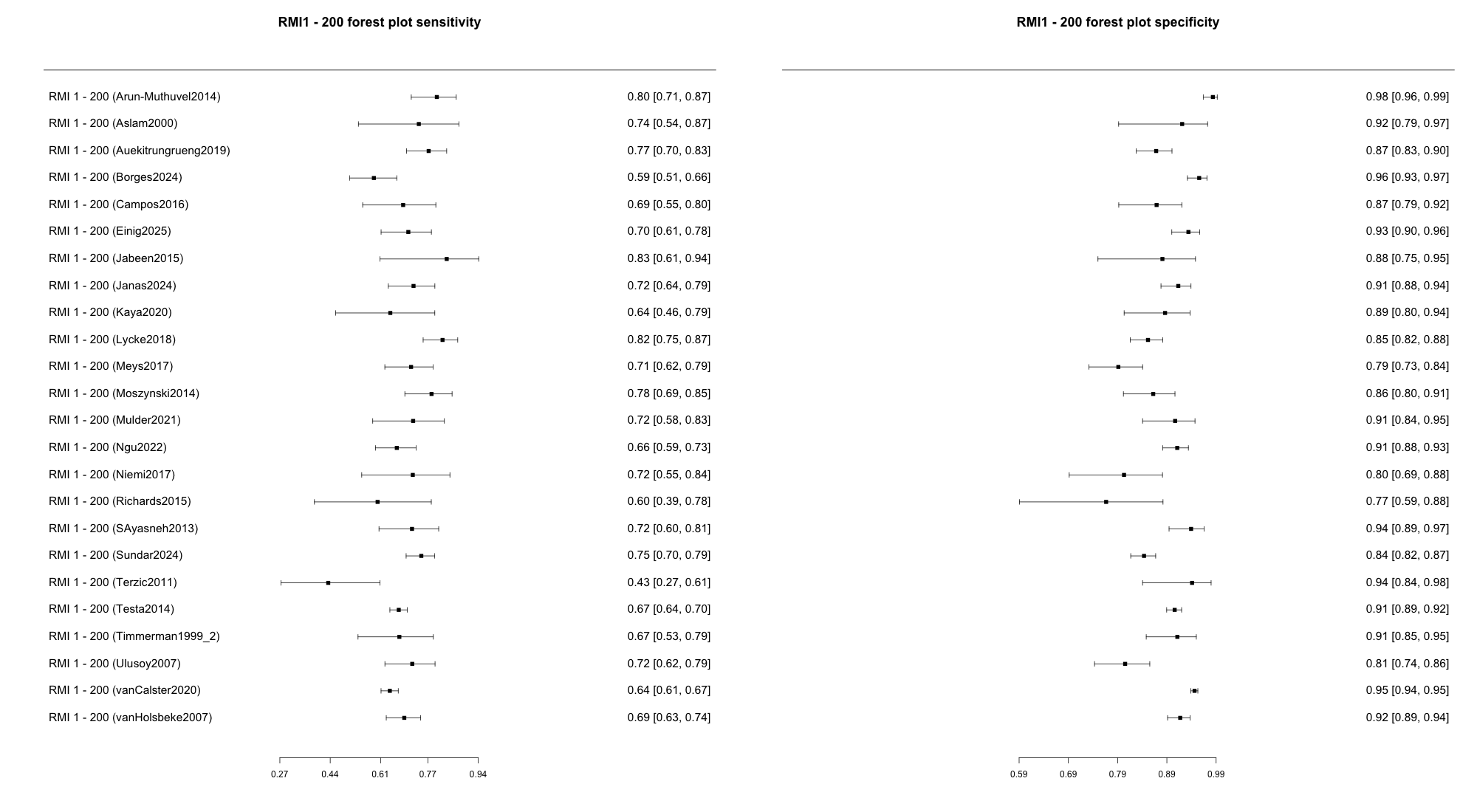


*S10.2, Forest plot RMI 1 – 250, studies with high prevalence*

*
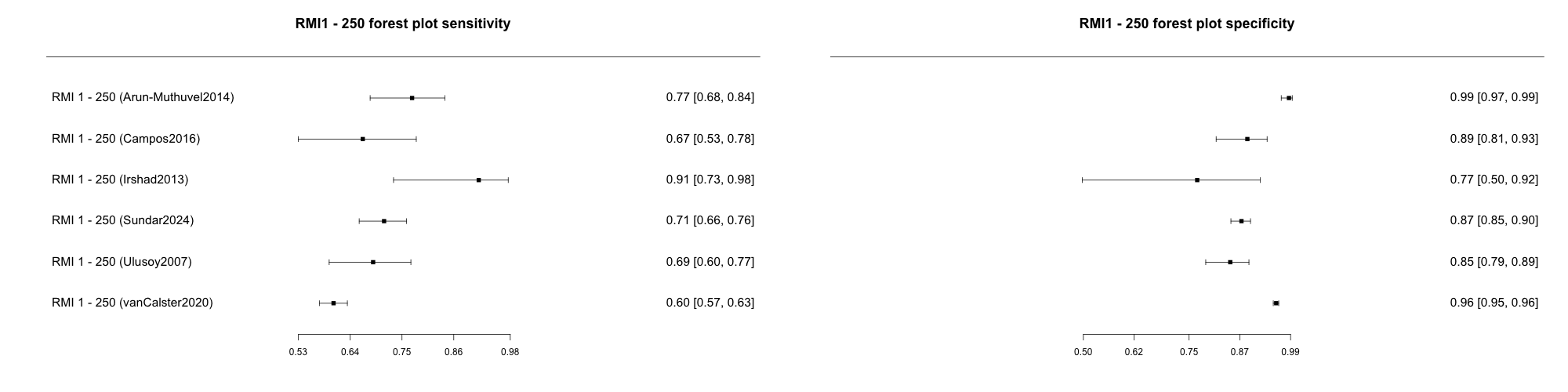
*

*S10.3 Forest plot RMI 2 – 200, studies with high prevalence*

*
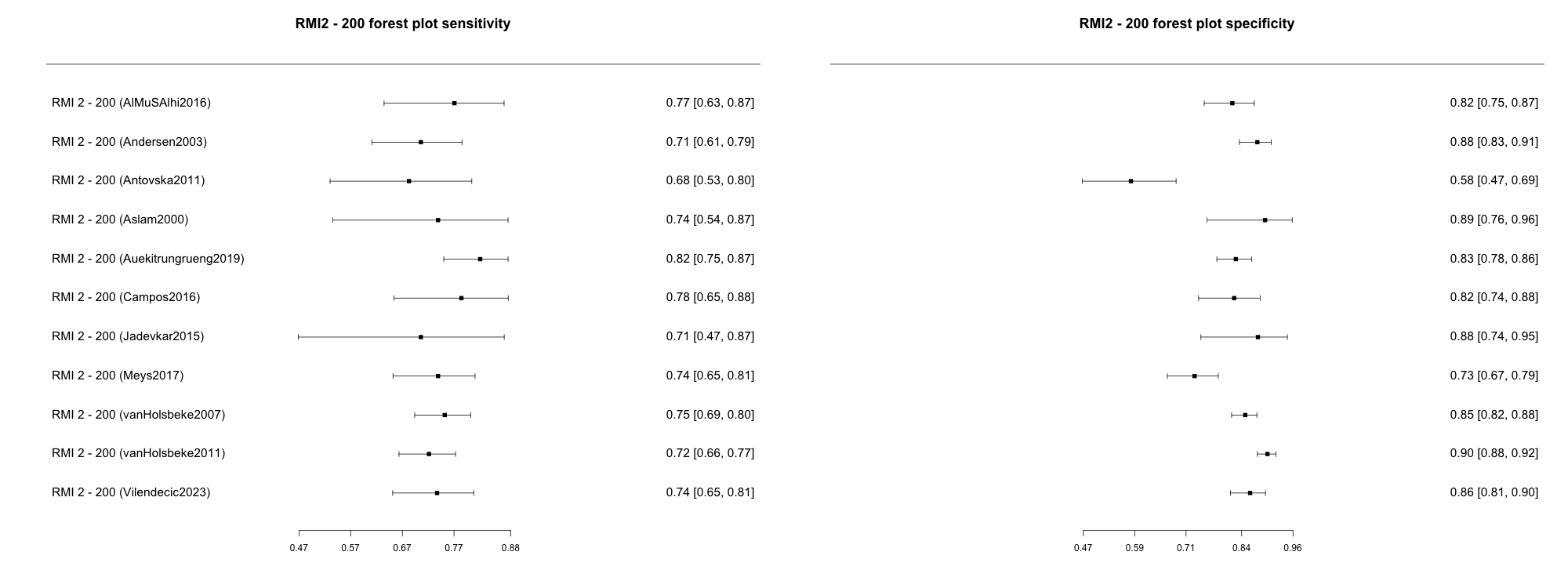
*

*S10.4 Forest plot RMI 2 – 250, studies with high prevalence*

*
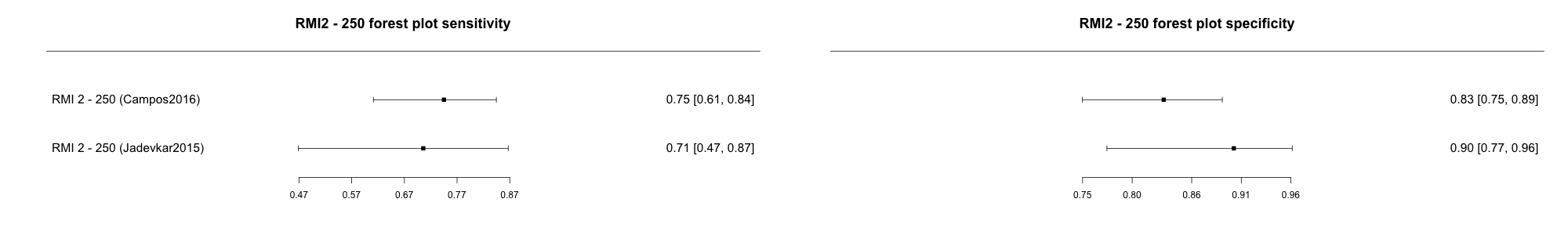
*

*S10.5, Forest plot RMI 3 – 200, studies with high prevalence*

*S10.6, Forest plot RMI 3 – 250, studies with high prevalence*

*S10.7, Forest plot LR2 – 10%, studies with high prevalence*

*S10.8, Forest plot Simple Rules + Malignant, studies with high prevalence*

*S10.9, Forest plot Simple Rules + Subjective Assessment, studies with high prevalence*

*S10.10, Forest plot ADNEX – 5%, studies with high prevalence*

*S10.11, Forest plot ADNEX – 10%, studies with high prevalence*

*S10.12, Forest plot ADNEX – 20%, studies with high prevalence*

*S10.13, Forest plot ADNEX – 30%, studies with high prevalence*

*S10.14, Forest plot ADNEX – 40%, studies with high prevalence*

*S10.15, Forest plot Subjective Assessment, studies with high prevalence*

**Figure S11** Summary point estimates of sensitivity and specificity and hierarchical summary receiver-operating-characteristics curves for studies with low (<21.1%) vs high (≥21.1%) prevalence of ovarian cancer

*S11.1, Summary point estimates of sensitivity and specificity and HSROC curves for studies with low (<21.1%) prevalence*

*RMI, Risk of Malignancy Index; LR2, Logistic Regression model 2; ADNEX, Assessment of Different NEoplasias in the adneXa; SA, Subjective Assessment; SR, Simple Rules*

*S11.2, Summary point estimates of sensitivity and specificity and HSROC curves for studies with high (≥21.1%) prevalence*

*RMI, Risk of Malignancy Index; LR2, Logistic Regression model 2; ADNEX, Assessment of Different NEoplasias in the adneXa; SA, Subjective Assessment; SR, Simple Rules*
